# Guanidine–Amide-Catalyzed Aza-Henry Reaction of Isatin-Derived Ketimines: Origin of Selectivity and New Catalyst Design

**DOI:** 10.3390/molecules26071965

**Published:** 2021-03-31

**Authors:** Jiajia He, Dianyong Tang, Changwei Hu, Zhishan Su

**Affiliations:** 1 Key Laboratory of Green Chemistry and Technology, Ministry of Education, College of Chemistry, Sichuan University, Chengdu 610064, China; helanjiajia@stu.scu.edu.cn (J.H.); changweihu@scu.edu.cn (C.H.); 2 College of Pharmacy & International Academy of Targeted Therapeutics and Innovation, Chongqing University of Arts and Sciences, Chongqing 402160, China; tdy@cqwu.edu.cn

**Keywords:** aza-Henry reaction, ketimine, guanidine–amide, DFT calculation, mechanistic investigation, catalyst design

## Abstract

Density functional theory (DFT) calculations were performed to investigate the mechanism and the enantioselectivity of the aza-Henry reaction of isatin-derived ketimine catalyzed by chiral guanidine–amide catalysts at the M06-2X-D3/6-311+G(d,p)//M06-2X-D3/6-31G(d,p) (toluene, SMD) theoretical level. The catalytic reaction occurred via a three-step mechanism: (i) the deprotonation of nitromethane by a chiral guanidine–amide catalyst; (ii) formation of C–C bonds; (iii) H-transfer from guanidine to ketimine, accompanied with the regeneration of the catalyst. A dual activation model was proposed, in which the protonated guanidine activated the nitronate, and the amide moiety simultaneously interacted with the ketimine substrate by intermolecular hydrogen bonding. The repulsion of CPh_3_ group in guanidine as well as *N*-Boc group in ketimine raised the Pauli repulsion energy (∆*E*_Pauli_) and the strain energy (∆*E*_strain_) of reacting species in the unfavorable *si*-face pathway, contributing to a high level of stereoselectivity. A new catalyst with cyclopropenimine and 1,2-diphenylethylcarbamoyl as well as sulfonamide substituent was designed. The strong basicity of cyclopropenimine moiety accelerated the activation of CH_3_NO_2_ by decreasing the energy barrier in the deprotonation step. The repulsion between the *N*-Boc group in ketimine and cyclohexyl group as well as chiral backbone in the new catalyst raised the energy barrier in C–C bond formation along the *si*-face attack pathway, leading to the formation of *R*-configuration product. A possible synthetic route for the new catalyst is also suggested.

## 1. Introduction

The aza-Henry reaction (or nitro-Mannich reaction) is one of the most effective methods for the construction of carbon–carbon bonds with concomitant generation of two vicinal stereogenic centers bearing nitro and amino functional groups [1]. The resulting β-nitroamine products can be conveniently converted into a variety of chiral building blocks (e.g., α-amino acids [2,3,4,5,6] and 1,2-diamines [7,8,9,10]); therefore, the development of efficient procedures to promote the reaction in a highly enantioselective way have received wide attention. Since the first example of a catalytic enantioselective aza-Henry reaction was reported by Shibasaki [11], many metal-based [12,13,14,15] and organic catalysts [16,17] have been employed in the nucleophilic addition of aldimine, affording β-nitroamines in high yield and stereoselectivity [18]. Compared to aldimine substrate, the reports using a ketimine as the electrophile in the asymmetric Henry reaction were limited, due to its low reactivity and poor prochiral face control [19].

The aza-Henry reaction of isatin-derived *N*-Boc ketimine could produce 3-substituted-3-amino-2-oxindoles, which are key structural units in many natural products and pharmacologically active molecules [20,21,22,23]. The bis(imidazolidine) pyridine-NiCl_2_ [24], bis-oxazoline-Cu(II) [25] and *N,N*′-dioxide-Cu(I) [26] complexes were proven to be efficient organometallic catalysts for aza-Henry reaction of isatin-derived ketimines under mild conditions. In organocatalysis, the cinchona alkaloid and its derivatives are popular catalysts for the construction of indole skeletons [27]. A dual activation model is generally adopted to explain catalyst–ketimine interactions as well as the stereoinduction of catalysts [28], in which the tertiary amine group and a hydrogen-bond donor group orientated on the chiral scaffold activate CH_3_NO_2_ and govern facial selectivity in the reaction simultaneously. For example, Chimni et al. proposed that the OH group at C6′-position of the cinchona alkaloid affected the reactivity and selectivity of the product. In the catalysis, the isatin ketimine bonded to the C6′–OH group of the catalyst by hydrogen bonding. Meanwhile, the quinuclidine tertiary amine moiety deprotonated the nitromethane and induced the formed nitronate to attack the ketimine from the *re*-prochiral face [29]. Chiral thiourea [30] and thiourea- [31] or sulphone-amide-modified cinchona derivatives [32] are also applied into aza-Henry reactions of ketimine. They serve to simultaneously activate the electrophile and nucleophile during C–C bond formation by hydrogen bonding, and are essential for reaction rate and selectivity. Dixon and co-workers designed a bifunctional iminophosphorane organocatalysts to enhance the synergistic effects of Brønsted base and H-bond-donor in the addition of nitromethane to unreactive ketimine [33]. Chen and co-workers theoretically studied the mechanism of quinine derivative-catalyzed aza-Henry reaction of isatin-derived ketimine using the density functional theory (DFT) method. The synergistic effects of multiple non-covalent interactions (i.e., classical, and non-classical H bonds and anion–π interactions) were responsible for the high reactivity and enantioselectivity of reactions [34]. In addition, chiral phase transfer catalysts with a quaternary ammonium center derived from l-tert Leucine also exhibited good catalytic performance in the aza-Henry reaction of isatin-derived ketimines. The urea motif captured the isatin-derived ketimines by H-bond interactions. Meanwhile, the nucleophilic nitro group anion paired with ammonium motifs by electrostatic interactions [35].

Due to strong basicity and hydrogen-bond donor ability, chiral guanidine organocatalysts have been widely used in many asymmetric catalysis (e.g., Michael reaction [33], Henry reaction [36], and Mannich reaction [37]). It can abstract a proton from the substrate and catalyze the reaction as its conjugated acid (i.e., the guanidinium cation). A bifunctional Brønsted acid activation mode has been proposed, in which the electrophile and nucleophile are positioned by a guanidinium cation via hydrogen bonding [38,39,40,41,42,43]. In 2015, Feng and Liu [44] developed an open-chain guanidine–amide organocatalyst for asymmetric aza-Henry reactions of isatin-derived *N*-Boc ketimines. The 3-substituted 3-amino-2-oxindoles were obtained with excellent results (up to 99% yield, 94% ee). They proposed that the guanidine moiety deprotonated the nitromethane and bonded the nucleophile through dual hydrogen-bonds. The amide unit acted as a Brønsted acid to activate the ketimine substrate simultaneously. The shielding effect from phenyl groups of the amide unit induced the nucleophile to preferably attack the ketimine from the *re*-face, affording an *R*-configuration product.

**Scheme 1:**
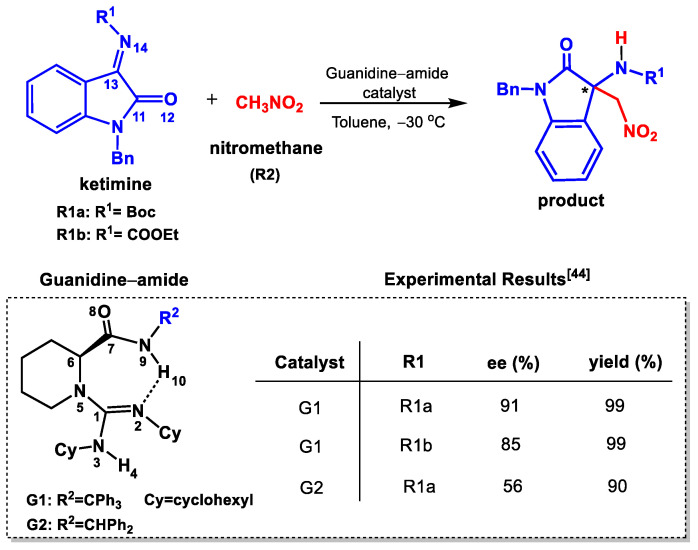
Asymmetric aza-Henry reaction of isatin-derived *N*-protected ketimines (R1) and nitromethane (R2) catalyzed by guanidine–amide catalysts.

Herein, we performed a theoretical investigation on the mechanisms and stereoselectivity of asymmetric aza-Henry reactions between isatin-derived *N*-Boc ketimines and nitroalkane (see Scheme 1). Based on the calculations, we modified the structure of guanidine–amide and design a new catalyst containing cyclopropenimine and 1,2-diphenylethylcarbamoyl as well as 2,6-difluorobenzenesulfonamide group to enhance its catalytic activity and chiral induction ability. Our hope was that through the synergistic effects of the stronger Brønsted base and the hydrogen bonding donor as well as the modification of the chiral backbone, superior reactivity and selectivity could be obtained. These results are expected to provide useful information for the synthesis of new chiral organocatalysts for aza-Henry reactions of ketimine.

## 2. Results and Discussion

### 2.1. Analysis of Reactivity at N Atoms in G1

It is well known that guanidine can act as a Brønsted base to activate the reactant by hydrogen bonding in organocatalysis [45,46,47,48]. Three types of nitrogen atoms (N2, N3 and N5) exist in the structural unit of guanidine moiety; therefore, we first optimized the geometry of G1 and evaluated the reactivity of nitrogen atoms. As shown in Table S1, Supplementary Materials, the key structural parameters in G1 were similar to those of the corresponding crystal structure obtained in the experiment [44]. Intramolecular hydrogen bonding exists between the guanidine moiety and amide moiety, with an N2⋯H10 distance of 1.94 Å. The proton affinity of the N2 atom (226.4 kcal·mol^−1^) is higher than those of N3 (196.3 kcal·mol^−1^) and N5 (199.6 kcal·mol^−1^). This indicated that the N2 atom with strong basicity [38,39,40,41,42,43,49] could work as the reacting site for the deprotonation of CH_3_NO_2_ in the Henry reaction.

### 2.2. Catalytic Mechanism for R1a

**Scheme 2:**
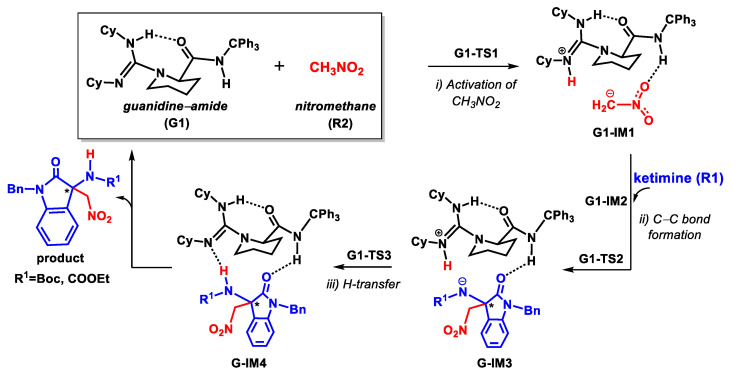
Mechanism of aza-Henry reaction between ketimine (R1a and R1b) and nitromethane (R2), catalyzed by guanidine–amide catalyst (G1).

As shown in Scheme 2, the reaction mechanism of asymmetric Henry reactions between ketimine (R1a) and nitromethane (R2) consisted of three consecutive steps, including: (i) the deprotonation of CH_3_NO_2_ by guanidine–amide catalyst; (ii) formation of a C–C bond; and (iii) H-transfer from the guanidine to ketimine substrate, accompanied with the regeneration of the catalyst. The step associated with the activation of CH_3_NO_2_ was predicted to be the rate-determining step (RDS) [18]. The C–C bond formation step was the chiral-controlling step, affording *R*- or *S*-enantiomers along *re*- or *si*-face attack pathways, alternatively.

#### 2.2.1. Activation of CH_3_NO_2_

**Figure 1:**
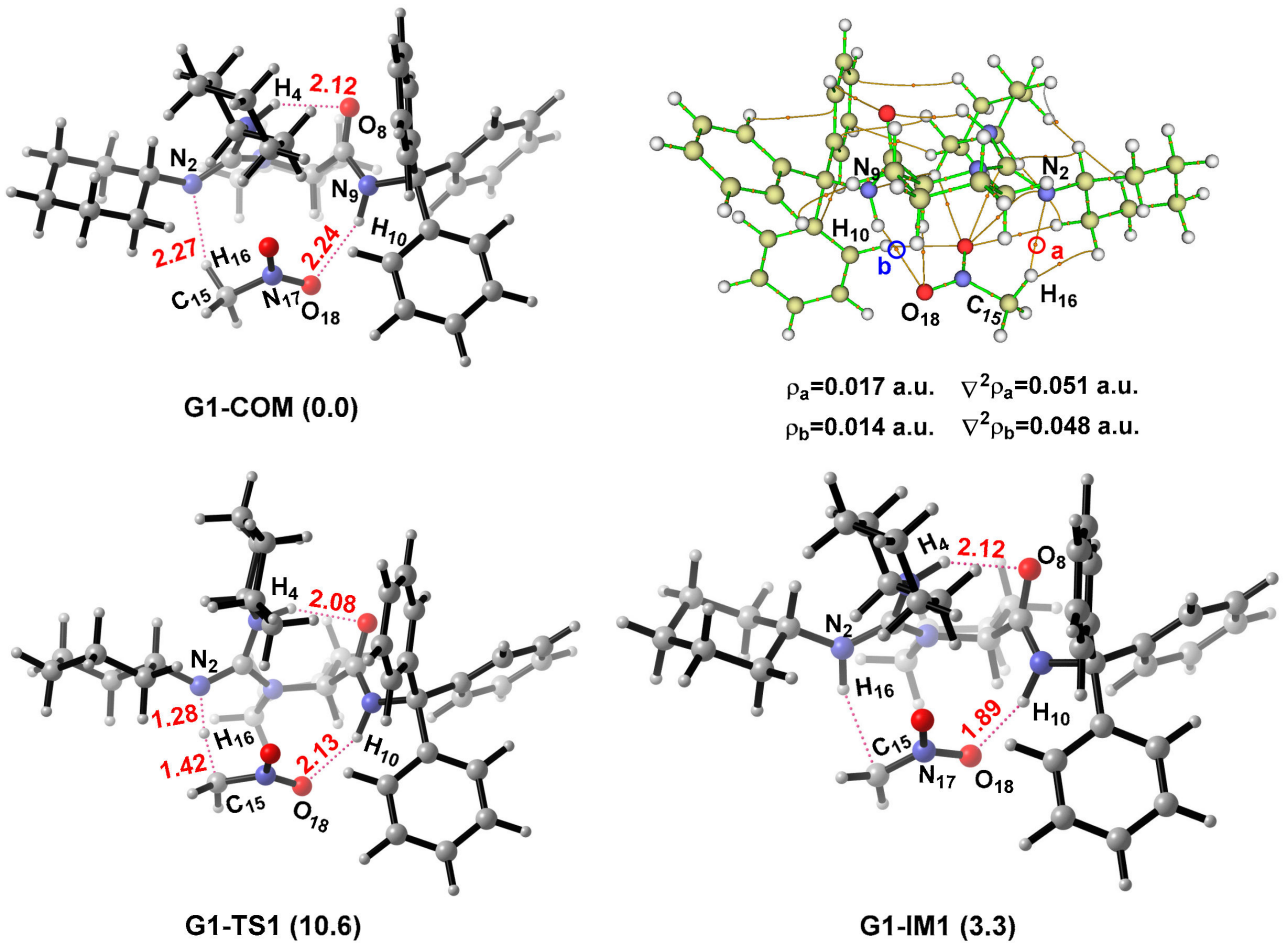
Optimized structures of G1-COM, G1-TS1 and G1-IM1 and their relative Gibbs free energies (in kcal·mol^−1^). The distances are in Å. Laplacian (∇^2^*ρ*) and electronic density (*ρ*) values of the selected bond critical points (BCP) in G1-COM were obtained by atom in molecule (AIM) analysis.

When G1 interacted with CH_3_NO_2_, it underwent a change of conformation, constructing O8⋯H4 intramolecular hydrogen bonding. This new conformation facilitated the N2 atom and amide moiety to interact with CH_3_NO_2_ simultaneously by intermolecular hydrogen bonding [50]. For the molecular complex G1-COM, the N2⋯H16 and H10⋯O18 distances were 2.27 Å and 2.24 Å, respectively. AIM analysis indicated that the electronic densities (*ρ*) at the (3, −1) bond critical points (BCP) (a and b in Figure 1) were 0.017 a.u. and 0.014 a.u., with the corresponding hydrogen bonding energies of *E*_a_ = −2.99 kcal·mol^−1^ and *E*_b_ = −2.27 kcal·mol^−1^, respectively [51]. Compared to the free CH_3_NO_2_, the C15–H16 bond in G1-COM enlengthened from 1.09 to 1.10 Å. Meanwhile, the corresponding Wiberg bond index was decreased from 0.921 to 0.875. These results suggested that the C–H bond in CH_3_NO_2_ was significantly weakened. The deprotonation of CH_3_NO_2_ occurred via transition state G1-TS1, accompanied with the global electron density transfer (GEDT) of 0.23 e from G1 to CH_3_NO_2_. Accordingly, an ion-pair intermediate (G1-IM1) was formed. This step was endergonic by 3.3 kcal·mol^−1^, with an energy barrier of 10.6 kcal·mol^−1^.

#### 2.2.2. Formation of C–C Bond

**Scheme 3:**
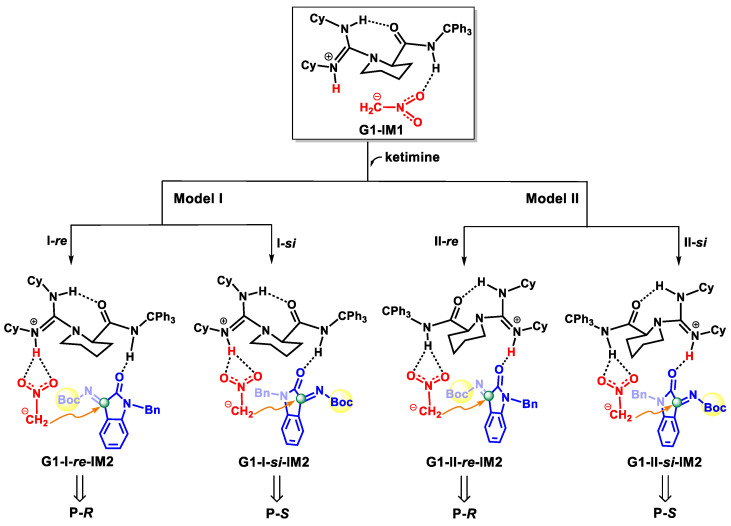
Four possible reaction pathways along *re*- or *si*-face attacks to obtain the *R*- or *S*-configuration products, respectively.

**Figure 2:**
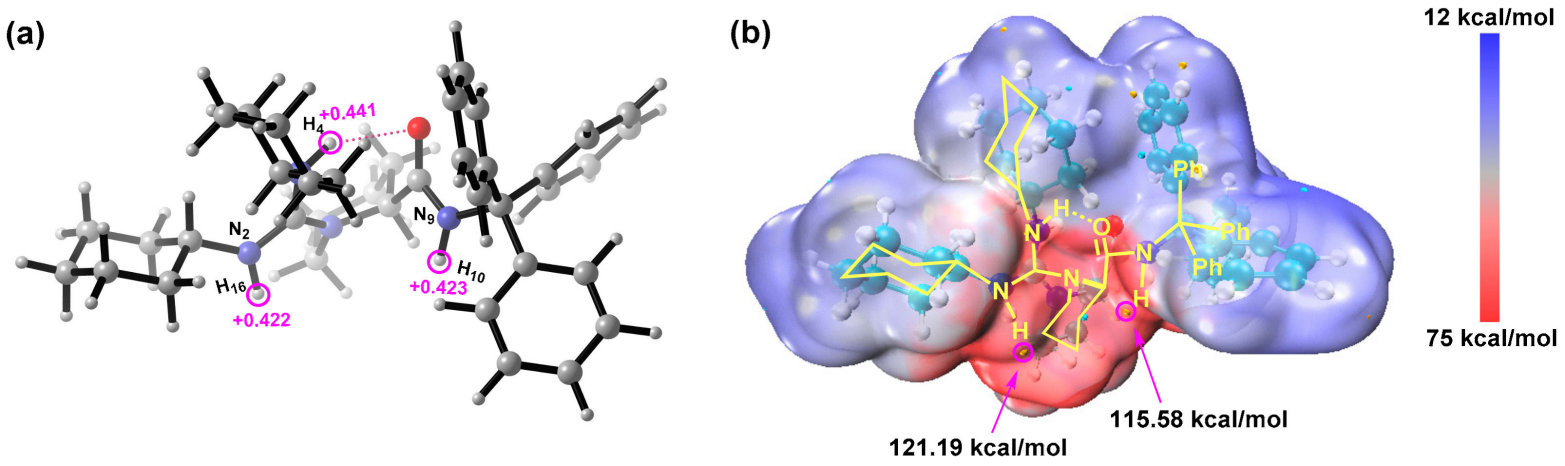
(**a**) The natural atom charges at H atoms in guanidine cation species, obtained by natural bond orbital (NBO) analysis. (**b**) Electrostatic potential (ESP) mapped Van der Waals surface of protonated G1. The redder the color, the more positive the ESP. The purple and red spheres represented the local minima and maxima points, respectively.

**Figure 3:**
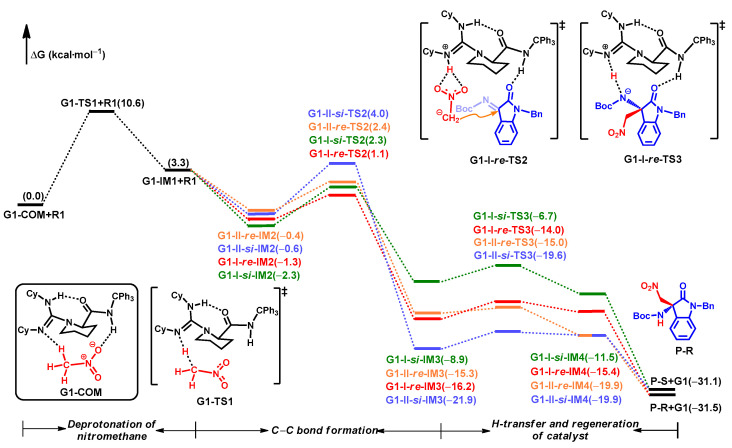
Energy profiles for aza-Henry reaction between *N*-Boc ketimine (R1a) and nitromethane (R2) catalyzed by guanidine (G1) along *re*- and *si*-face pathways in models I and II, respectively.

**Figure 4:**
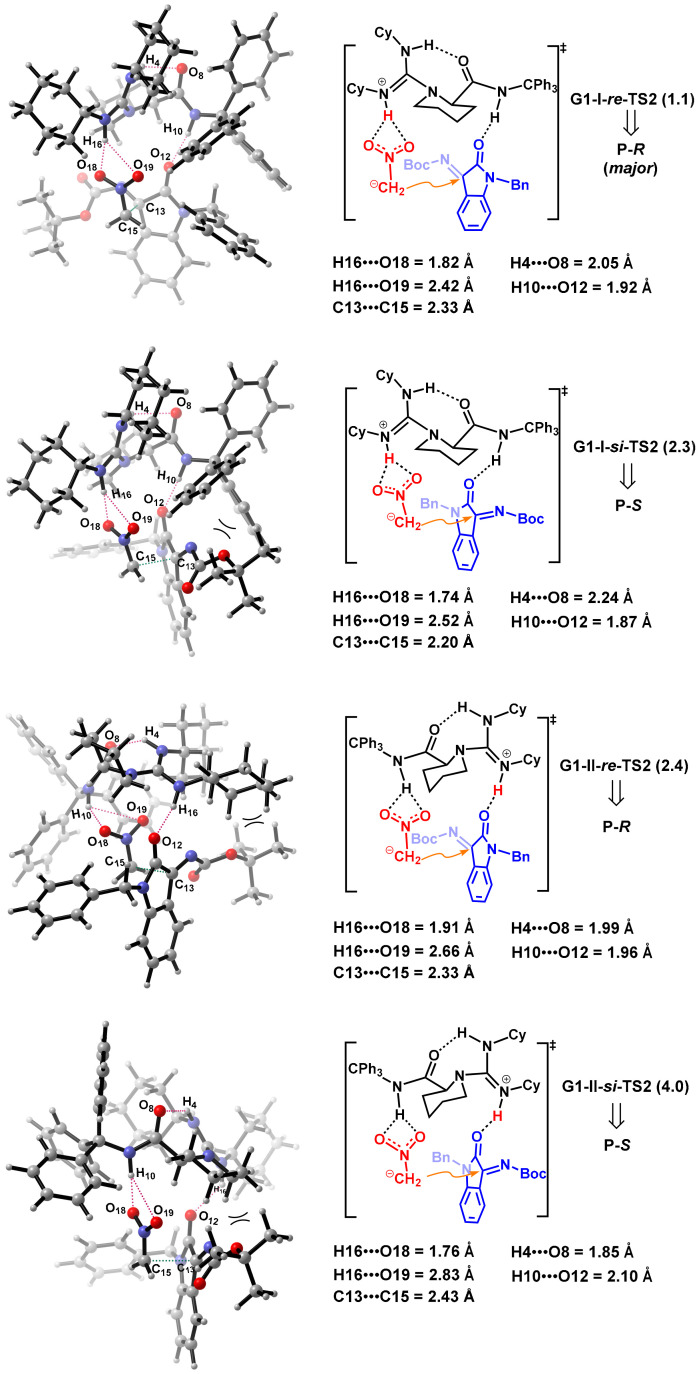
Optimized geometries of four transition states along *re*- or *si*-face attack pathways. The relative Gibbs free energies are given in kcal·mol^−1^.

Starting from G1-IM1, four molecular complexes (G1-I-*re*-IM2 ~ G1-II-*si*-IM2) were formed in models I and II, according to the different orientations of *N*-Boc ketimine (R1a) and nitronate (Scheme 3). The hydrogen bonding between the protonated guanidine with R1a and nitronate were verified by AIM analysis because the magnitudes of Laplacian of electron densities (∇^2^*ρ*) at (3, −1) bond critical points (a and b) were positive (Figure S1). Electrostatic potential (ESP) analysis (Figure 2b) for the protonated guanidium fragment in G1-IM1 indicated that the surface global maximum (121.2 kcal·mol^−1^) was from the positively charged H16 atom (not H10 atom in Figure 2a), leading to preferable orientation of the CH_2_NO_2_ anion by the H16 atom in the guanidium cation [52]. Accordingly, G1-I-*re*-IM2 and G1-I-*si*-IM2 in model I were slightly more stable than those of G1-II-*re*-IM2 and G1-II-*si*-IM2 by 0.9~1.7 kcal·mol^−1^ (Figure 3). In the following step, the C=N bond addition was achieved by nucleophilic attack of the nitronate towards the C13 atom of R1a along four pathways (I-*re*, I-*si*, II-*re* or II-*si*), followed by easy proton transfer from the N2 atom to N14 atom. The hydrogen bonds between the guanidine cation and *N*-Boc ketimine (R1a) in four TSs were also proved by AIM analysis (Figure S2). The relative Gibbs free energy of transition states G1-I-*re*-TS2 and G1-I-*si*-TS2 were 1.1 and 2.3 kcal·mol^−1^, respectively, which were lower than those of G1-II-*re*-TS2 and G1-II-*si*-TS2 (2.4 and 4.0 kcal·mol^−1^, respectively; Figure 3 and Figure 4). In addition, the activation barrier in the C–C bond formation step along the I-*re* path (∆*G*^≠^ = 2.4 kcal·mol^−1^) was lower than that of the I-*si* path (∆*G^≠^* = 4.6 kcal·mol^−1^) by 2.2 kcal·mol^−1^. For the II-*re* pathway, ∆*G^≠^* in the C–C bond formation step was 0.4 kcal·mol^−1^ higher than that along I-*re*. Meanwhile, both G1-II-*re*-IM2 and G1-II-*re*-TS2 were less stable than the corresponding species in I-*re* pathway. These results indicated that the predominant *R*-configuration product observed in the experiment was predominantly produced along the I-*re* path. According to the Curtin–Hammett principle [53], the theoretical enantioselectivity (ee %) was 85%, which was close to the experimental result (91% ee).

We further analyzed the orbital interactions between *N*-Boc ketimine (R1a) and nitromethane in the C–C bond formation step. The structures of G1-I-*re*-TS2 and G1-I-*si*-TS2 were decomposed into a guanidine–nitromethane ion-pair fragment (G1-R2, Frag. 1) and an *N*-Boc ketimine fragment (R1a, Frag. 2). The schematic orbital interaction diagrams are shown in Figures S3 and S4. For the highest occupied molecular orbital (HOMO) of G1-I-*re*-TS2, it was mainly formed by the mixture of the occupied highest occupied fragment orbital (HOFO) of the G1-R2 fragment (Frag. 1, 73.35%) and the unoccupied lowest unoccupied fragment orbital (LUFO) of the R1a fragment (Frag. 2, 16.99%). The electronic density delocalization between BD(π) C15–N17 to the unoccupied BD(π*) C13–N14 (E(2) = 19.2 kcal·mol^−1^) promoted the formation of a C13–C15 bond, contributing to the orbital interaction energy (∆*E*_orb_) of −45.29 kcal·mol^−1^. Similar molecular orbital interactions was also observed in G1-I-*si*-TS2, with less contribution of the HOFO orbital of the G1-R2 fragment (63.59%) in the HOMO.

For comparison, we also studied the dual hydrogen bond model proposed by the experiment [44]. Four optimized geometries of transition states (G1-I-*re*-TS2-a ~ G1-II-*si*-TS2-a) along *re*- and *si*-pathways in two models were located at the same theriacal level (Figure S5). The ∆*G* of transition states in model I were significantly lower than those in model II by 7.2~10.2 kcal·mol^−1^. For the TSs in model I, the CH_2_NO_2_ anion was positioned by two NH groups in the guanidine moiety of G1 catalyst, with a N–H⋯O distance of 1.95~2.47 Å. It seemed that two Cy groups in the guanidine exerted comparable hindrance effects towards ketimine in the two competing transition states. Moreover, the ∆*G* of G1-I-*re*-TS2-a was 0.9 kcal·mol^−1^ less stable than that of G1-I-*si*-TS2-a, affording the *S*-product as a major one (not the *R*-product observed in experimental observation). Although the relative Gibbs free energy of G1-I-*re*-TS2-a in C–C bond formation steps were lower than that of G1-I-*re*-TS2 by 2.6 kcal·mol^−1^, the guanidine–amide catalyst had to undergo remarkable conformation changes from G1-IM1 to realize the pre-organized structure in TSs (as shown in Figure S6, the ∆*G^≠^* to change the orientation of two N–Cy groups in the guanidine cation were about 10~13 kcal·mol^−1^). Thus, unless specified, we just focus on the conformation of the guanidine cation with an intramolecular H4⋯O8 in the following discussions.

#### 2.2.3. Origin of Stereoselectivity

**Figure 5:**
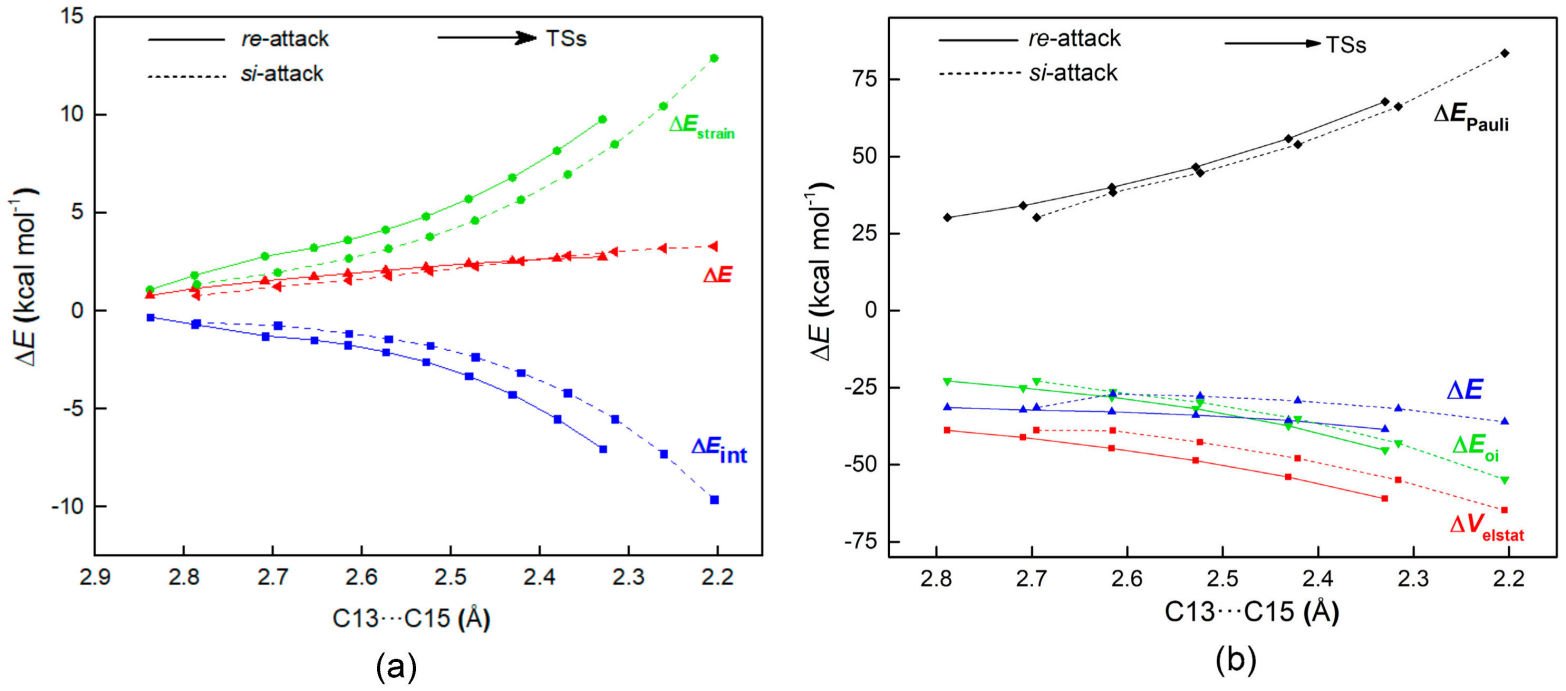
(**a**) ASM analysis of aza-Henry reaction between *N*-Boc ketimine (R1a) and nitromethane (R2) along the reaction coordinate projected onto the C13⋯C15 distance for *re*- and *si*-pathways. (**b**) Evolution of ∆*E*_int_ of two energy components along the reaction coordinate.

To understand the origin of stereoselectivity in the aza-Henry reaction between ketimine (R1a) and nitromethane (R2) catalyzed by guanidine G1, we analyzed the structures of the two key transition states (i.e., G1-I-*re*-TS2 and G1-I-*si*-TS2) in the chiral-controlling step (i.e., C–C bond formation step). As shown in Figure 4, the *N*-Boc group was placed at the same side as the bulky CPh_3_ group of G1 in the G1-I-*si*-TS2, with a distance of about 2.83 Å. The steric repulsion between them increased the Pauli repulsion energy ∆*E*_pauli_ (67.8 vs. 83.5 kcal·mol^−1^), as well as the strain energy ∆*E*_strain_ of the two reacting fragments (9.8 vs. 12.9 kcal·mol^−1^) at TSs along the I-*si* pathway (Figure 5). As a result, the activation energy barrier (∆*G*^≠^) along the I-*si* pathway was higher than those along the I-*re* pathway by 2.2 kcal·mol^−1^. In contrast, the unfavorable steric repulsion could be efficiently avoided in G1-I-*re*-TS2 because the CPh_3_ group was far away from the *N*-Boc group. The stronger interaction between the deformed reactants played the crucial factor in favoring the *re*-pathway [54,55]. That is, the more stabilizing electrostatic energy (∆*V*_elstat_) and orbital energy (∆*E*_orb_) efficiently offset the Pauli repulsion of two reacting fragments. Moreover, the Bn group with structural flexibility could weaken the steric hindrance from the CPh_3_ group, contributing to the low relative Gibbs free energy of transition state G1-I-*re*-TS2 (1.1 vs. 2.4 kcal·mol^−1^ for G1-I-*si*-TS2). The noncovalent interaction (NCI) plots in Figure S7, Supplementary Materials, also reveal a large green region in G1-I-*re*-TS2, associated with the stabilizing CH_2_⋯π interaction between the Bn group in ketimine and CPh_3_ group in the catalyst.

**Figure 6:**
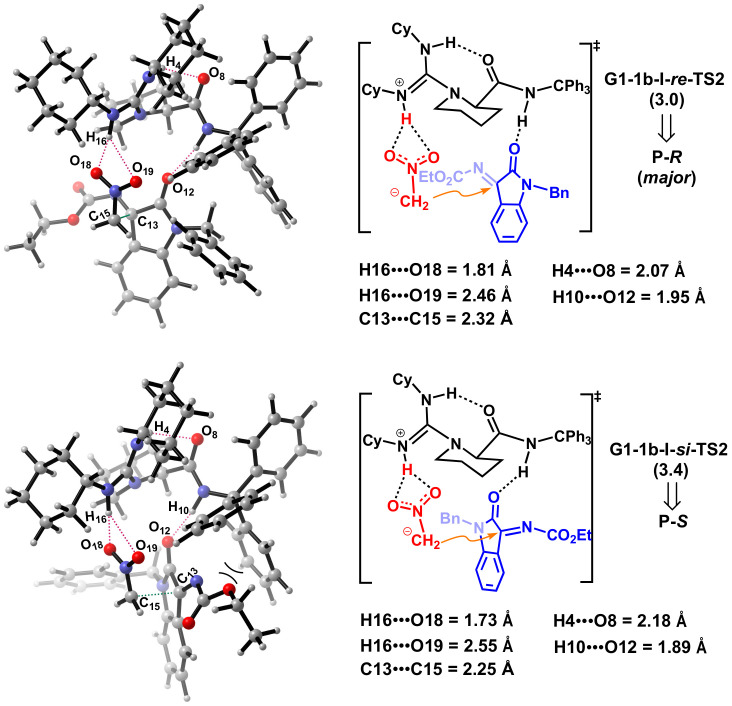
Optimized structures and the relative Gibbs free energy (kcal·mol^−1^) of transition states G1-1b-I-*re*-TS2 and G1-1b-I-*si*-TS2 in the C–C bond formation step in the aza-Henry reaction between ketimine (R1b) and nitromethane (R2) catalyzed by guanidine G1.

When the ketimine R1b with a small *N*-COOEt group was used as a reactant in the catalytic aza-Henry reaction, the repulsion between the *N*-protected group in R1b and CPh_3_ group in G1 was significantly decreased. Accordingly, the ∆∆*G* of the two competing transition states (G1-1b-I-*re* and G1-1b-I-*si* in Figure 6 and Figure S8) was decreased to 0.4 kcal·mol^−1^, leading to low stereoselectivity. The inferior enantioselectivity for R1b was also observed in the experiment [44]. In contrast to R1a, the charge accumulated on the O12 atom in R1b was slightly low (−0.580 e vs. −0.584 e in R1a). In addition, the H16⋯O12 distances in G1-1b-I-*re*-IM2 and G1-1b-I-*si*-IM2 were 1.976 and 1.986 Å, respectively, which were longer than those in the corresponding intermediates for R1a (1.950 and 1.917 Å). Due to weak catalyst–R1b interactions, more energy was required to deform R1b to the TS geometries. Accordingly, the Δ*E*^≠^_strain_ values of G1-1b-I-*re*-TS2 and G1-1b-I-*si*-TS2 (11.3 and 12.7 kcal·mol^−1^) were more destabilizing than that of G1-I-*re*-TS2 (9.8 kcal·mol^−1^), leading to higher activation barriers.

**Figure 7:**
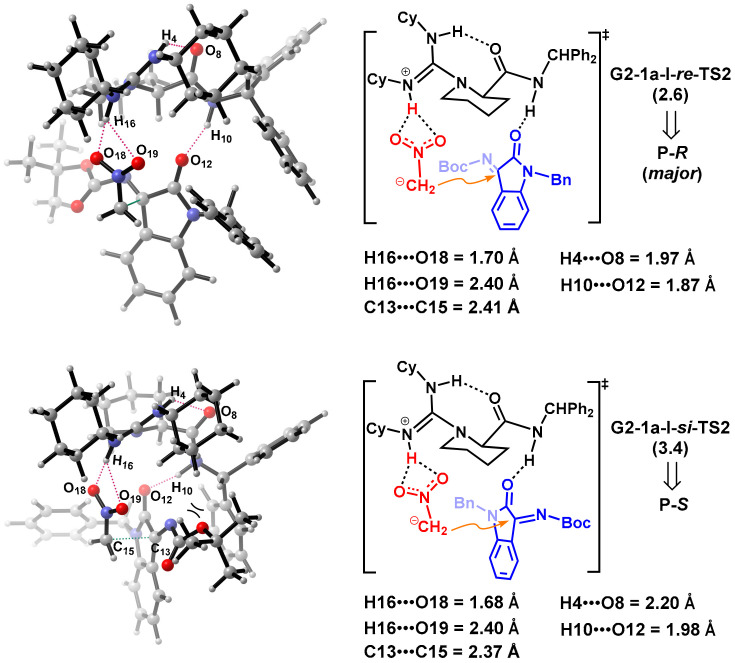
Optimized structures of transition states and the relative Gibbs free energies (kcal·mol^−1^) in the aza-Henry reaction between *N*-Boc ketimine (R1a) and nitromethane (R2) catalyzed by guanidine G2.

We then removed a phenyl in G1 to obtained guanidine–amide catalyst G2. The optimized geometries of the two key transition states (i.e., G2-1a-I-*re*-TS2 and G2-1a-I-*si*-TS2) in the C–C bond formation step were shown in Figure 7 and energy profile was in Figure S9, Supplementary Materials. Similar to G1, the *re*-face attack pathway was still more favorable than the *si*-face attack pathway in model I. However, the ∆∆*G* of the two competing transition states (G2-1a-I-*re*-TS2 and G2-1a-I-*si*-TS2) was decreased to 0.8 kcal·mol^−1^, associated with low selectivity (ee = 68%). Thus, the bulky CPh_3_ substituent was the key structural unit for asymmetric induction in the chiral guanidine–amide catalyst G1. The steric repulsion between *N*-Boc ketimine and CPh_3_ substituent in G1 increased the Pauli repulsion as well as the strain energy of the reacting fragments in the chiral-controlling step, contributing to the excellent stereochemical outcomes in the asymmetric aza-Henry reaction of *N*-Boc ketimine.

#### 2.2.4. Turnover Frequency (TOF) Analysis

To evaluate the efficiency of the catalyst G1 along four pathways in Figure 3, the theoretical turnover frequency (TOF) of the catalytic cycle was calculated [56]. As shown in Table 1, G1-1a-I-*re*-IM2 ~ G1-1a-II-*si*-IM2 were predicated to be TOF-determining intermediates, and G1-1a-I-*re*-TS2 ~ G1-1a-II-*si*-TS2 were the TOF-determining transition states. The TOF value along the *re*-face pathway in model I was 2.06 × 10^10^ s^−1^, which was higher than those of other three pathways. That is, G1 exhibited better catalytic efficiency when the Henry reaction of *N*-Boc ketimine R1a occurred along the I-*re*-pathway.

**Figure 8:**
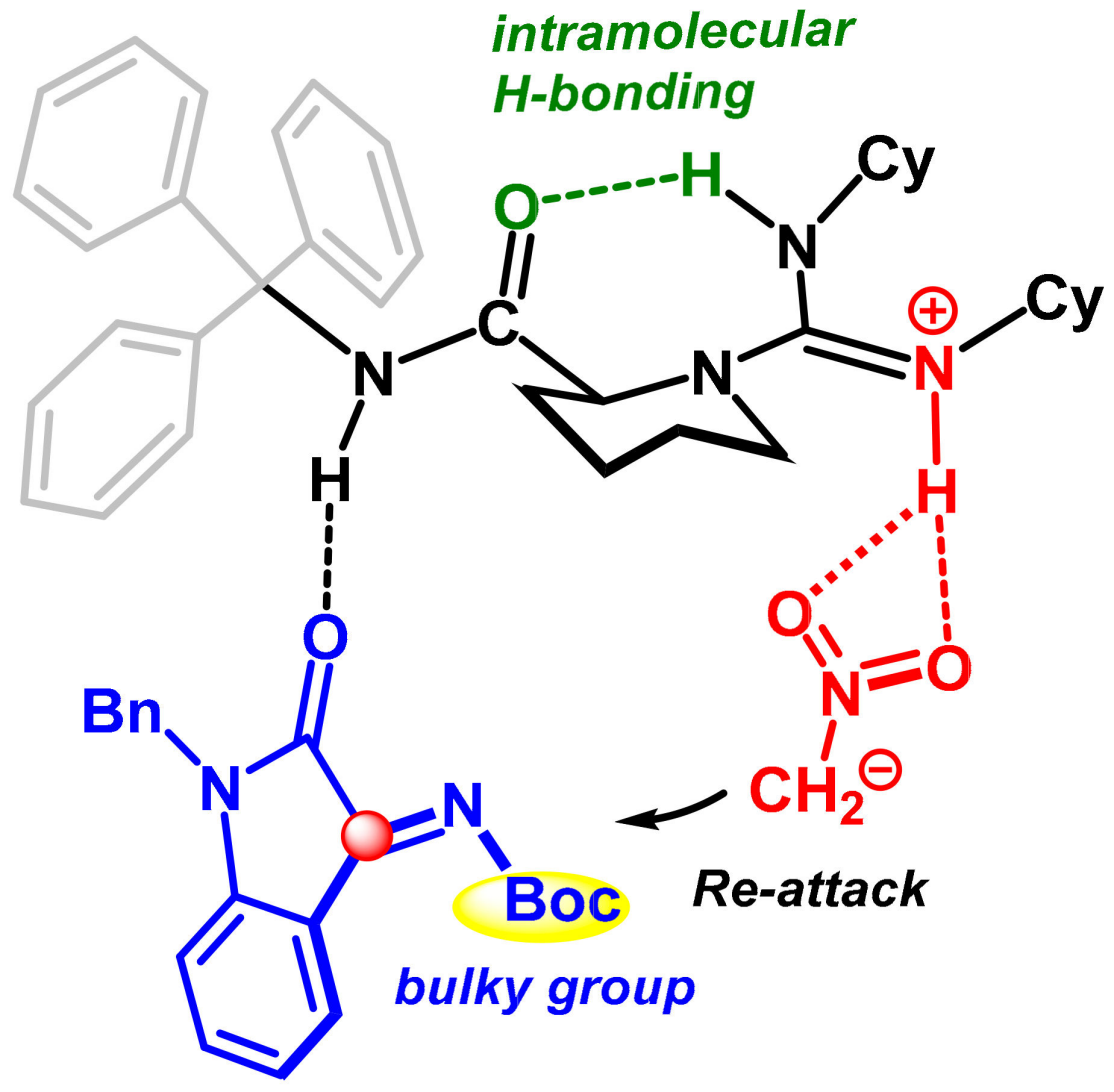
Origin of enantioselectivity in the aza-Henry reaction.

Based on the results above, a working model was proposed to explain the activation of the reactants as well as the chiral induction effect of the guanidine–amide catalyst in asymmetric aza-Henry reactions. As shown in Figure 8, three factors were important for the high level of stereoselective outcomes in the catalysis, including: (i) intramolecular hydrogen bonding between the amide skeleton and imine unit of guanidine enhanced the stability of catalyst, and fixed the position of the bulky CPh_3_ group in the chiral environment; (ii) *N*-Boc in the ketimine was placed away from the CPh_3_ group, avoiding the unfavorable steric repulsion from the CPh_3_ group; and (iii) the nitronate was oriented by hydrogen bonding from the imine moiety of guanidine for the favorable *re*-face attack for predominant *R*-configuration products.

### 2.3. Design of New Catalysts

**Scheme 4:**
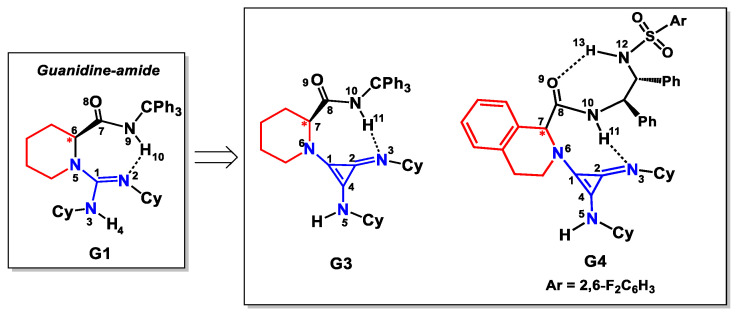
New catalysts G3 and G4.

**Figure 9:**
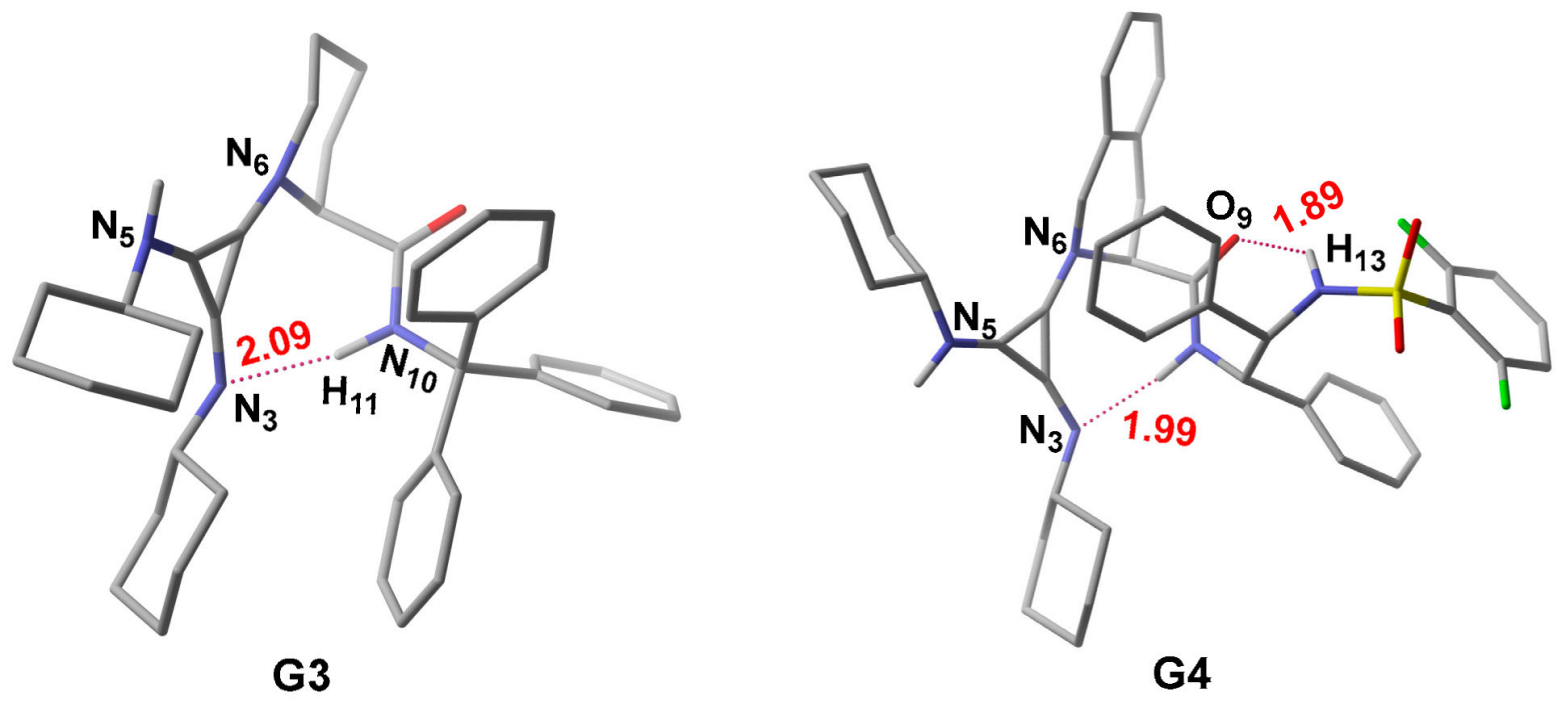
Optimized geometries of catalysts G3 and G4. The distances are in Å. The H atoms in phenyl and cyclohexyl groups are omitted for clarity.

In 2012, Lambert et al. developed a chiral 2,3-bis(dialkylamino) cyclopropenimine for the Michael reaction of a glycine imine substrate. The strong basicity of the cyclopropenimine moiety accelerated the deprotonation of glycine imine. In addition, the alcohol moiety of the catalyst activated methyl acrylate via H-bonding, contributing to the high enantioselectivity results [57]. Feng and Liu found that the modified guanidine–amide with 1,2-diphenylethylcarbamoyl and the 2,6-difluorobenzenesulfonamide group was efficient in the asymmetric catalysis [58]. Inspired by the excellent performance of cyclopropenimine in Brønsted base catalysis, we modified the structure of G1 by replacing the guanidine moiety with cyclopropenimine. In addition, the diamine with a 2,6-difluorob-enzenesulfonamide group was introduced into the molecular skeleton of the catalyst. Two new catalysts (G3 and G4) were designed (Scheme 4), and the corresponding optimized structures are shown in Figure 9. The mechanism of aza-Henry reaction between *N*-Boc ketimine (R1a) and CH_3_NO_2_ catalyzed by G3 and G4 in low-energy model I were studied at the same theoretical level to evaluate their catalytic performance as well as chiral induction effect.

**Figure 10:**
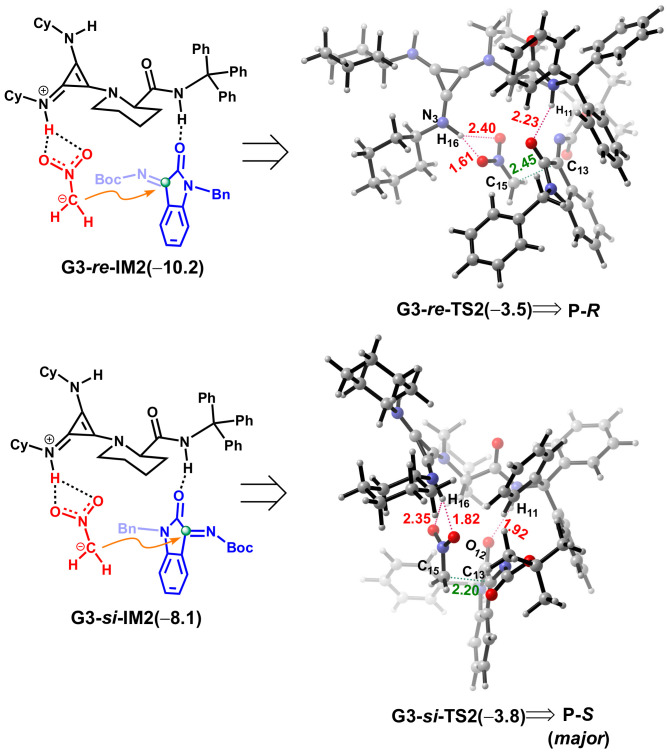
Optimized structures and the relative Gibbs free energies (in kcal·mol^−1^) of two competing transition states (G3-*re*-TS2 and G3-*si*-TS2) in the C–C bonding formation step of the aza-Henry reaction catalyzed by G3. The distances are in Å.

The calculations indicated that the reaction mechanisms in the presence of G3 or G4 were the same as that of G1. Due to the strong basicity of cyclopropenimine moiety, the energy barriers associated with the activation of CH_3_NO_2_ were decreased to 7.9 kcal·mol^−1^ for G3, and 7.2 kcal·mol^−1^ for G4. When protonated G3 interacted with *N*-Boc ketimine R1a, the molecular complexes G3-*re*-IM2 and G3-*si*-IM2 were formed, in which the amide moiety activated ketimine by hydrogen bonding. Compared to G3-*si*-IM2, the *N*-Boc in G3-*re*-IM2 was closer to the six-membered ring of the chiral skeleton as well as the CPh_3_ group, with a distance of about 2.51 Å and 2.46 Å, respectively. Suffering from the steric repulsion from the catalyst, the relative Gibbs free energy of transition state G3-*re*-TS2 in the C–C bond formation along the *re*-face attack pathway was slightly higher than via the transition state G3-*si*-TS2 by 0.3 kcal·mol^−1^ (Figure 10). Consequently, the product with *S*-configuration was formed with slight predominance.

**Figure 11:**
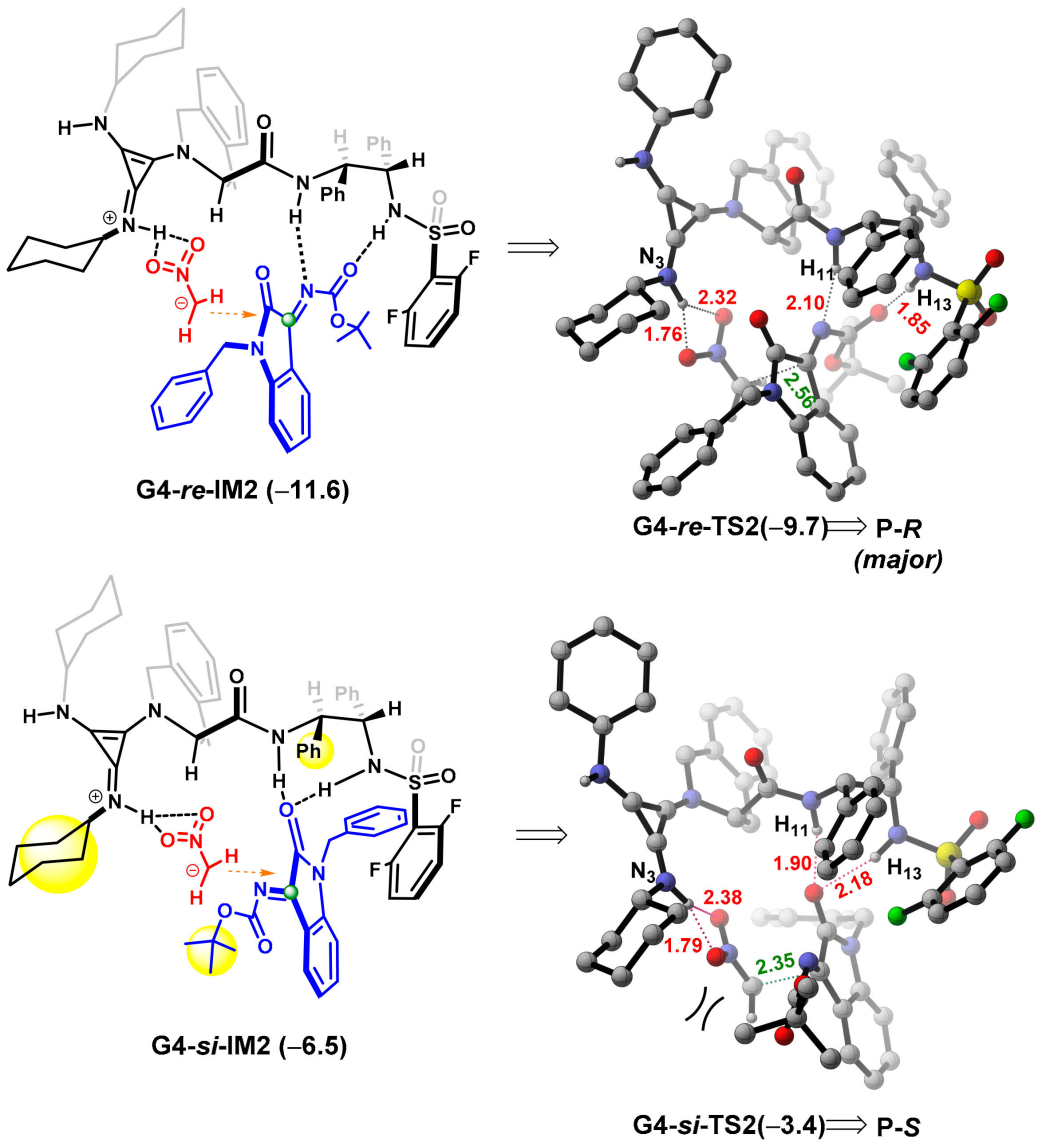
Optimized structures and the relative Gibbs free energies (in kcal·mol^−1^) of two competing transition states (G4-*re*-TS2 and G4-*si*-TS2) in the C–C bonding formation step of the aza-Henry reaction catalyzed by G4. The distances are in Å.

**Figure 12:**
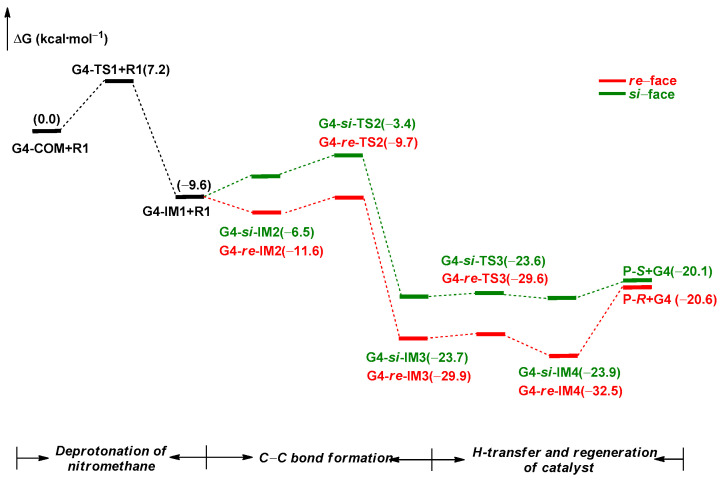
Energy profiles for aza-Henry reaction between *N*-Boc ketimine (R1a) and nitromethane (R2) catalyzed by G4 along *re*-face and *si*-face pathways, respectively.

When G4 was used as the catalyst, the O9⋯H13 and N3⋯H11 intramolecular hydrogen bonding weakened its structural flexibility (Figure 9). The molecular complexes G4-*re*-IM2 and G4-*si*-IM2 were formed, in which the cyclopropenimine activated the CH_2_NO_2_ anion, and N10-H11 as well as the N12-H13 moiety interacted with ketimine R1a by double hydrogen-bonding. In the following step, the C–C bond was constructed via transition state G4-*re*-TS2 or G4-*si*-TS2, alternatively (Figure 11 and Figure 12). Compared to G4-*re*-TS2, the *N*-Boc group of ketimine in G4-*si*-TS2 was closer to 2,6-difluorobenzenesulfonamide, Ph group in the chiral backbone, as well as Cy group in the guanidine moiety. This unfavorable steric hindrance made G4-*si*-TS2 less stable than G4-*re*-TS2 by 6.3 kcal·mol^−1^. The activation barrier from G4-*si*-IM2 to G4-*si*-IM3 along the *si*-pathway was 3.1 kcal·mol^−1^, which was higher than ΔG^≠^ from G4-*re*-IM2 to G4-*re*-IM3 along the *re*-pathway (1.9 kcal·mol^−1^), contributing to the predominant formation of P-*R* (inconsistent with G1). In addition, because G4-*re*-IM2 was 5.1 kcal·mol^−1^ lower than that of G4-*si*-IM2, the catalyst concentration could be higher in the G4-*re*-IM2 reaction system.

Therefore, the synergistic effects of the stronger Brønsted base (i.e., cyclopropenimine) and dual hydrogen bonding donor accelerated the activation of CH_3_NO_2_ and governed the facial selectivity in substrate binding, contributing to good reactivity and selectivity.

**Scheme 5:**
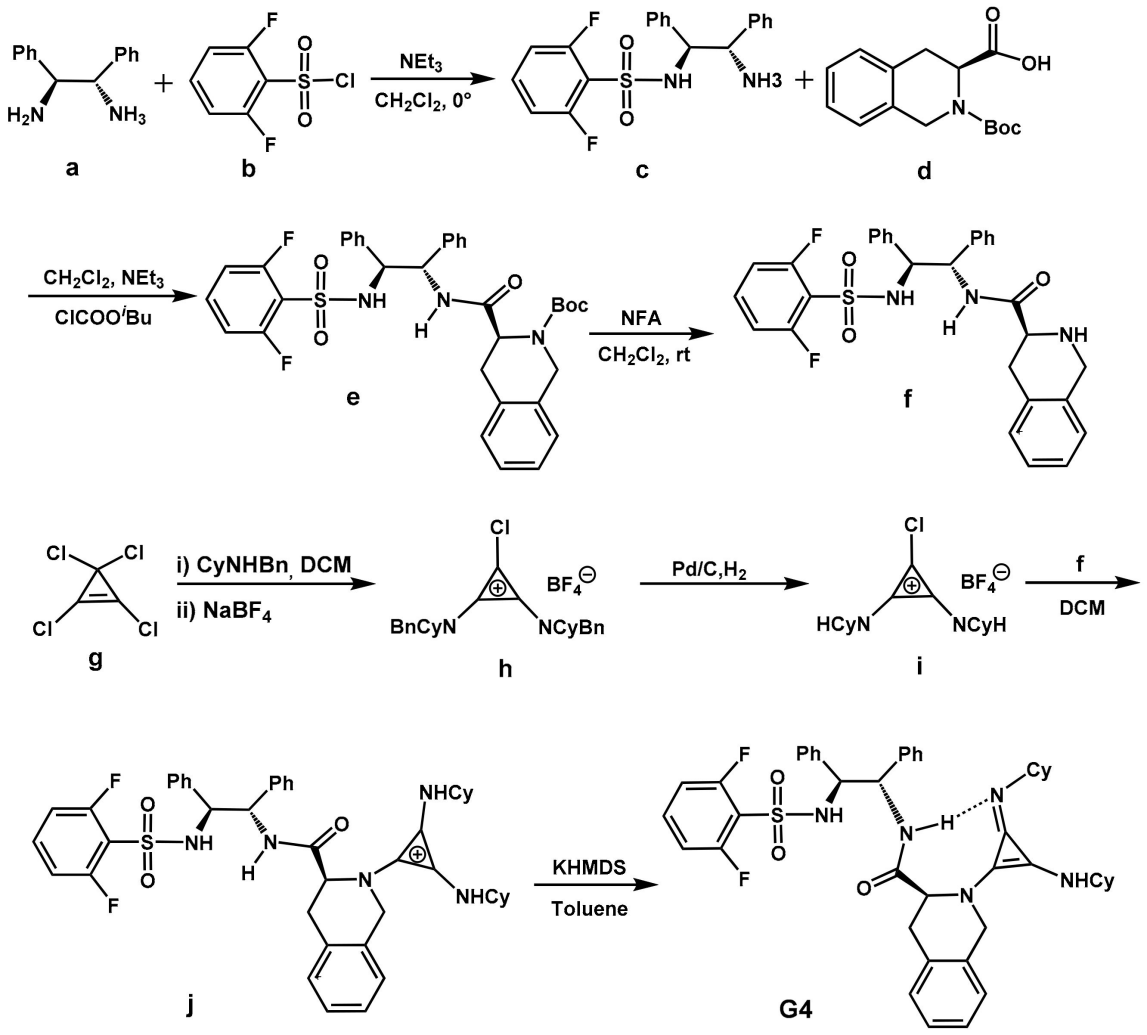
A possible synthesis route of catalyst G4.

According to synthesis investigation on guanidine derivatives with 1,2-diphenylethane-1,2-diamine groups by Feng and Liu [59], and guanidine–cyclopropenimine proton sponges by Dudding [58], we proposed a possible synthesis route for a new catalyst, G4 (Scheme 5). We expected that these results would be helpful for the development for the new catalytic system in the aza-Henry reaction of ketimine.

## 3. Materials and Methods

All DFT calculations were performed with the Gaussian 09 program [60]. The structures were optimized using the M06-2X [61] -D3 [62] function with 6-31G(d,p) basis set, and characterized by the vibrational frequency analysis at 243 K. The SMD solvation model [63] for toluene was employed in the structure optimization. The intrinsic reaction coordinate (IRC) [64] paths were traced to check the energy profiles connecting each transition state to the associated minima of the proposed mechanism. Single point energy was obtained using the same functional and 6-311+G(d,p) basis set. Natural bond orbital (NBO) [65] and reactivity indices (electrophilicity index *ω* and nucleophilicity index *N*) analysis for the reactants and the key intermediates were performed at the M06-2X-D3/6-311+G(d,p)(SMD, toluene) theoretical level. The gas-phase proton affinity (PA) [66], defined as the enthalpy change (Δ*H*) of the deprotonation reaction (Equation (1)), was used to evaluate the reactivity of the nitrogen atoms in guanidine (G):

GH^+^ (g) → G (g) + H^+^ (g)   Δ*H* = PA (1)

To gain insights into the factors affecting activation barriers in the reaction process, we performed activation strain model (ASM) [67] (or distortion/interaction model [68]) analysis. Accordingly, the energy barrier (∆*E*) was decomposed into distortion energy (∆*E*_strain_) and interaction energy (∆*E*_int_) (Equation (2)):

∆*E* = ∆*E*_strain_ + ∆*E*_int_ (2)

In addition, the interaction energy (∆*E*_int_) between the strained reactants was further decomposed into electrostatic interactions (∆*V*_elstat_), orbital interactions (∆*E*_oi_), and Pauli repulsions (∆*E*_Pauli_) by energy decomposition analysis (EDA) (Equation (3)) [69]. The EDA and the fragmental orbital interaction analysis were performed with the Amsterdam density functional (ADF) program package [70] at the M06-2X/TZ2P theoretical level.

∆*E*_int_ = ∆*V*_elstat_ + ∆*E*_Pauli_ + ∆*E*_oi_ (3)

Atom in molecule (AIM) analysis was carried out using Multiwfn software [71] to reveal the inter- and intramolecular interactions between catalyst and reactant. A positive or negative Laplacian value of the electron density (*ρ*) at the (3, −1) bond critical point (BCP) indicates that the electron density is divergent or aggregated, respectively [72,73]. Various possible conformations of the protonated guanidine species were generated by Material studio software and optimized with Gaussian 09 program. Two low-energy conformers were studied in the catalytic reaction.

## 4. Conclusions

The DFT method was adopted to study the reaction mechanism and the origin of enantioselectivity of the aza-Henry reaction between isatin-derived *N*-Boc ketimine and nitromethane catalyzed by the guanidine–amide catalyst. The calculations showed that the catalytic reaction occurred via a three-step mechanism. The deprotonation of nitromethane was the rate-determining step (RDS), while the C–C bond formation was the chiral-controlling step. The intramolecular hydrogen bonding formed between the amide skeleton and imine unit of guanidine enhanced the rigidity of catalyst, facilitating the imine and amide moiety to simultaneously interact with CH_3_NO_2_ and *N*-Boc ketimine by hydrogen bonding. The repulsion between the CPh_3_ group in guanidine and the *N*-Boc group in ketimine played an important role in controlling the enantioselectivity of the product. An unfavorable steric arrangement at the *si*-face attack pathway enhancing the Pauli repulsion energy as well as strain energy of the reacting species, leading to a predominant *R*-configuration product. The new catalysts with cyclopropenimine and sulfonamide unit were designed. The strong basicity of the cyclopropenimine moiety accelerated the activation of CH_3_NO_2_ by decreasing the energy barrier in the deprotonation step. The hydrogen bonding from diamine oriented ketimine well. The repulsion between the *N*-Boc group in ketimine and 2,6-difluoro-benzenesulfonamide and Cy groups in the catalyst raised the energy of the C–C bond formation transition state along the *si*-face attack pathway, leading to the formation of a predominant *R*-configuration product.

## Figures and Tables

**Table 1:** Turnover frequency (TOF) of the catalytic cycle of aza-Henry reactions of isatin-derived ketimine (R1a) and nitromethane (R2) along four pathways. TDI and TDTS are the TOF-determining intermediates and TOF-determining transition states, respectively.

Path	TDI	TDTS	TOF (s^−1^)	Product
I-*re*	G1-1a-I-*re*-IM2	G1-1a-I-*re*-TS2	2.06 × 10^10^	P-*R* (major)
I-*si*	G1-1a-I-*si*-IM2	G1-1a-I-*si*-TS2	2.98 × 10^8^	P-*S*
II-*re*	G1-1a-II-*re*-IM2	G1-1a-II-*re*-TS2	1.24 × 10^10^	P-*R*
II-*si*	G1-1a-II-*si*-IM2	G1-1a-II-*si*-TS2	1.03 × 10^10^	P-*S*

## Data Availability

Data are contained within the article or Supplementary Materials.

## Supplementary Materials

The following are available online. Table S1: Comparison of key geometric parameters obtained by experiments and theoretical calculations at the M06-2X-D3/6-31G(d,p) (SMD, toluene) level; Figure S1: Contour line maps of Laplace field for four intermediates. The blue points represent BCPs, and the solid and dashed lines represent the areas with positive and negative Laplacian of electron density, respectively; Figure S2: Selected bond critical points with corresponding Laplacian (∇^2^*ρ*) and electronic density (*ρ*) values for four transition states G1-I-*re*-TS2 ~ G1-II-*si*-TS2 by AIM analysis; Figure S3: Schematic molecular orbital interaction diagram for transition state G1-I-*re*-TS2, constructed by G1-R2 (Frag. 1) and *N*-Boc ketimine (Frag. 2) fragments obtained with the ADF program; Figure S4: Schematic molecular orbital interaction diagram for transition state G1-I-*si*-TS2, constructed by G1-R2 (Frag.1) and *N*-Boc ketimine (Frag. 2) fragments obtained with the ADF program; Figure S5: Optimized geometries of transition states G1-I-*re*-TS2-a ~ G1-II-*si*-TS2-a. Relative Gibbs free energies are given in kcal·mol^−1^; Figure S6: Relaxed potential energy scan of guanidine cation, calculated by scanning the dihedral angle N5-C1-N2-Cy (a) and N5-C1-N3-Cy (b) at M06-2X-D3/6-31G(d,p)(SMD, toluene) theoretical level; Figure S7: Noncovalent interaction (NCI) plots near bulky CPh_3_ group areas for G1-I-*re*-TS2 ~ G1-II-*si*-TS2. Strong and attractive interactions are blue, weak interactions are green, and strong and repulsive interactions are red; Figure S8: Energy profiles for aza-Henry reaction between *N*-COOEt ketimine (R1b) and nitromethane (R2) catalyzed by guanidine (G1) along *re*-face and *si*-face pathways in model I; Figure S9: Energy profiles for aza-Henry reaction between *N*-Boc ketimine (R1a) and nitromethane (R2) catalyzed by guanidine (G2) along *re*-face and *si*-face pathways in model I.

## Sample Availability

Samples of the compounds are not available from the authors.

## References

[1] Marques-Lopez E., Merino P., Tejero T., Herrera R.P. (2009). Catalytic Enantioselective Aza-Henry Reactions. Eur. J. Org. Chem..

[2] Nef J.U. (1894). Ueber die Constitution der Salze der Nitroparaffine. Justus Liebigs Ann. Chem..

[3] Ballini R., Petrini M. (2004). Recent Synthetic Developments in the Nitro to Carbonyl Conversion (Nef Reaction). Tetrahedron.

[4] Krawczyk H., Albrecht L., Wojciechowski J., Wolf W.M. (2006). Spontaneous Nef Reaction of 3-Aryl-2-(Diethoxyphosphoryl)-4-Nitroalkanoic Acids. Tetrahedron.

[5] Bhat C., Tilve S.G. (2013). Henry-Nef Reaction: A Practical and Versatile Chiral Pool Route to 2-Substituted Pyrrolidine and Piperidine Alkaloids. Tetrahedron.

[6] Ju M., Guan W.Y., Schomaker J.M., Harper K.C. (2019). Sequential Reduction of Nitroalkanes Mediated by CS_2_ and Amidine/Guanidine Bases: A Controllable Nef Reaction. Org. Lett..

[7] Lloyd D.H., Nichols D.E. (1986). Nickel Boride/Hydrazine Hydrate: Reduction of Aromatic and Aliphatic Nitro Compounds. Synthesis of 4-(Benzyloxy)Indole and α-Alkyltryptamines. J. Org. Chem..

[8] Lucet D., Gall T.L., Mioskowski C. (1998). The Chemistry of Vicinal Diamines. Angew. Chem. Int. Ed..

[9] Kotti S.R.S.S., Timmons C., Li G.G. (2006). Vicinal Diamino Functionalities as Privileged Structural Elements in Biologically Active Compounds and Exploitation of Their Synthetic Chemistry. Chem. Biol. Drug. Des..

[10] Kizirian J.C. (2008). Chiral Tertiary Diamines in Asymmetric Synthesis. Chem. Rev..

[11] Yamada K., Moll G., Shibasaki M. (2001). The First Enantioselective and Diastereoselective Catalytic Nitro-Mannich Reaction: A New Entry to Chiral Vicinal Diamines. Synlett.

[12] Palomo C., Oiarbide M., Halder R., Laso A., Lópze R. (2006). Enantioselective Aza-Henry Reactions Assisted by Zn(II) and *N*-Methylephedrine. Angew. Chem. Int. Ed..

[13] Dudek A., Mlynarski J. (2017). Iron-Catalyzed Asymmetric Nitro-Mannich Reaction. J. Org. Chem..

[14] Yamada K., Harwood S.J., Gröger H., Shibasaki M. (1999). The First Catalytic Asymmetric Nitro Mannich-Type Reaction Promoted by a New Heterobimetallic Complex. Angew. Chem. Int. Ed..

[15] Zhang S., Li Y.N., Xu Y.G., Wang Z.Y. (2018). Recent Progress in Copper Catalyzed Asymmetric Henry Reaction. Chin. Chem. Lett..

[16] Okino T., Nakamura S., Furukawa T., Takemoto Y. (2004). Enantioselective Aza-Henry Reaction Catalyzed by a Bifunctional Organocatalyst. Org. Lett..

[17] Ting A., Schaus S.E. (2007). Organocatalytic Asymmetric Mannich Reactions: New Methodology, Catalyst Design, and Synthetic Applications. Eur. J. Org. Chem..

[18] Belding L., Taimoory S.M., Dudding T. (2015). Mirroring Enzymes: The Role of Hydrogen Bonding in an Asymmetric Organocatalyzed Aza-Henry Reaction-a DFT Study. ACS Catal..

[19] Notz W., Tanaka F., Watanabe S.-i., Chowdari N.S., Turner J.M., Thayumanavan R., Barbas C.F. (2003). The Direct Organocatalytic Asymmetric Mannich Reaction: Unmodified Aldehydes as Nucleophiles. J. Org. Chem..

[20] Ochi M., Kawasaki K., Kataoka H., Uchio Y., Nishi H. (2001). AG-041R, a Gastrin/CCK-B Antagonist, Stimulates Chondrocyte Proliferation and Metabolism in Vitro. Biochem. Biophys. Res. Commun..

[21] Lin H., Danishefsky S.J. (2003). Gelsemine: A Thought-Provoking Target for Total Synthesis. Angew. Chem. Int. Ed..

[22] Oost T., Backfisch G., Bhowmik S., van Gaalen M.M., Geneste H., Hornberger W., Lubisch W., Netz A., Unger L., Wernet W. (2011). Potent and Selective Oxindole-based Vasopressin 1b Receptor Antagonists with Improved Pharmacokinetic Properties. Bioorg. Med. Chem. Lett..

[23] Berlinck R.G.S., Romminger S. (2016). The Chemistry and Biology of Guanidine Natural Products. Nat. Prod. Rep..

[24] Arai T., Matsumura E., Masu H. (2014). Bis(imidazolidine)pyridine-NiCl_2_ Catalyst for Nitro-Mannich Reaction of Isatin-Derived *N*-Boc Ketimines: Asymmetric Synthesis of Chiral 3-Substituted 3-Amino-2-oxindoles. Org. Lett..

[25] Holmquist M., Blay G., Pedro J.R. (2014). Highly Enantioselective Aza-Henry Reaction with Isatin *N*-Boc Ketimines. Chem. Commun..

[26] Tan C., Liu X.H., Wang L.W., Wang J., Feng X.M. (2008). Highly Enantioselective Aza-Henry Reaction of Ketoimines Catalyzed by Chiral *N, N′*-Dioxide-Copper(I) Complexes. Org. Lett..

[27] Bernardi L., Fini F., Herrera R.P., Ricci A., Sgarzani V. (2006). Enantioselective Aza-Henry Reaction Using Cinchona Organocatalysts. Tetrahedron.

[28] Bode C.M., Ting A., Schaus S.E. (2006). A General Organic Catalyst for Asymmetric Addition of Stabilized Nucleophiles to Acyl Imines. Tetrahedron.

[29] Kumar A., Kuar J., Chimni S.S., Jassal A.K. (2014). Organocatalytic Enantioselective Aza-Henry Reaction of Ketimines Derived from Isatins: Access to Optically Active 3-Amino-2-Oxindoles. RSC Adv..

[30] Li P., Sun D.W., Jiang M., Liu J.T. (2019). Asymmetric Aza-Henry Reaction of Fluoromethylated Imines Catalyzed by Cinchona-Derived Bifunctional Thiourea. Tetrahedron.

[31] Rampalakos C., Wulff W.D. (2008). A Novel Bis-Thiourea Organocatalyst for the Asymmetric Aza-Henry Reaction. Adv. Synth. Catal..

[32] Hajra S., Jana B. (2017). Quinine-Based Trifunctional Organocatalyst for Tandem Aza-Henry Reaction-Cyclization: Asymmetric Synthesis of Spiroxindole-Pyrrolidine/Piperidines. Org. Lett..

[33] Nunez M.G., Farley A.J., Dixon D.J. (2013). Bifunctional Iminophosphorane Organocatalysts for Enantioselective Synthesis: Application to the Ketimine Nitro-Mannich Reaction. J. Am. Chem. Soc..

[34] Xue Y.S., Wang Y.H., Cao Z.Y., Zhou J., Chen Z.X. (2016). Computational Insight into the Cooperative Role of Non-covalent Interactions in the Aza-Henry Reaction Catalyzed by Quinine Derivatives: Mechanism and Enantioselectivity. Org. Biomol. Chem..

[35] Wang J.D., Liu Y., Liu Y.X., Wei Z.L., Cao J.G., Liang D.P., Lin Y.J., Duan H.F. (2019). L-tert-Leucine Derived Urea-Ammonium Salts: Efficient Bifunctional Phase Transfer Catalysts for Highly Diastereo- and Enantioselective Aza-Henry Reaction of Isatin-derived *N*-Boc Ketimines with α-Aryl Nitromethanes. Tetrahedron.

[36] Sohtome Y., Nagasawa K. (2012). Dynamic Asymmetric Organocatalysis: Cooperative Effects of Weak Interactions and Conformational Flexibility in Asymmetric Organocatalysts. Chem. Commun..

[37] Fu X., Loh W.T., Zhang Y., Chen T., Ma T., Liu H., Wang J., Tan C.H. (2009). Chiral Guanidinium Salt Catalyzed Enantioselective Phospha-Mannich Reactions. Angew. Chem. Int. Ed..

[38] Corey E.J., Grogan M.J. (1999). Enantioselective Synthesis of α-Amino Nitriles from *N*-Benzhydryl Imines and HCN with a Chiral Bicyclic Guanidine as Catalyst. Org. Lett..

[39] Cho B., Wong M.W. (2015). Unconventional Bifunctional Lewis-Brønsted Acid Activation Mode in Bicyclic Guanidine-Catalyzed Conjugate Addition Reactions. Molecules.

[40] Xue H.S., Jiang D.F., Jiang H., Kee C.W., Hirao H., Nishimura T., Wong M.W., Tan C.H. (2015). Mechanistic Insights into Bicyclic Guanidine-Catalyzed Reactions from Microscopic and Macroscopic Perspectives. J. Org. Chem..

[41] Li J., Jiang W.Y., Han K.L., He G.Z., Li C. (2003). Density Functional Study on the Mechanism of Bicyclic Guanidine-Catalyzed Strecker Reaction. J. Org. Chem..

[42] Kee C.W., Peh K.Q.E., Wong M.W. (2017). Coupling Reactions of Alkynyl Indoles and CO_2_ by Bicyclic Guanidine: Origin of Catalytic Activity?. Chem. Asian J..

[43] Cho B., Tan C.H., Wong M.W. (2011). Sequential Catalytic Role of Bifunctional Bicyclic Guanidine in Asymmetric Phospha-Michael Reaction. Org. Biomol. Chem..

[44] Fang B., Liu X.H., Zhao J.N., Tang Y., Lin L.L., Feng X.M. (2015). Chiral Bifunctional Guanidine-catalyzed Enantioselective Aza-Henry Reaction of Isatin-derived Ketimines. J. Org. Chem..

[45] Cao W.D., Liu X.H., Feng X.M. (2018). Chiral Organobases: Properties and Applications in Asymmetric Catalysis. Chin. Chem. Lett..

[46] Dong S.X., Feng X.M., Liu X.H. (2018). Chiral Guanidines and Their Derivatives in Asymmetric Synthesis. Chem. Soc. Rev..

[47] Chou H., Leow D., Tan C.H. (2019). Recent Advances in Chiral Guanidine-Catalyzed Enantioselective Reactions. Chem. Asian J..

[48] Liu X.H., Lin L.L., Feng X.M. (2009). Amide-based Bifunctional Organocatalysts in Asymmetric Reactions. Chem. Commun..

[49] Li J., Su Z.S., Wang J.M., Hu C.W. (2018). Mechanistic Investigations on Asymmetric N-H Insertion of Amines Catalyzed by Palladium-chiral Guanidine Complex. J. Catal..

[50] Li J., Meng X.X., Hu C.W., Su Z.S. (2019). Cooperative Catalysis of Chiral Guanidine and Rh_2_(OAc)_4_ in Asymmetric O-H Insertion of Carboxylic Acid: A Theoretical Investigation. J. Org. Chem..

[51] Emamian S., Lv T., Kruse H., Emamian H. (2019). Exploring Nature and Predicting Strength of Hydrogen Bonds: A Correlation Analysis between Atoms-in-Molecules Descriptors, Binding Energies, and Energy Components of Symmetry-Adapted Perturbation Theory. J. Comput. Chem..

[52] Manzetti S., Lv T. (2013). The Geometry and Electronic Structure of Aristolochic Acid: Possible Implications for a Frozen Resonance. J. Phys. Org. Chem..

[53] Schneebeli S.T., Hall M.L., Breslow R., Friesner R. (2009). Quantitative DFT Modeling of the Enantiomeric Excess for Dioxirane-catalyzed Epoxidations. J. Am. Chem. Soc..

[54] Fernández I., Bickelhaupt F.M. (2014). The Activation Strain Model and Molecular Orbital Theory: Understanding and Designing Chemical Reactions. Chem. Soc. Rev..

[55] Bickelhaupt F.M., Houk K.N. (2017). Analyzing Reaction Rates with the Distortion/Interaction-Activation Strain Model. Angew. Chem. Int. Ed..

[56] Kozuch S., Shaik S. (2006). A Combined Kinetic-Quantum Mechanical Model for Assessment of Catalytic Cycles: Application to Cross-Coupling and Heck Reactions. J. Am. Chem. Soc..

[57] Bandar J.S., Lambert T.H. (2012). Enantioselective Brønsted Base Catalysis with Chiral Cyclopropenimines. J. Am. Chem. Soc..

[58] Guest M., Sueur R.L., Pilkington M., Dudding T. (2020). Development of an Unsymmetrical Cyclopropenimine-Guanidine Platform for Accessing Strongly Basic Proton Sponges and Boron-Difluoride Diaminonaphthalene Fluorophores. Chem. Eur. J..

[59] Ruan S., Lin X.B., Xie L.H., Lin L.L., Feng X.M., Liu X.H. (2018). Asymmetric Synthesis of 3-Aminodihydrocoumarins via the Chiral Guanidine Catalyzed Cascade Reaction of Azlactones. Org. Chem. Front..

[60] Frisch M.J., Trucks G.W., Schlegel H.B., Scuseria G.E., Robb M.A., Cheeseman J.R., Scalmani G., Barone V., Mennucci B., Petersson G.A. (2013). Gaussian 09, Revision D.01.

[61] Zhao Y., Truhlar D.G. (2008). The M06 Suite of Density Functionals for Main Group Thermochemistry, Thermochemical Kinetics, Noncovalent Interactions, Excited States, and Transition Elements: Two New Functionals and Systematic Testing of Four M06-Class Functionals and 12 Other Functionals. Theor. Chem. Acc..

[62] Grimme S., Antony J., Ehrlich S., Krieg H. (2010). A Consistent and Accurate Ab Initio Parametrization of Density Functional Dispersion Correction (DFT-D) for the 94 Elements H-Pu. J. Chem. Phys..

[63] Marenich A.V., Cramer C.J., Truhlar D.G. (2009). Universal Solvation Model Based on Solute Eectron Density and on a Continuum Model of the Solvent Defined by the Bulk Dielectric Constant and Atomic Surface Tensions. J. Phys. Chem. B.

[64] Gonzalez C., Schlegel H.B. (1989). An Improved Algorithm for Reaction-path Following. J. Chem. Phys..

[65] Reed A.E., Curtiss L.A., Weinhold F. (1988). Intermolecular Interactions from a Natural Bond Orbital, Donor-acceptor Viewpoint. Chem. Rev..

[66] Carlosa L.R., Loroa H., Lagob A.F., Dávalos J.Z. (2018). Gas-phase Proton Afnity and Basicity of Hydroxybenzophenones. Chem. Phys. Lett..

[67] Wolters L.P., Bickelhaupt F.M. (2015). The Activation Strain Model and Molecular Orbital Theory. WIREs Comput. Mol. Sci..

[68] Ess D.H., Houk K.N. (2007). Distortion/Interaction Energy Control of 1,3-Dipolar Cycloaddition Reactivity. J. Am. Chem. Soc..

[69] Hopffgarten M.V., Frenking G. (2012). Energy Decomposition Analysis. WIREs Comput. Mol. Sci..

[70] Te Velde G., Bickelhaupt F.M., Baerends E.J., Guerra C.F., Gisbergen S.J.A.V., Snijders J.G., Ziegler T. (2001). Chemistry with ADF. J. Comput. Chem..

[71] Lu T., Chen F. (2012). Multiwfn: A Multifunctional Wavefunction Analyzer. J. Comput. Chem..

[72] Bader R.F.W., Beddall P.M. (1972). Virial Field Relationship for Molecular Charge Distributions and the Spatial Partitioning of Molecular Properties. J. Chem. Phys..

[73] Bianchi R., Gervasio G., Marabello D. (2000). Experimental Electron Density Analysis of Mn_2_(CO)_10_: Metal-Metal and Metal-Ligand Bond Characterization. Inorg. Chem..

