# Supplementary material for: Guanidine–Amide-Catalyzed Aza-Henry Reaction of Isatin-Derived Ketimines: Origin of Selectivity and New Catalyst Design
Source: Molecules. 2021 Mar 31;26(7):1965. doi: 10.3390/molecules26071965 (PMC8037019; doi:10.3390/molecules26071965)
Supplement: Supplementary file 1 [file molecules-26-01965-s001.pdf]

Supporting Information

Guanidine-Amide-Catalyzed Aza-Henry Reaction of Isatin-Derived Ketimines: Origin of Selectivity and New Catalyst Design

Jiajia He,[1] Dianyong Tang,[2] Changwei Hu,[1] and Zhishan Su[1]*

[1] Key Laboratory of Green Chemistry and Technology, Ministry of Education, College of Chemistry, Sichuan University, Chengdu, Sichuan, 610064, P.R. China

[2] College of Pharmacy & International Academy of Targeted Therapeutics and Innovation, Chongqing University of Arts and Sciences, Chongqing 402160, China.

*Email: suzhishan@scu.edu.cn

Contents

Figure S1. Optimized geometry of guanidine G1 obtained at the M06-2X-D3/6-31G(d,p) (SMD, toluene) theoretical level (left), the proton affinity (PA) and charge at nitrogen atoms (right). The bond distance was in angstrom (Å), and H atoms in phenyl and cyclohexyl groups were omitted for clarity.. ....S1

Table S1. Comparison of key geometric parameters obtained by experiments and theoretical calculations at the M06-2X-D3/6-31G(d,p) (SMD, toluene) level. S1

Figure S2. Schematic molecular orbital interaction diagram for transition state G1-I-*re*-TS2, constructed by G1-R2 (Frag.1) and *N*-Boc ketimine (Frag.2) fragments obtained by ADF program. S2

Figure S3. Schematic molecular orbital interaction diagram for transition state G1-I-*si*-TS2, constructed by G1-R2 (Frag.1) and *N*-Boc ketimine (Frag.2) fragments obtained by ADF program. S2

Figure S4. Optimized geometries of transition states G1-I-*re*-TS2-a ~ G1-II-*si*-TS2-a. Relative Gibbs free energies were given in kcal mol^{-1}... ....S3

Figure S5. Relaxed potential energy scan of guanidine cation, calculated by scanning the dihedral angle N5-C1-N2-Cy (a) and N5-C1-N3-Cy (b) at M06-2X-D3/6-31G(d,p)(SMD, toluene) theoretical level.... ....S4

Figure S6. Energy profiles for aza-Henry reaction between *N*-COOEt ketimine (R1b) and nitromethane (R2) catalyzed by guanidine (G1) along *re*-face and *si*-face pathways in model I. S5

Figure S7. Energy profiles for aza-Henry reaction between *N*-Boc ketimine (R1a) and nitromethane (R2) catalyzed by guanidine (G2) along *re*-face and *si*-face pathways in model I... ....S5

Cartesian coordinates of all stationary points...S6

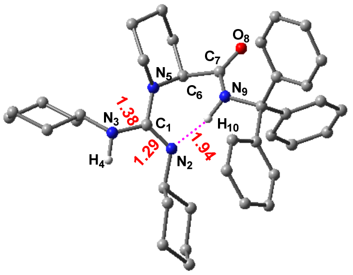

Atom	PA (kcal mol^{-1})	Charge
N2	226.4	-0.658
N3	196.3	-0.697
N5	199.6	-0.581

Figure S1. Optimized geometry of guanidine G1 obtained at the M06-2X-D3/6-31G(d,p) (SMD, toluene) theoretical level (left), the proton affinity (PA) and charge at nitrogen atoms (right). The bond distance was in angstrom (Å), and H atoms in phenyl and cyclohexyl groups were omitted for clarity.

Table S1. Comparison of key geometric parameters for guanidine-amide **G1** obtained by experiments and theoretical calculations at the M06-2X-D3/6-31G(d,p) (SMD, toluene) level.

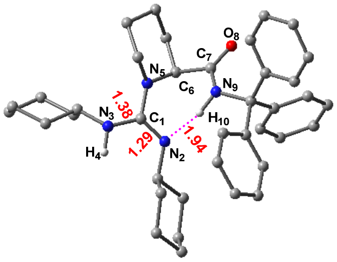

	Parameters	Exp.	Calc.	|Δ|
**Bonds (Å)**	N2–H10	2.25	1.94	0.31
	C1–N2	1.28	1.29	0.01
	C1–N3	1.40	1.38	0.02
	C7–N9	1.35	1.36	0.01
**Angles (°)**	N2-C1-N5	119.7	119.5	0.2
	N5-C6-C7	114.5	114.8	0.4
	C6-C7-N9	114.0	114.4	0.4
**Dihedral Angles (°)**	N5-C1-N2-Cy	-165.7	-172.2	6.5
	N5-C6-C7-N9	44.1	34.9	9.2

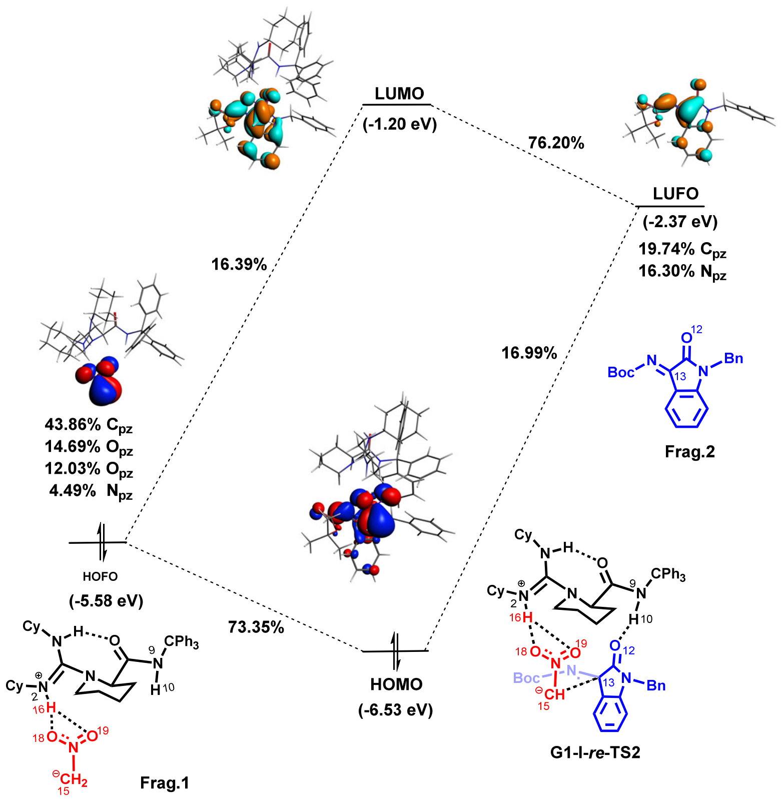

Figure S2. Schematic molecular orbital interaction diagram for transition state G1-I-*re*-TS2, constructed by G1-R2 (Frag.1) and *N*-Boc ketimine (Frag.2) fragments obtained by ADF program.

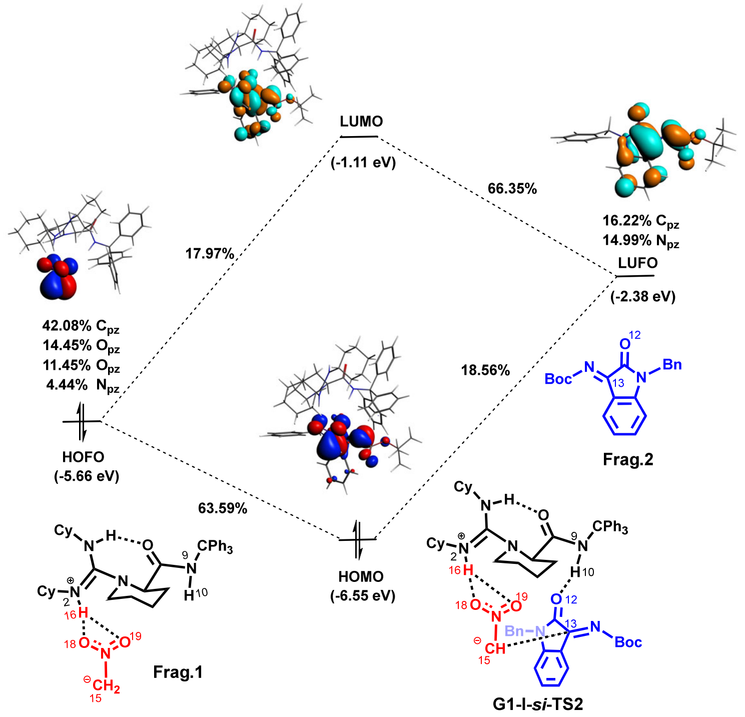

Figure S3. Schematic molecular orbital interaction diagram for transition state G1-I-*si*-TS2, constructed by G1-R2 (Frag.1) and *N*-Boc ketimine (Frag.2) fragments obtained by ADF program.

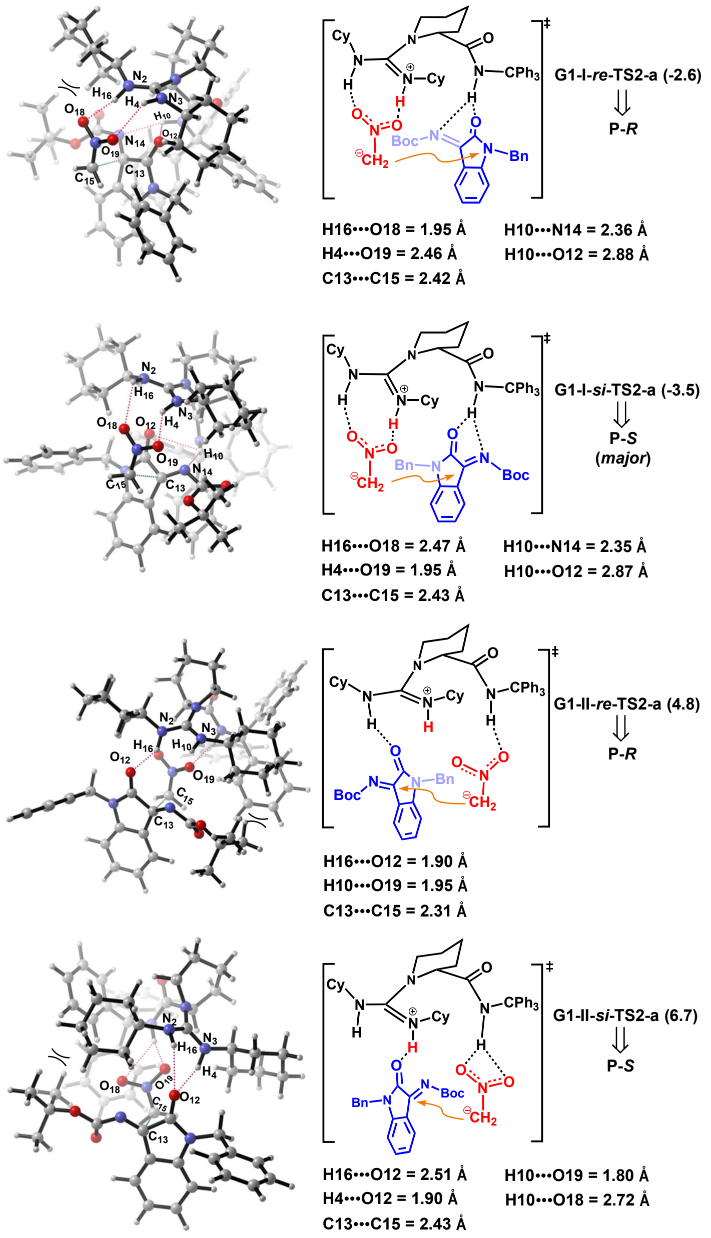

Figure S4. Optimized geometries of transition states G1-I-*re*-TS2-a ~ G1-II-*si*-TS2-a. Relative Gibbs free energies were given in kcal mol^{-1}.

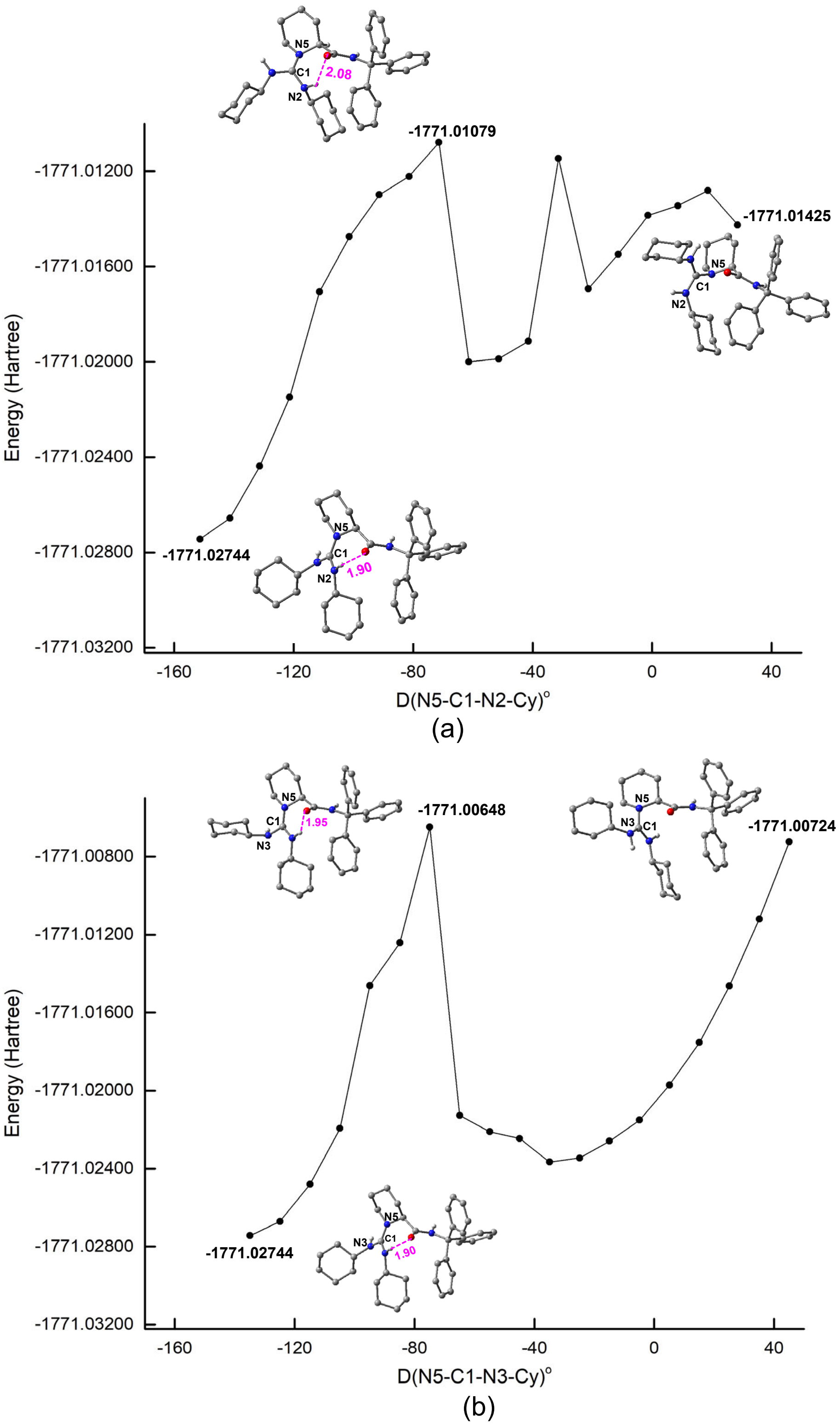

Figure S5. Relaxed potential energy scan of guanidine cation, calculated by scanning the dihedral angle N5-C1-N2-Cy (a) and N5-C1-N3-Cy (b) at M06-2X-D3/6-31G(d,p)(SMD, toluene) theoretical level.

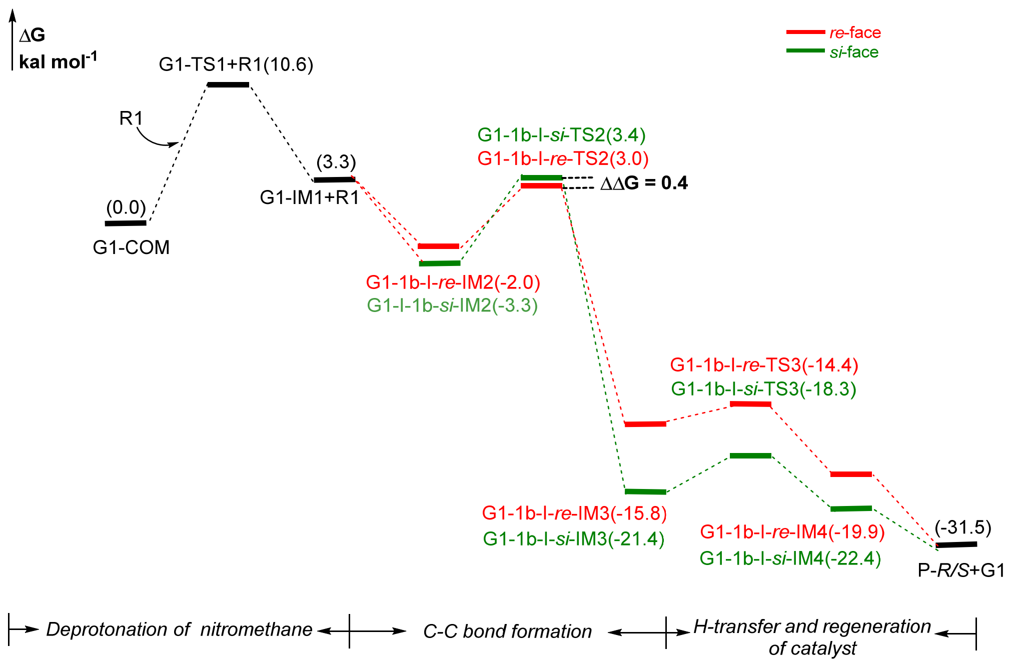

Figure S6. Energy profiles for aza-Henry reaction between *N*-COOEt ketimine (**R1b**) and nitromethane (**R2**) catalyzed by guanidine (**G1**) along *re*-face and *si*-face pathways in model I.

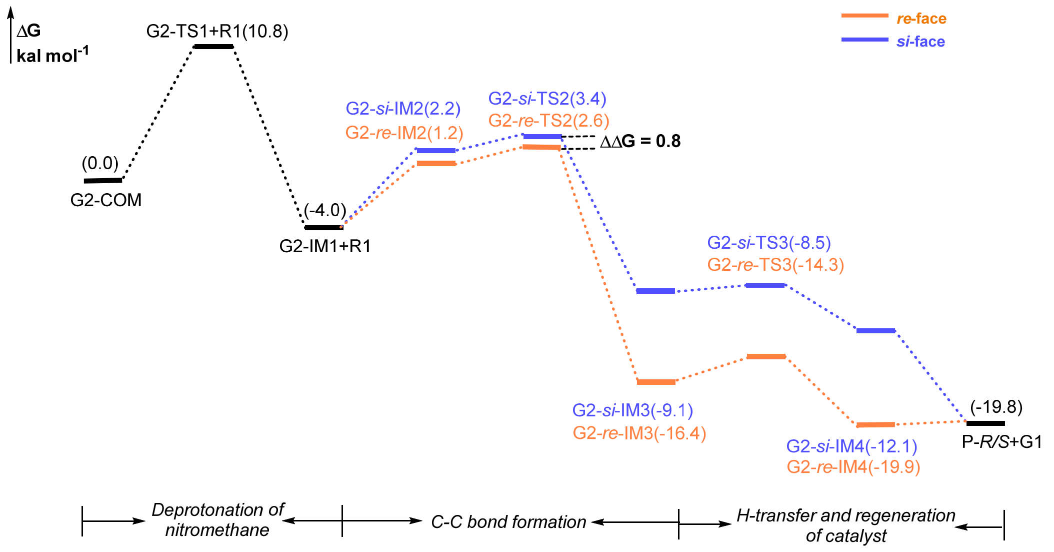

Figure S7. Energy profiles for aza-Henry reaction between *N*-Boc ketimine (**R1a**) and nitromethane (**R2**) catalyzed by guanidine (**G2**) along *re*-face and *si*-face pathways in model I.

Cartesian coordinates of all stationary points

G1

Zero-point correction= 0.80707 (a.u.)

Thermal correction to Gibbs Free Energy= 0.75608 (a.u.)

Sum of electronic and zero-point Energies= -1769.77996 (a.u.)

Sum of electronic and thermal Free Energies= -1769.83095 (a.u.)

Standard orientation:

Center Number	Atomic Number	Atomic Type	Coordinates (Angstroms) X	Y	Z
1	7	0	-1.684749	-1.365645	0.185522
2	7	0	-3.146769	0.174335	-0.783131
3	1	0	-3.327002	1.153886	-0.966339
4	8	0	1.483288	-2.541121	1.430142
5	7	0	-1.232497	0.891450	0.442129
6	7	0	1.083347	-0.790396	0.016164
7	1	0	0.387978	-0.046094	-0.085548
8	6	0	0.711561	-1.747675	0.915679
9	6	0	3.349354	-1.287969	-0.903391
10	6	0	-0.791001	-1.733272	1.281093
11	1	0	-0.887576	-0.957427	2.049510
12	6	0	2.762988	3.343865	-1.322287
13	1	0	3.152684	4.294183	-0.970077
14	6	0	-1.999695	-0.028188	-0.033908
15	6	0	-1.617554	2.290217	0.376644
16	1	0	-2.711616	2.413592	0.489159
17	6	0	2.371167	0.985547	-0.932624
18	6	0	3.753293	-3.409139	-1.999216
19	1	0	3.412022	-4.409537	-2.247538
20	6	0	2.860146	2.217752	-0.500890
21	1	0	3.326557	2.307716	0.474124
22	6	0	-4.378129	-0.588878	-0.546399
23	1	0	-4.172506	-1.647296	-0.745414
24	6	0	2.935167	-2.576573	-1.235552
25	1	0	1.976722	-2.942445	-0.889342
26	6	0	3.021920	-0.025226	1.311290
27	6	0	2.472803	-0.314999	-0.101138
28	6	0	4.595010	-0.846731	-1.367800
29	1	0	4.918275	0.167134	-1.145041
30	6	0	2.178001	3.251899	-2.579692
31	1	0	2.107386	4.128756	-3.215871
32	6	0	-1.186489	2.902654	-0.963421
33	1	0	-1.664685	2.371898	-1.796892

34	1	0	-0.106487	2.732052	-1.063057
35	6	0	-1.701581	-2.333719	-0.914479
36	1	0	-0.684029	-2.419183	-1.331721
37	1	0	-2.343328	-1.953962	-1.710601
38	6	0	-6.199975	-1.238893	1.074809
39	1	0	-6.010489	-2.310277	0.920330
40	1	0	-6.560108	-1.128164	2.102461
41	6	0	4.297309	-0.381722	1.742542
42	1	0	4.958194	-0.943911	1.092846
43	6	0	-4.890686	-0.468520	0.891720
44	1	0	-5.049405	0.595872	1.117757
45	1	0	-4.124586	-0.840348	1.581548
46	6	0	-2.159388	-3.697478	-0.408207
47	1	0	-3.199326	-3.620260	-0.063321
48	1	0	-2.133333	-4.419856	-1.230638
49	6	0	-1.214515	-3.091991	1.845269
50	1	0	-0.508263	-3.382257	2.625496
51	1	0	-2.204903	-2.975857	2.300346
52	6	0	5.415980	-1.679086	-2.119298
53	1	0	6.377983	-1.314805	-2.466336
54	6	0	1.782835	0.902174	-2.202248
55	1	0	1.411157	-0.058587	-2.549652
56	6	0	-1.267725	-4.155224	0.747646
57	1	0	-1.626444	-5.104551	1.157014
58	1	0	-0.249639	-4.329860	0.376791
59	6	0	-5.435837	-0.119157	-1.544284
60	1	0	-5.613791	0.955272	-1.385630
61	1	0	-5.052726	-0.236523	-2.563576
62	6	0	-7.260573	-0.766338	0.078888
63	1	0	-8.183509	-1.342741	0.200616
64	1	0	-7.509535	0.282431	0.290695
65	6	0	-1.252345	4.545597	1.472250
66	1	0	-2.331237	4.703847	1.609809
67	1	0	-0.750811	5.060388	2.298327
68	6	0	4.995755	-2.968350	-2.439918
69	1	0	5.629910	-3.619033	-3.033953
70	6	0	2.185508	0.665827	2.198555
71	1	0	1.177958	0.930423	1.882717
72	6	0	1.687535	2.021397	-3.019079
73	1	0	1.230121	1.934242	-3.999849
74	6	0	-1.479973	4.401393	-1.034327
75	1	0	-1.127045	4.803085	-1.990470
76	1	0	-2.566258	4.567283	-1.001904
77	6	0	4.733968	-0.038947	3.023419

78	1	0	5.729230	-0.333718	3.341804
79	6	0	-0.945239	3.048545	1.523973
80	1	0	-1.254592	2.610157	2.479555
81	1	0	0.139196	2.895279	1.431591
82	6	0	-6.750263	-0.880114	-1.359330
83	1	0	-7.499263	-0.505191	-2.063971
84	1	0	-6.588654	-1.938984	-1.602038
85	6	0	-0.821944	5.142719	0.130715
86	1	0	-1.062391	6.210527	0.090318
87	1	0	0.268873	5.053424	0.034205
88	6	0	2.620783	1.011520	3.470955
89	1	0	1.954546	1.550999	4.137840
90	6	0	3.904404	0.662265	3.889329
91	1	0	4.247647	0.928106	4.884264

R1a

Zero-point correction= 0.36828 (a.u.)

Thermal correction to Gibbs Free Energy= 0.32863(a.u.)

Sum of electronic and zero-point Energies= -1108.60379(a.u.)

Sum of electronic and thermal Free Energies= -1108.64344(a.u.)

Standard orientation:

Center Number	Atomic Number	Atomic Type	Coordinates (Angstroms) X	Y	Z
1	6	0	-1.748850	2.224338	-0.157155
2	6	0	-1.093556	1.090036	-0.603051
3	6	0	0.303967	0.962669	-0.501594
4	6	0	1.065221	1.988136	0.043332
5	6	0	0.417304	3.135728	0.492882
6	6	0	-0.968367	3.243307	0.392396
7	1	0	-2.829715	2.317679	-0.219015
8	1	0	2.148213	1.905848	0.102693
9	1	0	0.993602	3.951063	0.920686
10	1	0	-1.459527	4.145031	0.750429
11	7	0	-1.647703	-0.047400	-1.201569
12	6	0	-0.666241	-0.966573	-1.516532
13	8	0	-0.829534	-2.044875	-2.038947
14	6	0	-3.055479	-0.244105	-1.490743
15	1	0	-3.096051	-1.100115	-2.175313
16	6	0	0.649098	-0.342337	-1.054366
17	7	0	1.737312	-0.983681	-1.187340
18	6	0	2.961320	-0.415999	-0.814036

19	8	0	3.600396	0.307427	-1.543640
20	8	0	3.307363	-0.869205	0.386345
21	6	0	4.620851	-0.555609	0.968389
22	6	0	4.587152	-1.307875	2.285107
23	1	0	5.525712	-1.151456	2.828728
24	1	0	3.760388	-0.959142	2.914067
25	1	0	4.460712	-2.382741	2.115313
26	6	0	4.736673	0.938371	1.208293
27	1	0	4.767703	1.498279	0.269896
28	1	0	3.895679	1.294997	1.816047
29	1	0	5.660086	1.146711	1.761724
30	6	0	5.723592	-1.090370	0.074263
31	1	0	5.561895	-2.153850	-0.137039
32	1	0	5.780045	-0.546769	-0.871872
33	1	0	6.686219	-0.990705	0.589597
34	1	0	-3.434237	0.632070	-2.033942
35	6	0	-3.888486	-0.501668	-0.261920
36	6	0	-5.002472	0.286623	0.018578
37	6	0	-3.561859	-1.549793	0.600462
38	6	0	-5.782329	0.034214	1.143710
39	1	0	-5.263196	1.104286	-0.653799
40	6	0	-4.337061	-1.801515	1.724741
41	1	0	-2.697496	-2.175651	0.380691
42	6	0	-5.449382	-1.009243	1.999210
43	1	0	-6.649496	0.656228	1.353476
44	1	0	-4.075111	-2.622584	2.387988
45	1	0	-6.055052	-1.208402	2.880146

R2

Zero-point correction= 0.05133 (a.u.)

Thermal correction to Gibbs Free Energy= 0.03058 (a.u.)

Sum of electronic and zero-point Energies= -244.85717 (a.u.)

Sum of electronic and thermal Free Energies= -244.87791 (a.u.)

Standard orientation:

Center Number	Atomic Number	Atomic Type	Coordinates (Angstroms) X	Y	Z
1	6	0	1.317320	-0.038848	-0.000059
2	1	0	1.632107	-1.077758	-0.006737
3	1	0	1.648730	0.485530	0.895497
4	1	0	1.649750	0.498003	-0.887690
5	7	0	-0.169312	-0.000370	-0.000262

6	8	0	-0.767674	-1.058747	0.000071
7	8	0	-0.688492	1.099985	0.000069

G1-COM

Zero-point correction= 0.85996 (a.u.)
Thermal correction to Gibbs Free Energy= 0.80395 (a.u.)
Sum of electronic and zero-point Energies= -2014.63386 (a.u.)
Sum of electronic and thermal Free Energies= -2014.68987 (a.u.)

Standard orientation:

Center Number	Atomic Number	Atomic Type	Coordinates (Angstroms) X	Y	Z
1	6	0	0.007642	-4.737482	-1.126210
2	6	0	-1.458788	-4.629880	-1.521449
3	6	0	-2.122424	-3.485402	-0.769183
4	7	0	-1.457284	-2.201785	-1.028889
5	6	0	0.675199	-3.390044	-1.357901
6	6	0	-0.046036	-2.246172	-0.614291
7	1	0	0.520198	-5.516411	-1.699436
8	1	0	0.083054	-5.012559	-0.065329
9	1	0	-1.999246	-5.556888	-1.302996
10	1	0	-1.539337	-4.443769	-2.598875
11	1	0	-3.166720	-3.380654	-1.059125
12	1	0	-2.116737	-3.708507	0.313021
13	1	0	1.723997	-3.408400	-1.045195
14	1	0	0.657888	-3.134132	-2.422265
15	1	0	0.005706	-2.454528	0.467164
16	6	0	-2.263543	-1.071641	-0.741995
17	7	0	-3.332133	-1.254886	-0.045517
18	7	0	-1.806190	0.080033	-1.325150
19	6	0	-2.037796	1.442016	-0.858898
20	6	0	0.818721	-1.017429	-0.940073
21	8	0	0.964062	-0.645772	-2.097748
22	7	0	1.502309	-0.528599	0.125258
23	1	0	1.250047	-0.921253	1.025484
24	6	0	2.767491	0.247139	0.096642
25	6	0	-0.752282	2.112966	-0.371333
26	6	0	-1.067589	3.508470	0.170882
27	6	0	-1.703773	4.380630	-0.911606
28	6	0	-2.687011	2.295295	-1.955151
29	1	0	-0.041199	2.189531	-1.205809
30	1	0	-0.285791	1.487403	0.394411

31	1	0	-1.764022	3.414081	1.017238
32	1	0	-0.156711	3.975340	0.557395
33	1	0	-2.708818	1.381828	0.001099
34	1	0	-1.939180	5.376019	-0.519050
35	1	0	-3.607074	1.816213	-2.308597
36	1	0	-1.997210	2.326836	-2.810599
37	6	0	-2.966432	3.719180	-1.467971
38	1	0	-0.975509	4.518708	-1.723015
39	1	0	-3.389497	4.318080	-2.281574
40	1	0	-3.727937	3.682236	-0.675728
41	1	0	-1.068989	-0.042807	-2.009631
42	6	0	3.861794	-0.617373	-0.566591
43	6	0	3.909506	-1.980316	-0.254467
44	6	0	4.885734	-0.084551	-1.351571
45	6	0	4.917557	-2.798311	-0.750412
46	1	0	3.153779	-2.402124	0.401549
47	6	0	5.900978	-0.902444	-1.848129
48	1	0	4.904607	0.975642	-1.577451
49	6	0	5.916688	-2.261936	-1.559904
50	1	0	4.926275	-3.854002	-0.496816
51	1	0	6.681762	-0.466327	-2.463602
52	1	0	6.704594	-2.897398	-1.951596
53	6	0	2.597860	1.618920	-0.603658
54	6	0	2.532713	1.706679	-2.002173
55	6	0	2.518805	2.805547	0.129358
56	6	0	2.359349	2.931286	-2.636620
57	1	0	2.596532	0.803542	-2.593263
58	6	0	2.347664	4.035603	-0.507251
59	1	0	2.588083	2.790071	1.210679
60	6	0	2.257942	4.105133	-1.891486
61	1	0	2.304558	2.965952	-3.720433
62	1	0	2.288299	4.940173	0.090822
63	1	0	2.122634	5.061905	-2.386729
64	6	0	3.150666	0.461617	1.577254
65	6	0	4.489314	0.507001	1.973591
66	6	0	2.162006	0.684983	2.543216
67	6	0	4.831636	0.749157	3.301066
68	1	0	5.275664	0.345319	1.244376
69	6	0	2.505410	0.919086	3.872153
70	1	0	1.112324	0.690934	2.261946
71	6	0	3.841666	0.949533	4.258416
72	1	0	5.879074	0.773275	3.585285
73	1	0	1.720926	1.081850	4.604782
74	1	0	4.108947	1.130770	5.294744

75	6	0	-4.458783	-0.340742	-0.060720
76	6	0	-5.712948	-1.117710	-0.488108
77	6	0	-4.704216	0.279481	1.321090
78	1	0	-4.331929	0.474540	-0.788569
79	6	0	-6.952539	-0.220347	-0.503933
80	1	0	-5.855532	-1.949674	0.215480
81	1	0	-5.543432	-1.559554	-1.476256
82	6	0	-5.924404	1.202267	1.308212
83	1	0	-4.879880	-0.541419	2.031512
84	1	0	-3.806436	0.806626	1.665717
85	6	0	-7.175641	0.446202	0.855327
86	1	0	-7.835528	-0.799699	-0.794268
87	1	0	-6.821372	0.558507	-1.267852
88	1	0	-6.080293	1.641393	2.299515
89	1	0	-5.737654	2.037581	0.618544
90	1	0	-8.036410	1.122188	0.812860
91	1	0	-7.413693	-0.326266	1.599731
92	1	0	-2.923780	-1.963836	2.074378
93	6	0	-2.342907	-1.774237	2.986139
94	1	0	-2.087131	-2.677294	3.532799
95	1	0	-2.850889	-1.037051	3.606577
96	7	0	-1.085564	-1.150736	2.513690
97	8	0	-1.164447	-0.057674	1.985067
98	8	0	-0.042657	-1.775705	2.643636

G1-TS1

Zero-point correction= 0.85637 (a.u.)

Thermal correction to Gibbs Free Energy= 0.80298 (a.u.)

Sum of electronic and zero-point Energies= -2014.61996 (a.u.)

Sum of electronic and thermal Free Energies= -2014.67335 (a.u.)

Standard orientation:

Center Number	Atomic Number	Atomic Type	Coordinates (Angstroms) X	Y	Z
1	6	0	-0.283724	-4.179997	-2.454511
2	6	0	-1.717576	-4.216626	-1.922993
3	6	0	-1.852589	-3.462194	-0.596404
4	7	0	-1.337158	-2.093285	-0.730695
5	6	0	0.252119	-2.748348	-2.496334
6	6	0	0.077159	-2.109035	-1.115556
7	1	0	-0.232012	-4.638172	-3.446968
8	1	0	0.359835	-4.776376	-1.793887

9	1	0	-2.047046	-5.250896	-1.777687
10	1	0	-2.393628	-3.761613	-2.656989
11	1	0	-2.894190	-3.413725	-0.282678
12	1	0	-1.278986	-3.959342	0.197169
13	1	0	1.311673	-2.734030	-2.772323
14	1	0	-0.283352	-2.147319	-3.240722
15	1	0	0.541835	-2.778114	-0.378519
16	6	0	-2.178064	-1.009674	-0.694880
17	7	0	-3.196991	-1.112464	0.125705
18	7	0	-1.891947	0.035505	-1.511039
19	6	0	-2.172358	1.448148	-1.236532
20	6	0	0.877931	-0.791506	-1.049874
21	8	0	1.014372	-0.093031	-2.050226
22	7	0	1.520406	-0.600459	0.120366
23	1	0	1.263868	-1.230954	0.877474
24	6	0	2.745235	0.211306	0.281606
25	6	0	-1.261998	2.049224	-0.163608
26	6	0	-1.684966	3.497455	0.102684
27	6	0	-1.644745	4.331821	-1.180742
28	6	0	-2.061898	2.240164	-2.537924
29	1	0	-0.224846	2.021159	-0.523805
30	1	0	-1.303829	1.443941	0.749558
31	1	0	-2.708168	3.507188	0.508461
32	1	0	-1.036654	3.940413	0.864861
33	1	0	-3.205717	1.536125	-0.896196
34	1	0	-1.995944	5.350878	-0.984538
35	1	0	-2.691068	1.778135	-3.306741
36	1	0	-1.020656	2.197741	-2.889670
37	6	0	-2.474872	3.694021	-2.296842
38	1	0	-0.603440	4.408744	-1.515874
39	1	0	-2.376589	4.269774	-3.222879
40	1	0	-3.538298	3.717499	-2.020241
41	1	0	-1.105936	-0.103909	-2.138904
42	6	0	3.796179	-0.269872	-0.743624
43	6	0	3.823662	-1.612194	-1.132441
44	6	0	4.811257	0.570424	-1.209751
45	6	0	4.807468	-2.092899	-1.990143
46	1	0	3.072290	-2.298085	-0.752571
47	6	0	5.801211	0.090756	-2.065185
48	1	0	4.836100	1.610570	-0.901661
49	6	0	5.799529	-1.240712	-2.467140
50	1	0	4.799167	-3.138955	-2.281122
51	1	0	6.573967	0.766804	-2.417871
52	1	0	6.565603	-1.612666	-3.139984

53	6	0	2.421915	1.718257	0.129021
54	6	0	2.367014	2.313191	-1.138769
55	6	0	2.150075	2.515113	1.242956
56	6	0	2.070294	3.664348	-1.280846
57	1	0	2.546517	1.707504	-2.017853
58	6	0	1.837964	3.866480	1.100004
59	1	0	2.188467	2.093080	2.239921
60	6	0	1.804945	4.450019	-0.160701
61	1	0	2.041518	4.102864	-2.274225
62	1	0	1.630449	4.461372	1.984347
63	1	0	1.570986	5.504755	-0.272544
64	6	0	3.276293	-0.085692	1.703541
65	6	0	4.645847	-0.100255	1.980930
66	6	0	2.382139	-0.296686	2.760612
67	6	0	5.108882	-0.329946	3.274065
68	1	0	5.366863	0.056121	1.186989
69	6	0	2.847904	-0.531277	4.051435
70	1	0	1.309628	-0.262567	2.590233
71	6	0	4.213583	-0.550565	4.315523
72	1	0	6.178078	-0.342038	3.461693
73	1	0	2.131632	-0.695823	4.850445
74	1	0	4.576551	-0.735936	5.321686
75	6	0	-4.421839	-0.333680	0.120856
76	6	0	-5.614029	-1.283724	0.293291
77	6	0	-4.433198	0.708210	1.248589
78	1	0	-4.547741	0.168447	-0.848874
79	6	0	-6.937268	-0.514840	0.312748
80	1	0	-5.484956	-1.831593	1.237742
81	1	0	-5.605583	-2.024855	-0.513525
82	6	0	-5.742001	1.498631	1.254217
83	1	0	-4.320174	0.173207	2.201114
84	1	0	-3.568888	1.374494	1.167734
85	6	0	-6.943990	0.563276	1.399162
86	1	0	-7.771572	-1.208968	0.456624
87	1	0	-7.087735	-0.039203	-0.665977
88	1	0	-5.731663	2.238247	2.061553
89	1	0	-5.827835	2.059574	0.312077
90	1	0	-7.879179	1.131880	1.363828
91	1	0	-6.901304	0.080297	2.384949
92	1	0	-2.878632	-1.766404	1.196958
93	6	0	-2.294552	-2.237870	2.388774
94	1	0	-2.250401	-3.311902	2.539400
95	1	0	-2.816836	-1.682046	3.164039
96	7	0	-0.996960	-1.705583	2.223684

97	8	0	-0.861392	-0.478349	2.260794
98	8	0	-0.043885	-2.456224	1.947999

G1-IM1

Zero-point correction= 0.86066 (a.u.)

Thermal correction to Gibbs Free Energy= 0.80532 (a.u.)

Sum of electronic and zero-point Energies= -2014.62798 (a.u.)

Sum of electronic and thermal Free Energies= -2014.68332 (a.u.)

Standard orientation:

Center Number	Atomic Number	Atomic Type	Coordinates (Angstroms) X	Y	Z
1	6	0	-0.544681	-4.381415	-1.955578
2	6	0	-1.941250	-4.274168	-1.345442
3	6	0	-1.956326	-3.360550	-0.118989
4	7	0	-1.398430	-2.042956	-0.469600
5	6	0	0.058779	-2.997159	-2.198099
6	6	0	0.003176	-2.183456	-0.902659
7	1	0	-0.579483	-4.952774	-2.887885
8	1	0	0.107178	-4.932571	-1.265195
9	1	0	-2.312745	-5.261183	-1.051664
10	1	0	-2.640944	-3.870438	-2.087550
11	1	0	-2.974835	-3.245623	0.245924
12	1	0	-1.339100	-3.758048	0.696139
13	1	0	1.101584	-3.078331	-2.519430
14	1	0	-0.478038	-2.457676	-2.986270
15	1	0	0.456791	-2.770757	-0.097884
16	6	0	-2.128025	-0.918486	-0.442249
17	7	0	-3.229805	-0.904820	0.329635
18	7	0	-1.792035	0.146378	-1.181606
19	6	0	-2.041305	1.538313	-0.781451
20	1	0	-3.152798	-1.457933	1.182220
21	6	0	0.868681	-0.910904	-1.044183
22	8	0	1.007219	-0.390160	-2.150560
23	7	0	1.526949	-0.577087	0.075888
24	1	0	1.198203	-1.058867	0.925032
25	6	0	2.798417	0.173712	0.128716
26	6	0	-0.786539	2.204491	-0.219519
27	6	0	-1.139075	3.622220	0.239186
28	6	0	-1.698414	4.450391	-0.918740
29	6	0	-2.606415	2.332887	-1.959318
30	1	0	-0.015244	2.246952	-1.000747

31	1	0	-0.397866	1.596062	0.602871
32	1	0	-1.889552	3.563230	1.040955
33	1	0	-0.255421	4.103587	0.665777
34	1	0	-2.776802	1.510120	0.023944
35	1	0	-1.959963	5.459004	-0.581288
36	1	0	-3.500139	1.838273	-2.358211
37	1	0	-1.857428	2.340199	-2.763762
38	6	0	-2.921647	3.769711	-1.536380
39	1	0	-0.917807	4.559826	-1.684205
40	1	0	-3.291259	4.339817	-2.394811
41	1	0	-3.732704	3.750775	-0.794349
42	1	0	-1.049607	0.016727	-1.864574
43	6	0	3.840185	-0.567816	-0.738473
44	6	0	3.791449	-1.961525	-0.835807
45	6	0	4.911566	0.097819	-1.339986
46	6	0	4.759532	-2.666457	-1.543457
47	1	0	2.993135	-2.505220	-0.339830
48	6	0	5.885286	-0.606330	-2.046086
49	1	0	4.992972	1.176462	-1.256370
50	6	0	5.810029	-1.990469	-2.158621
51	1	0	4.694544	-3.748421	-1.607309
52	1	0	6.703959	-0.065193	-2.510429
53	1	0	6.565648	-2.537835	-2.713183
54	6	0	2.623397	1.646632	-0.323989
55	6	0	2.569256	1.974417	-1.686530
56	6	0	2.522222	2.685919	0.603759
57	6	0	2.397706	3.290827	-2.100735
58	1	0	2.649054	1.187131	-2.424515
59	6	0	2.353449	4.006947	0.188734
60	1	0	2.579350	2.478322	1.665579
61	6	0	2.282924	4.316812	-1.164050
62	1	0	2.357661	3.514555	-3.162668
63	1	0	2.279792	4.792699	0.934960
64	1	0	2.152384	5.345390	-1.487093
65	6	0	3.251278	0.125288	1.605372
66	6	0	4.603964	0.073763	1.949874
67	6	0	2.300291	0.203584	2.631036
68	6	0	4.999034	0.093399	3.285323
69	1	0	5.362177	0.006630	1.177342
70	6	0	2.699694	0.218746	3.964458
71	1	0	1.237414	0.255617	2.401322
72	6	0	4.049384	0.164565	4.299196
73	1	0	6.056103	0.046954	3.528882
74	1	0	1.943711	0.273330	4.741963

75	1	0	4.357982	0.174123	5.340101
76	6	0	-4.500344	-0.229189	0.087800
77	6	0	-5.593152	-1.243775	-0.272642
78	6	0	-4.913818	0.578505	1.321498
79	1	0	-4.366647	0.443006	-0.766852
80	6	0	-6.935495	-0.544263	-0.500865
81	1	0	-5.686063	-1.963803	0.553156
82	1	0	-5.296516	-1.809197	-1.162545
83	6	0	-6.253753	1.281536	1.098376
84	1	0	-5.002704	-0.118626	2.167502
85	1	0	-4.127213	1.292387	1.587988
86	6	0	-7.346873	0.280544	0.719709
87	1	0	-7.704621	-1.284767	-0.741900
88	1	0	-6.850010	0.118451	-1.372833
89	1	0	-6.535751	1.837821	1.997559
90	1	0	-6.142657	2.019847	0.292060
91	1	0	-8.289362	0.802330	0.525076
92	1	0	-7.525440	-0.394922	1.567405
93	6	0	-1.871152	-1.948458	3.040479
94	1	0	-1.763231	-2.959761	3.398147
95	1	0	-2.615657	-1.258101	3.406883
96	7	0	-0.856815	-1.427958	2.367885
97	8	0	-0.881891	-0.212273	1.985459
98	8	0	0.159707	-2.151834	2.062744

G1-I-*re*-IM2

Zero-point correction= 1.23103 (a.u.)

Thermal correction to Gibbs Free Energy= 1.15827 (a.u.)

Sum of electronic and zero-point Energies= -3123.26367 (a.u.)

Sum of electronic and thermal Free Energies= -3123.33644 (a.u.)

Standard orientation:

Center Number	Atomic Number	Atomic Type	Coordinates (Angstroms) X	Y	Z
1	6	0	-0.053989	2.196340	-4.569003
2	6	0	-1.005839	2.908569	-3.603826
3	6	0	-1.156251	2.142600	-2.288156
4	7	0	0.173130	1.906500	-1.692826
5	6	0	1.281812	1.858567	-3.898691
6	6	0	1.006408	1.100418	-2.599125
7	1	0	0.113458	2.805866	-5.462430
8	1	0	-0.522705	1.261371	-4.903109

9	1	0	-1.996378	3.014837	-4.058911
10	1	0	-0.636160	3.920735	-3.394359
11	1	0	-1.777486	2.687432	-1.579083
12	1	0	-1.625087	1.167877	-2.437852
13	1	0	1.904314	1.242676	-4.555271
14	1	0	1.850781	2.770307	-3.680629
15	1	0	0.366206	0.242260	-2.840938
16	6	0	0.623935	2.690967	-0.674680
17	7	0	-0.245045	2.977290	0.282229
18	7	0	1.895258	3.132904	-0.695798
19	6	0	2.747355	3.474808	0.450868
20	1	0	-1.074554	2.315362	0.360367
21	6	0	2.294843	0.533648	-1.970951
22	8	0	3.352899	1.147500	-2.065372
23	7	0	2.107881	-0.679944	-1.411387
24	1	0	1.137930	-0.984328	-1.358648
25	6	0	2.783951	2.376518	1.513396
26	6	0	3.691730	2.790636	2.671454
27	6	0	5.107661	3.075345	2.168403
28	6	0	4.157466	3.744036	-0.078844
29	1	0	3.174905	1.462437	1.045933
30	1	0	1.774556	2.135064	1.863236
31	1	0	3.284229	3.692556	3.151573
32	1	0	3.701897	2.000912	3.428729
33	1	0	2.381132	4.407292	0.901943
34	1	0	5.753653	3.394725	2.992796
35	1	0	4.120982	4.534262	-0.837882
36	1	0	4.521141	2.831345	-0.570673
37	6	0	5.095191	4.135779	1.064488
38	1	0	5.532800	2.142885	1.771132
39	1	0	6.104953	4.296551	0.673828
40	1	0	4.762768	5.093008	1.488474
41	1	0	2.424226	2.872694	-1.522184
42	6	0	4.481714	-1.454357	-1.239693
43	6	0	3.043247	-1.567750	-0.685937
44	6	0	2.973586	-1.251269	0.823381
45	6	0	2.505171	-2.997769	-0.918574
46	6	0	5.020317	-2.410366	-2.100759
47	6	0	6.324532	-2.286711	-2.584408
48	6	0	7.115139	-1.211475	-2.204110
49	6	0	6.592170	-0.253489	-1.335297
50	6	0	5.293817	-0.376211	-0.863416
51	1	0	4.434629	-3.271902	-2.399692
52	1	0	6.717375	-3.044516	-3.255031

53	1	0	8.130708	-1.117833	-2.576205
54	1	0	7.195459	0.595549	-1.028402
55	1	0	4.893189	0.380688	-0.198045
56	6	0	1.915479	-3.339315	-2.142283
57	6	0	1.483519	-4.639347	-2.389290
58	6	0	2.646359	-3.992264	0.052674
59	6	0	2.214817	-5.294175	-0.194773
60	6	0	1.634331	-5.624408	-1.415827
61	1	0	1.029032	-4.879671	-3.345510
62	1	0	1.804598	-2.583552	-2.913729
63	1	0	1.297918	-6.638996	-1.606735
64	1	0	2.331779	-6.048553	0.577043
65	1	0	3.085773	-3.754065	1.015131
66	6	0	1.755573	-0.857882	1.383086
67	6	0	1.645500	-0.610500	2.749333
68	6	0	2.746792	-0.775205	3.583915
69	6	0	4.069958	-1.436420	1.673178
70	6	0	3.958461	-1.196168	3.040719
71	1	0	0.865967	-0.706833	0.775533
72	1	0	0.685208	-0.276332	3.130525
73	1	0	2.663411	-0.580183	4.649064
74	1	0	4.824610	-1.340471	3.679709
75	1	0	5.020522	-1.770503	1.270927
76	6	0	-0.196153	4.052413	1.266352
77	6	0	-0.221123	5.435189	0.610587
78	6	0	-1.381285	3.870923	2.216646
79	1	0	0.712490	3.972068	1.877890
80	6	0	-0.219776	6.532128	1.677544
81	1	0	-1.131487	5.504497	-0.000736
82	1	0	0.632437	5.547050	-0.069634
83	6	0	-1.378941	4.964917	3.283311
84	1	0	-2.311719	3.907689	1.635148
85	1	0	-1.326762	2.868994	2.656745
86	6	0	-1.389451	6.357969	2.649033
87	1	0	-0.257646	7.517693	1.202482
88	1	0	0.724955	6.487562	2.238156
89	1	0	-2.240315	4.840202	3.947063
90	1	0	-0.480923	4.860336	3.907980
91	1	0	-1.355611	7.130938	3.424418
92	1	0	-2.332185	6.495428	2.102273
93	8	0	-5.107699	0.797598	-0.804042
94	8	0	-0.778683	-1.309989	-1.196273
95	7	0	-2.022510	-3.036123	-0.278248
96	7	0	-3.324850	-0.170107	-1.785138

97	8	0	-4.804466	1.031153	-3.043324
98	6	0	-5.848665	2.040083	-0.584872
99	6	0	-3.233454	-1.273392	-1.174585
100	6	0	-1.837309	-1.832762	-0.900592
101	6	0	-4.175416	-2.275153	-0.655100
102	6	0	-3.391462	-3.317942	-0.130766
103	6	0	-5.559987	-2.353546	-0.631343
104	1	0	-6.165291	-1.547002	-1.031647
105	6	0	-4.490942	0.575486	-1.968834
106	6	0	-6.152664	-3.485629	-0.069540
107	1	0	-7.233398	-3.567832	-0.036170
108	6	0	-0.943821	-3.910163	0.143696
109	1	0	-0.027546	-3.472058	-0.263183
110	6	0	-3.966929	-4.443168	0.434829
111	1	0	-3.360403	-5.235838	0.858517
112	6	0	-5.363390	-4.507767	0.455366
113	1	0	-5.840426	-5.377446	0.896106
114	6	0	-6.068000	2.031612	0.921521
115	6	0	-4.971721	3.224971	-0.976895
116	6	0	-7.165507	2.000518	-1.348095
117	1	0	-6.989385	1.977143	-2.424286
118	1	0	-7.756354	2.888705	-1.104055
119	1	0	-7.745527	1.117395	-1.062106
120	1	0	-6.638564	2.914480	1.222936
121	1	0	-5.099755	2.038859	1.431322
122	1	0	-6.622506	1.138589	1.225956
123	1	0	-5.453291	4.151893	-0.651472
124	1	0	-4.818974	3.276090	-2.056703
125	1	0	-4.005278	3.128793	-0.471004
126	1	0	-1.079318	-4.893130	-0.320963
127	6	0	-0.845181	-4.041222	1.646690
128	6	0	-0.751093	-5.298721	2.242041
129	6	0	-0.823471	-2.896013	2.446226
130	6	0	-0.640838	-5.417911	3.625867
131	1	0	-0.763359	-6.189527	1.617447
132	6	0	-0.720252	-3.017783	3.828916
133	1	0	-0.882494	-1.904464	1.995603
134	6	0	-0.630716	-4.275984	4.422186
135	1	0	-0.568970	-6.401266	4.080397
136	1	0	-0.705759	-2.122019	4.443002
137	1	0	-0.550761	-4.365706	5.501163
138	6	0	-3.191201	-0.312707	1.587313
139	1	0	-2.978288	-1.096667	2.297260
140	1	0	-4.162346	-0.116086	1.165723

141	7	0	-2.219689	0.516338	1.283451
142	8	0	-2.445618	1.501684	0.471152
143	8	0	-1.042463	0.388138	1.761515

G1-I-*si*-IM2

Zero-point correction= 1.23047 (a.u.)

Thermal correction to Gibbs Free Energy= 1.15840 (a.u.)

Sum of electronic and zero-point Energies= -3123.26651 (a.u.)

Sum of electronic and thermal Free Energies= -3123.33858 (a.u.)

Standard orientation:

Center Number	Atomic Number	Atomic Type	Coordinates (Angstroms) X	Y	Z
1	6	0	-1.748439	3.381984	-2.593659
2	6	0	-0.672642	3.455091	-3.686333
3	6	0	0.348598	4.570145	-3.446070
4	6	0	-0.334592	5.921813	-3.234492
5	6	0	-2.390370	4.757044	-2.358599
6	1	0	-1.187603	3.629999	-4.639069
7	1	0	-0.177688	2.479315	-3.748678
8	1	0	0.958013	4.313822	-2.571432
9	1	0	1.035851	4.617073	-4.297624
10	1	0	-2.517169	2.679695	-2.937598
11	1	0	0.410553	6.698139	-3.030596
12	1	0	-2.957133	5.013287	-3.263424
13	6	0	-1.346911	5.842596	-2.090707
14	1	0	-0.858011	6.217042	-4.154989
15	1	0	-1.845262	6.806648	-1.945060
16	1	0	-0.814644	5.615649	-1.156684
17	6	0	-0.415704	4.008541	3.346523
18	6	0	0.092163	4.549157	2.006286
19	6	0	0.368855	3.428983	1.001958
20	7	0	-0.819286	2.572653	0.873483
21	6	0	-1.596362	3.048042	3.162453
22	6	0	-1.185130	1.973624	2.159429
23	1	0	-0.693436	4.834342	4.008231
24	1	0	0.399779	3.464287	3.842372
25	1	0	1.012922	5.124776	2.150406
26	1	0	-0.653439	5.232213	1.579294
27	1	0	0.645470	3.824000	0.025922
28	1	0	1.191378	2.786828	1.331766
29	1	0	-1.874250	2.577418	4.109264

30	1	0	-2.480955	3.587027	2.801135
31	1	0	-0.244087	1.529803	2.518250
32	6	0	-1.658178	2.675570	-0.191754
33	7	0	-1.110237	2.863024	-1.380447
34	7	0	-2.987321	2.582327	0.020022
35	6	0	-3.996804	2.041321	-0.902892
36	1	0	-0.091330	2.531259	-1.463450
37	6	0	-2.164140	0.782021	2.064692
38	8	0	-3.252032	0.842252	2.620845
39	7	0	-1.629909	-0.249008	1.369428
40	1	0	-0.697249	-0.048948	1.009742
41	6	0	-3.577865	0.694082	-1.485593
42	6	0	-4.668756	0.126716	-2.392559
43	6	0	-5.988189	-0.014709	-1.632189
44	6	0	-5.304545	1.899293	-0.120983
45	1	0	-3.401157	0.008472	-0.649576
46	1	0	-2.625024	0.770501	-2.020249
47	1	0	-4.812397	0.791349	-3.256614
48	1	0	-4.340881	-0.841830	-2.783057
49	1	0	-4.165536	2.761075	-1.715677
50	1	0	-6.776893	-0.383799	-2.296007
51	1	0	-5.604548	2.876045	0.276802
52	1	0	-5.120429	1.238968	0.738973
53	6	0	-6.408265	1.318168	-1.007240
54	1	0	-5.861896	-0.766812	-0.841149
55	1	0	-7.324193	1.196668	-0.420055
56	1	0	-6.638242	2.034240	-1.807879
57	1	0	-3.113487	4.705509	-1.536764
58	1	0	-3.280593	2.602408	0.989943
59	6	0	-3.095294	-2.085466	2.247026
60	6	0	-1.879293	-1.713185	1.391046
61	6	0	-2.083413	-2.195818	-0.062383
62	6	0	-0.571314	-2.313818	1.956910
63	6	0	-3.010093	-2.962856	3.323874
64	6	0	-4.159401	-3.344530	4.022450
65	6	0	-5.403256	-2.860196	3.644360
66	6	0	-5.499525	-1.987356	2.558091
67	6	0	-4.357710	-1.611863	1.869388
68	1	0	-2.048715	-3.363502	3.625414
69	1	0	-4.071175	-4.030220	4.859408
70	1	0	-6.295357	-3.158741	4.186266
71	1	0	-6.466477	-1.602319	2.248093
72	1	0	-4.432308	-0.936179	1.022736
73	6	0	0.017746	-1.729782	3.087006

74	6	0	1.226461	-2.198972	3.585748
75	6	0	0.068061	-3.395724	1.357431
76	6	0	1.284754	-3.865842	1.854189
77	6	0	1.872882	-3.267517	2.961010
78	1	0	1.669952	-1.727238	4.457732
79	1	0	-0.474349	-0.892188	3.574985
80	1	0	2.828030	-3.623594	3.334760
81	1	0	1.784158	-4.683714	1.343359
82	1	0	-0.346475	-3.846704	0.462861
83	6	0	-1.321265	-1.624436	-1.085937
84	6	0	-1.444166	-2.068919	-2.398442
85	6	0	-2.336951	-3.093159	-2.711862
86	6	0	-2.953869	-3.241929	-0.379162
87	6	0	-3.085379	-3.683091	-1.696234
88	1	0	-0.607276	-0.827940	-0.901653
89	1	0	-0.826965	-1.598173	-3.159249
90	1	0	-2.448140	-3.430446	-3.738713
91	1	0	-3.778641	-4.487651	-1.923006
92	1	0	-3.540741	-3.717687	0.399781
93	6	0	2.854678	0.652473	-2.300429
94	1	0	2.977128	-0.288270	-2.815129
95	1	0	3.663885	1.310068	-2.023640
96	7	0	1.625094	1.074052	-2.107075
97	8	0	1.432276	2.220595	-1.510934
98	8	0	0.608085	0.412881	-2.467578
99	8	0	1.273670	-4.097355	-1.513329
100	8	0	1.186386	0.271724	0.850308
101	7	0	3.441875	0.073443	1.275643
102	7	0	1.646819	-1.988950	-0.904969
103	8	0	2.557448	-2.801123	-2.864972
104	1	0	4.161462	0.688910	3.124369
105	6	0	1.341771	-5.285751	-2.359683
106	6	0	2.593363	-1.391124	-0.312297
107	6	0	2.266269	-0.250262	0.649312
108	6	0	4.051278	-1.589289	-0.208531
109	6	0	4.507055	-0.682613	0.765527
110	6	0	4.946563	-2.449717	-0.830329
111	1	0	4.607406	-3.138542	-1.595309
112	6	0	1.910247	-2.979369	-1.855229
113	6	0	6.293397	-2.390070	-0.468200
114	1	0	7.007806	-3.051005	-0.946479
115	6	0	3.561813	1.090078	2.299784
116	1	0	2.545610	1.253047	2.677076
117	6	0	5.842263	-0.602430	1.123919

118	1	0	6.189577	0.121345	1.853393
119	6	0	6.729097	-1.476750	0.490315
120	1	0	7.782551	-1.435251	0.748760
121	6	0	4.154443	2.386522	1.786625
122	6	0	5.094596	3.090815	2.537464
123	6	0	3.724816	2.901298	0.561292
124	6	0	5.597114	4.305934	2.072924
125	1	0	5.436631	2.690663	3.489541
126	6	0	4.225818	4.114976	0.100523
127	1	0	2.980776	2.374469	-0.035301
128	6	0	5.162987	4.820035	0.854118
129	1	0	6.331867	4.846849	2.661328
130	1	0	3.876984	4.503310	-0.852130
131	1	0	5.555417	5.765070	0.491465
132	6	0	0.760708	-4.991064	-3.737646
133	1	0	1.391245	-4.302496	-4.300339
134	1	0	0.673342	-5.928187	-4.295969
135	1	0	-0.237291	-4.556719	-3.632313
136	6	0	0.458317	-6.274544	-1.610400
137	1	0	0.855590	-6.468312	-0.609566
138	1	0	-0.555459	-5.874512	-1.513201
139	1	0	0.410657	-7.221597	-2.154652
140	6	0	2.781560	-5.781602	-2.431680
141	1	0	2.803816	-6.762776	-2.914808
142	1	0	3.406375	-5.098887	-3.010083
143	1	0	3.197647	-5.887348	-1.424509

G1-II-*re*-IM2

Zero-point correction= 1.23085 (a.u.)

Thermal correction to Gibbs Free Energy= 1.15846 (a.u.)

Sum of electronic and zero-point Energies= -3123.26401 (a.u.)

Sum of electronic and thermal Free Energies= -3123.33640 (a.u.)

Standard orientation:

Center Number	Atomic Number	Atomic Type	Coordinates (Angstroms) X	Y	Z
1	6	0	1.693406	1.166746	4.976826
2	6	0	0.242183	0.670551	4.936910
3	6	0	-0.329873	0.605814	3.514760
4	7	0	0.595763	-0.158624	2.669530
5	6	0	2.586077	0.433654	3.966026
6	6	0	1.897223	0.511879	2.602333

7	1	0	2.095467	1.066193	5.989536
8	1	0	1.710824	2.236670	4.731871
9	1	0	-0.393431	1.321170	5.546746
10	1	0	0.187947	-0.332403	5.379410
11	1	0	-1.308772	0.131364	3.509836
12	1	0	-0.441346	1.593455	3.055457
13	1	0	3.573960	0.903271	3.913681
14	1	0	2.740508	-0.612823	4.256757
15	1	0	1.624784	1.557435	2.435465
16	6	0	0.344142	-1.421976	2.297074
17	7	0	-0.917957	-1.759118	2.000031
18	7	0	1.343813	-2.313782	2.251325
19	6	0	1.402307	-3.508009	1.400447
20	1	0	-1.487049	-0.989190	1.637222
21	6	0	2.817247	0.141788	1.420277
22	8	0	3.625653	-0.784047	1.478166
23	7	0	2.721323	1.051923	0.435792
24	1	0	1.835118	1.590752	0.451275
25	6	0	3.521114	1.084969	-0.787377
26	6	0	1.308227	-3.190786	-0.092399
27	6	0	1.391026	-4.499201	-0.884672
28	6	0	2.689781	-5.247981	-0.582470
29	6	0	2.714815	-4.224490	1.720390
30	1	0	2.144388	-2.529972	-0.357621
31	1	0	0.385562	-2.639309	-0.311751
32	1	0	0.533529	-5.139515	-0.624331
33	1	0	1.318381	-4.290911	-1.955083
34	1	0	0.578975	-4.178140	1.673966
35	1	0	2.731035	-6.188075	-1.143249
36	1	0	2.769614	-4.427758	2.796038
37	1	0	3.542730	-3.547440	1.467012
38	6	0	2.836201	-5.518370	0.915559
39	1	0	3.533171	-4.631103	-0.919335
40	1	0	3.795638	-6.000657	1.128200
41	1	0	2.051078	-6.216610	1.237276
42	1	0	2.262936	-1.948752	2.488280
43	6	0	5.014955	1.296111	-0.470413
44	6	0	5.459785	1.646924	0.803126
45	6	0	5.950720	1.252470	-1.510514
46	6	0	6.806687	1.916844	1.039901
47	1	0	4.751469	1.723696	1.619805
48	6	0	7.294927	1.516368	-1.276397
49	1	0	5.623532	1.009187	-2.517380
50	6	0	7.731483	1.846374	0.005041

51	1	0	7.128457	2.183936	2.041876
52	1	0	8.001542	1.469803	-2.099349
53	1	0	8.780513	2.054418	0.190844
54	6	0	3.238971	-0.179695	-1.618813
55	6	0	4.159203	-1.217577	-1.766053
56	6	0	1.983298	-0.302766	-2.226321
57	6	0	3.864813	-2.314419	-2.577611
58	1	0	5.114496	-1.170466	-1.255018
59	6	0	1.683069	-1.401307	-3.022932
60	1	0	1.237729	0.474253	-2.091661
61	6	0	2.635714	-2.399538	-3.224427
62	1	0	4.606012	-3.097709	-2.706151
63	1	0	0.702696	-1.466130	-3.485102
64	1	0	2.414482	-3.245234	-3.869998
65	6	0	3.093673	2.360974	-1.552566
66	6	0	3.140906	2.425194	-2.947758
67	6	0	2.741462	3.514779	-0.845826
68	6	0	2.825117	3.603230	-3.618346
69	1	0	3.409603	1.543741	-3.520707
70	6	0	2.421238	4.691351	-1.516767
71	1	0	2.719759	3.502173	0.239106
72	6	0	2.458634	4.741315	-2.906486
73	1	0	2.860762	3.625426	-4.703158
74	1	0	2.146209	5.573274	-0.946358
75	1	0	2.206518	5.658025	-3.430172
76	6	0	-1.611581	-2.993337	2.363915
77	6	0	-2.792509	-2.651301	3.281735
78	6	0	-2.102609	-3.756211	1.131090
79	1	0	-0.907824	-3.616159	2.930106
80	6	0	-3.575806	-3.905306	3.676357
81	1	0	-3.445199	-1.949431	2.744700
82	1	0	-2.422475	-2.130415	4.172625
83	6	0	-2.862731	-5.020499	1.534611
84	1	0	-2.756703	-3.090461	0.556165
85	1	0	-1.257856	-3.996741	0.476363
86	6	0	-4.044061	-4.680441	2.443934
87	1	0	-4.428674	-3.628375	4.304415
88	1	0	-2.931619	-4.554059	4.285777
89	1	0	-3.209875	-5.547938	0.639959
90	1	0	-2.183358	-5.705485	2.062553
91	1	0	-4.571938	-5.591251	2.745668
92	1	0	-4.759764	-4.065267	1.882054
93	8	0	-2.925801	-2.739661	-1.897166
94	8	0	-2.835942	0.542402	1.752422

95	7	0	-4.088728	2.093998	0.587779
96	7	0	-2.767291	-0.860755	-0.719287
97	8	0	-2.083545	-0.909073	-2.935399
98	6	0	-2.804479	-3.618956	-3.056297
99	6	0	-3.321774	0.279716	-0.637904
100	6	0	-3.350923	0.952852	0.728738
101	6	0	-4.063713	1.179995	-1.539699
102	6	0	-4.502737	2.255885	-0.743656
103	6	0	-4.391428	1.138103	-2.889271
104	1	0	-4.042917	0.325656	-3.515560
105	6	0	-2.568223	-1.460814	-1.970984
106	6	0	-5.154040	2.176123	-3.426239
107	1	0	-5.413796	2.161334	-4.478972
108	6	0	-4.302846	3.055885	1.652896
109	1	0	-4.061770	2.528038	2.580444
110	6	0	-5.245383	3.299899	-1.267653
111	1	0	-5.547124	4.141318	-0.653354
112	6	0	-5.566585	3.240166	-2.626213
113	1	0	-6.144371	4.046985	-3.066082
114	6	0	-3.778071	-3.166770	-4.137889
115	1	0	-3.495275	-2.189131	-4.533084
116	1	0	-3.767961	-3.886135	-4.961893
117	1	0	-4.795708	-3.114667	-3.739424
118	6	0	-3.206540	-4.972022	-2.484123
119	1	0	-2.489518	-5.287802	-1.719831
120	1	0	-4.200646	-4.916244	-2.031671
121	1	0	-3.223507	-5.724688	-3.276788
122	6	0	-1.363046	-3.657706	-3.555176
123	1	0	-1.222240	-4.545778	-4.178983
124	1	0	-1.115662	-2.772551	-4.140412
125	1	0	-0.677745	-3.723104	-2.704387
126	6	0	-0.955346	1.867387	-1.260107
127	1	0	-1.002930	2.944564	-1.302098
128	1	0	-1.281595	1.205784	-2.048355
129	7	0	-0.316101	1.325869	-0.243627
130	8	0	-0.156537	0.072732	-0.139962
131	8	0	0.188286	2.099787	0.681016
132	1	0	-5.364028	3.325339	1.680479
133	6	0	-3.434497	4.284812	1.492941
134	6	0	-3.972533	5.567178	1.580547
135	6	0	-2.068644	4.123682	1.253777
136	6	0	-3.150460	6.683950	1.434505
137	1	0	-5.037311	5.695696	1.762924
138	6	0	-1.251563	5.237409	1.099308

139	1	0	-1.617899	3.135186	1.182859
140	6	0	-1.789722	6.521012	1.189616
141	1	0	-3.576801	7.680043	1.503405
142	1	0	-0.195101	5.083088	0.901183
143	1	0	-1.152366	7.391458	1.066888

G1-II-*si*-IM2

Zero-point correction= 1.23141 (a.u.)

Thermal correction to Gibbs Free Energy= 1.15815 (a.u.)

Sum of electronic and zero-point Energies= -3123.25996 (a.u.)

Sum of electronic and thermal Free Energies= -3123.33321 (a.u.)

Standard orientation:

Center Number	Atomic Number	Atomic Type	Coordinates (Angstroms) X	Y	Z
1	6	0	-0.064912	-1.770375	-4.563722
2	6	0	1.272966	-2.349900	-4.127712
3	6	0	1.685987	-1.777460	-2.777705
4	7	0	0.657620	-2.084665	-1.753259
5	6	0	-1.109873	-2.023698	-3.482450
6	6	0	-0.647833	-1.476474	-2.120325
7	1	0	-0.392181	-2.205974	-5.513218
8	1	0	0.039444	-0.688084	-4.697469
9	1	0	2.058334	-2.115047	-4.853224
10	1	0	1.206504	-3.442848	-4.049032
11	1	0	2.626551	-2.230484	-2.463932
12	1	0	1.812165	-0.689917	-2.838400
13	1	0	-2.036286	-1.506199	-3.747702
14	1	0	-1.333519	-3.092755	-3.379129
15	1	0	-0.442267	-0.408536	-2.236653
16	6	0	1.069531	-2.402880	-0.506393
17	7	0	2.312372	-2.036514	-0.143183
18	7	0	0.281247	-3.106301	0.308052
19	6	0	0.392678	-3.178977	1.764525
20	1	0	2.637122	-1.186456	-0.604520
21	6	0	-1.840618	-1.650173	-1.176063
22	8	0	-2.218802	-2.744966	-0.750680
23	7	0	-2.544125	-0.508337	-1.051474
24	1	0	-2.046458	0.356533	-1.292132
25	6	0	-3.863300	-0.409024	-0.413029
26	6	0	0.192478	-1.830380	2.457691
27	6	0	0.403245	-1.997432	3.965678

28	6	0	-0.501184	-3.083718	4.555516
29	6	0	-0.607501	-4.206216	2.287779
30	1	0	-0.825477	-1.472542	2.241503
31	1	0	0.888315	-1.092519	2.044093
32	1	0	1.455080	-2.260253	4.150351
33	1	0	0.225315	-1.042942	4.473513
34	1	0	1.389100	-3.554910	2.022559
35	1	0	-0.261658	-3.241072	5.612674
36	1	0	-0.480389	-5.153388	1.751162
37	1	0	-1.624219	-3.842006	2.085747
38	6	0	-0.394640	-4.405501	3.790249
39	1	0	-1.537970	-2.737032	4.509195
40	1	0	-1.121460	-5.125915	4.178929
41	1	0	0.599999	-4.844528	3.950327
42	1	0	-0.619169	-3.375792	-0.095064
43	6	0	-4.877857	-1.399525	-1.024916
44	6	0	-4.725345	-1.884772	-2.322589
45	6	0	-6.053409	-1.713261	-0.335468
46	6	0	-5.698713	-2.691745	-2.904425
47	1	0	-3.841143	-1.624324	-2.891106
48	6	0	-7.029358	-2.518030	-0.914534
49	1	0	-6.211341	-1.330346	0.667949
50	6	0	-6.854525	-3.017229	-2.201948
51	1	0	-5.550125	-3.063963	-3.913452
52	1	0	-7.930127	-2.751344	-0.355144
53	1	0	-7.613481	-3.647848	-2.654175
54	6	0	-3.683175	-0.608203	1.104821
55	6	0	-3.825911	-1.860580	1.710735
56	6	0	-3.309623	0.476392	1.901080
57	6	0	-3.666822	-2.000653	3.087704
58	1	0	-4.074478	-2.723614	1.103427
59	6	0	-3.128793	0.334129	3.274254
60	1	0	-3.183633	1.454504	1.448641
61	6	0	-3.328161	-0.903647	3.877946
62	1	0	-3.807136	-2.977047	3.543563
63	1	0	-2.852423	1.203262	3.865649
64	1	0	-3.215729	-1.015570	4.952548
65	6	0	-4.452004	0.978738	-0.754263
66	6	0	-5.458415	1.525877	0.049873
67	6	0	-4.102045	1.647127	-1.930030
68	6	0	-6.089071	2.712731	-0.301616
69	1	0	-5.750621	1.021778	0.965663
70	6	0	-4.739695	2.836115	-2.284088
71	1	0	-3.324181	1.261124	-2.579842

72	6	0	-5.735516	3.373547	-1.476275
73	1	0	-6.861158	3.118462	0.344773
74	1	0	-4.447051	3.339873	-3.200213
75	1	0	-6.230505	4.298880	-1.754731
76	6	0	3.292101	-2.751535	0.672328
77	6	0	3.331870	-4.247576	0.349222
78	6	0	4.661177	-2.120169	0.408483
79	1	0	3.069414	-2.617152	1.742184
80	6	0	4.400928	-4.947466	1.190024
81	1	0	3.565809	-4.355545	-0.719059
82	1	0	2.352839	-4.710838	0.507813
83	6	0	5.749005	-2.814868	1.229246
84	1	0	4.882312	-2.216216	-0.665118
85	1	0	4.624729	-1.047927	0.633471
86	6	0	5.777428	-4.321208	0.961132
87	1	0	4.420522	-6.015027	0.950319
88	1	0	4.134543	-4.867203	2.253422
89	1	0	6.725222	-2.371199	1.005227
90	1	0	5.559871	-2.637951	2.296522
91	1	0	6.527229	-4.804517	1.595684
92	1	0	6.079819	-4.494935	-0.080160
93	6	0	2.462641	1.669665	-0.360245
94	6	0	1.116435	1.272972	0.233988
95	8	0	0.459172	0.285984	-0.023776
96	6	0	1.802960	3.228167	1.201446
97	6	0	2.795514	2.971984	0.238264
98	7	0	3.125663	0.826237	-1.049105
99	6	0	4.424954	1.148029	-1.489653
100	8	0	4.698290	1.374438	-2.641417
101	8	0	5.281625	1.070737	-0.464836
102	6	0	6.727988	1.151701	-0.679416
103	6	0	7.111435	2.517732	-1.236813
104	1	0	8.199502	2.568476	-1.338818
105	1	0	6.663069	2.687573	-2.216682
106	1	0	6.803252	3.311779	-0.550004
107	6	0	7.174051	0.012038	-1.587426
108	1	0	6.782891	0.130538	-2.598686
109	1	0	8.266697	-0.006830	-1.634121
110	1	0	6.833718	-0.945808	-1.180474
111	6	0	7.281014	0.970365	0.727338
112	1	0	8.372959	1.020350	0.710075
113	1	0	6.904056	1.753509	1.390945
114	1	0	6.982080	-0.000963	1.132733
115	7	0	0.844932	2.209492	1.206001

116	6	0	-0.276226	2.119408	2.118759
117	1	0	-0.713259	1.128475	1.949678
118	6	0	3.829047	3.875248	0.036400
119	1	0	4.575002	3.701014	-0.732414
120	6	0	3.870780	5.028998	0.820791
121	1	0	4.664210	5.753382	0.673963
122	6	0	2.889878	5.261095	1.785274
123	1	0	2.934430	6.164535	2.385072
124	6	0	1.835519	4.367760	1.989759
125	1	0	1.059231	4.569386	2.719545
126	1	0	0.100108	2.154254	3.148860
127	6	0	-1.318583	3.192189	1.898933
128	6	0	-1.817111	3.928371	2.972102
129	6	0	-1.832674	3.402349	0.615534
130	6	0	-2.827350	4.869681	2.772401
131	1	0	-1.414675	3.765147	3.970032
132	6	0	-2.837084	4.344738	0.419074
133	1	0	-1.473194	2.809922	-0.227984
134	6	0	-3.336900	5.079004	1.495015
135	1	0	-3.210601	5.438018	3.614426
136	1	0	-3.238518	4.491719	-0.578351
137	1	0	-4.124685	5.809038	1.336279
138	6	0	0.766717	3.095227	-2.042598
139	1	0	1.729023	3.284789	-2.491193
140	1	0	0.219789	3.816293	-1.454865
141	7	0	0.120001	2.016887	-2.461557
142	8	0	-1.088712	1.800311	-2.075308
143	8	0	0.684711	1.180119	-3.222454

G1-I-*re*-TS2

Zero-point correction= 1.23040 (a.u.)

Thermal correction to Gibbs Free Energy= 1.15765 (a.u.)

Sum of electronic and zero-point Energies= -3123.26127 (a.u.)

Sum of electronic and thermal Free Energies= -3123.33402 (a.u.)

Standard orientation:

Center Number	Atomic Number	Atomic Type	Coordinates (Angstroms) X	Y	Z
1	6	0	-0.178449	2.208353	-4.492282
2	6	0	-0.988347	3.028220	-3.485363
3	6	0	-1.102133	2.315002	-2.138320
4	7	0	0.249561	1.982138	-1.636835

5	6	0	1.173111	1.782598	-3.912155
6	6	0	0.944798	1.080854	-2.573056
7	1	0	-0.032299	2.772779	-5.418481
8	1	0	-0.747344	1.306636	-4.752652
9	1	0	-2.002146	3.199525	-3.860465
10	1	0	-0.519779	4.010807	-3.342068
11	1	0	-1.618964	2.935036	-1.407753
12	1	0	-1.665799	1.382518	-2.216860
13	1	0	1.696263	1.103765	-4.593054
14	1	0	1.826715	2.650147	-3.759984
15	1	0	0.222770	0.268652	-2.727807
16	6	0	0.819709	2.693185	-0.639402
17	7	0	0.026989	3.072055	0.363463
18	7	0	2.125136	3.002621	-0.693511
19	6	0	3.037488	3.265955	0.427492
20	1	0	-0.829205	2.506518	0.481859
21	6	0	2.241907	0.442652	-2.031692
22	8	0	3.328585	0.960917	-2.268852
23	7	0	2.011115	-0.710362	-1.370315
24	1	0	1.026518	-0.943878	-1.249030
25	6	0	2.976084	2.184670	1.506164
26	6	0	3.976775	2.484786	2.621922
27	6	0	5.397105	2.587729	2.064374
28	6	0	4.451914	3.371705	-0.148629
29	1	0	3.218586	1.221833	1.038988
30	1	0	1.960959	2.083932	1.904606
31	1	0	3.707454	3.432155	3.111632
32	1	0	3.915420	1.701877	3.383716
33	1	0	2.786840	4.238741	0.873800
34	1	0	6.107739	2.826202	2.862580
35	1	0	4.481630	4.167037	-0.902740
36	1	0	4.686142	2.429419	-0.662198
37	6	0	5.473842	3.641365	0.956625
38	1	0	5.689938	1.608645	1.659220
39	1	0	6.479428	3.674912	0.525382
40	1	0	5.283389	4.632570	1.390229
41	1	0	2.595457	2.738327	-1.554260
42	6	0	4.359799	-1.571071	-1.244206
43	6	0	2.918840	-1.669924	-0.702764
44	6	0	2.868315	-1.422552	0.821515
45	6	0	2.331895	-3.066406	-1.005922
46	6	0	4.895508	-2.518473	-2.114350
47	6	0	6.209772	-2.404286	-2.574564
48	6	0	7.009070	-1.347788	-2.162601

49	6	0	6.486393	-0.397287	-1.284161
50	6	0	5.180595	-0.512705	-0.834332
51	1	0	4.299436	-3.363474	-2.439716
52	1	0	6.602777	-3.154818	-3.253164
53	1	0	8.031511	-1.262193	-2.517284
54	1	0	7.097133	0.437839	-0.953359
55	1	0	4.779612	0.234662	-0.157709
56	6	0	1.745931	-3.322718	-2.251818
57	6	0	1.258070	-4.587824	-2.564951
58	6	0	2.412830	-4.109123	-0.080324
59	6	0	1.924712	-5.376557	-0.393887
60	6	0	1.347084	-5.622451	-1.635945
61	1	0	0.806170	-4.761719	-3.536593
62	1	0	1.678872	-2.525027	-2.985207
63	1	0	0.964918	-6.609640	-1.877661
64	1	0	1.990366	-6.167708	0.346820
65	1	0	2.843977	-3.935269	0.899581
66	6	0	1.684213	-0.969379	1.408226
67	6	0	1.605980	-0.759458	2.783166
68	6	0	2.702078	-1.023394	3.598978
69	6	0	3.957927	-1.709739	1.651626
70	6	0	3.876197	-1.509128	3.027437
71	1	0	0.802129	-0.744211	0.814887
72	1	0	0.674637	-0.378132	3.192374
73	1	0	2.643553	-0.857314	4.670607
74	1	0	4.736819	-1.733310	3.650496
75	1	0	4.880910	-2.089502	1.225725
76	8	0	-5.257971	0.950592	-0.663492
77	8	0	-0.859356	-1.091223	-0.931768
78	7	0	-2.170726	-2.858134	-0.216014
79	7	0	-3.321898	0.148367	-1.564995
80	8	0	-4.452967	1.827618	-2.594181
81	6	0	-6.202901	2.032026	-0.428574
82	6	0	-3.306095	-0.925360	-0.846306
83	6	0	-1.944149	-1.589969	-0.680307
84	6	0	-4.291424	-1.980138	-0.494030
85	6	0	-3.545184	-3.115390	-0.122912
86	6	0	-5.677299	-2.048044	-0.521119
87	1	0	-6.257626	-1.181400	-0.814861
88	6	0	-4.375523	1.021904	-1.687628
89	6	0	-6.301356	-3.243428	-0.156137
90	1	0	-7.384050	-3.308860	-0.171532
91	6	0	-1.124222	-3.822937	0.052096
92	1	0	-0.204481	-3.387286	-0.349954

93	6	0	-4.151409	-4.302239	0.256320
94	1	0	-3.562616	-5.160008	0.563964
95	6	0	-5.547442	-4.348775	0.233244
96	1	0	-6.050071	-5.266260	0.522759
97	6	0	-6.872274	1.621121	0.877859
98	6	0	-5.444027	3.342893	-0.245867
99	6	0	-7.225795	2.100046	-1.557862
100	1	0	-6.750083	2.380903	-2.497510
101	1	0	-7.994255	2.839558	-1.311653
102	1	0	-7.717593	1.129755	-1.683469
103	1	0	-7.644575	2.346216	1.149629
104	1	0	-6.138740	1.576863	1.688272
105	1	0	-7.340793	0.637077	0.779078
106	1	0	-6.125928	4.109773	0.134329
107	1	0	-5.015980	3.685750	-1.188959
108	1	0	-4.634736	3.193127	0.475100
109	1	0	-1.333447	-4.741887	-0.506566
110	6	0	-0.954591	-4.122284	1.524411
111	6	0	-0.851045	-5.439210	1.970750
112	6	0	-0.856391	-3.074625	2.442732
113	6	0	-0.652542	-5.713115	3.322409
114	1	0	-0.922390	-6.253574	1.252608
115	6	0	-0.658718	-3.349041	3.792773
116	1	0	-0.922538	-2.042851	2.097571
117	6	0	-0.557264	-4.666897	4.235709
118	1	0	-0.576133	-6.741855	3.661039
119	1	0	-0.574722	-2.529671	4.500743
120	1	0	-0.403083	-4.876792	5.289542
121	6	0	-3.208686	-0.259299	1.384129
122	1	0	-2.983885	-1.115053	2.004726
123	1	0	-4.207763	0.123074	1.252569
124	7	0	-2.222949	0.651571	1.257161
125	8	0	-2.475390	1.738613	0.658393
126	8	0	-1.054071	0.405210	1.651962
127	6	0	0.195408	4.193920	1.281451
128	6	0	0.359807	5.526316	0.546720
129	6	0	-1.025466	4.232528	2.202809
130	1	0	1.074902	4.031243	1.920699
131	6	0	0.502107	6.671586	1.550606
132	1	0	-0.529090	5.682079	-0.079870
133	1	0	1.225379	5.489283	-0.124580
134	6	0	-0.894079	5.375671	3.209371
135	1	0	-1.925638	4.369430	1.588851
136	1	0	-1.128318	3.263548	2.703670

137	6	0	-0.695123	6.719331	2.503090
138	1	0	0.609890	7.622828	1.020080
139	1	0	1.424128	6.527744	2.131956
140	1	0	-1.780456	5.408155	3.850433
141	1	0	-0.035465	5.180736	3.866349
142	1	0	-0.564588	7.519475	3.238861
143	1	0	-1.598993	6.958522	1.927257

G1-I-*si*-TS2

Zero-point correction= 1.23138 (a.u.)

Thermal correction to Gibbs Free Energy= 1.16053 (a.u.)

Sum of electronic and zero-point Energies= -3123.26218 (a.u.)

Sum of electronic and thermal Free Energies= -3123.33304 (a.u.)

Standard orientation:

Center Number	Atomic Number	Atomic Type	Coordinates (Angstroms) X	Y	Z
1	6	0	1.052111	-3.551298	-2.702348
2	6	0	0.009309	-3.318179	-3.803893
3	6	0	-1.255549	-4.158595	-3.614537
4	6	0	-0.919603	-5.643049	-3.467631
5	6	0	1.340497	-5.046859	-2.513597
6	1	0	0.480634	-3.576653	-4.759699
7	1	0	-0.234355	-2.250194	-3.834155
8	1	0	-1.797273	-3.808745	-2.726480
9	1	0	-1.925508	-3.997657	-4.465358
10	1	0	1.972419	-3.036919	-3.002363
11	1	0	-1.832077	-6.226267	-3.304833
12	1	0	1.861828	-5.396474	-3.414060
13	6	0	0.061777	-5.863162	-2.315564
14	1	0	-0.468444	-6.009613	-4.400231
15	1	0	0.314318	-6.923993	-2.218387
16	1	0	-0.422258	-5.566573	-1.375028
17	6	0	-0.581399	-3.982573	3.225231
18	6	0	-1.119878	-4.417067	1.858601
19	6	0	-1.110293	-3.270881	0.844202
20	7	0	0.235288	-2.680291	0.780567
21	6	0	0.779916	-3.286577	3.105673
22	6	0	0.650477	-2.155842	2.088350
23	1	0	-0.513906	-4.843903	3.896274
24	1	0	-1.292800	-3.280559	3.681217
25	1	0	-2.145208	-4.790375	1.953097

26	1	0	-0.509060	-5.241220	1.468111
27	1	0	-1.406076	-3.617086	-0.145450
28	1	0	-1.800162	-2.470749	1.127290
29	1	0	1.098008	-2.875020	4.067788
30	1	0	1.554951	-3.996283	2.791666
31	1	0	-0.198528	-1.529881	2.393967
32	6	0	1.069728	-2.919997	-0.258345
33	7	0	0.530378	-2.943102	-1.473918
34	7	0	2.375455	-3.130182	-0.019391
35	6	0	3.503276	-2.806915	-0.907402
36	1	0	-0.357153	-2.409121	-1.549375
37	6	0	1.871881	-1.208370	2.037838
38	8	0	2.951612	-1.580977	2.483254
39	7	0	1.544848	-0.013678	1.503929
40	1	0	0.578828	0.041630	1.179390
41	6	0	3.418919	-1.374865	-1.426117
42	6	0	4.631905	-1.029420	-2.288696
43	6	0	5.931751	-1.241967	-1.512103
44	6	0	4.792902	-3.015466	-0.112105
45	1	0	3.380526	-0.708773	-0.556115
46	1	0	2.485961	-1.208466	-1.975331
47	1	0	4.635325	-1.662957	-3.187376
48	1	0	4.547127	0.007429	-2.627734
49	1	0	3.509369	-3.511216	-1.750318
50	1	0	6.797337	-1.030895	-2.148479
51	1	0	4.846354	-4.052995	0.237797
52	1	0	4.756774	-2.370643	0.777533
53	6	0	6.016158	-2.668355	-0.963429
54	1	0	5.969377	-0.526265	-0.678914
55	1	0	6.925960	-2.800412	-0.369397
56	1	0	6.080931	-3.373651	-1.803260
57	1	0	2.026317	-5.194465	-1.671439
58	1	0	2.630125	-3.197793	0.960962
59	6	0	3.436808	1.375046	2.393911
60	6	0	2.190069	1.324567	1.497760
61	6	0	2.546159	1.701024	0.043329
62	6	0	1.081262	2.275928	2.007171
63	6	0	3.520291	2.202678	3.511306
64	6	0	4.698826	2.276337	4.258769
65	6	0	5.810100	1.530691	3.892293
66	6	0	5.741657	0.706481	2.767384
67	6	0	4.570318	0.636567	2.030130
68	1	0	2.670589	2.807597	3.806029
69	1	0	4.739347	2.928863	5.125303

70	1	0	6.726954	1.590901	4.470579
71	1	0	6.603660	0.119065	2.465324
72	1	0	4.524267	-0.000469	1.153122
73	6	0	0.321083	1.908181	3.126348
74	6	0	-0.706712	2.720803	3.589427
75	6	0	0.788439	3.478524	1.368791
76	6	0	-0.246102	4.291612	1.829702
77	6	0	-0.997560	3.918991	2.936411
78	1	0	-1.286126	2.416895	4.456195
79	1	0	0.537851	0.972589	3.635879
80	1	0	-1.811548	4.547939	3.283508
81	1	0	-0.476835	5.207185	1.294206
82	1	0	1.325760	3.767937	0.473211
83	6	0	1.667760	1.348290	-0.985285
84	6	0	1.920833	1.735084	-2.297315
85	6	0	3.058200	2.480818	-2.604311
86	6	0	3.668817	2.473374	-0.266511
87	6	0	3.928364	2.853746	-1.582926
88	1	0	0.758246	0.788483	-0.789659
89	1	0	1.210173	1.449345	-3.068176
90	1	0	3.262804	2.774241	-3.630473
91	1	0	4.812467	3.443910	-1.805215
92	1	0	4.348855	2.786363	0.518657
93	6	0	-2.886698	0.336601	-2.071581
94	1	0	-2.824881	1.158859	-2.771195
95	1	0	-3.827085	-0.131001	-1.814250
96	7	0	-1.816980	-0.499324	-2.074259
97	8	0	-1.924853	-1.644372	-1.527310
98	8	0	-0.722665	-0.122260	-2.531862
99	8	0	-0.233453	4.164930	-1.585661
100	8	0	-1.253752	-0.052386	0.815855
101	7	0	-3.462420	0.497720	1.147980
102	7	0	-1.227742	2.338098	-0.799165
103	8	0	-2.043900	3.381870	-2.701095
104	1	0	-4.214511	0.144426	3.051006
105	6	0	-0.022241	5.274053	-2.496036
106	6	0	-2.342508	1.747673	-0.467869
107	6	0	-2.235608	0.624055	0.551907
108	6	0	-3.745673	2.245019	-0.332225
109	6	0	-4.362831	1.450890	0.651004
110	6	0	-4.468002	3.265775	-0.935410
111	1	0	-4.010091	3.872740	-1.706706
112	6	0	-1.255588	3.310656	-1.765715
113	6	0	-5.793763	3.475270	-0.544528

114	1	0	-6.366464	4.270013	-1.010721
115	6	0	-3.741066	-0.413344	2.235213
116	1	0	-2.764987	-0.758887	2.593896
117	6	0	-5.681678	1.631959	1.033512
118	1	0	-6.146682	0.987276	1.772330
119	6	0	-6.391335	2.667198	0.419157
120	1	0	-7.427041	2.836306	0.696628
121	6	0	-4.603035	-1.594301	1.837966
122	6	0	-5.558558	-2.090585	2.724718
123	6	0	-4.418434	-2.222685	0.603558
124	6	0	-6.314998	-3.212170	2.391437
125	1	0	-5.710941	-1.597329	3.682012
126	6	0	-5.174163	-3.344620	0.273094
127	1	0	-3.673445	-1.845595	-0.095718
128	6	0	-6.122122	-3.842909	1.165327
129	1	0	-7.057275	-3.589471	3.088010
130	1	0	-5.021333	-3.827709	-0.687500
131	1	0	-6.711894	-4.715779	0.903586
132	6	0	0.259141	4.772569	-3.909445
133	1	0	-0.619758	4.295495	-4.342375
134	1	0	0.553919	5.616137	-4.541492
135	1	0	1.082887	4.053629	-3.889345
136	6	0	1.220258	5.942687	-1.918841
137	1	0	1.031376	6.289595	-0.898275
138	1	0	2.050615	5.230203	-1.895241
139	1	0	1.509383	6.802577	-2.529736
140	6	0	-1.215002	6.225992	-2.455486
141	1	0	-0.972761	7.141448	-3.003795
142	1	0	-2.096783	5.770268	-2.907473
143	1	0	-1.445610	6.499054	-1.420715

G1-II-*re*-TS2

Zero-point correction= 1.23145 (a.u.)

Thermal correction to Gibbs Free Energy= 1.15966 (a.u.)

Sum of electronic and zero-point Energies= -3123.26115 (a.u.)

Sum of electronic and thermal Free Energies= -3123.33293 (a.u.)

Standard orientation:

Center Number	Atomic Number	Atomic Type	Coordinates (Angstroms) X	Y	Z
1	6	0	1.983519	0.592386	4.912902
2	6	0	0.467048	0.387787	4.964541

3	6	0	-0.187262	0.514974	3.585653
4	7	0	0.485915	-0.390230	2.643285
5	6	0	2.630421	-0.272451	3.826777
6	6	0	1.897468	-0.017703	2.504178
7	1	0	2.428774	0.380447	5.889712
8	1	0	2.195323	1.645565	4.686855
9	1	0	0.007898	1.113281	5.644078
10	1	0	0.247013	-0.610801	5.363153
11	1	0	-1.244499	0.266934	3.634554
12	1	0	-0.110855	1.527302	3.174433
13	1	0	3.690961	-0.022248	3.716371
14	1	0	2.575128	-1.338442	4.078865
15	1	0	1.856181	1.063781	2.348308
16	6	0	-0.090632	-1.528786	2.222760
17	7	0	-1.416221	-1.536553	2.028674
18	7	0	0.649916	-2.625930	2.028361
19	6	0	0.336632	-3.721191	1.100645
20	1	0	-1.814634	-0.631657	1.759734
21	6	0	2.703802	-0.578827	1.317761
22	8	0	3.225337	-1.690951	1.343758
23	7	0	2.943221	0.365097	0.384896
24	1	0	2.242427	1.110426	0.347129
25	6	0	3.824867	0.194901	-0.771415
26	6	0	0.264271	-3.262085	-0.355374
27	6	0	-0.030829	-4.464758	-1.255038
28	6	0	1.008202	-5.573370	-1.081091
29	6	0	1.408597	-4.796272	1.282918
30	1	0	1.229605	-2.810569	-0.623281
31	1	0	-0.495124	-2.480687	-0.473080
32	1	0	-1.026422	-4.865374	-1.007422
33	1	0	-0.072080	-4.139068	-2.299023
34	1	0	-0.628642	-4.157904	1.381442
35	1	0	0.756716	-6.435951	-1.707912
36	1	0	1.454170	-5.098248	2.335709
37	1	0	2.381897	-4.357712	1.020515
38	6	0	1.121295	-5.998720	0.383433
39	1	0	1.981968	-5.198395	-1.420445
40	1	0	1.906006	-6.751940	0.506735
41	1	0	0.179000	-6.466665	0.700519
42	1	0	1.645134	-2.524257	2.216206
43	6	0	5.280587	-0.042382	-0.323338
44	6	0	5.692978	0.157447	0.992731
45	6	0	6.251146	-0.350568	-1.283230
46	6	0	7.032932	0.019421	1.349642

47	1	0	4.969662	0.436043	1.750418
48	6	0	7.587593	-0.493131	-0.929281
49	1	0	5.956687	-0.480240	-2.321014
50	6	0	7.985131	-0.313615	0.393923
51	1	0	7.327744	0.174655	2.383103
52	1	0	8.320598	-0.737556	-1.691964
53	1	0	9.028343	-0.423642	0.672849
54	6	0	3.264416	-0.919940	-1.671585
55	6	0	3.851162	-2.181325	-1.776593
56	6	0	2.082187	-0.669372	-2.377761
57	6	0	3.315068	-3.141489	-2.634488
58	1	0	4.733124	-2.419437	-1.193029
59	6	0	1.537496	-1.630307	-3.221852
60	1	0	1.589872	0.294313	-2.279492
61	6	0	2.166583	-2.865217	-3.370171
62	1	0	3.801655	-4.108265	-2.725113
63	1	0	0.621263	-1.409819	-3.760498
64	1	0	1.755799	-3.611829	-4.043818
65	6	0	3.859357	1.556390	-1.502255
66	6	0	4.026570	1.637639	-2.886951
67	6	0	3.834811	2.743163	-0.762676
68	6	0	4.139730	2.873353	-3.517808
69	1	0	4.055611	0.730446	-3.481344
70	6	0	3.945997	3.978195	-1.394080
71	1	0	3.742959	2.707174	0.318918
72	6	0	4.094827	4.049605	-2.775774
73	1	0	4.260053	2.912669	-4.595974
74	1	0	3.925435	4.886627	-0.799996
75	1	0	4.179764	5.012691	-3.269023
76	6	0	-2.351270	-2.582622	2.438681
77	6	0	-3.256670	-2.037970	3.551146
78	6	0	-3.188712	-3.078379	1.258731
79	1	0	-1.763233	-3.408710	2.856589
80	6	0	-4.282228	-3.081606	3.997683
81	1	0	-3.771736	-1.146822	3.165830
82	1	0	-2.639564	-1.716160	4.398374
83	6	0	-4.202239	-4.131860	1.709217
84	1	0	-3.703609	-2.216399	0.818057
85	1	0	-2.531905	-3.468454	0.471972
86	6	0	-5.108482	-3.587982	2.814676
87	1	0	-4.932948	-2.658567	4.769902
88	1	0	-3.754891	-3.928815	4.457686
89	1	0	-4.803199	-4.458521	0.854494
90	1	0	-3.669406	-5.018994	2.081001

91	1	0	-5.812774	-4.358208	3.146871
92	1	0	-5.705228	-2.759239	2.410901
93	8	0	-3.851583	-1.946116	-1.712137
94	8	0	-2.315253	1.258030	1.728042
95	7	0	-3.093534	3.092869	0.566105
96	7	0	-2.999355	-0.187120	-0.641534
97	8	0	-2.610251	-0.478993	-2.909119
98	6	0	-4.177519	-2.773045	-2.863278
99	6	0	-2.942743	1.109017	-0.638015
100	6	0	-2.717817	1.782891	0.703195
101	6	0	-3.517337	2.169643	-1.509972
102	6	0	-3.584528	3.336848	-0.724070
103	6	0	-3.983179	2.198317	-2.818224
104	1	0	-3.922035	1.313005	-3.439146
105	6	0	-3.123411	-0.830449	-1.858678
106	6	0	-4.507987	3.391750	-3.321194
107	1	0	-4.871812	3.426102	-4.342481
108	6	0	-3.047979	4.065400	1.639500
109	1	0	-2.816381	3.494580	2.543933
110	6	0	-4.087689	4.530444	-1.215370
111	1	0	-4.107982	5.426780	-0.604601
112	6	0	-4.553147	4.538883	-2.532409
113	1	0	-4.951883	5.460270	-2.944979
114	6	0	-4.977203	-1.964998	-3.881623
115	1	0	-4.348463	-1.225365	-4.379030
116	1	0	-5.390277	-2.638865	-4.638059
117	1	0	-5.809454	-1.453709	-3.387645
118	6	0	-5.038573	-3.871636	-2.251588
119	1	0	-4.460811	-4.433922	-1.511414
120	1	0	-5.916046	-3.443657	-1.758247
121	1	0	-5.375286	-4.564567	-3.027516
122	6	0	-2.914132	-3.372992	-3.468831
123	1	0	-3.191898	-4.099784	-4.238586
124	1	0	-2.284506	-2.604482	-3.916789
125	1	0	-2.345884	-3.894129	-2.694297
126	6	0	-0.786800	1.693931	-1.299547
127	1	0	-0.691454	2.768975	-1.356063
128	1	0	-1.025600	1.076500	-2.153533
129	7	0	-0.082228	1.092837	-0.319062
130	8	0	-0.020815	-0.153516	-0.263860
131	8	0	0.480099	1.807097	0.581722
132	1	0	-4.042753	4.508382	1.763607
133	6	0	-2.013628	5.147843	1.411302
134	6	0	-2.333951	6.489449	1.612216

135	6	0	-0.721821	4.799025	1.010733
136	6	0	-1.372215	7.480014	1.418890
137	1	0	-3.341216	6.762800	1.919194
138	6	0	0.234075	5.790158	0.810784
139	1	0	-0.459441	3.752151	0.860080
140	6	0	-0.087203	7.131570	1.013682
141	1	0	-1.631067	8.522559	1.576608
142	1	0	1.233391	5.507949	0.493455
143	1	0	0.661080	7.902109	0.855917

G1-II-*si*-TS2

Zero-point correction= 1.23138 (a.u.)
Thermal correction to Gibbs Free Energy= 1.15811 (a.u.)
Sum of electronic and zero-point Energies= -3123.25718 (a.u.)
Sum of electronic and thermal Free Energies= -3123.33045 (a.u.)

Standard orientation:

Center Number	Atomic Number	Atomic Type	Coordinates (Angstroms) X	Y	Z
1	6	0	-1.279315	-1.674414	-3.500785
2	6	0	-1.613735	-0.303609	-4.074948
3	6	0	-1.358050	0.784665	-3.040930
4	7	0	0.046005	0.755338	-2.571860
5	6	0	0.160387	-1.683219	-3.008576
6	6	0	0.411539	-0.564656	-1.986579
7	1	0	-1.423745	-2.457537	-4.253930
8	1	0	-1.944937	-1.884657	-2.657054
9	1	0	-2.671097	-0.250695	-4.354143
10	1	0	-1.013242	-0.106809	-4.972234
11	1	0	-1.536977	1.767989	-3.480907
12	1	0	-2.030373	0.635498	-2.187049
13	1	0	0.395992	-2.633263	-2.516975
14	1	0	0.865921	-1.546855	-3.836624
15	1	0	-0.257658	-0.708400	-1.130480
16	6	0	0.600179	1.920820	-2.159494
17	7	0	-0.204784	2.930802	-1.811823
18	7	0	1.930517	2.055386	-2.170368
19	6	0	2.746439	2.807990	-1.206022
20	1	0	-1.093541	2.631700	-1.420904
21	6	0	1.876734	-0.759214	-1.567042
22	8	0	2.795190	-0.529992	-2.355573
23	7	0	1.974891	-1.319630	-0.346044

24	1	0	1.087238	-1.371336	0.178426
25	6	0	3.115718	-1.988224	0.303702
26	6	0	2.314795	2.532885	0.235719
27	6	0	3.258571	3.202933	1.232777
28	6	0	4.693808	2.724497	1.014848
29	6	0	4.198015	2.379395	-1.435750
30	1	0	2.338941	1.445799	0.386877
31	1	0	1.278700	2.853257	0.395299
32	1	0	3.211157	4.294813	1.111228
33	1	0	2.931272	2.980523	2.252950
34	1	0	2.667494	3.884725	-1.415570
35	1	0	5.371884	3.197909	1.732515
36	1	0	4.493615	2.636487	-2.459583
37	1	0	4.240692	1.285571	-1.354036
38	6	0	5.144836	3.014626	-0.417959
39	1	0	4.733087	1.641191	1.203090
40	1	0	6.160758	2.642358	-0.586479
41	1	0	5.176557	4.101645	-0.574569
42	1	0	2.404449	1.262355	-2.606587
43	6	0	3.549555	-1.168400	1.543098
44	6	0	4.838085	-1.275716	2.082526
45	6	0	2.624468	-0.361478	2.214128
46	6	0	5.200364	-0.574618	3.229579
47	1	0	5.580667	-1.896910	1.592914
48	6	0	2.987821	0.343247	3.360878
49	1	0	1.606455	-0.270902	1.848303
50	6	0	4.278565	0.246826	3.871340
51	1	0	6.210299	-0.670441	3.616251
52	1	0	2.246785	0.963676	3.856313
53	1	0	4.559835	0.800453	4.761631
54	6	0	0.091044	4.362052	-1.803130
55	6	0	0.684326	4.832328	-3.133856
56	6	0	-1.210713	5.108508	-1.507851
57	1	0	0.798905	4.592261	-0.992343
58	6	0	0.940268	6.340396	-3.093895
59	1	0	-0.035937	4.594079	-3.928404
60	1	0	1.606123	4.288550	-3.362191
61	6	0	-0.971753	6.618596	-1.470912
62	1	0	-1.938222	4.863141	-2.293757
63	1	0	-1.626580	4.757541	-0.555906
64	6	0	-0.341757	7.111440	-2.776020
65	1	0	1.359075	6.669900	-4.049455
66	1	0	1.696133	6.557609	-2.326254
67	1	0	-1.915761	7.137681	-1.279335

68	1	0	-0.302961	6.855454	-0.632665
69	1	0	-0.135960	8.184762	-2.714467
70	1	0	-1.058078	6.970063	-3.596255
71	6	0	2.619430	-3.368624	0.813252
72	6	0	3.490714	-4.179392	1.550528
73	6	0	1.342408	-3.854424	0.531232
74	6	0	3.093789	-5.431785	2.002199
75	1	0	4.495327	-3.834232	1.772173
76	6	0	0.941947	-5.109625	0.991921
77	1	0	0.626130	-3.274607	-0.039407
78	6	0	1.810942	-5.903601	1.728181
79	1	0	3.789812	-6.040707	2.571117
80	1	0	-0.062236	-5.453878	0.763227
81	1	0	1.496902	-6.879667	2.084637
82	6	0	-3.619519	-0.077347	0.365617
83	6	0	-2.581065	0.970903	0.753815
84	8	0	-1.588964	1.296385	0.122251
85	6	0	-4.209290	0.962319	2.352548
86	6	0	-4.656720	-0.003785	1.428712
87	7	0	-3.545488	-0.537592	-0.839031
88	6	0	-4.345449	-1.606849	-1.216545
89	8	0	-4.849083	-2.429631	-0.473027
90	8	0	-4.430451	-1.637544	-2.553171
91	6	0	-5.091097	-2.746005	-3.225859
92	6	0	-6.572004	-2.784277	-2.859228
93	1	0	-7.086455	-3.503760	-3.503213
94	1	0	-6.715116	-3.079409	-1.819588
95	1	0	-7.025804	-1.800784	-3.016195
96	6	0	-4.380936	-4.057292	-2.904173
97	1	0	-4.503444	-4.331041	-1.856371
98	1	0	-4.795098	-4.854563	-3.529099
99	1	0	-3.312381	-3.968777	-3.122947
100	6	0	-4.916333	-2.397072	-4.699044
101	1	0	-5.391071	-3.157964	-5.324523
102	1	0	-5.373151	-1.428150	-4.920981
103	1	0	-3.853535	-2.350545	-4.955528
104	7	0	-2.988055	1.514780	1.942680
105	6	0	-2.218434	2.488005	2.693353
106	1	0	-1.465909	2.871789	1.998584
107	6	0	-5.877570	-0.632961	1.643608
108	1	0	-6.230630	-1.385434	0.951375
109	6	0	-6.622662	-0.297265	2.776761
110	1	0	-7.574291	-0.787549	2.952037
111	6	0	-6.149708	0.641857	3.689647

112	1	0	-6.733939	0.877028	4.573640
113	6	0	-4.927676	1.288518	3.490661
114	1	0	-4.549240	2.010918	4.205932
115	1	0	-2.871997	3.318323	2.982988
116	6	0	-1.557838	1.872400	3.908904
117	6	0	-1.718074	2.425065	5.177716
118	6	0	-0.782843	0.720569	3.749451
119	6	0	-1.113664	1.830242	6.285079
120	1	0	-2.321080	3.321894	5.303687
121	6	0	-0.193494	0.118627	4.857289
122	1	0	-0.652026	0.279940	2.761024
123	6	0	-0.355519	0.674180	6.127182
124	1	0	-1.245441	2.266040	7.270682
125	1	0	0.396902	-0.783274	4.721132
126	1	0	0.105578	0.203498	6.989866
127	6	0	-2.478294	-1.770029	1.691807
128	1	0	-3.307782	-2.411373	1.435804
129	1	0	-2.315127	-1.358891	2.677269
130	7	0	-1.421036	-1.833682	0.878303
131	8	0	-0.336633	-1.230731	1.200558
132	8	0	-1.509191	-2.415878	-0.232544
133	6	0	4.252564	-2.194719	-0.714347
134	6	0	4.217345	-3.297889	-1.568025
135	6	0	5.274369	-1.256538	-0.882110
136	6	0	5.179683	-3.462890	-2.561080
137	1	0	3.424851	-4.032821	-1.464820
138	6	0	6.240866	-1.421967	-1.867250
139	1	0	5.311754	-0.376474	-0.247916
140	6	0	6.197969	-2.527784	-2.712448
141	1	0	5.130614	-4.327199	-3.216002
142	1	0	7.023783	-0.677873	-1.979537
143	1	0	6.951656	-2.657663	-3.482832

G1-I-*re*-IM3

Zero-point correction= 1.23323 (a.u.)

Thermal correction to Gibbs Free Energy= 1.16208 (a.u.)

Sum of electronic and zero-point Energies= -3123.29213 (a.u.)

Sum of electronic and thermal Free Energies= -3123.36328 (a.u.)

Standard orientation:

Center Number	Atomic Number	Atomic Type	Coordinates (Angstroms) X	Y	Z

1	6	0	0.033929	0.017682	4.763917
2	6	0	-1.352392	-0.544817	4.461170
3	6	0	-1.637738	-0.495162	2.964328
4	7	0	-0.601769	-1.252417	2.239422
5	6	0	1.095321	-0.700136	3.933635
6	6	0	0.735513	-0.669769	2.443142
7	1	0	0.268702	-0.067847	5.829329
8	1	0	0.048775	1.087961	4.516694
9	1	0	-2.127923	0.022673	4.984581
10	1	0	-1.416288	-1.585268	4.801694
11	1	0	-2.618526	-0.905717	2.724131
12	1	0	-1.643899	0.539366	2.598139
13	1	0	2.074445	-0.226289	4.062895
14	1	0	1.195481	-1.747789	4.236115
15	1	0	0.638143	0.380393	2.135720
16	6	0	-0.940611	-2.130786	1.261294
17	7	0	-2.107807	-1.988563	0.647714
18	7	0	-0.090638	-3.140345	0.992926
19	6	0	0.068120	-3.811900	-0.301537
20	1	0	-2.454816	-0.986689	0.579128
21	6	0	1.943744	-1.263155	1.674711
22	8	0	2.541089	-2.237481	2.116416
23	7	0	2.321801	-0.529330	0.595978
24	1	0	1.646745	0.185599	0.332629
25	6	0	0.314568	-2.811868	-1.429892
26	6	0	0.525795	-3.536391	-2.758348
27	6	0	1.663419	-4.555383	-2.659915
28	6	0	1.201716	-4.828756	-0.177155
29	1	0	1.200636	-2.221242	-1.171862
30	1	0	-0.519832	-2.107239	-1.505193
31	1	0	-0.403286	-4.053282	-3.038615
32	1	0	0.727037	-2.803062	-3.545480
33	1	0	-0.841775	-4.379961	-0.529963
34	1	0	1.770430	-5.100137	-3.603953
35	1	0	0.953520	-5.553880	0.607110
36	1	0	2.116176	-4.312247	0.141718
37	6	0	1.423420	-5.542707	-1.513362
38	1	0	2.610233	-4.023233	-2.496129
39	1	0	2.260307	-6.243434	-1.424196
40	1	0	0.534282	-6.145459	-1.744213
41	1	0	0.665151	-3.267714	1.658012
42	6	0	4.600915	-1.483638	0.140453
43	6	0	3.697731	-0.240554	0.133466
44	6	0	3.625036	0.209782	-1.336910

45	6	0	4.211067	0.866901	1.077555
46	6	0	5.926497	-1.446252	0.566744
47	6	0	6.738701	-2.576421	0.447901
48	6	0	6.237498	-3.749929	-0.099328
49	6	0	4.915746	-3.785802	-0.546298
50	6	0	4.116384	-2.658656	-0.435327
51	1	0	6.339704	-0.539841	0.996123
52	1	0	7.768048	-2.529883	0.789551
53	1	0	6.868668	-4.628933	-0.183459
54	1	0	4.508997	-4.693472	-0.984832
55	1	0	3.094612	-2.675515	-0.796517
56	6	0	4.438648	0.525005	2.421024
57	6	0	4.809249	1.488113	3.349215
58	6	0	4.353517	2.199686	0.695448
59	6	0	4.731859	3.167261	1.631192
60	6	0	4.961187	2.818556	2.955192
61	1	0	4.985365	1.200014	4.381252
62	1	0	4.327750	-0.510866	2.732069
63	1	0	5.258506	3.572434	3.677551
64	1	0	4.841321	4.197661	1.307319
65	1	0	4.168919	2.502344	-0.330948
66	6	0	2.454471	0.140455	-2.092934
67	6	0	2.465510	0.484600	-3.447460
68	6	0	3.637278	0.913671	-4.056162
69	6	0	4.803953	0.636193	-1.962909
70	6	0	4.809629	0.996450	-3.303013
71	1	0	1.511495	-0.172318	-1.655480
72	1	0	1.539418	0.416221	-4.009866
73	1	0	3.643790	1.180524	-5.108679
74	1	0	5.733933	1.329712	-3.764661
75	1	0	5.726854	0.678701	-1.390037
76	6	0	-2.896656	-3.042649	-0.000434
77	6	0	-3.016848	-4.301146	0.868575
78	6	0	-4.300004	-2.505729	-0.291436
79	1	0	-2.438272	-3.302446	-0.967131
80	6	0	-3.835849	-5.371437	0.143331
81	1	0	-3.518695	-4.014711	1.802644
82	1	0	-2.035741	-4.700067	1.142021
83	6	0	-5.133595	-3.556341	-1.025585
84	1	0	-4.771423	-2.237089	0.660299
85	1	0	-4.225405	-1.588194	-0.882207
86	6	0	-5.227887	-4.854075	-0.221390
87	1	0	-3.908617	-6.268609	0.766666
88	1	0	-3.305869	-5.666699	-0.773764

89	1	0	-6.134953	-3.157906	-1.221432
90	1	0	-4.677969	-3.763769	-2.003866
91	1	0	-5.783312	-5.614901	-0.780214
92	1	0	-5.789476	-4.660571	0.702402
93	8	0	-5.089437	1.190849	-0.373022
94	8	0	-0.191969	0.588221	-0.144767
95	7	0	-0.516604	2.805542	0.371260
96	7	0	-2.998448	0.658796	0.420449
97	8	0	-4.847099	-0.048153	1.522438
98	6	0	-6.535000	1.171855	-0.286933
99	6	0	-2.407077	1.626276	-0.457331
100	6	0	-0.904386	1.581159	-0.081984
101	6	0	-2.723637	3.102343	-0.200463
102	6	0	-1.572195	3.730479	0.287476
103	6	0	-3.860617	3.864230	-0.412365
104	1	0	-4.750525	3.402295	-0.816887
105	6	0	-4.319145	0.575982	0.600838
106	6	0	-3.842455	5.227196	-0.094347
107	1	0	-4.735389	5.823279	-0.252561
108	6	0	0.828206	3.148658	0.788933
109	1	0	1.344871	2.212747	1.028979
110	6	0	-1.525516	5.078666	0.602785
111	1	0	-0.613885	5.544931	0.961292
112	6	0	-2.692430	5.822698	0.412161
113	1	0	-2.690932	6.881661	0.649939
114	6	0	-6.984667	1.963373	-1.513817
115	6	0	-7.062319	-0.258359	-0.392115
116	6	0	-7.010003	1.867340	0.986539
117	1	0	-6.748290	1.283928	1.868456
118	1	0	-8.095921	2.002635	0.951516
119	1	0	-6.544771	2.855621	1.067731
120	1	0	-8.074182	1.927437	-1.603742
121	1	0	-6.550506	1.539464	-2.424536
122	1	0	-6.692249	3.015577	-1.444654
123	1	0	-8.155509	-0.244136	-0.451849
124	1	0	-6.759389	-0.848498	0.472115
125	1	0	-6.678082	-0.728608	-1.303684
126	1	0	0.774941	3.734543	1.712428
127	6	0	1.599476	3.893677	-0.278573
128	6	0	2.335687	5.032181	0.043292
129	6	0	1.633508	3.398252	-1.583858
130	6	0	3.117134	5.662656	-0.923553
131	1	0	2.307591	5.418238	1.059742
132	6	0	2.416050	4.024284	-2.548621

133	1	0	1.060866	2.510216	-1.843236
134	6	0	3.162631	5.155510	-2.219129
135	1	0	3.688236	6.548797	-0.664117
136	1	0	2.449175	3.621337	-3.555844
137	1	0	3.774236	5.641189	-2.972915
138	6	0	-2.553446	1.377370	-1.989165
139	1	0	-2.102468	2.182368	-2.568798
140	1	0	-3.610057	1.239161	-2.215455
141	7	0	-1.847431	0.133655	-2.406070
142	8	0	-2.412020	-0.928578	-2.218088
143	8	0	-0.739904	0.248488	-2.904826

G1-I-*si*-IM3

Zero-point correction= 1.23397 (a.u.)

Thermal correction to Gibbs Free Energy= 1.16315 (a.u.)

Sum of electronic and zero-point Energies= -3123.27908 (a.u.)

Sum of electronic and thermal Free Energies= -3123.34990 (a.u.)

Standard orientation:

Center Number	Atomic Number	Atomic Type	Coordinates (Angstroms) X	Y	Z
1	6	0	-1.921585	3.236961	-2.823498
2	6	0	-0.971856	3.172045	-4.025424
3	6	0	0.167673	4.191941	-3.934419
4	6	0	-0.372012	5.607480	-3.725037
5	6	0	-2.419995	4.668798	-2.588909
6	1	0	-1.565906	3.370111	-4.924945
7	1	0	-0.579609	2.152251	-4.113982
8	1	0	0.837840	3.929488	-3.105543
9	1	0	0.771974	4.140554	-4.845456
10	1	0	-2.773602	2.581192	-3.034082
11	1	0	0.454168	6.320053	-3.633846
12	1	0	-3.059782	4.933909	-3.440042
13	6	0	-1.267188	5.670909	-2.486441
14	1	0	-0.955711	5.907612	-4.605885
15	1	0	-1.668231	6.680012	-2.350010
16	1	0	-0.665087	5.447039	-1.594550
17	6	0	0.223544	4.181810	2.830940
18	6	0	0.535872	4.613595	1.395361
19	6	0	0.623629	3.418394	0.443923
20	7	0	-0.600091	2.606802	0.555020
21	6	0	-0.998233	3.258768	2.895602

22	6	0	-0.784798	2.102002	1.924735
23	1	0	0.071470	5.061163	3.463669
24	1	0	1.091369	3.645271	3.237253
25	1	0	1.486798	5.155428	1.359828
26	1	0	-0.243988	5.296870	1.034410
27	1	0	0.755337	3.747540	-0.586699
28	1	0	1.463317	2.765104	0.695950
29	1	0	-1.139015	2.859518	3.903424
30	1	0	-1.914904	3.804179	2.638433
31	1	0	0.180927	1.633362	2.157640
32	6	0	-1.567280	2.643477	-0.385466
33	7	0	-1.183120	2.722157	-1.663071
34	7	0	-2.855370	2.608943	-0.017225
35	6	0	-3.997285	2.083773	-0.785871
36	1	0	-0.236913	2.380315	-1.838470
37	6	0	-1.834911	0.974070	2.062461
38	8	0	-2.865614	1.186924	2.690769
39	7	0	-1.434431	-0.162839	1.457031
40	1	0	-0.514362	-0.085324	1.022297
41	6	0	-3.721457	0.688097	-1.342589
42	6	0	-4.954366	0.134767	-2.056120
43	6	0	-6.156143	0.098456	-1.111954
44	6	0	-5.206745	2.076974	0.152857
45	1	0	-3.452463	0.029833	-0.508708
46	1	0	-2.853760	0.695371	-2.010972
47	1	0	-5.192272	0.763331	-2.926682
48	1	0	-4.727914	-0.866342	-2.434090
49	1	0	-4.218832	2.773975	-1.611528
50	1	0	-7.042140	-0.279880	-1.631970
51	1	0	-5.408879	3.099610	0.492795
52	1	0	-4.950951	1.486585	1.043228
53	6	0	-6.438916	1.488117	-0.537113
54	1	0	-5.943364	-0.603440	-0.293486
55	1	0	-7.269846	1.450842	0.174290
56	1	0	-6.749383	2.157251	-1.351086
57	1	0	-3.051449	4.708523	-1.694201
58	1	0	-3.027753	2.701425	0.979024
59	6	0	-3.024067	-1.794508	2.543028
60	6	0	-1.826948	-1.596665	1.605824
61	6	0	-2.170832	-2.134708	0.198945
62	6	0	-0.552838	-2.266182	2.158539
63	6	0	-2.997615	-2.698074	3.602407
64	6	0	-4.147592	-2.944210	4.358024
65	6	0	-5.337342	-2.295365	4.059583

66	6	0	-5.379151	-1.401066	2.987944
67	6	0	-4.236859	-1.164883	2.240433
68	1	0	-2.084200	-3.230806	3.840827
69	1	0	-4.102688	-3.653527	5.178606
70	1	0	-6.230119	-2.489108	4.646221
71	1	0	-6.304034	-0.890953	2.733981
72	1	0	-4.273946	-0.483136	1.398506
73	6	0	0.010931	-1.743692	3.332731
74	6	0	1.178429	-2.276705	3.859181
75	6	0	0.083320	-3.324638	1.519056
76	6	0	1.264940	-3.853879	2.044285
77	6	0	1.813395	-3.338869	3.209572
78	1	0	1.597711	-1.864000	4.772223
79	1	0	-0.481693	-0.915897	3.838882
80	1	0	2.737234	-3.747443	3.607719
81	1	0	1.765615	-4.653503	1.507373
82	1	0	-0.287978	-3.708127	0.575085
83	6	0	-1.357796	-1.773626	-0.880269
84	6	0	-1.640815	-2.226321	-2.163987
85	6	0	-2.739066	-3.052962	-2.393496
86	6	0	-3.245791	-2.997554	-0.030930
87	6	0	-3.532580	-3.447542	-1.319479
88	1	0	-0.460486	-1.179055	-0.734175
89	1	0	-0.981957	-1.937483	-2.977250
90	1	0	-2.964829	-3.401127	-3.397761
91	1	0	-4.379172	-4.109122	-1.477577
92	1	0	-3.873637	-3.321618	0.791648
93	6	0	2.758246	-0.219632	-2.207805
94	1	0	2.774582	-0.915883	-3.047042
95	1	0	3.589540	0.483659	-2.208549
96	7	0	1.534436	0.599940	-2.315494
97	8	0	1.613690	1.786972	-1.988911
98	8	0	0.505030	0.077868	-2.689732
99	8	0	0.921454	-4.034005	-1.615993
100	8	0	1.319895	0.358485	0.520045
101	7	0	3.529491	0.024778	1.028189
102	7	0	1.757224	-2.131183	-0.870664
103	8	0	2.616563	-3.080873	-2.814374
104	1	0	4.120944	0.314104	2.997555
105	6	0	0.872294	-5.194267	-2.458298
106	6	0	2.727832	-1.098788	-0.897949
107	6	0	2.403723	-0.161690	0.285683
108	6	0	4.187270	-1.450021	-0.606417
109	6	0	4.603366	-0.732480	0.521088

110	6	0	5.086352	-2.279831	-1.255953
111	1	0	4.760980	-2.840015	-2.125045
112	6	0	1.851855	-3.038036	-1.826576
113	6	0	6.391925	-2.378968	-0.757853
114	1	0	7.104016	-3.031712	-1.252379
115	6	0	3.574696	0.847703	2.212613
116	1	0	2.537963	0.952992	2.551268
117	6	0	5.893522	-0.801490	1.019477
118	1	0	6.201570	-0.218008	1.881086
119	6	0	6.788597	-1.647019	0.357096
120	1	0	7.807801	-1.727796	0.721726
121	6	0	4.188964	2.213659	1.972319
122	6	0	4.797574	2.893127	3.029135
123	6	0	4.112962	2.830043	0.721674
124	6	0	5.311984	4.173365	2.843876
125	1	0	4.870197	2.414897	4.003039
126	6	0	4.629766	4.110262	0.534993
127	1	0	3.641663	2.312147	-0.111105
128	6	0	5.228859	4.785791	1.595563
129	1	0	5.786599	4.688854	3.672961
130	1	0	4.566169	4.576835	-0.443368
131	1	0	5.636272	5.781082	1.449089
132	6	0	0.481084	-4.825842	-3.888962
133	1	0	1.238933	-4.184041	-4.338530
134	1	0	0.369753	-5.733177	-4.492600
135	1	0	-0.478556	-4.299456	-3.884490
136	6	0	-0.231544	-6.031297	-1.816283
137	1	0	0.039741	-6.292411	-0.788423
138	1	0	-1.166177	-5.462018	-1.789724
139	1	0	-0.395595	-6.955479	-2.379167
140	6	0	2.195915	-5.958195	-2.415840
141	1	0	2.086122	-6.926080	-2.916480
142	1	0	2.987913	-5.389091	-2.902253
143	1	0	2.482068	-6.142678	-1.374876

G1-II-*re*-IM3

Zero-point correction= 1.23291 (a.u.)

Thermal correction to Gibbs Free Energy= 1.16094 (a.u.)

Sum of electronic and zero-point Energies= -3123.28825 (a.u.)

Sum of electronic and thermal Free Energies= -3123.36021 (a.u.)

Standard orientation:

Center Atomic Atomic Coordinates (Angstroms)

Number	Number	Type	X	Y	Z
1	6	0	-0.219326	-0.196558	4.343826
2	6	0	0.974424	-0.215051	3.384098
3	6	0	0.596357	-0.750574	2.005252
4	7	0	-0.549505	0.009170	1.453567
5	6	0	-1.438884	0.499165	3.724432
6	6	0	-1.702848	-0.128206	2.352975
7	1	0	0.051897	0.288838	5.286053
8	1	0	-0.497424	-1.230594	4.588306
9	1	0	1.783055	-0.839265	3.775244
10	1	0	1.387621	0.793951	3.265316
11	1	0	1.441516	-0.690167	1.329415
12	1	0	0.290613	-1.800474	2.059048
13	1	0	-2.326290	0.379344	4.352824
14	1	0	-1.256788	1.576544	3.634565
15	1	0	-1.770269	-1.212120	2.519952
16	6	0	-0.314622	1.156912	0.733749
17	7	0	0.681598	1.158183	-0.138867
18	7	0	-1.124474	2.211008	0.966967
19	6	0	-1.427462	3.377790	0.132372
20	6	0	-3.068575	0.245458	1.744756
21	8	0	-3.729489	1.183436	2.168189
22	7	0	-3.491397	-0.636139	0.810192
23	1	0	-2.807774	-1.294324	0.459380
24	6	0	-4.849275	-0.711459	0.254891
25	6	0	-2.080764	2.999683	-1.194174
26	6	0	-2.314444	4.261945	-2.027366
27	6	0	-3.153568	5.291688	-1.264553
28	6	0	-2.347025	4.309369	0.920737
29	1	0	-3.034789	2.505467	-0.970277
30	1	0	-1.463964	2.276907	-1.741847
31	1	0	-1.341265	4.703592	-2.288911
32	1	0	-2.804144	3.998621	-2.970571
33	1	0	-0.504809	3.936552	-0.054329
34	1	0	-3.239214	6.215760	-1.846148
35	1	0	-1.897382	4.535268	1.894723
36	1	0	-3.301881	3.795989	1.102051
37	6	0	-2.574211	5.593375	0.120045
38	1	0	-4.167292	4.893869	-1.140393
39	1	0	-3.239840	6.264089	0.672045
40	1	0	-1.614668	6.117363	0.011806
41	1	0	-1.764831	2.114432	1.744589
42	6	0	-5.892560	-0.846111	1.385899

43	6	0	-5.543477	-1.311436	2.653303
44	6	0	-7.244587	-0.628241	1.104973
45	6	0	-6.516399	-1.522840	3.627209
46	1	0	-4.505103	-1.519884	2.890965
47	6	0	-8.217492	-0.838508	2.076300
48	1	0	-7.541188	-0.285185	0.118200
49	6	0	-7.856993	-1.283271	3.345582
50	1	0	-6.219080	-1.876186	4.609602
51	1	0	-9.260314	-0.655046	1.837125
52	1	0	-8.614575	-1.443889	4.105814
53	6	0	-5.091376	0.522252	-0.638485
54	6	0	-5.663745	1.685890	-0.114277
55	6	0	-4.679510	0.527516	-1.974982
56	6	0	-5.882502	2.796058	-0.926053
57	1	0	-5.939445	1.721143	0.933434
58	6	0	-4.895536	1.638364	-2.787222
59	1	0	-4.206341	-0.351720	-2.398551
60	6	0	-5.514299	2.772202	-2.269308
61	1	0	-6.344885	3.682793	-0.502364
62	1	0	-4.581554	1.610815	-3.826242
63	1	0	-5.697174	3.635979	-2.902127
64	6	0	-4.953132	-2.044133	-0.519897
65	6	0	-5.860049	-2.191809	-1.574483
66	6	0	-4.229411	-3.167885	-0.110300
67	6	0	-5.991892	-3.407180	-2.237821
68	1	0	-6.462761	-1.347176	-1.891011
69	6	0	-4.357954	-4.385015	-0.776082
70	1	0	-3.569413	-3.120796	0.751227
71	6	0	-5.230231	-4.507386	-1.851824
72	1	0	-6.694481	-3.491187	-3.060815
73	1	0	-3.773961	-5.237646	-0.444053
74	1	0	-5.327860	-5.453468	-2.374176
75	6	0	1.296508	2.338930	-0.755867
76	6	0	1.999049	3.223884	0.278604
77	6	0	2.279108	1.909045	-1.847396
78	1	0	0.527264	2.926184	-1.269124
79	6	0	2.613401	4.451124	-0.398289
80	1	0	2.774349	2.617537	0.764702
81	1	0	1.294573	3.527932	1.062254
82	6	0	2.868963	3.138876	-2.537095
83	1	0	3.094611	1.331275	-1.401136
84	1	0	1.777998	1.243944	-2.557387
85	6	0	3.568747	4.043682	-1.521806
86	1	0	3.130536	5.068058	0.343687

87	1	0	1.808732	5.072409	-0.819516
88	1	0	3.571408	2.819217	-3.313397
89	1	0	2.070284	3.702088	-3.040256
90	1	0	3.980716	4.931168	-2.013476
91	1	0	4.416359	3.494679	-1.087257
92	1	0	1.194766	0.240451	-0.364540
93	8	0	0.312508	-1.533562	-1.965809
94	8	0	3.429009	0.277833	1.372179
95	7	0	5.254054	0.319432	-0.013305
96	7	0	2.177387	-1.023901	-0.895816
97	8	0	2.070280	-2.970809	-2.157960
98	6	0	-0.639095	-2.494026	-2.457147
99	6	0	3.559330	-1.237880	-0.590586
100	6	0	4.024160	-0.132726	0.393424
101	6	0	4.612648	-1.109055	-1.692243
102	6	0	5.612714	-0.229921	-1.253767
103	6	0	4.740782	-1.730683	-2.923946
104	1	0	3.969716	-2.414028	-3.260769
105	6	0	1.600708	-1.925693	-1.686548
106	6	0	5.870140	-1.452833	-3.704858
107	1	0	5.978228	-1.925724	-4.675515
108	6	0	6.061047	1.225524	0.779185
109	1	0	5.367909	1.733330	1.457090
110	6	0	6.747758	0.039126	-2.001526
111	1	0	7.522815	0.705110	-1.636941
112	6	0	6.859617	-0.589801	-3.245074
113	1	0	7.735806	-0.396974	-3.856441
114	6	0	-0.271736	-3.007442	-3.849330
115	1	0	0.583883	-3.679228	-3.808374
116	1	0	-1.128626	-3.532955	-4.284789
117	1	0	-0.023324	-2.162318	-4.499248
118	6	0	-1.928802	-1.682114	-2.538262
119	1	0	-2.117826	-1.184155	-1.582107
120	1	0	-1.841726	-0.903743	-3.303825
121	1	0	-2.782471	-2.325753	-2.778840
122	6	0	-0.785908	-3.634901	-1.453508
123	1	0	-1.563727	-4.330735	-1.783284
124	1	0	0.159160	-4.169813	-1.347491
125	1	0	-1.070736	-3.235779	-0.472593
126	6	0	3.873734	-2.614585	0.088675
127	1	0	4.889722	-2.638664	0.485005
128	1	0	3.670776	-3.400636	-0.634329
129	7	0	2.960616	-2.852333	1.238950
130	8	0	1.905774	-3.416344	1.013586

131	8	0	3.307738	-2.449591	2.338729
132	1	0	6.511863	1.977376	0.123601
133	6	0	7.124834	0.487686	1.564907
134	6	0	8.467710	0.844803	1.472058
135	6	0	6.750920	-0.571562	2.398445
136	6	0	9.433446	0.154942	2.204176
137	1	0	8.761295	1.667232	0.823954
138	6	0	7.714119	-1.261355	3.125783
139	1	0	5.701465	-0.851471	2.478325
140	6	0	9.057881	-0.899282	3.029958
141	1	0	10.478216	0.438553	2.122868
142	1	0	7.416096	-2.082749	3.769869
143	1	0	9.808957	-1.440249	3.597027

G1-II-*si*-IM3

Zero-point correction= 1.23251 (a.u.)
Thermal correction to Gibbs Free Energy= 1.16088 (a.u.)
Sum of electronic and zero-point Energies= -3123.30072 (a.u.)
Sum of electronic and thermal Free Energies= -3123.37235 (a.u.)

Standard orientation:

Center Number	Atomic Number	Atomic Type	Coordinates (Angstroms) X	Y	Z
1	6	0	-0.197999	0.199748	-4.908276
2	6	0	-1.317539	1.204207	-4.661522
3	6	0	-1.677087	1.207823	-3.183722
4	7	0	-0.501133	1.607307	-2.379840
5	6	0	0.988402	0.506267	-3.999116
6	6	0	0.577933	0.614085	-2.516285
7	1	0	0.124408	0.207493	-5.954382
8	1	0	-0.566645	-0.808179	-4.682157
9	1	0	-2.211197	0.942574	-5.236916
10	1	0	-1.002674	2.209993	-4.965799
11	1	0	-2.496766	1.892966	-2.972867
12	1	0	-2.012840	0.210149	-2.879773
13	1	0	1.734475	-0.290691	-4.084850
14	1	0	1.463067	1.453100	-4.284782
15	1	0	0.170263	-0.352045	-2.197077
16	6	0	-0.749787	2.317139	-1.217698
17	7	0	-1.913497	2.138356	-0.627485
18	7	0	0.195349	3.189498	-0.849433
19	6	0	0.364445	3.871797	0.429115

20	1	0	-2.361653	1.157249	-0.760178
21	6	0	1.888529	0.857546	-1.756824
22	8	0	2.504144	1.918474	-1.784029
23	7	0	2.393806	-0.299354	-1.264087
24	1	0	1.716464	-1.032418	-1.076619
25	6	0	3.729411	-0.474048	-0.679590
26	6	0	1.783333	3.655907	0.960186
27	6	0	1.971566	4.390546	2.290430
28	6	0	1.639424	5.879487	2.178036
29	6	0	0.067070	5.369440	0.284530
30	1	0	2.502368	4.029988	0.218142
31	1	0	1.973527	2.582543	1.071027
32	1	0	1.322401	3.927812	3.048207
33	1	0	2.999364	4.258989	2.637856
34	1	0	-0.332532	3.428148	1.146886
35	1	0	1.743284	6.365403	3.154014
36	1	0	-0.937397	5.519908	-0.124889
37	1	0	0.770511	5.784676	-0.450188
38	6	0	0.231631	6.090692	1.621946
39	1	0	2.363836	6.357614	1.504881
40	1	0	0.018129	7.157623	1.502579
41	1	0	-0.505394	5.697604	2.336810
42	1	0	0.997953	3.211005	-1.474300
43	6	0	4.857801	-0.204526	-1.695992
44	6	0	4.625171	0.106261	-3.032600
45	6	0	6.184706	-0.359689	-1.273725
46	6	0	5.686137	0.271096	-3.923041
47	1	0	3.615255	0.228521	-3.397686
48	6	0	7.243330	-0.191111	-2.156598
49	1	0	6.391273	-0.619486	-0.239179
50	6	0	6.998339	0.126737	-3.491568
51	1	0	5.475367	0.515960	-4.959533
52	1	0	8.261599	-0.315562	-1.801490
53	1	0	7.822275	0.255887	-4.186168
54	6	0	3.850954	0.398828	0.582234
55	6	0	4.700610	1.500067	0.676563
56	6	0	3.064547	0.063183	1.688803
57	6	0	4.815217	2.203312	1.875727
58	1	0	5.283702	1.810394	-0.183005
59	6	0	3.180214	0.759115	2.887049
60	1	0	2.378226	-0.775643	1.615263
61	6	0	4.073930	1.823521	2.990086
62	1	0	5.491873	3.050672	1.934114
63	1	0	2.586821	0.453456	3.745075

64	1	0	4.180934	2.359025	3.928656
65	6	0	3.854723	-1.977839	-0.336182
66	6	0	4.576847	-2.423414	0.774548
67	6	0	3.324382	-2.930133	-1.212926
68	6	0	4.760028	-3.784460	1.003761
69	1	0	4.996184	-1.706820	1.472934
70	6	0	3.492668	-4.290879	-0.973766
71	1	0	2.776447	-2.613621	-2.095487
72	6	0	4.217090	-4.724962	0.132735
73	1	0	5.323135	-4.105636	1.874753
74	1	0	3.063982	-5.010123	-1.664539
75	1	0	4.356975	-5.786037	0.315046
76	6	0	-2.668300	2.983887	0.291255
77	6	0	-4.062163	3.218767	-0.303873
78	6	0	-2.778681	2.300129	1.660011
79	1	0	-2.173940	3.953455	0.402037
80	6	0	-4.946214	4.015684	0.657234
81	1	0	-4.511599	2.242294	-0.528278
82	1	0	-3.957992	3.742067	-1.261029
83	6	0	-3.659878	3.101694	2.618618
84	1	0	-3.218371	1.306076	1.497448
85	1	0	-1.777045	2.142434	2.080768
86	6	0	-5.049085	3.328213	2.019740
87	1	0	-5.940359	4.150112	0.219441
88	1	0	-4.522060	5.020762	0.793918
89	1	0	-3.733901	2.579556	3.578392
90	1	0	-3.188859	4.074768	2.820005
91	1	0	-5.666288	3.922653	2.701665
92	1	0	-5.546512	2.357190	1.896454
93	6	0	-2.472077	-1.313679	0.224084
94	6	0	-1.185395	-0.720501	0.857534
95	8	0	-0.230139	-0.259762	0.255653
96	6	0	-2.503734	-1.399772	2.602321
97	6	0	-3.282007	-1.667902	1.469278
98	7	0	-2.991766	-0.323236	-0.661279
99	6	0	-4.037065	-0.534439	-1.462204
100	8	0	-4.527918	0.327462	-2.193190
101	8	0	-4.544910	-1.819245	-1.433928
102	6	0	-5.540230	-2.234952	-2.397825
103	6	0	-5.027333	-2.036467	-3.822700
104	1	0	-5.689360	-2.550263	-4.527190
105	1	0	-4.981737	-0.978683	-4.079163
106	1	0	-4.023793	-2.464004	-3.914561
107	6	0	-6.857670	-1.505535	-2.152298

108	1	0	-6.740713	-0.437264	-2.330864
109	1	0	-7.631089	-1.900380	-2.819007
110	1	0	-7.186269	-1.659562	-1.118982
111	6	0	-5.700451	-3.725307	-2.110162
112	1	0	-6.465866	-4.157922	-2.760789
113	1	0	-4.759288	-4.255350	-2.289368
114	1	0	-5.999835	-3.890444	-1.070137
115	7	0	-1.276268	-0.838534	2.216126
116	6	0	-0.187802	-0.510369	3.110249
117	1	0	0.445821	0.200766	2.567270
118	1	0	-0.588665	-0.001208	3.993169
119	6	0	0.611161	-1.735701	3.506802
120	6	0	1.028087	-1.922623	4.823706
121	6	0	0.969316	-2.676471	2.535565
122	6	0	1.798255	-3.032722	5.169087
123	1	0	0.746741	-1.197476	5.584231
124	6	0	1.736059	-3.784266	2.879960
125	1	0	0.667498	-2.531936	1.498651
126	6	0	2.152513	-3.964098	4.198434
127	1	0	2.115237	-3.170081	6.198237
128	1	0	2.018075	-4.498842	2.113117
129	1	0	2.750978	-4.829169	4.466297
130	6	0	-4.556028	-2.187292	1.623300
131	1	0	-5.165729	-2.388726	0.749696
132	6	0	-5.032987	-2.437050	2.915679
133	1	0	-6.030898	-2.841299	3.049146
134	6	0	-4.237777	-2.179648	4.028156
135	1	0	-4.618332	-2.389521	5.022781
136	6	0	-2.949689	-1.653798	3.889212
137	1	0	-2.323319	-1.460184	4.754043
138	6	0	-2.030529	-2.685627	-0.395473
139	1	0	-2.897770	-3.175340	-0.830456
140	1	0	-1.525617	-3.301311	0.349200
141	7	0	-1.058357	-2.476531	-1.497757
142	8	0	0.133335	-2.626745	-1.256213
143	8	0	-1.498389	-2.143130	-2.581581

G1-I-*re*-TS3

Zero-point correction= 1.22938 (a.u.)

Thermal correction to Gibbs Free Energy= 1.15933 (a.u.)

Sum of electronic and zero-point Energies= -3123.29167 (a.u.)

Sum of electronic and thermal Free Energies= -3123.36172 (a.u.)

Standard orientation:

Center Number	Atomic Number	Atomic Type	Coordinates (Angstroms) X	Y	Z
1	6	0	0.047342	0.317268	4.691331
2	6	0	-1.322084	-0.316650	4.464140
3	6	0	-1.625321	-0.399544	2.972798
4	7	0	-0.586402	-1.183674	2.292061
5	6	0	1.116181	-0.432340	3.900482
6	6	0	0.731564	-0.543194	2.417965
7	1	0	0.305777	0.329891	5.754802
8	1	0	0.018561	1.363431	4.355443
9	1	0	-2.109118	0.264971	4.954431
10	1	0	-1.342101	-1.327466	4.888989
11	1	0	-2.594988	-0.857597	2.777370
12	1	0	-1.664452	0.612547	2.540931
13	1	0	2.081243	0.081626	3.971578
14	1	0	1.255312	-1.446933	4.286923
15	1	0	0.607863	0.476278	2.027192
16	6	0	-0.950974	-2.053814	1.287112
17	7	0	-2.103718	-1.889258	0.684428
18	7	0	-0.081474	-3.072671	1.061354
19	6	0	0.109012	-3.777475	-0.205124
20	6	0	1.953610	-1.170031	1.700369
21	8	0	2.556094	-2.110017	2.202475
22	7	0	2.345472	-0.494063	0.584941
23	1	0	1.665425	0.190906	0.267346
24	6	0	0.368950	-2.808906	-1.357908
25	6	0	0.582065	-3.567722	-2.665586
26	6	0	1.722364	-4.579903	-2.532121
27	6	0	1.246293	-4.784449	-0.041605
28	1	0	1.259775	-2.221672	-1.109355
29	1	0	-0.456943	-2.096146	-1.449681
30	1	0	-0.345782	-4.094340	-2.932084
31	1	0	0.784816	-2.858679	-3.474708
32	1	0	-0.789945	-4.362105	-0.437626
33	1	0	1.835762	-5.153186	-3.458618
34	1	0	0.991841	-5.490276	0.758130
35	1	0	2.155142	-4.257456	0.275435
36	6	0	1.483050	-5.533677	-1.356526
37	1	0	2.666345	-4.038672	-2.381353
38	1	0	2.323895	-6.226424	-1.243748
39	1	0	0.598980	-6.148442	-1.575253
40	1	0	0.656549	-3.178517	1.748166

41	6	0	4.640900	-1.431313	0.184431
42	6	0	3.725977	-0.198277	0.143825
43	6	0	3.671276	0.227964	-1.335477
44	6	0	4.213161	0.932294	1.074031
45	6	0	5.971827	-1.364105	0.591192
46	6	0	6.794918	-2.488734	0.502057
47	6	0	6.300107	-3.686153	0.001667
48	6	0	4.973346	-3.752388	-0.425503
49	6	0	4.161912	-2.631084	-0.341783
50	1	0	6.379301	-0.438762	0.984450
51	1	0	7.827682	-2.419572	0.829368
52	1	0	6.940162	-4.560531	-0.059937
53	1	0	4.571036	-4.679075	-0.826734
54	1	0	3.135158	-2.671390	-0.685286
55	6	0	4.475074	0.612159	2.416096
56	6	0	4.814818	1.599998	3.331174
57	6	0	4.287367	2.268024	0.680048
58	6	0	4.634108	3.259726	1.601819
59	6	0	4.899633	2.932361	2.925115
60	1	0	5.018100	1.328829	4.362780
61	1	0	4.410984	-0.424167	2.737425
62	1	0	5.172219	3.705073	3.637257
63	1	0	4.691659	4.291542	1.268719
64	1	0	4.072615	2.552247	-0.345642
65	6	0	2.519968	0.112656	-2.115809
66	6	0	2.547051	0.447401	-3.472207
67	6	0	3.715598	0.911933	-4.060584
68	6	0	4.847871	0.686675	-1.942169
69	6	0	4.869209	1.036605	-3.284940
70	1	0	1.579125	-0.230109	-1.696841
71	1	0	1.635126	0.343401	-4.052331
72	1	0	3.734334	1.172683	-5.114461
73	1	0	5.791566	1.396593	-3.730026
74	1	0	5.757288	0.764549	-1.351773
75	6	0	-2.827996	-2.979347	0.020538
76	6	0	-2.969038	-4.221204	0.915568
77	6	0	-4.234144	-2.517375	-0.377092
78	1	0	-2.316339	-3.258905	-0.913741
79	6	0	-3.694063	-5.347602	0.175573
80	1	0	-3.546046	-3.924964	1.802279
81	1	0	-1.997028	-4.571193	1.273326
82	6	0	-4.966426	-3.620415	-1.142645
83	1	0	-4.783744	-2.263275	0.535203
84	1	0	-4.168597	-1.610928	-0.984753

85	6	0	-5.073288	-4.897383	-0.307717
86	1	0	-3.781682	-6.225355	0.824779
87	1	0	-3.089425	-5.655389	-0.690106
88	1	0	-5.964553	-3.272480	-1.431380
89	1	0	-4.424569	-3.836226	-2.074310
90	1	0	-5.558751	-5.695206	-0.880408
91	1	0	-5.708545	-4.697582	0.566040
92	1	0	-2.507726	-0.671282	0.530164
93	8	0	-5.132674	1.065626	-0.351879
94	8	0	-0.195900	0.503024	-0.256041
95	7	0	-0.573830	2.690339	0.354649
96	7	0	-3.023880	0.499144	0.325189
97	8	0	-4.821059	-0.144885	1.546025
98	6	0	-6.580357	1.075680	-0.204444
99	6	0	-2.429856	1.497509	-0.528718
100	6	0	-0.927794	1.475087	-0.147326
101	6	0	-2.782698	2.955712	-0.228096
102	6	0	-1.649157	3.592455	0.288824
103	6	0	-3.924091	3.706836	-0.455449
104	1	0	-4.795247	3.246311	-0.898928
105	6	0	-4.350632	0.451452	0.587646
106	6	0	-3.934899	5.061967	-0.107034
107	1	0	-4.832477	5.647835	-0.275754
108	6	0	0.774208	3.074232	0.732783
109	1	0	1.326958	2.157165	0.960281
110	6	0	-1.631090	4.933956	0.634382
111	1	0	-0.732573	5.406402	1.016795
112	6	0	-2.805986	5.662944	0.439066
113	1	0	-2.826086	6.716152	0.700133
114	6	0	-7.064667	1.855500	-1.424426
115	6	0	-7.127960	-0.348506	-0.264826
116	6	0	-6.984434	1.798345	1.077187
117	1	0	-6.708184	1.220161	1.958241
118	1	0	-8.066906	1.960402	1.079749
119	1	0	-6.492237	2.775383	1.126872
120	1	0	-8.156132	1.817299	-1.479046
121	1	0	-6.659019	1.422361	-2.343626
122	1	0	-6.773175	2.908845	-1.373404
123	1	0	-8.221725	-0.316505	-0.291073
124	1	0	-6.811647	-0.929460	0.600892
125	1	0	-6.779707	-0.842553	-1.177960
126	1	0	0.728067	3.658862	1.656949
127	6	0	1.488629	3.839367	-0.359469
128	6	0	2.175320	5.016548	-0.069292

129	6	0	1.524085	3.322575	-1.656557
130	6	0	2.908347	5.666624	-1.060977
131	1	0	2.148047	5.417822	0.941308
132	6	0	2.259051	3.968060	-2.645483
133	1	0	0.991472	2.402748	-1.890617
134	6	0	2.955232	5.139583	-2.348518
135	1	0	3.441940	6.582624	-0.826727
136	1	0	2.296346	3.547467	-3.645347
137	1	0	3.529712	5.640459	-3.121264
138	6	0	-2.575946	1.274733	-2.059740
139	1	0	-2.166667	2.113280	-2.622003
140	1	0	-3.626798	1.095662	-2.286733
141	7	0	-1.816059	0.072738	-2.513119
142	8	0	-2.330392	-1.016394	-2.343547
143	8	0	-0.721968	0.253559	-3.020288

G1-I-*si*-TS3

Zero-point correction= 1.22752 (a.u.)
Thermal correction to Gibbs Free Energy= 1.15723 (a.u.)
Sum of electronic and zero-point Energies= -3123.27754 (a.u.)
Sum of electronic and thermal Free Energies= -3123.34783 (a.u.)

Standard orientation:

Center Number	Atomic Number	Atomic Type	Coordinates (Angstroms) X	Y	Z
1	6	0	-1.175887	-2.462235	-4.266066
2	6	0	0.331726	-2.644607	-4.163481
3	6	0	0.907123	-1.657309	-3.159439
4	7	0	0.317260	-1.853856	-1.819539
5	6	0	-1.799018	-2.557883	-2.878857
6	6	0	-1.134556	-1.599800	-1.862400
7	1	0	-1.629426	-3.206649	-4.927929
8	1	0	-1.390721	-1.473696	-4.692589
9	1	0	0.814313	-2.481905	-5.132195
10	1	0	0.565736	-3.667590	-3.843489
11	1	0	1.984089	-1.752762	-3.057181
12	1	0	0.709607	-0.622818	-3.472867
13	1	0	-2.863976	-2.311410	-2.935163
14	1	0	-1.709570	-3.574956	-2.480477
15	1	0	-1.242276	-0.566535	-2.220362
16	6	0	1.161319	-1.770057	-0.750448
17	7	0	2.438542	-1.408413	-0.963696

18	7	0	0.713500	-2.080402	0.476410
19	6	0	1.342179	-1.567212	1.691194
20	6	0	-1.988112	-1.766070	-0.597020
21	8	0	-1.967118	-2.773561	0.102936
22	7	0	-2.921553	-0.792386	-0.459720
23	1	0	-2.780625	0.072082	-0.969110
24	6	0	-4.014990	-0.794129	0.529625
25	6	0	0.639712	-0.288308	2.156964
26	6	0	1.281673	0.244212	3.438651
27	6	0	1.321027	-0.821452	4.536603
28	6	0	1.338792	-2.635422	2.781201
29	1	0	-0.427484	-0.499006	2.317199
30	1	0	0.723056	0.455754	1.356444
31	1	0	2.305058	0.567371	3.203245
32	1	0	0.739877	1.131955	3.784979
33	1	0	2.375189	-1.301382	1.450635
34	1	0	1.831299	-0.432634	5.424585
35	1	0	1.854491	-3.532102	2.419051
36	1	0	0.297767	-2.919666	2.996552
37	6	0	2.002477	-2.102749	4.051810
38	1	0	0.293215	-1.063403	4.835779
39	1	0	1.982607	-2.866827	4.836081
40	1	0	3.058849	-1.894394	3.836505
41	1	0	-0.195854	-2.523999	0.575806
42	6	0	-4.847532	-2.091667	0.468420
43	6	0	-4.928338	-2.846346	-0.700441
44	6	0	-5.661779	-2.447171	1.547427
45	6	0	-5.772968	-3.949109	-0.781667
46	1	0	-4.331478	-2.569918	-1.561003
47	6	0	-6.508369	-3.548602	1.468647
48	1	0	-5.636031	-1.863289	2.462005
49	6	0	-6.563942	-4.309626	0.304732
50	1	0	-5.809541	-4.526931	-1.699860
51	1	0	-7.125797	-3.809352	2.322484
52	1	0	-7.220993	-5.171193	0.243775
53	6	0	-3.399572	-0.542401	1.919422
54	6	0	-3.000188	-1.600667	2.743136
55	6	0	-3.155630	0.764311	2.349488
56	6	0	-2.427748	-1.349668	3.988307
57	1	0	-3.142859	-2.621586	2.407764
58	6	0	-2.571420	1.016913	3.588143
59	1	0	-3.447640	1.595856	1.714877
60	6	0	-2.220496	-0.041635	4.420757
61	1	0	-2.140600	-2.184485	4.620878

62	1	0	-2.403054	2.042063	3.905719
63	1	0	-1.780193	0.150447	5.394533
64	6	0	-5.014700	0.304469	0.106085
65	6	0	-5.887236	0.865789	1.045380
66	6	0	-5.173860	0.651727	-1.237694
67	6	0	-6.872339	1.765093	0.654132
68	1	0	-5.797017	0.597549	2.093348
69	6	0	-6.164192	1.549659	-1.629489
70	1	0	-4.546230	0.205753	-2.003398
71	6	0	-7.015736	2.113039	-0.686772
72	1	0	-7.535798	2.188440	1.401768
73	1	0	-6.264574	1.804385	-2.679910
74	1	0	-7.787629	2.811875	-0.993421
75	6	0	3.525643	-2.312159	-0.554351
76	6	0	3.139999	-3.792614	-0.670815
77	6	0	4.711885	-2.021697	-1.482694
78	1	0	3.862162	-2.130439	0.481423
79	6	0	4.328238	-4.710970	-0.379807
80	1	0	2.779339	-3.973439	-1.694836
81	1	0	2.312699	-4.028587	0.006500
82	6	0	5.903044	-2.941158	-1.211277
83	1	0	4.367709	-2.160685	-2.516725
84	1	0	4.994739	-0.967858	-1.393490
85	6	0	5.501438	-4.412844	-1.313338
86	1	0	4.020343	-5.758499	-0.468964
87	1	0	4.650156	-4.564439	0.661441
88	1	0	6.717943	-2.713430	-1.907750
89	1	0	6.293592	-2.754491	-0.201782
90	1	0	6.352880	-5.061315	-1.079093
91	1	0	5.203753	-4.635720	-2.347108
92	1	0	2.693736	-0.166866	-0.679143
93	6	0	0.661642	4.721826	1.191652
94	6	0	1.040880	3.645939	0.406197
95	6	0	2.359547	3.448122	-0.026923
96	6	0	3.325082	4.383405	0.311689
97	6	0	2.964606	5.475133	1.111220
98	6	0	1.654870	5.636639	1.549184
99	1	0	-0.370628	4.862039	1.495272
100	1	0	4.345416	4.253813	-0.024297
101	1	0	3.719294	6.203591	1.388539
102	1	0	1.391740	6.491844	2.163614
103	7	0	0.212729	2.634606	-0.104363
104	6	0	0.911746	1.794755	-0.917984
105	8	0	0.433079	0.844026	-1.508276

106	6	0	-1.153410	2.359491	0.297518
107	1	0	-1.257696	1.267235	0.296088
108	1	0	-1.271373	2.690291	1.333863
109	6	0	-2.199335	2.991996	-0.594601
110	6	0	-2.138821	2.816510	-1.981942
111	6	0	-3.241483	3.741291	-0.047283
112	6	0	-3.100647	3.397930	-2.803080
113	1	0	-1.324160	2.240772	-2.415273
114	6	0	-4.206940	4.317171	-0.869685
115	1	0	-3.290413	3.886429	1.029968
116	6	0	-4.133621	4.151889	-2.249362
117	1	0	-3.038562	3.266579	-3.879065
118	1	0	-5.012709	4.896918	-0.430415
119	1	0	-4.881820	4.605320	-2.892345
120	6	0	2.404493	2.182614	-0.888950
121	7	0	3.035709	1.027360	-0.280147
122	6	0	4.316038	1.204403	0.113711
123	8	0	5.048559	2.152619	-0.160576
124	8	0	4.720174	0.171506	0.899715
125	6	0	6.009412	0.226847	1.561775
126	6	0	6.020855	-1.047901	2.398010
127	1	0	6.957861	-1.125219	2.956606
128	1	0	5.190366	-1.039479	3.110303
129	1	0	5.921481	-1.931860	1.761898
130	6	0	6.084505	1.443008	2.483772
131	1	0	6.101074	2.371540	1.913912
132	1	0	5.222656	1.456150	3.158512
133	1	0	6.992083	1.382530	3.092488
134	6	0	7.151558	0.211241	0.549532
135	1	0	7.042238	-0.636693	-0.133092
136	1	0	7.171050	1.132281	-0.031974
137	1	0	8.104567	0.102091	1.077026
138	6	0	2.919618	2.547339	-2.299170
139	1	0	3.997969	2.694557	-2.241621
140	1	0	2.402241	3.416412	-2.703075
141	7	0	2.679203	1.435788	-3.266212
142	8	0	3.429345	0.480891	-3.225295
143	8	0	1.742862	1.558137	-4.040717

G1-II-*re*-TS3

Zero-point correction= 1.22979 (a.u.)

Thermal correction to Gibbs Free Energy= 1.15854(a.u.)

Sum of electronic and zero-point Energies= -3123.28982 (a.u.)

Sum of electronic and thermal Free Energies= -3123.36106(a.u.)

Standard orientation:

Center Number	Atomic Number	Atomic Type	Coordinates (Angstroms) X	Y	Z
1	6	0	0.244293	-0.237913	-4.367150
2	6	0	-0.951232	-0.310802	-3.413352
3	6	0	-0.560961	-0.863358	-2.044376
4	7	0	0.550475	-0.083410	-1.459537
5	6	0	1.433782	0.487300	-3.725133
6	6	0	1.707306	-0.155880	-2.361463
7	1	0	-0.037570	0.254817	-5.302553
8	1	0	0.557941	-1.257934	-4.627773
9	1	0	-1.742755	-0.945243	-3.823202
10	1	0	-1.390227	0.684094	-3.271629
11	1	0	-1.413006	-0.842204	-1.378388
12	1	0	-0.223300	-1.902823	-2.120428
13	1	0	2.330379	0.409495	-4.346848
14	1	0	1.211727	1.555859	-3.620773
15	1	0	1.815820	-1.233010	-2.553082
16	6	0	0.248208	1.064172	-0.737338
17	7	0	-0.761063	1.027606	0.102664
18	7	0	1.044216	2.138763	-0.982433
19	6	0	1.331089	3.334426	-0.186356
20	6	0	3.061274	0.243994	-1.744515
21	8	0	3.719880	1.177247	-2.180883
22	7	0	3.485298	-0.615325	-0.788183
23	1	0	2.803315	-1.270862	-0.429407
24	6	0	4.843512	-0.671830	-0.232132
25	6	0	2.027903	3.019663	1.135789
26	6	0	2.250878	4.312685	1.923037
27	6	0	3.053447	5.330446	1.107757
28	6	0	2.207806	4.265776	-1.024098
29	1	0	2.989329	2.543036	0.904632
30	1	0	1.444255	2.299101	1.721280
31	1	0	1.273869	4.743634	2.188785
32	1	0	2.764723	4.091108	2.864287
33	1	0	0.400013	3.876038	0.007676
34	1	0	3.140933	6.273839	1.657354
35	1	0	1.727308	4.453714	-1.991455
36	1	0	3.168640	3.768354	-1.218550
37	6	0	2.430153	5.577630	-0.268576
38	1	0	4.069543	4.942648	0.968256

39	1	0	3.065802	6.244817	-0.859000
40	1	0	1.463222	6.085300	-0.148040
41	1	0	1.689460	2.040787	-1.753545
42	6	0	5.888701	-0.824307	-1.359649
43	6	0	5.542573	-1.306818	-2.621330
44	6	0	7.240210	-0.603007	-1.078772
45	6	0	6.517773	-1.531364	-3.590070
46	1	0	4.504732	-1.517630	-2.858955
47	6	0	8.215429	-0.826637	-2.044659
48	1	0	7.534320	-0.246363	-0.095993
49	6	0	7.857762	-1.288210	-3.308773
50	1	0	6.222442	-1.897253	-4.568491
51	1	0	9.257729	-0.640090	-1.805521
52	1	0	8.616997	-1.458681	-4.065235
53	6	0	5.080498	0.582290	0.634130
54	6	0	5.648060	1.735584	0.082860
55	6	0	4.664850	0.619139	1.968952
56	6	0	5.853532	2.869060	0.865020
57	1	0	5.928359	1.745021	-0.964048
58	6	0	4.868441	1.752853	2.752154
59	1	0	4.196222	-0.252037	2.413689
60	6	0	5.478482	2.878582	2.206511
61	1	0	6.310985	3.747784	0.419900
62	1	0	4.550421	1.750414	3.790367
63	1	0	5.649579	3.761293	2.816027
64	6	0	4.956759	-1.988845	0.567180
65	6	0	5.853360	-2.110476	1.633650
66	6	0	4.251793	-3.127250	0.165165
67	6	0	5.993809	-3.315678	2.314190
68	1	0	6.441973	-1.254292	1.945435
69	6	0	4.388725	-4.333686	0.847885
70	1	0	3.600617	-3.097775	-0.703900
71	6	0	5.251171	-4.430870	1.934394
72	1	0	6.688203	-3.379773	3.145954
73	1	0	3.819251	-5.198099	0.520884
74	1	0	5.355875	-5.369050	2.469521
75	6	0	-1.334758	2.217386	0.736185
76	6	0	-2.060655	3.109628	-0.277898
77	6	0	-2.291681	1.821984	1.864549
78	1	0	-0.548457	2.802840	1.225270
79	6	0	-2.623351	4.357742	0.404588
80	1	0	-2.868769	2.516778	-0.726330
81	1	0	-1.383412	3.384691	-1.095274
82	6	0	-2.831059	3.069618	2.564241

83	1	0	-3.138854	1.263361	1.453653
84	1	0	-1.782762	1.153213	2.566373
85	6	0	-3.545269	3.983624	1.567187
86	1	0	-3.154708	4.978691	-0.324222
87	1	0	-1.790915	4.966329	0.789018
88	1	0	-3.513017	2.773610	3.368078
89	1	0	-2.002189	3.618117	3.034076
90	1	0	-3.916766	4.884883	2.066274
91	1	0	-4.422615	3.452601	1.170395
92	1	0	-1.370209	0.005948	0.395874
93	8	0	-0.259933	-1.526926	1.924181
94	8	0	-3.410903	0.160562	-1.485094
95	7	0	-5.182323	0.340091	-0.039382
96	7	0	-2.125557	-1.043000	0.846941
97	8	0	-2.023984	-2.947549	2.156641
98	6	0	0.685174	-2.457885	2.492378
99	6	0	-3.518400	-1.239393	0.559309
100	6	0	-3.980997	-0.168694	-0.463636
101	6	0	-4.542670	-1.044347	1.676777
102	6	0	-5.526607	-0.152710	1.228141
103	6	0	-4.658435	-1.617573	2.932556
104	1	0	-3.900953	-2.310739	3.279567
105	6	0	-1.537924	-1.925889	1.665758
106	6	0	-5.761269	-1.279666	3.727517
107	1	0	-5.860775	-1.715900	4.715936
108	6	0	-5.992603	1.224468	-0.854143
109	1	0	-5.310941	1.672514	-1.583278
110	6	0	-6.634464	0.177902	1.991855
111	1	0	-7.397154	0.854885	1.621651
112	6	0	-6.735167	-0.403545	3.259139
113	1	0	-7.590827	-0.163703	3.882564
114	6	0	0.277057	-2.908937	3.894231
115	1	0	-0.557640	-3.607047	3.861402
116	1	0	1.131656	-3.387569	4.384143
117	1	0	-0.014564	-2.039310	4.491365
118	6	0	1.962257	-1.628209	2.574362
119	1	0	2.163462	-1.154710	1.609002
120	1	0	1.850915	-0.830458	3.316295
121	1	0	2.817952	-2.254785	2.849021
122	6	0	0.863484	-3.639067	1.543515
123	1	0	1.633129	-4.317298	1.924328
124	1	0	-0.075774	-4.184123	1.435781
125	1	0	1.174121	-3.281538	0.555005
126	6	0	-3.862554	-2.632408	-0.062460

127	1	0	-4.886764	-2.647750	-0.436914
128	1	0	-3.666022	-3.399135	0.682600
129	7	0	-2.977181	-2.931958	-1.222086
130	8	0	-1.909989	-3.468518	-0.987826
131	8	0	-3.361228	-2.607956	-2.334189
132	1	0	-6.398249	2.023844	-0.225548
133	6	0	-7.105133	0.475773	-1.557548
134	6	0	-8.434080	0.871299	-1.427522
135	6	0	-6.792434	-0.634284	-2.349396
136	6	0	-9.446186	0.169228	-2.081221
137	1	0	-8.680715	1.733904	-0.812800
138	6	0	-7.801867	-1.336672	-2.997742
139	1	0	-5.754030	-0.943097	-2.460490
140	6	0	-9.131522	-0.936428	-2.864294
141	1	0	-10.479372	0.483908	-1.971874
142	1	0	-7.550541	-2.196895	-3.610279
143	1	0	-9.918810	-1.486005	-3.370576

G1-II-*si*-TS3

Zero-point correction= 1.22902 (a.u.)
Thermal correction to Gibbs Free Energy= 1.15785 (a.u.)
Sum of electronic and zero-point Energies= -3123.30004 (a.u.)
Sum of electronic and thermal Free Energies= -3123.37121 (a.u.)

Standard orientation:

Center Number	Atomic Number	Atomic Type	Coordinates (Angstroms) X	Y	Z
1	6	0	-0.484281	-5.053193	1.808116
2	6	0	0.981529	-4.628528	1.897386
3	6	0	1.282879	-3.423084	1.004190
4	7	0	0.364647	-2.317450	1.317685
5	6	0	-1.418144	-3.862026	2.029021
6	6	0	-1.020659	-2.723901	1.075156
7	1	0	-0.701548	-5.846642	2.530209
8	1	0	-0.677964	-5.468701	0.809686
9	1	0	1.640873	-5.456508	1.616169
10	1	0	1.224019	-4.361063	2.934059
11	1	0	2.310292	-3.092845	1.132390
12	1	0	1.153877	-3.671582	-0.057013
13	1	0	-2.460952	-4.147877	1.855889
14	1	0	-1.356836	-3.497320	3.061321
15	1	0	-0.998378	-3.138871	0.059601

16	6	0	0.799079	-1.277757	2.096718
17	7	0	2.040883	-0.895115	1.903507
18	7	0	-0.073421	-0.714921	2.962409
19	6	0	-0.009169	0.664888	3.433927
20	6	0	-2.141311	-1.666212	1.064358
21	8	0	-2.740686	-1.351631	2.088594
22	7	0	-2.546454	-1.316488	-0.176504
23	1	0	-1.892600	-1.435543	-0.943855
24	6	0	-3.800605	-0.608699	-0.452811
25	6	0	-1.249783	1.460360	3.017963
26	6	0	-1.125521	2.906847	3.509066
27	6	0	-0.920366	2.977940	5.023212
28	6	0	0.167357	0.710727	4.955693
29	1	0	-2.145197	0.993183	3.450626
30	1	0	-1.365798	1.413065	1.927851
31	1	0	-0.267651	3.382570	3.009817
32	1	0	-2.012488	3.475502	3.218593
33	1	0	0.853950	1.129380	2.946923
34	1	0	-0.805614	4.019196	5.343322
35	1	0	1.036949	0.115621	5.253560
36	1	0	-0.711715	0.235752	5.413984
37	6	0	0.291259	2.150730	5.452453
38	1	0	-1.816786	2.587366	5.523839
39	1	0	0.406619	2.167691	6.541061
40	1	0	1.202839	2.597552	5.029804
41	1	0	-0.987159	-1.150302	3.049290
42	6	0	-5.011346	-1.443835	0.021445
43	6	0	-4.899757	-2.796673	0.338064
44	6	0	-6.288435	-0.872565	-0.000273
45	6	0	-6.025990	-3.550652	0.662769
46	1	0	-3.929100	-3.279776	0.317978
47	6	0	-7.413389	-1.621103	0.325401
48	1	0	-6.404717	0.171279	-0.277263
49	6	0	-7.286816	-2.966125	0.665741
50	1	0	-5.910242	-4.600981	0.912509
51	1	0	-8.392153	-1.151740	0.306417
52	1	0	-8.163739	-3.552403	0.921134
53	6	0	-3.745298	0.795762	0.174214
54	6	0	-4.438537	1.121451	1.340874
55	6	0	-2.944762	1.774192	-0.425021
56	6	0	-4.410868	2.419767	1.845328
57	1	0	-5.002757	0.355943	1.860539
58	6	0	-2.930024	3.076997	0.066594
59	1	0	-2.349683	1.516321	-1.297945

60	6	0	-3.681745	3.407961	1.192052
61	1	0	-4.967216	2.655712	2.747588
62	1	0	-2.346811	3.838164	-0.441697
63	1	0	-3.684223	4.428351	1.564189
64	6	0	-3.973228	-0.558509	-1.988675
65	6	0	-4.599354	0.515951	-2.623660
66	6	0	-3.608044	-1.665829	-2.761362
67	6	0	-4.820065	0.498942	-3.999121
68	1	0	-4.911406	1.381255	-2.049056
69	6	0	-3.822268	-1.680740	-4.134668
70	1	0	-3.153968	-2.532741	-2.289194
71	6	0	-4.425991	-0.594259	-4.762443
72	1	0	-5.302090	1.349412	-4.471307
73	1	0	-3.521837	-2.548545	-4.713600
74	1	0	-4.594704	-0.604563	-5.834618
75	6	0	2.941882	-0.206782	2.810979
76	6	0	4.128570	-1.134918	3.109217
77	6	0	3.443733	1.094901	2.169389
78	1	0	2.449807	0.030590	3.762680
79	6	0	5.185251	-0.445969	3.973453
80	1	0	4.559155	-1.450784	2.148997
81	1	0	3.753969	-2.041572	3.598634
82	6	0	4.503847	1.785704	3.028044
83	1	0	3.869844	0.842957	1.186909
84	1	0	2.598105	1.769146	1.979452
85	6	0	5.675701	0.846646	3.318962
86	1	0	6.023820	-1.126614	4.154364
87	1	0	4.752941	-0.208913	4.956178
88	1	0	4.854464	2.694082	2.526299
89	1	0	4.052077	2.102530	3.979036
90	1	0	6.413219	1.344488	3.957890
91	1	0	6.180463	0.601874	2.374809
92	1	0	2.424644	-1.004306	0.717941
93	6	0	2.250543	0.054647	-1.378247
94	6	0	1.210849	0.880920	-0.555103
95	8	0	0.199172	0.453710	-0.034893
96	6	0	2.799657	2.367512	-1.277096
97	6	0	3.259643	1.135968	-1.760322
98	7	0	2.732262	-1.005190	-0.542344
99	6	0	3.624163	-1.928754	-0.926523
100	8	0	4.148352	-2.732490	-0.159998
101	8	0	3.901522	-1.922615	-2.270342
102	6	0	4.725993	-2.964055	-2.855372
103	6	0	4.092146	-4.333099	-2.621415

104	1	0	4.612354	-5.086932	-3.220592
105	1	0	4.142485	-4.614995	-1.570153
106	1	0	3.041986	-4.312894	-2.931851
107	6	0	6.151301	-2.885377	-2.316775
108	1	0	6.177048	-3.125260	-1.254568
109	1	0	6.788947	-3.588989	-2.861176
110	1	0	6.555612	-1.878144	-2.462482
111	6	0	4.703527	-2.626166	-4.342962
112	1	0	5.317344	-3.338933	-4.900545
113	1	0	3.683110	-2.672053	-4.736035
114	1	0	5.099171	-1.621080	-4.519387
115	7	0	1.597230	2.193036	-0.578653
116	6	0	0.790858	3.261795	-0.036282
117	1	0	0.023943	2.779867	0.583748
118	1	0	1.408450	3.893982	0.612248
119	6	0	0.130052	4.087598	-1.119952
120	6	0	-0.038731	5.461141	-0.953280
121	6	0	-0.389959	3.465884	-2.258164
122	6	0	-0.731535	6.206551	-1.905366
123	1	0	0.366770	5.947700	-0.069363
124	6	0	-1.082355	4.209431	-3.208352
125	1	0	-0.277298	2.390675	-2.387232
126	6	0	-1.256816	5.581489	-3.032892
127	1	0	-0.858725	7.275626	-1.765733
128	1	0	-1.494903	3.714655	-4.082139
129	1	0	-1.798989	6.160173	-3.773912
130	6	0	4.456923	1.082241	-2.454889
131	1	0	4.827046	0.134592	-2.827728
132	6	0	5.174616	2.266188	-2.661309
133	1	0	6.114246	2.234160	-3.202568
134	6	0	4.692416	3.481581	-2.185301
135	1	0	5.255469	4.392223	-2.363468
136	6	0	3.487255	3.553195	-1.482055
137	1	0	3.101172	4.499973	-1.118746
138	6	0	1.503093	-0.381964	-2.676040
139	1	0	2.082016	-1.143597	-3.192654
140	1	0	1.288843	0.477126	-3.309980
141	7	0	0.179698	-0.976760	-2.342742
142	8	0	-0.811228	-0.294705	-2.546515
143	8	0	0.152816	-2.095675	-1.861784

G1-I-*re*-IM4

Zero-point correction= 1.23411 (a.u.)

Thermal correction to Gibbs Free Energy= 1.16297 (a.u.)
Sum of electronic and zero-point Energies= -3123.28985 (a.u.)
Sum of electronic and thermal Free Energies= -3123.36098 (a.u.)

Standard orientation:

Center Number	Atomic Number	Atomic Type	Coordinates (Angstroms) X	Y	Z
1	6	0	-2.862745	-3.345213	-0.119288
2	6	0	-4.330256	-2.910164	-0.247407
3	6	0	-5.103654	-3.080198	1.060415
4	6	0	-5.050569	-4.527110	1.550334
5	6	0	-2.821994	-4.798661	0.401715
6	1	0	-4.799518	-3.519132	-1.033593
7	1	0	-4.360509	-1.870850	-0.587892
8	1	0	-4.663352	-2.416689	1.811750
9	1	0	-6.142985	-2.759114	0.926098
10	1	0	-2.432430	-3.313090	-1.131014
11	1	0	-5.589143	-4.631890	2.499216
12	1	0	-3.267214	-5.439523	-0.372554
13	6	0	-3.599855	-4.978067	1.706791
14	1	0	-5.554286	-5.181241	0.823725
15	1	0	-3.550819	-6.025940	2.023924
16	1	0	-3.121893	-4.384770	2.497162
17	6	0	-0.066368	0.310406	4.210633
18	6	0	-1.099850	-0.786856	4.427419
19	6	0	-1.585178	-1.306154	3.083432
20	7	0	-0.484183	-1.864493	2.282997
21	6	0	1.069068	-0.250863	3.366865
22	6	0	0.566508	-0.865331	2.043038
23	1	0	0.323651	0.692091	5.159483
24	1	0	-0.539884	1.153934	3.688145
25	1	0	-1.960789	-0.415012	4.991953
26	1	0	-0.653911	-1.609681	4.998901
27	1	0	-2.328053	-2.094181	3.210950
28	1	0	-2.090578	-0.490634	2.539424
29	1	0	1.810608	0.523044	3.127558
30	1	0	1.590144	-1.041562	3.917745
31	1	0	0.148903	-0.057310	1.426752
32	6	0	-0.954083	-2.580839	1.144875
33	7	0	-2.170316	-2.404356	0.760875
34	7	0	-0.017909	-3.451759	0.644777
35	6	0	0.096706	-3.856038	-0.752752
36	1	0	-2.549019	-0.200940	0.343546

37	6	0	1.868959	-1.373203	1.401338
38	8	0	2.497139	-2.312218	1.869198
39	7	0	2.326932	-0.573877	0.393502
40	1	0	1.666128	0.123403	0.064588
41	6	0	0.221940	-2.659128	-1.697891
42	6	0	0.376146	-3.118603	-3.145900
43	6	0	1.570459	-4.063648	-3.294279
44	6	0	1.265312	-4.830797	-0.889478
45	1	0	1.090684	-2.061899	-1.393968
46	1	0	-0.653162	-2.010170	-1.586282
47	1	0	-0.541796	-3.637142	-3.458777
48	1	0	0.480040	-2.248502	-3.801313
49	1	0	-0.799060	-4.415442	-1.047994
50	1	0	1.657047	-4.413620	-4.328765
51	1	0	1.075607	-5.704029	-0.254277
52	1	0	2.180153	-4.359904	-0.509959
53	6	0	1.446615	-5.262054	-2.347575
54	1	0	2.494662	-3.512360	-3.070095
55	1	0	2.320246	-5.917469	-2.436340
56	1	0	0.576038	-5.860892	-2.648657
57	1	0	-1.792388	-5.136152	0.545167
58	1	0	0.820082	-3.539106	1.208980
59	6	0	4.610547	-1.481832	-0.124613
60	6	0	3.746847	-0.243448	0.149399
61	6	0	3.811313	0.564567	-1.159675
62	6	0	4.195830	0.547682	1.390684
63	6	0	5.979666	-1.488719	0.143021
64	6	0	6.765982	-2.577475	-0.231973
65	6	0	6.197270	-3.665822	-0.885143
66	6	0	4.833121	-3.654170	-1.170068
67	6	0	4.054009	-2.566275	-0.797079
68	1	0	6.441130	-0.648240	0.652600
69	1	0	7.828261	-2.570591	-0.007991
70	1	0	6.809911	-4.514654	-1.171596
71	1	0	4.369998	-4.493294	-1.683221
72	1	0	2.995173	-2.545844	-1.025561
73	6	0	4.586156	-0.142192	2.545620
74	6	0	4.812426	0.542062	3.736712
75	6	0	4.035106	1.934613	1.461254
76	6	0	4.260587	2.617338	2.654945
77	6	0	4.648870	1.923806	3.797974
78	1	0	5.110081	-0.012135	4.621497
79	1	0	4.688115	-1.221470	2.513418
80	1	0	4.826306	2.455105	4.727891

81	1	0	4.136405	3.696183	2.680535
82	1	0	3.735099	2.493669	0.579640
83	6	0	2.820774	0.440920	-2.137487
84	6	0	2.955217	1.083680	-3.368588
85	6	0	4.079966	1.852775	-3.640749
86	6	0	4.943337	1.335887	-1.446294
87	6	0	5.074405	1.978853	-2.670703
88	1	0	1.934220	-0.160128	-1.959733
89	1	0	2.173874	0.968311	-4.113678
90	1	0	4.187643	2.346895	-4.601635
91	1	0	5.959660	2.574200	-2.871686
92	1	0	5.730957	1.430699	-0.704102
93	8	0	-5.103213	1.230008	-0.654545
94	8	0	-0.169389	0.607323	-0.659579
95	7	0	-0.597303	2.662918	0.302914
96	7	0	-3.044618	0.659270	0.078111
97	8	0	-4.882665	-0.048490	1.210709
98	6	0	-6.565490	1.258832	-0.585325
99	6	0	-2.402382	1.613966	-0.807944
100	6	0	-0.907135	1.544482	-0.406542
101	6	0	-2.732783	3.056951	-0.450789
102	6	0	-1.643842	3.600293	0.238436
103	6	0	-3.830668	3.853523	-0.723401
104	1	0	-4.663373	3.459724	-1.290564
105	6	0	-4.402798	0.568486	0.287394
106	6	0	-3.847076	5.171212	-0.253698
107	1	0	-4.706152	5.800743	-0.459806
108	6	0	0.677845	2.915887	0.951998
109	1	0	1.186284	1.950415	1.052579
110	6	0	-1.637927	4.900001	0.716382
111	1	0	-0.779106	5.309425	1.237006
112	6	0	-2.771327	5.678422	0.467035
113	1	0	-2.798026	6.702112	0.825845
114	6	0	-6.966588	2.075073	-1.809683
115	6	0	-7.116002	-0.158199	-0.706531
116	6	0	-7.012865	1.957460	0.693398
117	1	0	-6.788487	1.355450	1.573712
118	1	0	-8.091761	2.134525	0.650999
119	1	0	-6.509908	2.925587	0.787136
120	1	0	-8.053946	2.057903	-1.920160
121	1	0	-6.524504	1.653452	-2.717332
122	1	0	-6.659972	3.120898	-1.717030
123	1	0	-8.205672	-0.114076	-0.793638
124	1	0	-6.856167	-0.758974	0.164104

125	1	0	-6.719860	-0.640926	-1.605524
126	1	0	0.478609	3.281924	1.964963
127	6	0	1.551772	3.890402	0.195176
128	6	0	2.162513	4.952199	0.861760
129	6	0	1.808880	3.690688	-1.162925
130	6	0	3.034466	5.801493	0.182811
131	1	0	1.961839	5.108191	1.919487
132	6	0	2.672616	4.543311	-1.842511
133	1	0	1.346660	2.855463	-1.684338
134	6	0	3.290517	5.596952	-1.169724
135	1	0	3.509522	6.623140	0.709540
136	1	0	2.872238	4.375773	-2.895755
137	1	0	3.969080	6.256689	-1.701127
138	6	0	-2.581133	1.362256	-2.314783
139	1	0	-2.036198	2.100855	-2.900984
140	1	0	-3.644353	1.355016	-2.555947
141	7	0	-2.049734	0.023010	-2.714869
142	8	0	-2.659207	-0.952445	-2.315599
143	8	0	-1.056660	-0.005645	-3.415386

G1-I-*si*-IM4

Zero-point correction= 1.23212 (a.u.)

Thermal correction to Gibbs Free Energy= 1.16073 (a.u.)

Sum of electronic and zero-point Energies= -3123.28300 (a.u.)

Sum of electronic and thermal Free Energies= -3123.35439 (a.u.)

Standard orientation:

Center Number	Atomic Number	Atomic Type	Coordinates (Angstroms) X	Y	Z
1	6	0	-1.544030	-1.654045	-4.391345
2	6	0	-0.162844	-2.280879	-4.441901
3	6	0	0.707864	-1.704052	-3.339606
4	7	0	0.147422	-1.910276	-1.987343
5	6	0	-2.119820	-1.889947	-3.005115
6	6	0	-1.211007	-1.355672	-1.865372
7	1	0	-2.214100	-2.071047	-5.150102
8	1	0	-1.464903	-0.574278	-4.577688
9	1	0	0.326952	-2.093891	-5.402879
10	1	0	-0.244324	-3.367650	-4.315197
11	1	0	1.691916	-2.163755	-3.347526
12	1	0	0.870405	-0.627453	-3.502608
13	1	0	-3.095856	-1.402449	-2.913009

14	1	0	-2.264070	-2.965426	-2.839809
15	1	0	-1.151125	-0.261836	-1.956947
16	6	0	1.127767	-1.916519	-0.978734
17	7	0	2.367026	-1.738465	-1.324881
18	7	0	0.706225	-2.173894	0.296792
19	6	0	1.354491	-1.589285	1.459017
20	6	0	-2.047887	-1.684588	-0.617004
21	8	0	-2.003800	-2.739546	0.003924
22	7	0	-3.004896	-0.743803	-0.391404
23	1	0	-2.929998	0.134613	-0.889834
24	6	0	-4.062818	-0.821305	0.625749
25	6	0	0.593734	-0.361055	1.968304
26	6	0	1.297126	0.256739	3.177237
27	6	0	1.503534	-0.775028	4.288749
28	6	0	1.534563	-2.624068	2.568255
29	1	0	-0.436455	-0.648207	2.223287
30	1	0	0.542162	0.366436	1.151257
31	1	0	2.273652	0.643927	2.850709
32	1	0	0.722771	1.113549	3.549739
33	1	0	2.343202	-1.239180	1.151668
34	1	0	2.045367	-0.326697	5.129056
35	1	0	2.097539	-3.479314	2.176812
36	1	0	0.543497	-2.995917	2.868421
37	6	0	2.247697	-2.008097	3.771787
38	1	0	0.522733	-1.089505	4.668221
39	1	0	2.360356	-2.750545	4.568903
40	1	0	3.260241	-1.714906	3.463286
41	1	0	-0.216405	-2.572697	0.424878
42	6	0	-4.884112	-2.121946	0.523945
43	6	0	-4.944052	-2.862636	-0.655457
44	6	0	-5.708080	-2.495179	1.589662
45	6	0	-5.787656	-3.965192	-0.759278
46	1	0	-4.328020	-2.583886	-1.502657
47	6	0	-6.550288	-3.597338	1.488416
48	1	0	-5.692189	-1.920055	2.510611
49	6	0	-6.591620	-4.340834	0.312171
50	1	0	-5.810934	-4.533476	-1.683837
51	1	0	-7.175948	-3.871600	2.331983
52	1	0	-7.246598	-5.202449	0.231462
53	6	0	-3.395152	-0.634227	2.001099
54	6	0	-2.991202	-1.727870	2.772272
55	6	0	-3.086823	0.651931	2.454466
56	6	0	-2.351819	-1.532407	3.995614
57	1	0	-3.178119	-2.733293	2.411742

58	6	0	-2.441228	0.849151	3.671698
59	1	0	-3.376624	1.510208	1.854467
60	6	0	-2.086641	-0.245476	4.456658
61	1	0	-2.059095	-2.394359	4.587776
62	1	0	-2.225796	1.858333	4.011157
63	1	0	-1.598554	-0.095691	5.414828
64	6	0	-5.087253	0.287538	0.297822
65	6	0	-5.880766	0.855254	1.299668
66	6	0	-5.341141	0.641905	-1.030367
67	6	0	-6.875608	1.774683	0.983875
68	1	0	-5.720841	0.579860	2.337068
69	6	0	-6.338813	1.560214	-1.346555
70	1	0	-4.782140	0.178921	-1.838317
71	6	0	-7.107354	2.135562	-0.340807
72	1	0	-7.475352	2.205625	1.779558
73	1	0	-6.512910	1.820102	-2.386062
74	1	0	-7.886195	2.850859	-0.585896
75	6	0	3.459489	-2.500647	-0.726581
76	6	0	3.166103	-4.010217	-0.770446
77	6	0	4.715689	-2.192148	-1.551857
78	1	0	3.687278	-2.238356	0.322959
79	6	0	4.369575	-4.849711	-0.341900
80	1	0	2.898434	-4.264213	-1.806250
81	1	0	2.292440	-4.236209	-0.150182
82	6	0	5.918219	-3.059733	-1.174411
83	1	0	4.460714	-2.355898	-2.607416
84	1	0	4.950626	-1.125378	-1.455055
85	6	0	5.579071	-4.549861	-1.227657
86	1	0	4.117744	-5.915416	-0.379481
87	1	0	4.622976	-4.622504	0.704209
88	1	0	6.763425	-2.832394	-1.834839
89	1	0	6.249057	-2.820281	-0.155465
90	1	0	6.443132	-5.150144	-0.921627
91	1	0	5.342378	-4.832159	-2.262588
92	1	0	2.820169	0.249790	-0.537110
93	6	0	0.496091	4.634758	1.340002
94	6	0	0.968872	3.632083	0.510508
95	6	0	2.322779	3.514692	0.165978
96	6	0	3.229468	4.449305	0.641025
97	6	0	2.771818	5.465383	1.487471
98	6	0	1.427708	5.550946	1.834585
99	1	0	-0.558775	4.719523	1.578416
100	1	0	4.276384	4.378408	0.375193
101	1	0	3.476095	6.194068	1.873952

102	1	0	1.089228	6.348181	2.488656
103	7	0	0.217834	2.628972	-0.123492
104	6	0	1.008144	1.865984	-0.928380
105	8	0	0.651013	0.919012	-1.598380
106	6	0	-1.153048	2.262982	0.192924
107	1	0	-1.201040	1.168642	0.129719
108	1	0	-1.333001	2.529529	1.238464
109	6	0	-2.192638	2.883306	-0.712081
110	6	0	-2.140786	2.656911	-2.092703
111	6	0	-3.227124	3.655754	-0.183849
112	6	0	-3.110508	3.204822	-2.927418
113	1	0	-1.326323	2.067081	-2.508602
114	6	0	-4.196037	4.204911	-1.020566
115	1	0	-3.273065	3.832912	0.888450
116	6	0	-4.136824	3.982106	-2.393022
117	1	0	-3.060030	3.029260	-3.997548
118	1	0	-4.997674	4.802468	-0.598180
119	1	0	-4.890786	4.409851	-3.046634
120	6	0	2.472894	2.327352	-0.777137
121	7	0	3.121280	1.170267	-0.184043
122	6	0	4.416620	1.286304	0.252087
123	8	0	5.099023	2.283635	0.098085
124	8	0	4.792756	0.183021	0.908501
125	6	0	6.117033	0.126805	1.528222
126	6	0	6.078547	-1.187457	2.297968
127	1	0	7.078394	-1.435290	2.664734
128	1	0	5.407051	-1.107056	3.157308
129	1	0	5.726425	-2.001422	1.659860
130	6	0	6.309183	1.281715	2.508926
131	1	0	6.478407	2.229876	2.000966
132	1	0	5.428748	1.374298	3.153018
133	1	0	7.171449	1.060392	3.145332
134	6	0	7.187152	0.107219	0.444081
135	1	0	7.030972	-0.735953	-0.234160
136	1	0	7.174241	1.033884	-0.131113
137	1	0	8.172495	-0.001916	0.906929
138	6	0	3.060302	2.775090	-2.128425
139	1	0	4.123462	2.982095	-2.004740
140	1	0	2.518671	3.631282	-2.527325
141	7	0	2.943888	1.682600	-3.143357
142	8	0	3.691343	0.733362	-3.019764
143	8	0	2.109076	1.817823	-4.020351

G1-II-*re*-IM4

Zero-point correction= 1.23226 (a.u.)

Thermal correction to Gibbs Free Energy= 1.15789 (a.u.)

Sum of electronic and zero-point Energies= -3123.29369 (a.u.)

Sum of electronic and thermal Free Energies= -3123.36806 (a.u.)

Standard orientation:

Center Number	Atomic Number	Atomic Type	Coordinates (Angstroms) X	Y	Z
1	6	0	-0.178389	0.253338	4.428937
2	6	0	0.957292	0.137234	3.404475
3	6	0	0.505194	-0.576921	2.131018
4	7	0	-0.670528	0.099244	1.550095
5	6	0	-1.454259	0.840022	3.804322
6	6	0	-1.757255	0.042571	2.533756
7	1	0	0.135774	0.859952	5.283993
8	1	0	-0.410477	-0.747236	4.819345
9	1	0	1.812241	-0.396807	3.830307
10	1	0	1.315236	1.136023	3.121865
11	1	0	1.307179	-0.591914	1.406176
12	1	0	0.220961	-1.614016	2.339092
13	1	0	-2.301406	0.769656	4.492458
14	1	0	-1.305303	1.901203	3.577420
15	1	0	-1.774826	-1.011909	2.844292
16	6	0	-0.384568	1.307287	0.865639
17	7	0	0.712249	1.361916	0.183783
18	7	0	-1.318158	2.305301	1.037798
19	6	0	-1.627695	3.398672	0.121690
20	6	0	-3.157822	0.234215	1.933160
21	8	0	-3.963730	1.029480	2.394699
22	7	0	-3.414314	-0.662251	0.947875
23	1	0	-2.596533	-1.085659	0.525576
24	6	0	-4.717792	-0.955486	0.347577
25	6	0	-1.988954	2.915914	-1.282280
26	6	0	-2.245955	4.113391	-2.199745
27	6	0	-3.326752	5.036286	-1.629363
28	6	0	-2.777268	4.221556	0.700846
29	1	0	-2.889503	2.288455	-1.205905
30	1	0	-1.186271	2.284371	-1.678464
31	1	0	-1.308998	4.677618	-2.318101
32	1	0	-2.532897	3.765495	-3.198332
33	1	0	-0.766096	4.074849	0.056543
34	1	0	-3.435466	5.926920	-2.257937

35	1	0	-2.533656	4.527539	1.724948
36	1	0	-3.677372	3.591352	0.746283
37	6	0	-3.029668	5.444266	-0.184164
38	1	0	-4.285600	4.506692	-1.651946
39	1	0	-3.857912	6.035558	0.219732
40	1	0	-2.139662	6.087968	-0.160622
41	1	0	-2.061873	2.136890	1.699027
42	6	0	-5.788010	-1.150088	1.442420
43	6	0	-5.435636	-1.677534	2.686229
44	6	0	-7.140553	-0.942733	1.165199
45	6	0	-6.405752	-1.957224	3.641957
46	1	0	-4.392328	-1.878783	2.910328
47	6	0	-8.113854	-1.226468	2.120201
48	1	0	-7.443103	-0.548554	0.200227
49	6	0	-7.751247	-1.728886	3.365183
50	1	0	-6.106336	-2.355473	4.606466
51	1	0	-9.158934	-1.049602	1.885536
52	1	0	-8.508996	-1.942683	4.112238
53	6	0	-5.061216	0.175126	-0.639675
54	6	0	-5.631480	1.364960	-0.169381
55	6	0	-4.725966	0.084998	-1.992070
56	6	0	-5.905822	2.410786	-1.045084
57	1	0	-5.855491	1.464386	0.887243
58	6	0	-4.988402	1.138271	-2.867238
59	1	0	-4.263803	-0.818832	-2.375192
60	6	0	-5.594826	2.298577	-2.399736
61	1	0	-6.360848	3.320465	-0.663594
62	1	0	-4.723223	1.043031	-3.916108
63	1	0	-5.814172	3.115241	-3.081182
64	6	0	-4.616909	-2.338139	-0.332540
65	6	0	-5.595750	-2.729103	-1.254541
66	6	0	-3.635320	-3.266164	0.021141
67	6	0	-5.565698	-3.989375	-1.837382
68	1	0	-6.386298	-2.035367	-1.525040
69	6	0	-3.602665	-4.531501	-0.565855
70	1	0	-2.884953	-3.028850	0.768531
71	6	0	-4.561390	-4.896486	-1.501478
72	1	0	-6.331973	-4.264581	-2.555438
73	1	0	-2.820155	-5.227628	-0.280611
74	1	0	-4.534773	-5.879895	-1.959762
75	6	0	1.277187	2.613916	-0.299004
76	6	0	1.705607	3.514652	0.868676
77	6	0	2.489436	2.331645	-1.192072
78	1	0	0.577346	3.177738	-0.933195

79	6	0	2.324199	4.821691	0.370013
80	1	0	2.440927	2.952044	1.461611
81	1	0	0.850957	3.713575	1.525786
82	6	0	3.118638	3.628519	-1.704166
83	1	0	3.234825	1.775268	-0.605509
84	1	0	2.187244	1.688150	-2.025635
85	6	0	3.516821	4.546960	-0.547198
86	1	0	2.626828	5.445956	1.217482
87	1	0	1.565925	5.391924	-0.186840
88	1	0	3.988841	3.399739	-2.328975
89	1	0	2.396904	4.152001	-2.346835
90	1	0	3.932568	5.485788	-0.929000
91	1	0	4.310063	4.061436	0.038548
92	1	0	1.742727	-0.095018	-0.560357
93	8	0	0.426334	-0.765126	-2.366481
94	8	0	3.459611	-0.116365	1.479711
95	7	0	5.264689	0.033256	0.070388
96	7	0	2.210206	-0.868300	-1.069571
97	8	0	1.840201	-2.537719	-2.587673
98	6	0	-0.577416	-1.293135	-3.277665
99	6	0	3.497984	-1.366399	-0.653060
100	6	0	4.031606	-0.415178	0.453234
101	6	0	4.614029	-1.291801	-1.689317
102	6	0	5.631653	-0.473758	-1.183580
103	6	0	4.774578	-1.905758	-2.921263
104	1	0	3.995215	-2.545954	-3.316759
105	6	0	1.508497	-1.490197	-2.053745
106	6	0	5.950404	-1.667524	-3.642442
107	1	0	6.084732	-2.133997	-4.612535
108	6	0	6.071317	0.916303	0.892715
109	1	0	5.382441	1.368243	1.612632
110	6	0	6.807532	-0.237596	-1.876431
111	1	0	7.593468	0.382777	-1.459563
112	6	0	6.948591	-0.848140	-3.125408
113	1	0	7.858174	-0.681136	-3.693712
114	6	0	0.018815	-1.561458	-4.656067
115	1	0	0.698177	-2.412325	-4.639644
116	1	0	-0.790721	-1.765251	-5.363781
117	1	0	0.562886	-0.678514	-5.005691
118	6	0	-1.583741	-0.152512	-3.352194
119	1	0	-1.968854	0.081299	-2.355867
120	1	0	-1.114167	0.747206	-3.761995
121	1	0	-2.420810	-0.432439	-3.998695
122	6	0	-1.212799	-2.533613	-2.660054

123	1	0	-2.073096	-2.854221	-3.256292
124	1	0	-0.497866	-3.356261	-2.600919
125	1	0	-1.564253	-2.304022	-1.649618
126	6	0	3.452268	-2.817963	-0.108207
127	1	0	4.398326	-3.083579	0.362831
128	1	0	3.184490	-3.489742	-0.921649
129	7	0	2.389637	-2.974884	0.928522
130	8	0	1.252684	-3.153827	0.529627
131	8	0	2.717630	-2.908204	2.098977
132	1	0	6.478567	1.711835	0.259842
133	6	0	7.181266	0.173925	1.604955
134	6	0	8.512248	0.555593	1.454523
135	6	0	6.866599	-0.914029	2.424636
136	6	0	9.523944	-0.139480	2.116068
137	1	0	8.759758	1.403570	0.819980
138	6	0	7.874906	-1.609740	3.081503
139	1	0	5.826235	-1.206961	2.552185
140	6	0	9.206459	-1.223754	2.927427
141	1	0	10.558612	0.164526	1.992071
142	1	0	7.622327	-2.452979	3.716619
143	1	0	9.993297	-1.767642	3.440398

G1-II-*si*-IM4

Zero-point correction= 1.23201 (a.u.)

Thermal correction to Gibbs Free Energy= 1.16122 (a.u.)

Sum of electronic and zero-point Energies= -3123.30023 (a.u.)

Sum of electronic and thermal Free Energies= -3123.37102 (a.u.)

Standard orientation:

Center Number	Atomic Number	Atomic Type	Coordinates (Angstroms) X	Y	Z
1	6	0	-0.701745	-3.909931	3.617641
2	6	0	0.785818	-3.565264	3.532738
3	6	0	1.128606	-2.834556	2.231894
4	7	0	0.270773	-1.655411	2.053393
5	6	0	-1.572464	-2.686545	3.324804
6	6	0	-1.130955	-2.066677	1.986942
7	1	0	-0.946994	-4.327443	4.599541
8	1	0	-0.932461	-4.686322	2.875035
9	1	0	1.394760	-4.472789	3.607486
10	1	0	1.065451	-2.922028	4.376833
11	1	0	2.169023	-2.521598	2.228227

12	1	0	0.981111	-3.491703	1.364604
13	1	0	-2.631118	-2.964755	3.283237
14	1	0	-1.470898	-1.933571	4.115642
15	1	0	-1.133670	-2.873060	1.241275
16	6	0	0.767590	-0.410672	2.413242
17	7	0	2.023491	-0.205752	2.150650
18	7	0	-0.113914	0.466525	2.974636
19	6	0	-0.022435	1.918274	2.869350
20	6	0	-2.211846	-1.076961	1.511422
21	8	0	-2.787566	-0.318704	2.286317
22	7	0	-2.628347	-1.297571	0.242589
23	1	0	-1.986733	-1.752323	-0.399031
24	6	0	-3.835784	-0.696024	-0.332528
25	6	0	-1.225339	2.504665	2.123046
26	6	0	-1.088288	4.027044	2.020962
27	6	0	-0.954942	4.675359	3.399922
28	6	0	0.097184	2.556783	4.258757
29	1	0	-2.146211	2.249232	2.664621
30	1	0	-1.313013	2.034925	1.134869
31	1	0	-0.191559	4.269911	1.430582
32	1	0	-1.942710	4.443980	1.480604
33	1	0	0.873271	2.147437	2.281252
34	1	0	-0.838345	5.760301	3.302281
35	1	0	0.944986	2.122337	4.798366
36	1	0	-0.805572	2.293694	4.827998
37	6	0	0.222668	4.077596	4.170052
38	1	0	-1.880172	4.502771	3.966411
39	1	0	0.290338	4.511796	5.172975
40	1	0	1.158985	4.330583	3.651455
41	1	0	-1.055454	0.128609	3.145797
42	6	0	-5.088118	-1.109072	0.472006
43	6	0	-5.070947	-2.182009	1.361864
44	6	0	-6.314281	-0.492645	0.200570
45	6	0	-6.237707	-2.604406	1.994815
46	1	0	-4.141905	-2.705972	1.557326
47	6	0	-7.479394	-0.907994	0.835194
48	1	0	-6.359328	0.321574	-0.517029
49	6	0	-7.446054	-1.965087	1.741489
50	1	0	-6.195973	-3.438147	2.689100
51	1	0	-8.416882	-0.407093	0.614186
52	1	0	-8.354673	-2.291354	2.237622
53	6	0	-3.643832	0.827289	-0.433587
54	6	0	-4.251430	1.721131	0.449692
55	6	0	-2.794046	1.336364	-1.421501

56	6	0	-4.080120	3.095676	0.299361
57	1	0	-4.858408	1.343968	1.264573
58	6	0	-2.634332	2.709928	-1.583010
59	1	0	-2.271099	0.651379	-2.084228
60	6	0	-3.288436	3.595583	-0.729881
61	1	0	-4.568119	3.774029	0.993433
62	1	0	-2.012959	3.089781	-2.387174
63	1	0	-3.168773	4.667091	-0.864267
64	6	0	-4.064411	-1.352645	-1.713366
65	6	0	-4.652649	-0.654811	-2.770017
66	6	0	-3.799955	-2.715556	-1.881595
67	6	0	-4.938507	-1.295604	-3.973591
68	1	0	-4.885057	0.399346	-2.660919
69	6	0	-4.078400	-3.353764	-3.084753
70	1	0	-3.377540	-3.291834	-1.063060
71	6	0	-4.646523	-2.644925	-4.139859
72	1	0	-5.391403	-0.731538	-4.783189
73	1	0	-3.855334	-4.410618	-3.193600
74	1	0	-4.864668	-3.142364	-5.079607
75	6	0	2.897129	0.770394	2.765041
76	6	0	4.030709	0.023361	3.486011
77	6	0	3.495072	1.689593	1.691584
78	1	0	2.387463	1.394647	3.513672
79	6	0	5.072166	0.981642	4.066363
80	1	0	4.498267	-0.654874	2.758981
81	1	0	3.598330	-0.606861	4.271723
82	6	0	4.542647	2.648572	2.256656
83	1	0	3.951662	1.051313	0.919827
84	1	0	2.690750	2.247390	1.196886
85	6	0	5.654693	1.886760	2.979444
86	1	0	5.869475	0.417539	4.562016
87	1	0	4.601113	1.606263	4.838563
88	1	0	4.958324	3.264394	1.451174
89	1	0	4.059898	3.336541	2.965314
90	1	0	6.380775	2.586118	3.407997
91	1	0	6.195774	1.268201	2.250771
92	1	0	2.454017	-0.953340	0.741234
93	6	0	2.179610	-0.617275	-1.368955
94	6	0	1.191462	0.479513	-0.856281
95	8	0	0.130433	0.276169	-0.306170
96	6	0	2.932565	1.577858	-1.867461
97	6	0	3.258867	0.226816	-2.034377
98	7	0	2.637339	-1.368472	-0.225673
99	6	0	3.480990	-2.427619	-0.244668

100	8	0	3.970599	-2.916588	0.758538
101	8	0	3.707008	-2.891579	-1.500094
102	6	0	4.447056	-4.130204	-1.702089
103	6	0	3.750872	-5.281087	-0.981387
104	1	0	4.179386	-6.231210	-1.314137
105	1	0	3.864114	-5.198744	0.099043
106	1	0	2.683691	-5.281057	-1.226121
107	6	0	5.895501	-3.955524	-1.259795
108	1	0	5.957657	-3.798880	-0.183209
109	1	0	6.469315	-4.849224	-1.523289
110	1	0	6.347086	-3.098673	-1.770263
111	6	0	4.366924	-4.330117	-3.211441
112	1	0	4.916875	-5.229654	-3.500401
113	1	0	3.326884	-4.447012	-3.531661
114	1	0	4.801987	-3.477254	-3.741610
115	7	0	1.704799	1.701734	-1.201752
116	6	0	1.036148	2.947875	-0.911937
117	1	0	0.177941	2.688967	-0.280698
118	1	0	1.705507	3.591072	-0.325273
119	6	0	0.547143	3.686856	-2.140148
120	6	0	0.098278	5.000945	-1.989190
121	6	0	0.452763	3.074722	-3.388619
122	6	0	-0.457732	5.685002	-3.064819
123	1	0	0.168038	5.481452	-1.015722
124	6	0	-0.096433	3.762280	-4.469523
125	1	0	0.793397	2.051560	-3.517125
126	6	0	-0.558030	5.065364	-4.309969
127	1	0	-0.812886	6.702310	-2.932657
128	1	0	-0.169217	3.273501	-5.436036
129	1	0	-0.993106	5.596786	-5.150286
130	6	0	4.434712	-0.120931	-2.677932
131	1	0	4.698428	-1.164441	-2.806854
132	6	0	5.275395	0.896113	-3.143685
133	1	0	6.199064	0.637888	-3.650313
134	6	0	4.937236	2.233886	-2.962217
135	1	0	5.600895	3.010416	-3.328669
136	6	0	3.752291	2.600360	-2.318753
137	1	0	3.479903	3.642387	-2.188094
138	6	0	1.405522	-1.440440	-2.423991
139	1	0	1.985383	-2.309327	-2.727907
140	1	0	1.131083	-0.818445	-3.274276
141	7	0	0.119913	-1.948444	-1.858339
142	8	0	-0.913714	-1.493993	-2.316825
143	8	0	0.171810	-2.778781	-0.971014

G1-P-*R*

Zero-point correction= 0.42472 (a.u.)

Thermal correction to Gibbs Free Energy= 0.38208 (a.u.)

Sum of electronic and zero-point Energies= -1353.50802 (a.u.)

Sum of electronic and thermal Free Energies= 1353.55065 (a.u.)

Standard orientation:

Center Number	Atomic Number	Atomic Type	Coordinates (Angstroms) X	Y	Z
1	1	0	-1.757966	-0.722562	1.469722
2	8	0	-3.902780	-0.176650	0.797290
3	8	0	0.447847	-1.605086	1.640988
4	7	0	1.707128	0.244274	1.048940
5	7	0	-1.698815	-0.008730	0.750793
6	8	0	-2.912663	0.832228	-0.991298
7	6	0	-5.263862	-0.055731	0.278902
8	6	0	-0.427622	0.057437	0.074298
9	6	0	0.611763	-0.567790	1.033497
10	6	0	0.171243	1.421986	-0.199012
11	6	0	1.462539	1.436408	0.344354
12	6	0	-0.289469	2.504333	-0.927630
13	1	0	-1.286406	2.486586	-1.353513
14	6	0	-2.864796	0.256990	0.081664
15	6	0	0.555579	3.608906	-1.085817
16	1	0	0.209635	4.472426	-1.643707
17	6	0	2.933061	-0.072982	1.756988
18	1	0	2.697485	-0.930257	2.394493
19	6	0	2.319324	2.511719	0.179551
20	1	0	3.325247	2.500393	0.584939
21	6	0	1.838257	3.605454	-0.545729
22	1	0	2.484235	4.465498	-0.691091
23	6	0	-5.631000	1.414863	0.117671
24	1	0	-5.043158	1.884652	-0.671270
25	1	0	-6.691836	1.496281	-0.137068
26	1	0	-5.463478	1.950441	1.056953
27	6	0	-6.097696	-0.701362	1.377548
28	1	0	-5.807606	-1.746485	1.515861
29	1	0	-5.959066	-0.173252	2.324878
30	1	0	-7.157199	-0.665841	1.110496
31	6	0	-5.394294	-0.836421	-1.024039
32	1	0	-6.449108	-0.884757	-1.310174

33	1	0	-4.835128	-0.360991	-1.830022
34	1	0	-5.026287	-1.857657	-0.889725
35	6	0	-0.378693	-0.738880	-1.256269
36	1	0	0.618071	-0.695279	-1.693015
37	1	0	-1.143380	-0.357953	-1.933250
38	7	0	-0.684318	-2.179070	-1.015209
39	8	0	-1.842503	-2.462922	-0.765299
40	8	0	0.237103	-2.971060	-1.069938
41	1	0	3.200778	0.771097	2.401442
42	6	0	4.065448	-0.400541	0.806764
43	6	0	5.308379	0.214297	0.943572
44	6	0	3.870263	-1.336256	-0.212714
45	6	0	6.351042	-0.102827	0.074798
46	1	0	5.461802	0.944772	1.734470
47	6	0	4.909507	-1.651669	-1.080676
48	1	0	2.902492	-1.822840	-0.322016
49	6	0	6.152270	-1.034663	-0.938909
50	1	0	7.315327	0.382565	0.188233
51	1	0	4.751772	-2.382782	-1.867171
52	1	0	6.962327	-1.281068	-1.617958

G1-P-*S*

Zero-point correction= 0.42472 (a.u.)

Thermal correction to Gibbs Free Energy= 0.38209 (a.u.)

Sum of electronic and zero-point Energies= -1353.50802 (a.u.)

Sum of electronic and thermal Free Energies= -1353.55065 (a.u.)

Standard orientation:

Center Number	Atomic Number	Atomic Type	Coordinates (Angstroms) X	Y	Z
1	1	0	1.757966	-0.722560	1.469721
2	6	0	-2.319330	2.511714	0.179551
3	6	0	-1.462542	1.436406	0.344354
4	6	0	-0.171246	1.421987	-0.199011
5	6	0	0.289463	2.504335	-0.927629
6	6	0	-0.555587	3.608907	-1.085815
7	6	0	-1.838266	3.605451	-0.545728
8	1	0	-3.325253	2.500384	0.584938
9	1	0	1.286401	2.486591	-1.353511
10	1	0	-0.209646	4.472428	-1.643704
11	1	0	-2.484247	4.465492	-0.691090
12	7	0	-1.707129	0.244270	1.048940

13	6	0	-0.611762	-0.567792	1.033494
14	8	0	-0.447845	-1.605090	1.640982
15	6	0	-2.933061	-0.072989	1.756988
16	1	0	-2.697484	-0.930264	2.394492
17	1	0	-3.200778	0.771089	2.401443
18	6	0	-4.065448	-0.400546	0.806764
19	6	0	-3.870265	-1.336260	-0.212716
20	6	0	-5.308379	0.214293	0.943574
21	6	0	-4.909511	-1.651671	-1.080676
22	1	0	-2.902496	-1.822846	-0.322019
23	6	0	-6.351043	-0.102828	0.074800
24	1	0	-5.461800	0.944766	1.734473
25	6	0	-6.152273	-1.034664	-0.938908
26	1	0	-4.751778	-2.382783	-1.867173
27	1	0	-7.315327	0.382565	0.188237
28	1	0	-6.962331	-1.281067	-1.617956
29	6	0	0.427622	0.057439	0.074298
30	7	0	1.698814	-0.008724	0.750795
31	6	0	2.864797	0.256995	0.081666
32	8	0	2.912664	0.832233	-0.991296
33	8	0	3.902780	-0.176645	0.797293
34	6	0	5.263861	-0.055730	0.278902
35	6	0	6.097696	-0.701361	1.377550
36	1	0	7.157198	-0.665844	1.110496
37	1	0	5.959069	-0.173245	2.324877
38	1	0	5.807602	-1.746482	1.515868
39	6	0	5.631002	1.414862	0.117667
40	1	0	5.043162	1.884650	-0.671276
41	1	0	5.463481	1.950443	1.056947
42	1	0	6.691838	1.496277	-0.137072
43	6	0	5.394289	-0.836425	-1.024036
44	1	0	5.026284	-1.857661	-0.889717
45	1	0	4.835120	-0.360999	-1.830019
46	1	0	6.449102	-0.884760	-1.310176
47	6	0	0.378697	-0.738877	-1.256270
48	1	0	1.143382	-0.357946	-1.933251
49	1	0	-0.618067	-0.695280	-1.693016
50	7	0	0.684329	-2.179066	-1.015211
51	8	0	1.842515	-2.462912	-0.765300
52	8	0	-0.237088	-2.971060	-1.069941

G1-I-*re*-TS2-a

Zero-point correction= 1.23096 (a.u.)

Thermal correction to Gibbs Free Energy= 1.15947 (a.u.)
Sum of electronic and zero-point Energies= -3123.26764 (a.u.)
Sum of electronic and thermal Free Energies= -3123.33912 (a.u.)

Standard orientation:

Center Number	Atomic Number	Atomic Type	Coordinates (Angstroms) X	Y	Z
1	6	0	3.345684	-0.151345	-1.104614
2	1	0	3.253492	0.268276	-0.096818
3	1	0	3.634505	0.664255	-1.777913
4	6	0	4.391619	-1.246174	-1.119993
5	6	0	4.157685	-2.466394	-0.477394
6	6	0	5.623592	-1.022110	-1.733934
7	6	0	5.150071	-3.441655	-0.450680
8	1	0	3.205969	-2.651200	0.018783
9	6	0	6.617400	-1.998470	-1.704049
10	1	0	5.806359	-0.074917	-2.236746
11	6	0	6.381655	-3.210240	-1.061816
12	1	0	4.960158	-4.384396	0.053204
13	1	0	7.572227	-1.813268	-2.186346
14	1	0	7.153369	-3.973514	-1.037983
15	6	0	0.596969	-3.456304	-0.891888
16	1	0	1.642844	-3.429278	-1.157482
17	1	0	-0.164048	-3.857325	-1.541340
18	7	0	0.300882	-3.456969	0.416207
19	8	0	-0.887569	-3.670792	0.784183
20	8	0	1.180595	-3.159995	1.284082
21	8	0	-2.352300	-2.790072	-1.937183
22	8	0	0.963155	-0.311524	0.580328
23	7	0	2.018761	-0.607791	-1.458579
24	7	0	-1.386140	-1.069834	-0.823158
25	8	0	-3.618026	-1.013012	-1.312075
26	6	0	-3.472095	-3.645786	-2.303764
27	6	0	-0.222108	-1.224455	-1.363823
28	6	0	0.961520	-0.673117	-0.582399
29	6	0	0.281927	-1.426352	-2.744861
30	6	0	1.632283	-1.024786	-2.737327
31	6	0	-0.289515	-1.872502	-3.928557
32	1	0	-1.327071	-2.181674	-3.951944
33	6	0	-2.544966	-1.577609	-1.389943
34	6	0	0.491903	-1.917269	-5.085516
35	1	0	0.053882	-2.268676	-6.013934
36	6	0	2.419508	-1.063066	-3.877308

37	1	0	3.462592	-0.766518	-3.850188
38	6	0	1.825488	-1.517088	-5.057380
39	1	0	2.420038	-1.561276	-5.964495
40	6	0	-2.779236	-4.896634	-2.830012
41	6	0	-4.281394	-3.969964	-1.053380
42	6	0	-4.319863	-2.996952	-3.393282
43	1	0	-4.839702	-2.116289	-3.016724
44	1	0	-5.058497	-3.718922	-3.754638
45	1	0	-3.693817	-2.705699	-4.243675
46	1	0	-3.522659	-5.632255	-3.149515
47	1	0	-2.159536	-5.343611	-2.047435
48	1	0	-2.141253	-4.653659	-3.685641
49	1	0	-5.051858	-4.708590	-1.294537
50	1	0	-4.766539	-3.074293	-0.660461
51	1	0	-3.618115	-4.388721	-0.290195
52	6	0	-2.537619	1.947517	4.268041
53	6	0	-1.160435	2.499366	4.624937
54	6	0	-0.059643	1.466424	4.333676
55	7	0	-0.473334	0.577304	3.237599
56	6	0	-2.641044	1.721284	2.754544
57	6	0	-1.336397	1.104053	2.182758
58	1	0	-3.325045	2.634537	4.590386
59	1	0	-2.701851	1.004308	4.804556
60	1	0	-1.107451	2.772354	5.682662
61	1	0	-0.966965	3.401608	4.038819
62	1	0	0.877643	1.980250	4.104452
63	1	0	0.096548	0.827561	5.208695
64	1	0	-3.490377	1.072393	2.516552
65	1	0	-2.830657	2.677942	2.254060
66	1	0	-1.549316	0.268663	1.517235
67	6	0	-0.087400	-0.718240	3.163185
68	7	0	-0.934090	-1.652012	2.734814
69	7	0	1.144549	-1.084990	3.540124
70	6	0	2.359728	-0.270950	3.395762
71	1	0	-0.538158	-2.482491	2.287327
72	6	0	-0.593730	2.165460	1.361683
73	8	0	0.109849	3.018627	1.888075
74	7	0	-0.900367	2.094597	0.043162
75	1	0	-1.327981	1.223480	-0.258471
76	6	0	2.990216	0.046804	4.754769
77	6	0	4.276240	0.858890	4.580846
78	6	0	5.277851	0.133513	3.679958
79	6	0	3.359501	-1.022173	2.516045
80	1	0	3.217962	-0.905412	5.254074

81	1	0	2.283317	0.581448	5.394957
82	1	0	4.026132	1.831092	4.133897
83	1	0	4.719720	1.066089	5.560073
84	1	0	2.069137	0.650880	2.876693
85	1	0	6.173120	0.747282	3.534774
86	1	0	2.872714	-1.250097	1.567714
87	1	0	3.600551	-1.983688	2.994268
88	6	0	4.643401	-0.212572	2.332219
89	1	0	5.601939	-0.792983	4.172992
90	1	0	5.344661	-0.768597	1.700673
91	1	0	4.407797	0.722992	1.803337
92	1	0	1.292622	-2.089335	3.497410
93	6	0	-1.646051	4.234886	-0.935303
94	6	0	-0.622685	3.078350	-1.024487
95	6	0	0.807714	3.628096	-0.936485
96	6	0	-0.816100	2.304383	-2.345348
97	6	0	-2.393989	4.661625	-2.033934
98	6	0	-3.289612	5.725521	-1.917641
99	6	0	-3.448991	6.381542	-0.703701
100	6	0	-2.689429	5.977597	0.393360
101	6	0	-1.791488	4.922837	0.277400
102	1	0	-2.285635	4.169031	-2.993735
103	1	0	-3.864240	6.034092	-2.785599
104	1	0	-4.150632	7.204768	-0.612578
105	1	0	-2.789163	6.492130	1.344408
106	1	0	-1.177655	4.641395	1.126472
107	6	0	-2.015470	1.603535	-2.541393
108	6	0	-2.280535	0.963345	-3.745306
109	6	0	0.118553	2.325942	-3.380300
110	6	0	-0.150318	1.688502	-4.593035
111	6	0	-1.351725	1.017794	-4.784234
112	1	0	-3.216392	0.425294	-3.864703
113	1	0	-2.766114	1.564474	-1.755446
114	1	0	-1.555948	0.521889	-5.728254
115	1	0	0.592108	1.714899	-5.384950
116	1	0	1.060689	2.847957	-3.253219
117	6	0	1.849221	2.819292	-0.476164
118	6	0	3.160990	3.281137	-0.487344
119	6	0	3.456886	4.554083	-0.971577
120	6	0	1.109566	4.901410	-1.420093
121	6	0	2.425951	5.361031	-1.438955
122	1	0	1.619622	1.834676	-0.080531
123	1	0	3.957422	2.645200	-0.109634
124	1	0	4.481109	4.913571	-0.977689

125	1	0	2.639287	6.355841	-1.817872
126	1	0	0.315567	5.543720	-1.787790
127	6	0	-2.371779	-1.708530	3.021978
128	6	0	-3.250149	-1.724168	1.768676
129	6	0	-2.633309	-2.963836	3.867432
130	1	0	-2.616950	-0.821889	3.619564
131	6	0	-4.727491	-1.849054	2.145211
132	1	0	-2.938421	-2.569856	1.144013
133	1	0	-3.095952	-0.827981	1.164876
134	6	0	-4.113357	-3.109129	4.226103
135	1	0	-2.304667	-3.832411	3.280379
136	1	0	-2.016019	-2.929608	4.771924
137	6	0	-4.989401	-3.106901	2.972689
138	1	0	-5.335815	-1.842878	1.235174
139	1	0	-5.032051	-0.967377	2.729003
140	1	0	-4.266182	-4.026191	4.804616
141	1	0	-4.411816	-2.272183	4.872937
142	1	0	-6.047470	-3.175598	3.247261
143	1	0	-4.755355	-3.992060	2.365464

G1-I-*si*-TS2-a

Zero-point correction= 1.23115 (a.u.)

Thermal correction to Gibbs Free Energy= 1.15797 (a.u.)

Sum of electronic and zero-point Energies= -3123.26746 (a.u.)

Sum of electronic and thermal Free Energies= -3123.34064 (a.u.)

Standard orientation:

Center Number	Atomic Number	Atomic Type	Coordinates (Angstroms) X	Y	Z
1	6	0	-3.331166	-0.105707	-1.132446
2	1	0	-3.242962	0.280234	-0.110669
3	1	0	-3.605789	0.734120	-1.781507
4	6	0	-4.388548	-1.187796	-1.191679
5	6	0	-4.174623	-2.431976	-0.588854
6	6	0	-5.613120	-0.927216	-1.805752
7	6	0	-5.179989	-3.394256	-0.602560
8	1	0	-3.228999	-2.645231	-0.092175
9	6	0	-6.619590	-1.890697	-1.816784
10	1	0	-5.779742	0.038776	-2.277372
11	6	0	-6.403970	-3.126339	-1.214155
12	1	0	-5.006769	-4.356150	-0.129809
13	1	0	-7.567879	-1.676863	-2.300100

14	1	0	-7.185269	-3.880086	-1.222625
15	8	0	2.367935	-2.742739	-1.978513
16	8	0	-0.963696	-0.319305	0.563209
17	7	0	-2.006031	-0.564615	-1.489319
18	7	0	1.392799	-1.044665	-0.840891
19	8	0	3.627307	-0.974049	-1.317241
20	6	0	3.491995	-3.588523	-2.355544
21	6	0	0.233228	-1.185017	-1.394028
22	6	0	-0.955188	-0.652441	-0.607893
23	6	0	-0.261020	-1.353546	-2.782735
24	6	0	-1.610730	-0.949364	-2.775630
25	6	0	0.318812	-1.773606	-3.971721
26	1	0	1.355759	-2.085560	-3.994006
27	6	0	2.555812	-1.539267	-1.410550
28	6	0	-0.453305	-1.787821	-5.135763
29	1	0	-0.008833	-2.118485	-6.068788
30	6	0	-2.388496	-0.957252	-3.922657
31	1	0	-3.431146	-0.659033	-3.897024
32	6	0	-1.785751	-1.383621	-5.108728
33	1	0	-2.372940	-1.403910	-6.021479
34	6	0	2.804402	-4.835289	-2.898215
35	6	0	4.303317	-3.926007	-1.109730
36	6	0	4.335649	-2.920037	-3.436515
37	1	0	4.850026	-2.041275	-3.048111
38	1	0	5.078969	-3.631923	-3.808499
39	1	0	3.706777	-2.621052	-4.282096
40	1	0	3.550847	-5.563503	-3.227405
41	1	0	2.186657	-5.294903	-2.121506
42	1	0	2.165173	-4.583331	-3.750352
43	1	0	5.075295	-4.659591	-1.361067
44	1	0	4.786703	-3.034420	-0.705688
45	1	0	3.641876	-4.356537	-0.351415
46	6	0	-0.590594	-3.431336	-0.987064
47	1	0	-1.633725	-3.395046	-1.261727
48	1	0	0.177641	-3.812720	-1.640015
49	7	0	-0.307506	-3.463984	0.323027
50	8	0	0.876792	-3.689935	0.698161
51	8	0	-1.195425	-3.185328	1.188820
52	6	0	2.495411	1.820105	4.359957
53	6	0	1.116474	2.373131	4.708777
54	6	0	0.012985	1.357955	4.368347
55	7	0	0.441231	0.494172	3.257175
56	6	0	2.623236	1.635701	2.842446
57	6	0	1.323763	1.046477	2.231655

58	1	0	3.282582	2.491282	4.714828
59	1	0	2.642756	0.860208	4.871303
60	1	0	1.046492	2.616315	5.772765
61	1	0	0.940551	3.292473	4.144172
62	1	0	-0.914981	1.885776	4.132479
63	1	0	-0.167773	0.698184	5.222524
64	1	0	3.471564	0.986538	2.601458
65	1	0	2.828621	2.604004	2.371684
66	1	0	1.539381	0.231253	1.542757
67	6	0	0.056303	-0.798464	3.146397
68	7	0	0.904588	-1.723511	2.701776
69	7	0	-1.178271	-1.175291	3.505479
70	6	0	-2.395719	-0.363484	3.369457
71	1	0	0.508435	-2.538690	2.227283
72	6	0	0.602761	2.138981	1.431974
73	8	0	-0.088972	2.992058	1.973948
74	7	0	0.914502	2.093044	0.113626
75	1	0	1.328506	1.221399	-0.205702
76	6	0	-3.039870	-0.085660	4.730908
77	6	0	-4.333296	0.716874	4.569175
78	6	0	-5.320668	0.004547	3.642772
79	6	0	-3.381690	-1.096394	2.458911
80	1	0	-3.260799	-1.052007	5.205657
81	1	0	-2.342537	0.440340	5.388812
82	1	0	-4.091200	1.703494	4.150106
83	1	0	-4.785721	0.892604	5.550480
84	1	0	-2.104576	0.573432	2.878572
85	1	0	-6.222768	0.610979	3.509108
86	1	0	-2.886084	-1.288556	1.507140
87	1	0	-3.614070	-2.075918	2.903664
88	6	0	-4.673920	-0.296609	2.290149
89	1	0	-5.636469	-0.939059	4.108218
90	1	0	-5.364751	-0.842900	1.638939
91	1	0	-4.446213	0.655747	1.788398
92	1	0	-1.322712	-2.178973	3.440436
93	6	0	1.672413	4.251040	-0.819424
94	6	0	0.643916	3.100862	-0.933322
95	6	0	-0.784454	3.654746	-0.837198
96	6	0	0.837128	2.356952	-2.271297
97	6	0	2.420410	4.698191	-1.910011
98	6	0	3.321245	5.755093	-1.772971
99	6	0	3.486057	6.384222	-0.545548
100	6	0	2.726584	5.960230	0.543775
101	6	0	1.823033	4.912637	0.407157

102	1	0	2.308308	4.226628	-2.879868
103	1	0	3.895500	6.079277	-2.635514
104	1	0	4.192230	7.201489	-0.437825
105	1	0	2.830977	6.453618	1.505436
106	1	0	1.209575	4.616517	1.251348
107	6	0	2.034113	1.656116	-2.481483
108	6	0	2.302271	1.050144	-3.702259
109	6	0	-0.093825	2.409717	-3.308501
110	6	0	0.177986	1.806157	-4.537787
111	6	0	1.378676	1.138776	-4.743640
112	1	0	3.236599	0.512163	-3.833506
113	1	0	2.779467	1.588176	-1.692576
114	1	0	1.585530	0.670123	-5.700918
115	1	0	-0.561307	1.857386	-5.331320
116	1	0	-1.034745	2.931203	-3.170603
117	6	0	-1.829826	2.843390	-0.390420
118	6	0	-3.140053	3.310002	-0.398622
119	6	0	-3.430201	4.590465	-0.865864
120	6	0	-1.080474	4.935805	-1.303639
121	6	0	-2.395111	5.400280	-1.319331
122	1	0	-1.604733	1.852378	-0.008300
123	1	0	-3.939674	2.671203	-0.032580
124	1	0	-4.453135	4.953682	-0.869573
125	1	0	-2.603984	6.400924	-1.685039
126	1	0	-0.283076	5.579672	-1.661105
127	6	0	2.340370	-1.796839	2.995980
128	6	0	3.228558	-1.762648	1.749267
129	6	0	2.590373	-3.092059	3.783486
130	1	0	2.583631	-0.938628	3.634933
131	6	0	4.702304	-1.910466	2.130658
132	1	0	2.916810	-2.580389	1.088453
133	1	0	3.084345	-0.841157	1.182303
134	6	0	4.066981	-3.265672	4.144214
135	1	0	2.260094	-3.929175	3.153349
136	1	0	1.967499	-3.098370	4.684645
137	6	0	4.952632	-3.207267	2.898661
138	1	0	5.317634	-1.863781	1.226442
139	1	0	5.005272	-1.058444	2.757586
140	1	0	4.208186	-4.212177	4.676460
141	1	0	4.367183	-2.465635	4.835459
142	1	0	6.008199	-3.294675	3.177385
143	1	0	4.718978	-4.060655	2.247321

G1-II-*re*-TS2-a

Zero-point correction= 1.23110 (a.u.)

Thermal correction to Gibbs Free Energy= 1.15864 (a.u.)

Sum of electronic and zero-point Energies= -3123.25520 (a.u.)

Sum of electronic and thermal Free Energies= -3123.32766 (a.u.)

Standard orientation:

Center Number	Atomic Number	Atomic Type	Coordinates (Angstroms) X	Y	Z
1	6	0	-5.120072	-1.268458	1.451134
2	1	0	-4.490751	-2.090334	1.096505
3	6	0	-1.353577	0.868659	1.813444
4	1	0	-1.875169	0.544935	2.702553
5	1	0	-0.984938	1.876278	1.681420
6	7	0	-0.682913	-0.099060	1.155265
7	8	0	0.049796	0.233405	0.170952
8	8	0	-0.839503	-1.308508	1.452113
9	8	0	-1.478518	3.556578	-2.061255
10	8	0	-3.095680	-0.880998	-0.567971
11	7	0	-4.407578	-0.048911	1.124787
12	7	0	-2.206503	1.825995	-0.852926
13	8	0	-1.286940	3.685027	0.187433
14	6	0	-1.093490	4.945344	-2.266417
15	6	0	-2.888930	1.448247	0.189426
16	6	0	-3.429400	0.028247	0.174043
17	6	0	-3.733081	2.160179	1.196406
18	6	0	-4.637928	1.205629	1.703214
19	6	0	-3.805802	3.468978	1.659539
20	1	0	-3.103711	4.212056	1.306116
21	6	0	-1.643176	3.085015	-0.816845
22	6	0	-4.777403	3.801587	2.606738
23	1	0	-4.837763	4.821647	2.970688
24	6	0	-5.602301	1.519352	2.646148
25	1	0	-6.299872	0.770454	3.006157
26	6	0	-5.660020	2.840999	3.093265
27	1	0	-6.407556	3.119128	3.829609
28	6	0	-0.835928	5.003745	-3.767332
29	6	0	-2.271383	5.833582	-1.880654
30	6	0	0.174980	5.322290	-1.498422
31	1	0	-0.041944	5.637198	-0.478234
32	1	0	0.687688	6.135153	-2.023120
33	1	0	0.846319	4.462607	-1.449883
34	1	0	-0.644964	6.034983	-4.076224

35	1	0	-1.700085	4.625151	-4.319462
36	1	0	0.038062	4.398508	-4.028962
37	1	0	-2.024391	6.882003	-2.073065
38	1	0	-2.499493	5.726787	-0.817197
39	1	0	-3.158938	5.572605	-2.464137
40	6	0	2.412538	-4.839198	-1.447261
41	6	0	3.462315	-3.812841	-1.862872
42	6	0	2.789434	-2.496394	-2.277625
43	7	0	1.582789	-2.272885	-1.466031
44	6	0	1.727524	-4.379632	-0.151555
45	6	0	1.585498	-2.849672	-0.114315
46	1	0	2.866866	-5.822766	-1.302642
47	1	0	1.674610	-4.944891	-2.253859
48	1	0	4.066041	-4.164325	-2.703979
49	1	0	4.146596	-3.635837	-1.026205
50	1	0	3.508305	-1.673482	-2.182345
51	1	0	2.466668	-2.552132	-3.321434
52	1	0	0.746269	-4.851378	-0.048073
53	1	0	2.328082	-4.673759	0.711219
54	1	0	0.655663	-2.527451	0.367334
55	6	0	0.520369	-1.543350	-1.882262
56	7	0	-0.718868	-1.916519	-1.561152
57	7	0	0.690362	-0.442842	-2.623920
58	6	0	1.872848	0.422645	-2.644346
59	1	0	-1.409652	-1.182010	-1.394114
60	6	0	2.712296	-2.192238	0.725025
61	8	0	3.490856	-2.859394	1.385855
62	7	0	2.742533	-0.829629	0.680745
63	1	0	1.895006	-0.353518	0.363501
64	6	0	2.545463	0.384287	-4.022397
65	6	0	3.811415	1.240040	-4.042609
66	6	0	3.503674	2.676194	-3.623604
67	6	0	1.520431	1.862725	-2.260841
68	1	0	1.827223	0.767794	-4.760560
69	1	0	2.771728	-0.647196	-4.305717
70	1	0	4.544388	0.810212	-3.344474
71	1	0	4.268047	1.210861	-5.037189
72	1	0	2.557181	0.043531	-1.877802
73	1	0	4.423881	3.268000	-3.585139
74	1	0	1.042057	1.871347	-1.274399
75	1	0	0.791981	2.264242	-2.980963
76	6	0	2.800433	2.706279	-2.267035
77	1	0	2.855966	3.140293	-4.380562
78	1	0	2.582692	3.738427	-1.983416

79	1	0	3.478791	2.318037	-1.493889
80	1	0	-0.166876	-0.037528	-2.985874
81	6	0	3.492620	-0.248733	2.980169
82	6	0	3.742528	-0.090406	1.468083
83	6	0	5.116988	-0.591457	0.980977
84	6	0	3.584771	1.423827	1.200289
85	6	0	4.411520	0.285154	3.890357
86	6	0	4.168020	0.241416	5.258115
87	6	0	2.986203	-0.317610	5.740129
88	6	0	2.053670	-0.822079	4.840366
89	6	0	2.302288	-0.787594	3.469394
90	1	0	5.323034	0.748396	3.521071
91	1	0	4.897898	0.654590	5.947470
92	1	0	2.790229	-0.347657	6.807356
93	1	0	1.120293	-1.243757	5.200489
94	1	0	1.548124	-1.168350	2.785841
95	6	0	2.311782	2.000410	1.230477
96	6	0	2.135933	3.368941	1.047734
97	6	0	4.689400	2.267668	1.047416
98	6	0	4.518063	3.642053	0.889016
99	6	0	3.241976	4.197558	0.874603
100	1	0	1.127257	3.775095	1.038777
101	1	0	1.435615	1.383176	1.390558
102	1	0	3.108795	5.266289	0.733468
103	1	0	5.391437	4.276053	0.769339
104	1	0	5.692885	1.855440	1.044885
105	6	0	5.444550	-0.360204	-0.360349
106	6	0	6.637126	-0.826040	-0.901664
107	6	0	7.530446	-1.538268	-0.102879
108	6	0	6.007802	-1.320240	1.763973
109	6	0	7.208711	-1.783612	1.227201
110	1	0	4.751578	0.202658	-0.983513
111	1	0	6.871218	-0.629479	-1.943934
112	1	0	8.464381	-1.905028	-0.517280
113	1	0	7.885478	-2.355739	1.854308
114	1	0	5.752724	-1.561544	2.789235
115	6	0	-1.211188	-3.298611	-1.524116
116	6	0	-1.753014	-3.734321	-0.158411
117	6	0	-2.302659	-3.450394	-2.595337
118	1	0	-0.362987	-3.940198	-1.795298
119	6	0	-2.276591	-5.171329	-0.221743
120	1	0	-2.557714	-3.046709	0.119464
121	1	0	-0.989696	-3.635035	0.615100
122	6	0	-2.845328	-4.879264	-2.650312

123	1	0	-3.114327	-2.753885	-2.346494
124	1	0	-1.903723	-3.151966	-3.570795
125	6	0	-3.365784	-5.323286	-1.283457
126	1	0	-2.653811	-5.468051	0.762304
127	1	0	-1.447589	-5.855114	-0.458405
128	1	0	-3.635125	-4.946895	-3.405738
129	1	0	-2.042942	-5.559468	-2.968985
130	1	0	-3.719789	-6.358670	-1.327219
131	1	0	-4.227013	-4.699772	-1.006646
132	1	0	-5.194981	-1.348504	2.540373
133	6	0	-6.491159	-1.338699	0.813309
134	6	0	-7.622681	-1.628322	1.573208
135	6	0	-6.625216	-1.126961	-0.561298
136	6	0	-8.877024	-1.706273	0.969682
137	1	0	-7.522530	-1.795030	2.643361
138	6	0	-7.875891	-1.199986	-1.163410
139	1	0	-5.741303	-0.906602	-1.155173
140	6	0	-9.005393	-1.490564	-0.398477
141	1	0	-9.752597	-1.931668	1.570498
142	1	0	-7.971161	-1.032316	-2.231728
143	1	0	-9.981672	-1.547905	-0.869458

G1-II-*si*-TS2-a

Zero-point correction= 1.22980 (a.u.)

Thermal correction to Gibbs Free Energy= 1.15816 (a.u.)

Sum of electronic and zero-point Energies= -3123.25322 (a.u.)

Sum of electronic and thermal Free Energies= -3123.32486 (a.u.)

Standard orientation:

Center Number	Atomic Number	Atomic Type	Coordinates (Angstroms) X	Y	Z
1	6	0	-4.789310	-1.115378	-0.524472
2	1	0	-4.161581	-1.737647	0.122764
3	8	0	-0.659635	4.366926	1.196938
4	8	0	-2.598922	-0.169999	1.110125
5	7	0	-4.163894	0.186881	-0.541419
6	7	0	-1.903760	2.594076	0.658971
7	8	0	-1.261418	4.131798	-0.968288
8	6	0	0.322533	5.402531	0.914880
9	6	0	-2.719171	1.968794	-0.125028
10	6	0	-3.109546	0.543061	0.259623
11	6	0	-3.685021	2.352512	-1.192513

12	6	0	-4.540050	1.249115	-1.377108
13	6	0	-3.926503	3.518365	-1.912445
14	1	0	-3.278089	4.375118	-1.788678
15	6	0	-1.286587	3.750041	0.188915
16	6	0	-4.996181	3.552451	-2.809572
17	1	0	-5.186489	4.458273	-3.375423
18	6	0	-5.599293	1.264527	-2.269938
19	1	0	-6.249103	0.403770	-2.388530
20	6	0	-5.815424	2.441071	-2.989951
21	1	0	-6.639656	2.486536	-3.694798
22	6	0	0.835049	5.780651	2.300030
23	6	0	-0.333900	6.611723	0.252447
24	6	0	1.447787	4.812243	0.070106
25	1	0	1.078889	4.467489	-0.896093
26	1	0	2.221662	5.570376	-0.089966
27	1	0	1.899073	3.959165	0.587623
28	1	0	1.580592	6.576653	2.218516
29	1	0	0.014547	6.134132	2.931689
30	1	0	1.301272	4.918794	2.785713
31	1	0	0.372869	7.447155	0.244491
32	1	0	-0.632514	6.391304	-0.771519
33	1	0	-1.216281	6.917910	0.823198
34	1	0	-4.760363	-1.545401	-1.533503
35	6	0	-6.214140	-1.107107	-0.008979
36	6	0	-7.140484	-2.009381	-0.533029
37	6	0	-6.607273	-0.236167	1.007277
38	6	0	-8.442201	-2.050368	-0.040829
39	1	0	-6.839889	-2.682294	-1.333009
40	6	0	-7.909748	-0.273197	1.496177
41	1	0	-5.892939	0.476318	1.411354
42	6	0	-8.829500	-1.181351	0.975501
43	1	0	-9.155050	-2.755612	-0.456560
44	1	0	-8.208006	0.409987	2.285195
45	1	0	-9.845305	-1.207297	1.356750
46	6	0	-1.200637	1.035105	-1.776354
47	1	0	-1.676500	0.204470	-2.276445
48	1	0	-1.175915	2.041436	-2.165451
49	7	0	-0.240941	0.730879	-0.894738
50	8	0	0.432917	1.636397	-0.355072
51	8	0	-0.056527	-0.494313	-0.554582
52	6	0	2.122732	-5.306621	1.406629
53	6	0	2.873824	-4.419082	2.397804
54	6	0	2.132069	-3.082210	2.612734
55	7	0	1.208441	-2.830386	1.494932

56	6	0	2.099726	-4.630185	0.031645
57	6	0	1.734619	-3.133073	0.158033
58	1	0	2.603157	-6.285654	1.329383
59	1	0	1.098326	-5.479479	1.761978
60	1	0	2.986894	-4.916299	3.364820
61	1	0	3.871246	-4.219201	2.000559
62	1	0	2.844452	-2.254615	2.714518
63	1	0	1.522631	-3.119364	3.516831
64	1	0	1.397835	-5.135987	-0.638655
65	1	0	3.093076	-4.703632	-0.417579
66	1	0	0.973269	-2.828908	-0.560404
67	6	0	-0.021472	-2.318288	1.694516
68	7	0	-1.073203	-2.610889	0.927656
69	7	0	-0.235597	-1.500231	2.739502
70	6	0	0.638922	-0.370065	3.113240
71	1	0	-1.741356	-1.841802	0.846701
72	6	0	2.981347	-2.245933	-0.095661
73	8	0	4.107856	-2.635918	0.182870
74	7	0	2.700901	-1.073079	-0.700972
75	1	0	1.723032	-0.741948	-0.669912
76	6	0	1.070315	-0.441055	4.577735
77	6	0	1.901228	0.791763	4.944995
78	6	0	1.124411	2.084777	4.685416
79	6	0	-0.145023	0.910376	2.835597
80	1	0	0.165674	-0.478384	5.201183
81	1	0	1.637213	-1.355742	4.776605
82	1	0	2.818010	0.789698	4.340082
83	1	0	2.210977	0.731956	5.993774
84	1	0	1.518319	-0.400657	2.456573
85	1	0	1.748620	2.954130	4.924528
86	1	0	-0.405786	0.953867	1.778233
87	1	0	-1.088243	0.864744	3.402474
88	6	0	0.626629	2.159419	3.240822
89	1	0	0.261534	2.123229	5.365013
90	1	0	-0.016946	3.028584	3.083806
91	1	0	1.474578	2.274076	2.553313
92	1	0	-1.220669	-1.297937	2.882981
93	6	0	4.777574	-0.744721	-2.024903
94	6	0	3.744828	-0.109988	-1.071650
95	6	0	4.357639	0.442618	0.227246
96	6	0	3.079850	0.984240	-1.933299
97	6	0	5.962216	-0.057550	-2.306931
98	6	0	6.863594	-0.542226	-3.248728
99	6	0	6.590089	-1.719978	-3.939424

100	6	0	5.404531	-2.399681	-3.680553
101	6	0	4.505577	-1.914347	-2.734179
102	1	0	6.180334	0.870433	-1.785898
103	1	0	7.778709	0.007320	-3.447036
104	1	0	7.291196	-2.099942	-4.675767
105	1	0	5.172985	-3.314551	-4.217533
106	1	0	3.578956	-2.450140	-2.557107
107	6	0	2.076129	0.630378	-2.837665
108	6	0	1.505738	1.582097	-3.676453
109	6	0	3.534420	2.304624	-1.923807
110	6	0	2.967519	3.255937	-2.767547
111	6	0	1.942882	2.902680	-3.640318
112	1	0	0.710027	1.287864	-4.354174
113	1	0	1.716931	-0.394335	-2.873411
114	1	0	1.492246	3.647834	-4.288159
115	1	0	3.324775	4.280831	-2.732396
116	1	0	4.326091	2.603342	-1.244533
117	6	0	3.629038	1.378841	0.971611
118	6	0	4.144937	1.886720	2.161578
119	6	0	5.370862	1.435902	2.648275
120	6	0	5.567278	-0.030071	0.739420
121	6	0	6.070054	0.463249	1.942019
122	1	0	2.653662	1.703521	0.608745
123	1	0	3.588175	2.639392	2.713235
124	1	0	5.769485	1.834272	3.576428
125	1	0	7.013165	0.083448	2.322894
126	1	0	6.111965	-0.798418	0.203568
127	6	0	-1.307707	-3.769539	0.076223
128	6	0	-1.564240	-3.323190	-1.366574
129	6	0	-2.493330	-4.580295	0.613739
130	1	0	-0.422100	-4.409884	0.115496
131	6	0	-1.876674	-4.516116	-2.270446
132	1	0	-2.423601	-2.636227	-1.354330
133	1	0	-0.720215	-2.735790	-1.740186
134	6	0	-2.795976	-5.775806	-0.291837
135	1	0	-3.374141	-3.924147	0.663543
136	1	0	-2.276370	-4.904500	1.637047
137	6	0	-3.056352	-5.326026	-1.730226
138	1	0	-2.081047	-4.167423	-3.287589
139	1	0	-0.992072	-5.164922	-2.332030
140	1	0	-3.653311	-6.331738	0.100179
141	1	0	-1.939113	-6.463680	-0.277933
142	1	0	-3.251180	-6.191994	-2.370937
143	1	0	-3.961620	-4.702685	-1.752547

R1b

Zero-point correction= 0.31208 (a.u.)
Thermal correction to Gibbs Free Energy= 0.27428 (a.u.)
Sum of electronic and zero-point Energies= -1030.05894 (a.u.)
Sum of electronic and thermal Free Energies= -1030.09675 (a.u.)

Standard orientation:

Center Number	Atomic Number	Atomic Type	Coordinates (Angstroms) X	Y	Z
1	6	0	-1.070189	1.987982	-0.565351
2	6	0	-0.461065	0.744564	-0.514278
3	6	0	0.845689	0.586669	-0.019667
4	6	0	1.567931	1.684111	0.429725
5	6	0	0.969760	2.942475	0.375239
6	6	0	-0.328581	3.083133	-0.114970
7	1	0	-2.085003	2.105676	-0.929169
8	1	0	2.571502	1.568418	0.826778
9	1	0	1.513673	3.814646	0.720075
10	1	0	-0.781610	4.069165	-0.144799
11	7	0	-0.991580	-0.488574	-0.917797
12	6	0	-0.087433	-1.508669	-0.714259
13	8	0	-0.250718	-2.679904	-0.957875
14	6	0	-2.309102	-0.681696	-1.491324
15	1	0	-2.325155	-1.715697	-1.848878
16	6	0	1.157959	-0.845433	-0.112127
17	7	0	2.171917	-1.543271	0.188614
18	6	0	3.286340	-0.918544	0.774015
19	8	0	3.450151	-0.807744	1.963156
20	8	0	4.158410	-0.547930	-0.164217
21	6	0	5.393389	0.004517	0.333103
22	6	0	6.230156	0.380185	-0.866798
23	1	0	7.180269	0.803674	-0.531610
24	1	0	6.440084	-0.496911	-1.483183
25	1	0	5.715476	1.123963	-1.479725
26	1	0	5.166461	0.871411	0.961473
27	1	0	5.883612	-0.745638	0.960251
28	1	0	-2.423923	-0.021440	-2.358448
29	6	0	-3.427709	-0.442876	-0.497702
30	6	0	-4.540147	0.317912	-0.855212
31	6	0	-3.364782	-1.002837	0.779907
32	6	0	-5.580796	0.514967	0.050563

33	1	0	-4.593347	0.758085	-1.848341
34	6	0	-4.400519	-0.803880	1.685878
35	1	0	-2.499667	-1.596878	1.063797
36	6	0	-5.511864	-0.044212	1.322668
37	1	0	-6.442016	1.109770	-0.237367
38	1	0	-4.341018	-1.242784	2.676847
39	1	0	-6.318670	0.111873	2.031591

G1-1b-I-*re*-IM2

Zero-point correction= 1.17495 (a.u.)

Thermal correction to Gibbs Free Energy= 1.10360 (a.u.)

Sum of electronic and zero-point Energies= -3044.71820 (a.u.)

Sum of electronic and thermal Free Energies= -3044.78955 (a.u.)

Standard orientation:

Center Number	Atomic Number	Atomic Type	Coordinates (Angstroms) X	Y	Z
1	6	0	0.372334	2.397198	-4.559471
2	6	0	-0.371831	3.265659	-3.541688
3	6	0	-0.689872	2.494158	-2.258860
4	7	0	0.554014	1.920721	-1.712194
5	6	0	1.599297	1.723992	-3.935089
6	6	0	1.161727	0.988501	-2.668614
7	1	0	0.666429	2.992887	-5.428954
8	1	0	-0.307208	1.615140	-4.923267
9	1	0	-1.307945	3.637071	-3.971771
10	1	0	0.237673	4.143320	-3.288271
11	1	0	-1.157299	3.131414	-1.511575
12	1	0	-1.380145	1.666198	-2.437663
13	1	0	2.057877	1.016072	-4.631578
14	1	0	2.363943	2.469938	-3.686084
15	1	0	0.334188	0.318278	-2.938701
16	6	0	1.212475	2.531271	-0.691679
17	7	0	0.475899	3.010666	0.297695
18	7	0	2.556909	2.614125	-0.734409
19	6	0	3.478874	2.686283	0.406972
20	1	0	-0.509503	2.603160	0.383109
21	6	0	2.270729	0.093408	-2.076101
22	8	0	3.446568	0.320562	-2.334233
23	7	0	1.764756	-0.919424	-1.338143
24	1	0	0.752883	-0.888214	-1.220744
25	6	0	3.187424	1.620290	1.462313

26	6	0	4.188308	1.718816	2.613432
27	6	0	5.619855	1.566173	2.097202
28	6	0	4.902617	2.527631	-0.131860
29	1	0	3.273872	0.637038	0.983718
30	1	0	2.156542	1.691085	1.823708
31	1	0	4.079558	2.692954	3.112101
32	1	0	3.962470	0.949893	3.358429
33	1	0	3.404966	3.683185	0.863811
34	1	0	6.338936	1.662716	2.917382
35	1	0	5.102485	3.306730	-0.876712
36	1	0	4.972018	1.559623	-0.647300
37	6	0	5.923027	2.596370	1.006436
38	1	0	5.740054	0.553915	1.686115
39	1	0	6.930776	2.448309	0.605451
40	1	0	5.901910	3.602424	1.447159
41	1	0	2.989097	2.291363	-1.593580
42	6	0	3.832861	-2.314214	-1.178668
43	6	0	2.396349	-2.077802	-0.670633
44	6	0	2.361363	-1.850255	0.858537
45	6	0	1.513747	-3.299489	-1.011648
46	6	0	4.150200	-3.365381	-2.036117
47	6	0	5.464834	-3.567293	-2.463744
48	6	0	6.479568	-2.724370	-2.034459
49	6	0	6.173810	-1.668635	-1.173861
50	6	0	4.867649	-1.470891	-0.755026
51	1	0	3.379075	-4.046377	-2.377818
52	1	0	5.686596	-4.395316	-3.129720
53	1	0	7.502021	-2.884853	-2.362288
54	1	0	6.955072	-0.996343	-0.831382
55	1	0	4.637538	-0.643849	-0.091375
56	6	0	0.833341	-3.369213	-2.231785
57	6	0	0.076429	-4.490623	-2.564144
58	6	0	1.421136	-4.383178	-0.133689
59	6	0	0.665611	-5.505109	-0.466009
60	6	0	-0.009788	-5.564607	-1.682508
61	1	0	-0.444526	-4.521837	-3.516073
62	1	0	0.900515	-2.543833	-2.933403
63	1	0	-0.600207	-6.438518	-1.940115
64	1	0	0.605075	-6.332066	0.234786
65	1	0	1.931451	-4.351537	0.822907
66	6	0	1.286211	-1.167752	1.436187
67	6	0	1.237227	-0.951270	2.811516
68	6	0	2.243297	-1.442469	3.638109
69	6	0	3.354485	-2.362934	1.700679

70	6	0	3.296704	-2.160193	3.077613
71	1	0	0.474132	-0.751550	0.843235
72	1	0	0.403114	-0.381328	3.209711
73	1	0	2.206496	-1.271282	4.709811
74	1	0	4.083583	-2.561151	3.709455
75	1	0	4.186967	-2.919502	1.282986
76	6	0	0.820151	4.061441	1.248894
77	6	0	1.155145	5.381508	0.549233
78	6	0	-0.361062	4.229829	2.206700
79	1	0	1.682922	3.761923	1.859473
80	6	0	1.452274	6.474295	1.578789
81	1	0	0.291693	5.668721	-0.066259
82	1	0	2.005881	5.245911	-0.129777
83	6	0	-0.059526	5.317853	3.236462
84	1	0	-1.253500	4.491921	1.624765
85	1	0	-0.569385	3.263670	2.679905
86	6	0	0.286205	6.644244	2.555165
87	1	0	1.669631	7.418838	1.069762
88	1	0	2.356844	6.202852	2.141453
89	1	0	-0.915956	5.443768	3.906411
90	1	0	0.787345	5.002875	3.861915
91	1	0	0.526316	7.407032	3.303415
92	1	0	-0.593091	7.003438	2.003340
93	6	0	-3.368095	0.803590	1.580662
94	1	0	-3.449657	0.004125	2.300463
95	1	0	-4.206605	1.317150	1.138188
96	7	0	-2.167033	1.260128	1.303587
97	8	0	-2.037601	2.242448	0.474913
98	8	0	-1.110136	0.759997	1.817892
99	6	0	-4.903914	-3.147220	0.434693
100	6	0	-4.134693	-2.125796	-0.098799
101	6	0	-4.721309	-0.977470	-0.656447
102	6	0	-6.102111	-0.844315	-0.701556
103	6	0	-6.888815	-1.866230	-0.169101
104	6	0	-6.292472	-2.994873	0.391082
105	1	0	-4.447417	-4.024438	0.878829
106	1	0	-6.566104	0.030122	-1.146393
107	1	0	-7.969558	-1.781463	-0.191768
108	1	0	-6.919093	-3.779104	0.804024
109	7	0	-2.735078	-2.062819	-0.188274
110	6	0	-2.335006	-0.916307	-0.822135
111	8	0	-1.197629	-0.591853	-1.108805
112	6	0	-1.843596	-3.140017	0.201516
113	1	0	-0.840845	-2.830647	-0.107139

114	6	0	-3.613715	-0.144672	-1.151880
115	7	0	-3.552814	0.942302	-1.801569
116	6	0	-4.717238	1.671254	-2.065891
117	8	0	-5.171152	1.847626	-3.169573
118	8	0	-5.201982	2.213546	-0.942718
119	6	0	-6.333430	3.084608	-1.106327
120	6	0	-6.760701	3.509981	0.279003
121	1	0	-7.061867	2.642852	0.872639
122	1	0	-5.940999	4.015905	0.794690
123	1	0	-7.608288	4.196613	0.211896
124	1	0	-6.034466	3.935423	-1.725985
125	1	0	-7.126487	2.546741	-1.635409
126	1	0	-2.103805	-4.038939	-0.368638
127	6	0	-1.873195	-3.432952	1.684002
128	6	0	-2.010533	-4.745241	2.135744
129	6	0	-1.737409	-2.393749	2.605844
130	6	0	-2.015767	-5.023553	3.500842
131	1	0	-2.116709	-5.551408	1.412546
132	6	0	-1.746063	-2.674197	3.969749
133	1	0	-1.605029	-1.369246	2.254482
134	6	0	-1.886562	-3.985871	4.420045
135	1	0	-2.124835	-6.047581	3.844735
136	1	0	-1.636380	-1.863097	4.683904
137	1	0	-1.893516	-4.198870	5.484599

G1-1b-I-*si*-IM2

Zero-point correction= 1.17407 (a.u.)

Thermal correction to Gibbs Free Energy= 1.10221 (a.u.)

Sum of electronic and zero-point Energies= -3044.72157 (a.u.)

Sum of electronic and thermal Free Energies= -3044.79343 (a.u.)

Standard orientation:

Center Number	Atomic Number	Atomic Type	Coordinates (Angstroms) X	Y	Z
1	6	0	-0.621047	3.871359	-2.250318
2	6	0	0.471456	3.743593	-3.320057
3	6	0	1.776966	4.431464	-2.916172
4	6	0	1.540158	5.889802	-2.519625
5	6	0	-0.821177	5.333304	-1.828508
6	1	0	0.085196	4.199138	-4.240354
7	1	0	0.634209	2.678421	-3.520140
8	1	0	2.228884	3.880909	-2.082043

9	1	0	2.488993	4.372002	-3.746283
10	1	0	-1.550764	3.491551	-2.687750
11	1	0	2.478959	6.354577	-2.199300
12	1	0	-1.241998	5.871506	-2.688021
13	6	0	0.493172	5.993610	-1.408463
14	1	0	1.188789	6.453913	-3.395184
15	1	0	0.313192	7.040779	-1.143647
16	1	0	0.877332	5.499832	-0.505358
17	6	0	0.594969	3.226033	3.729870
18	6	0	1.282891	3.796275	2.485420
19	6	0	1.254414	2.819321	1.307004
20	7	0	-0.122771	2.361238	1.066562
21	6	0	-0.804453	2.688835	3.408470
22	6	0	-0.679918	1.702218	2.250900
23	1	0	0.542810	3.986157	4.515200
24	1	0	1.200514	2.399922	4.127256
25	1	0	2.327291	4.044060	2.704194
26	1	0	0.786483	4.728820	2.188188
27	1	0	1.649989	3.278351	0.402300
28	1	0	1.859471	1.929562	1.504831
29	1	0	-1.243306	2.183299	4.272314
30	1	0	-1.481504	3.509592	3.138395
31	1	0	0.085282	0.961109	2.527552
32	6	0	-0.856176	2.832406	0.024195
33	7	0	-0.219657	3.034023	-1.118173
34	7	0	-2.174274	3.056724	0.198413
35	6	0	-3.216368	2.942019	-0.832677
36	1	0	0.652745	2.418346	-1.242028
37	6	0	-1.947007	0.853854	2.011913
38	8	0	-2.968989	1.105176	2.634479
39	7	0	-1.746296	-0.163919	1.135634
40	1	0	-0.779241	-0.231059	0.814113
41	6	0	-3.098443	1.641265	-1.628448
42	6	0	-4.225661	1.525614	-2.654179
43	6	0	-5.597465	1.629882	-1.984793
44	6	0	-4.574722	3.028955	-0.134882
45	1	0	-3.148153	0.808865	-0.919388
46	1	0	-2.120330	1.555774	-2.114580
47	1	0	-4.122537	2.323823	-3.403236
48	1	0	-4.128778	0.574346	-3.186991
49	1	0	-3.140743	3.800663	-1.514171
50	1	0	-6.392350	1.600767	-2.737498
51	1	0	-4.646171	3.977720	0.410023
52	1	0	-4.641390	2.221970	0.608480

53	6	0	-5.712174	2.909246	-1.151950
54	1	0	-5.746342	0.755565	-1.336818
55	1	0	-6.674789	2.945725	-0.631817
56	1	0	-5.682441	3.778571	-1.822665
57	1	0	-1.562129	5.393788	-1.022330
58	1	0	-2.512356	3.016151	1.152975
59	6	0	-3.807504	-1.471303	1.722090
60	6	0	-2.407202	-1.494098	1.091369
61	6	0	-2.551981	-1.923806	-0.384495
62	6	0	-1.439088	-2.427836	1.848490
63	6	0	-4.196079	-2.336983	2.739301
64	6	0	-5.513146	-2.332163	3.210373
65	6	0	-6.452623	-1.468011	2.667256
66	6	0	-6.073699	-0.607521	1.634073
67	6	0	-4.770529	-0.622328	1.166395
68	1	0	-3.482049	-3.028939	3.171732
69	1	0	-5.796081	-3.014509	4.006028
70	1	0	-7.473564	-1.464615	3.036006
71	1	0	-6.797694	0.072302	1.193276
72	1	0	-4.478941	0.036247	0.355407
73	6	0	-1.065751	-2.081608	3.155540
74	6	0	-0.139966	-2.841543	3.856611
75	6	0	-0.863453	-3.551455	1.263638
76	6	0	0.074453	-4.312315	1.965823
77	6	0	0.441200	-3.961665	3.258323
78	1	0	0.133382	-2.559634	4.868962
79	1	0	-1.516166	-1.210184	3.626977
80	1	0	1.174226	-4.550954	3.800785
81	1	0	0.526369	-5.173745	1.482539
82	1	0	-1.111726	-3.822205	0.244394
83	6	0	-1.737646	-1.387408	-1.381313
84	6	0	-1.857142	-1.809172	-2.705434
85	6	0	-2.787430	-2.784200	-3.050891
86	6	0	-3.473868	-2.916086	-0.737958
87	6	0	-3.590940	-3.343293	-2.057338
88	1	0	-0.973930	-0.646946	-1.174689
89	1	0	-1.192064	-1.368643	-3.443094
90	1	0	-2.886323	-3.110256	-4.082505
91	1	0	-4.320400	-4.107700	-2.308925
92	1	0	-4.114102	-3.351873	0.023652
93	6	0	2.831398	-0.162317	-2.396514
94	1	0	2.655268	-1.014474	-3.035080
95	1	0	3.804111	0.175170	-2.073931
96	7	0	1.789671	0.567606	-2.072059

97	8	0	1.961726	1.613055	-1.305856
98	8	0	0.615099	0.298743	-2.460359
99	8	0	0.012982	-4.313509	-1.792608
100	8	0	1.196775	-0.387347	0.943403
101	7	0	3.410074	-1.036745	1.048325
102	7	0	0.990963	-2.454635	-1.033543
103	8	0	1.468826	-3.255141	-3.154988
104	1	0	4.340194	-0.895054	2.900642
105	6	0	-0.301370	-5.239368	-2.846679
106	6	0	2.110386	-2.131924	-0.533434
107	6	0	2.131267	-1.060873	0.555753
108	6	0	3.492596	-2.636054	-0.622364
109	6	0	4.227163	-1.948729	0.361340
110	6	0	4.116571	-3.586674	-1.419094
111	1	0	3.564509	-4.099748	-2.198046
112	6	0	0.899076	-3.360728	-2.090505
113	6	0	5.473057	-3.844990	-1.213927
114	1	0	5.978358	-4.581573	-1.828766
115	6	0	3.834590	-0.240433	2.181673
116	1	0	2.913603	0.119004	2.654164
117	6	0	5.575375	-2.186954	0.567770
118	1	0	6.138444	-1.635427	1.313104
119	6	0	6.186091	-3.152711	-0.236509
120	1	0	7.242735	-3.359198	-0.098664
121	6	0	4.728244	0.923670	1.804464
122	6	0	5.804331	1.282889	2.616260
123	6	0	4.451456	1.668690	0.656235
124	6	0	6.592315	2.385786	2.290684
125	1	0	6.028423	0.700058	3.506994
126	6	0	5.239520	2.770019	0.334272
127	1	0	3.610334	1.406244	0.015358
128	6	0	6.310238	3.132224	1.149482
129	1	0	7.430088	2.657355	2.925517
130	1	0	5.011773	3.342533	-0.560224
131	1	0	6.924738	3.990033	0.894429
132	6	0	0.744574	-6.330332	-2.948806
133	1	0	1.704286	-5.916738	-3.266291
134	1	0	0.871465	-6.831530	-1.985548
135	1	0	0.431269	-7.074674	-3.686011
136	1	0	-1.276592	-5.642600	-2.567788
137	1	0	-0.397499	-4.684826	-3.783236

G1-1b-I-*re*-TS2

Zero-point correction= 1.17616 (a.u.)
Thermal correction to Gibbs Free Energy= 1.10692 (a.u.)
Sum of electronic and zero-point Energies= 3044.71370 (a.u.)
Sum of electronic and thermal Free Energies= -3044.78294 (a.u.)

Standard orientation:

Center Number	Atomic Number	Atomic Type	Coordinates (Angstroms) X	Y	Z
1	6	0	0.296292	2.333968	-4.477506
2	6	0	-0.275563	3.313811	-3.450581
3	6	0	-0.581848	2.626453	-2.119327
4	7	0	0.636011	1.946607	-1.627980
5	6	0	1.487917	1.557359	-3.909084
6	6	0	1.077966	0.916408	-2.583372
7	1	0	0.587704	2.862648	-5.390136
8	1	0	-0.487357	1.618086	-4.757296
9	1	0	-1.200120	3.761944	-3.827736
10	1	0	0.438500	4.130505	-3.279192
11	1	0	-0.920950	3.344890	-1.376202
12	1	0	-1.369549	1.874087	-2.212361
13	1	0	1.813864	0.775524	-4.602021
14	1	0	2.348147	2.218103	-3.747480
15	1	0	0.170172	0.324724	-2.757842
16	6	0	1.379168	2.483131	-0.635778
17	7	0	0.724552	3.066692	0.369603
18	7	0	2.719123	2.424201	-0.688756
19	6	0	3.658798	2.444608	0.440091
20	1	0	-0.255267	2.759832	0.495693
21	6	0	2.153314	-0.051009	-2.045292
22	8	0	3.336250	0.134562	-2.310902
23	7	0	1.615936	-1.073027	-1.346796
24	1	0	0.606856	-1.009034	-1.214813
25	6	0	3.286450	1.439438	1.529463
26	6	0	4.319207	1.462067	2.655950
27	6	0	5.716748	1.158417	2.114784
28	6	0	5.053600	2.152596	-0.118071
29	1	0	3.253766	0.440216	1.077513
30	1	0	2.279097	1.633366	1.912660
31	1	0	4.319262	2.451985	3.135170
32	1	0	4.035089	0.734793	3.422559
33	1	0	3.679946	3.455825	0.870847
34	1	0	6.458329	1.198008	2.919606
35	1	0	5.309240	2.906051	-0.872393

36	1	0	5.026132	1.180985	-0.629501
37	6	0	6.096982	2.136166	1.000172
38	1	0	5.727790	0.131856	1.721941
39	1	0	7.076782	1.882528	0.583045
40	1	0	6.186297	3.145620	1.423949
41	1	0	3.108326	2.051274	-1.549567
42	6	0	3.642740	-2.534372	-1.197020
43	6	0	2.218850	-2.247141	-0.678637
44	6	0	2.206616	-2.002313	0.847644
45	6	0	1.289548	-3.437198	-1.003565
46	6	0	3.917461	-3.588237	-2.065728
47	6	0	5.220793	-3.828834	-2.507708
48	6	0	6.266813	-3.023364	-2.081250
49	6	0	6.004729	-1.968282	-1.205672
50	6	0	4.709586	-1.732657	-0.772653
51	1	0	3.121548	-4.241213	-2.404832
52	1	0	5.408895	-4.656548	-3.184304
53	1	0	7.279999	-3.213420	-2.421736
54	1	0	6.811440	-1.326270	-0.863622
55	1	0	4.512508	-0.908239	-0.095697
56	6	0	0.646221	-3.511732	-2.244487
57	6	0	-0.158059	-4.601446	-2.567351
58	6	0	1.107804	-4.481775	-0.093825
59	6	0	0.305054	-5.573780	-0.417741
60	6	0	-0.330924	-5.639005	-1.654271
61	1	0	-0.649361	-4.637104	-3.534725
62	1	0	0.781164	-2.713741	-2.967816
63	1	0	-0.957737	-6.489493	-1.904428
64	1	0	0.173834	-6.369444	0.309029
65	1	0	1.579755	-4.442517	0.881992
66	6	0	1.172857	-1.257111	1.422784
67	6	0	1.138565	-1.032028	2.797087
68	6	0	2.120372	-1.568794	3.624266
69	6	0	3.173878	-2.561744	1.689921
70	6	0	3.132938	-2.344632	3.065156
71	1	0	0.384018	-0.808246	0.823530
72	1	0	0.335293	-0.421125	3.198639
73	1	0	2.095853	-1.389848	4.695016
74	1	0	3.899529	-2.782291	3.697548
75	1	0	3.971559	-3.167812	1.273102
76	6	0	1.182303	4.151455	1.232773
77	6	0	1.701118	5.345558	0.427404
78	6	0	0.008498	4.570250	2.120901
79	1	0	1.982681	3.796340	1.898681

80	6	0	2.128461	6.477113	1.363122
81	1	0	0.890702	5.687270	-0.231316
82	1	0	2.533182	5.041194	-0.217811
83	6	0	0.425561	5.703055	3.059176
84	1	0	-0.819881	4.895917	1.477913
85	1	0	-0.350636	3.698996	2.679627
86	6	0	0.976483	6.898720	2.278067
87	1	0	2.486566	7.329908	0.777813
88	1	0	2.974686	6.137808	1.977338
89	1	0	-0.428078	6.007524	3.672590
90	1	0	1.196292	5.335469	3.750405
91	1	0	1.306623	7.683471	2.966800
92	1	0	0.172529	7.328676	1.665569
93	6	0	-3.377199	0.813471	1.302815
94	1	0	-3.447531	-0.062650	1.931646
95	1	0	-4.207383	1.482011	1.138767
96	7	0	-2.153625	1.381988	1.229349
97	8	0	-2.035952	2.494889	0.642634
98	8	0	-1.135942	0.786742	1.666444
99	6	0	-5.173202	-2.940015	0.250618
100	6	0	-4.324132	-1.915931	-0.138614
101	6	0	-4.807876	-0.662288	-0.558557
102	6	0	-6.177034	-0.444074	-0.628158
103	6	0	-7.044823	-1.472168	-0.253093
104	6	0	-6.547177	-2.696926	0.186967
105	1	0	-4.784653	-3.891669	0.597293
106	1	0	-6.563551	0.510456	-0.964479
107	1	0	-8.116568	-1.311929	-0.300993
108	1	0	-7.235834	-3.480960	0.485572
109	7	0	-2.924374	-1.951664	-0.196683
110	6	0	-2.428953	-0.775490	-0.697777
111	8	0	-1.260496	-0.527739	-0.950935
112	6	0	-2.114034	-3.115049	0.100749
113	1	0	-1.101553	-2.870850	-0.233703
114	6	0	-3.616286	0.154033	-0.910351
115	7	0	-3.420352	1.185440	-1.667709
116	6	0	-4.350180	2.177396	-1.865750
117	8	0	-4.413997	2.860248	-2.865831
118	8	0	-5.143012	2.397741	-0.785946
119	6	0	-6.037480	3.510927	-0.888715
120	6	0	-6.702392	3.672445	0.459798
121	1	0	-7.239660	2.762346	0.741014
122	1	0	-5.958226	3.887025	1.231117
123	1	0	-7.417630	4.498509	0.428068

124	1	0	-5.467810	4.400168	-1.172851
125	1	0	-6.766153	3.316180	-1.683133
126	1	0	-2.465182	-3.961685	-0.499801
127	6	0	-2.102951	-3.480562	1.567734
128	6	0	-2.280245	-4.806460	1.961670
129	6	0	-1.878606	-2.498931	2.535305
130	6	0	-2.235816	-5.154543	3.309788
131	1	0	-2.449967	-5.569372	1.204679
132	6	0	-1.839317	-2.847225	3.882533
133	1	0	-1.714703	-1.465720	2.228900
134	6	0	-2.019084	-4.172890	4.273054
135	1	0	-2.376747	-6.189376	3.606738
136	1	0	-1.662118	-2.079951	4.630461
137	1	0	-1.988505	-4.439851	5.324900

G1-1b-I-*si*-TS2

Zero-point correction= 1.17654 (a.u.)

Thermal correction to Gibbs Free Energy= 1.10693 (a.u.)

Sum of electronic and zero-point Energies= -3044.71598 (a.u.)

Sum of electronic and thermal Free Energies= -3044.78560 (a.u.)

Standard orientation:

Center Number	Atomic Number	Atomic Type	Coordinates (Angstroms) X	Y	Z
1	6	0	-1.189222	3.800745	-2.194530
2	6	0	-0.154793	3.748342	-3.325927
3	6	0	1.116108	4.538760	-3.002777
4	6	0	0.791015	5.977866	-2.600390
5	6	0	-1.471263	5.246194	-1.761460
6	1	0	-0.627517	4.165713	-4.222956
7	1	0	0.084052	2.698960	-3.534403
8	1	0	1.661021	4.037115	-2.193665
9	1	0	1.779073	4.524599	-3.873989
10	1	0	-2.113667	3.346000	-2.567968
11	1	0	1.708227	6.518900	-2.345408
12	1	0	-1.984083	5.745208	-2.593673
13	6	0	-0.187506	6.007517	-1.424986
14	1	0	0.339037	6.501762	-3.454289
15	1	0	-0.431816	7.038791	-1.150505
16	1	0	0.288803	5.550799	-0.546330
17	6	0	0.273645	3.302898	3.737309
18	6	0	0.838570	3.972502	2.479587

19	6	0	0.939796	3.001966	1.299777
20	7	0	-0.363583	2.351802	1.088445
21	6	0	-1.033273	2.552272	3.449782
22	6	0	-0.782840	1.602881	2.280304
23	1	0	0.122883	4.047248	4.524563
24	1	0	1.009708	2.581930	4.118168
25	1	0	1.833236	4.384215	2.681245
26	1	0	0.194317	4.813159	2.191101
27	1	0	1.243968	3.516275	0.388548
28	1	0	1.668010	2.205993	1.482332
29	1	0	-1.358164	1.980710	4.323816
30	1	0	-1.839525	3.255532	3.207881
31	1	0	0.101017	1.002705	2.527788
32	6	0	-1.202629	2.754334	0.101074
33	7	0	-0.659082	3.000604	-1.086875
34	7	0	-2.512493	2.886633	0.366565
35	6	0	-3.638918	2.720303	-0.565735
36	1	0	0.246861	2.511976	-1.239138
37	6	0	-1.923881	0.593876	2.020015
38	8	0	-3.065485	0.847568	2.390863
39	7	0	-1.471265	-0.522998	1.416369
40	1	0	-0.496733	-0.474045	1.112778
41	6	0	-3.543252	1.411800	-1.344321
42	6	0	-4.751821	1.235434	-2.262728
43	6	0	-6.052097	1.274288	-1.459055
44	6	0	-4.928814	2.753892	0.257445
45	1	0	-3.508022	0.592160	-0.614871
46	1	0	-2.608652	1.359939	-1.912512
47	1	0	-4.761418	2.038012	-3.014536
48	1	0	-4.658078	0.289273	-2.803959
49	1	0	-3.655221	3.570714	-1.260767
50	1	0	-6.917952	1.179214	-2.122365
51	1	0	-4.993477	3.701251	0.805402
52	1	0	-4.879159	1.944299	0.999004
53	6	0	-6.151549	2.565023	-0.642505
54	1	0	-6.073170	0.410836	-0.779902
55	1	0	-7.059317	2.565571	-0.031156
56	1	0	-6.230817	3.419800	-1.327933
57	1	0	-2.162898	5.257013	-0.910739
58	1	0	-2.770197	2.735527	1.336566
59	6	0	-3.260439	-2.121798	2.101564
60	6	0	-2.094178	-1.836746	1.137245
61	6	0	-2.528813	-1.896356	-0.343809
62	6	0	-0.954791	-2.868335	1.339241

63	6	0	-3.134877	-3.026691	3.154820
64	6	0	-4.209395	-3.287334	4.007236
65	6	0	-5.426914	-2.649545	3.813405
66	6	0	-5.565621	-1.743133	2.762057
67	6	0	-4.494395	-1.484145	1.919158
68	1	0	-2.197414	-3.546742	3.315199
69	1	0	-4.086832	-4.000303	4.816555
70	1	0	-6.265551	-2.856894	4.471119
71	1	0	-6.511531	-1.235475	2.599931
72	1	0	-4.607311	-0.770778	1.109126
73	6	0	0.075766	-2.633596	2.255813
74	6	0	1.118854	-3.543466	2.407903
75	6	0	-0.937449	-4.060486	0.615245
76	6	0	0.096956	-4.977239	0.775988
77	6	0	1.137477	-4.718661	1.661807
78	1	0	1.918316	-3.330449	3.111960
79	1	0	0.078636	-1.723658	2.847318
80	1	0	1.955314	-5.424225	1.769329
81	1	0	0.097665	-5.887011	0.184166
82	1	0	-1.710941	-4.259790	-0.117490
83	6	0	-1.716274	-1.291696	-1.305638
84	6	0	-2.004733	-1.410783	-2.661029
85	6	0	-3.115152	-2.139404	-3.081752
86	6	0	-3.635488	-2.635364	-0.773159
87	6	0	-3.929456	-2.751529	-2.131467
88	1	0	-0.823545	-0.744276	-1.024741
89	1	0	-1.335991	-0.939986	-3.376395
90	1	0	-3.342201	-2.235121	-4.139898
91	1	0	-4.796059	-3.327824	-2.441562
92	1	0	-4.271284	-3.134863	-0.049314
93	6	0	2.887837	-0.050516	-2.074644
94	1	0	2.869983	-0.781016	-2.871440
95	1	0	3.801939	0.408075	-1.723731
96	7	0	1.779147	0.720310	-1.982930
97	8	0	1.814186	1.782346	-1.276558
98	8	0	0.713503	0.357090	-2.516965
99	8	0	0.057546	-3.717728	-2.261933
100	8	0	1.348647	-0.119855	0.896960
101	7	0	3.556561	-0.746264	1.051816
102	7	0	1.205747	-2.210806	-1.078980
103	8	0	1.942269	-2.891063	-3.176148
104	1	0	4.449715	-0.585622	2.921599
105	6	0	-0.208060	-4.398645	-3.490425
106	6	0	2.346181	-1.730417	-0.678116

107	6	0	2.304829	-0.767184	0.497630
108	6	0	3.742377	-2.255037	-0.686032
109	6	0	4.418187	-1.621272	0.373514
110	6	0	4.416241	-3.187065	-1.463519
111	1	0	3.911623	-3.669996	-2.290746
112	6	0	1.160062	-2.948765	-2.235919
113	6	0	5.754584	-3.466749	-1.172594
114	1	0	6.291113	-4.192634	-1.774319
115	6	0	3.912368	0.048437	2.207934
116	1	0	2.964160	0.342254	2.672039
117	6	0	5.749997	-1.871346	0.659706
118	1	0	6.261688	-1.349418	1.461767
119	6	0	6.411040	-2.813284	-0.132309
120	1	0	7.454950	-3.032963	0.068197
121	6	0	4.732845	1.275063	1.865571
122	6	0	5.827771	1.637977	2.648833
123	6	0	4.371240	2.073202	0.776838
124	6	0	6.553766	2.790801	2.353617
125	1	0	6.115647	1.016780	3.494010
126	6	0	5.097260	3.223779	0.483640
127	1	0	3.517605	1.803503	0.156687
128	6	0	6.189606	3.585826	1.270583
129	1	0	7.407937	3.062697	2.965993
130	1	0	4.807684	3.835023	-0.366164
131	1	0	6.757336	4.481161	1.037339
132	6	0	0.596372	-5.679422	-3.601101
133	1	0	1.664946	-5.456282	-3.625775
134	1	0	0.389756	-6.336425	-2.752041
135	1	0	0.331975	-6.212264	-4.519076
136	1	0	-1.280631	-4.606026	-3.462676
137	1	0	0.003275	-3.728272	-4.328006

G1-1b-I-*re*-IM3

Zero-point correction= 1.17715 (a.u.)

Thermal correction to Gibbs Free Energy= 1.10647 (a.u.)

Sum of electronic and zero-point Energies= -3044.74317 (a.u.)

Sum of electronic and thermal Free Energies= -3044.81385 (a.u.)

Standard orientation:

Center Number	Atomic Number	Atomic Type	Coordinates (Angstroms) X	Y	Z
1	6	0	-0.186988	-0.655571	4.879251

2	6	0	-1.424779	-1.283526	4.240658
3	6	0	-1.554094	-0.911997	2.763375
4	7	0	-0.311215	-1.257209	2.048326
5	6	0	1.072046	-0.986749	4.076590
6	6	0	0.863825	-0.589111	2.615541
7	1	0	-0.075496	-0.988929	5.915324
8	1	0	-0.310848	0.435669	4.906298
9	1	0	-2.333996	-0.964557	4.760202
10	1	0	-1.373624	-2.376579	4.326187
11	1	0	-2.395881	-1.420454	2.295977
12	1	0	-1.728020	0.164546	2.639709
13	1	0	1.944329	-0.458960	4.470565
14	1	0	1.298547	-2.058600	4.136811
15	1	0	0.606663	0.483413	2.593274
16	6	0	-0.268219	-2.306842	1.189709
17	7	0	-1.296992	-2.487108	0.372917
18	7	0	0.812607	-3.112300	1.221800
19	6	0	1.366278	-3.963129	0.159786
20	1	0	-1.928106	-1.647089	0.266851
21	6	0	2.179286	-0.690925	1.806365
22	8	0	3.156611	-1.214622	2.319699
23	7	0	2.139273	-0.083608	0.590622
24	1	0	1.243518	0.358001	0.380363
25	6	0	1.380507	-3.267256	-1.201767
26	6	0	1.998430	-4.181068	-2.261058
27	6	0	3.405776	-4.631206	-1.863335
28	6	0	2.778906	-4.373954	0.579980
29	1	0	1.962294	-2.342680	-1.105867
30	1	0	0.372821	-2.960957	-1.501377
31	1	0	1.357231	-5.064506	-2.392991
32	1	0	2.020338	-3.658404	-3.222286
33	1	0	0.773262	-4.886163	0.092100
34	1	0	3.804363	-5.330232	-2.605797
35	1	0	2.733293	-4.892016	1.545726
36	1	0	3.384970	-3.470961	0.725500
37	6	0	3.409406	-5.282548	-0.477817
38	1	0	4.073949	-3.759994	-1.862852
39	1	0	4.429212	-5.543618	-0.177044
40	1	0	2.843785	-6.223313	-0.522699
41	1	0	1.412670	-2.999658	2.030191
42	6	0	4.610467	-0.089494	0.204333
43	6	0	3.268410	0.606923	-0.081128
44	6	0	3.051949	0.557449	-1.605009
45	6	0	3.231902	2.054730	0.452752

46	6	0	5.704926	0.546831	0.780587
47	6	0	6.911034	-0.139193	0.951958
48	6	0	7.036285	-1.459903	0.545473
49	6	0	5.948163	-2.096101	-0.056519
50	6	0	4.758805	-1.408679	-0.233470
51	1	0	5.636895	1.580584	1.099794
52	1	0	7.753134	0.372707	1.407506
53	1	0	7.973169	-1.989974	0.684997
54	1	0	6.032014	-3.126288	-0.392886
55	1	0	3.916952	-1.890625	-0.719579
56	6	0	3.316351	2.249361	1.841275
57	6	0	3.230457	3.521284	2.391355
58	6	0	3.049309	3.168483	-0.364243
59	6	0	2.962611	4.448467	0.189126
60	6	0	3.050697	4.630455	1.562520
61	1	0	3.305216	3.648406	3.467108
62	1	0	3.474430	1.392070	2.491792
63	1	0	2.972045	5.626190	1.988066
64	1	0	2.788656	5.298340	-0.465283
65	1	0	2.953597	3.049412	-1.437841
66	6	0	1.849113	0.166490	-2.191500
67	6	0	1.726352	0.111256	-3.582225
68	6	0	2.789677	0.468311	-4.399855
69	6	0	4.116153	0.927818	-2.439179
70	6	0	3.986854	0.890295	-3.820782
71	1	0	0.986260	-0.108403	-1.593855
72	1	0	0.774272	-0.195108	-4.000893
73	1	0	2.690099	0.424250	-5.479994
74	1	0	4.826352	1.178977	-4.445759
75	1	0	5.058499	1.238074	-1.995633
76	6	0	-1.777311	-3.788744	-0.101470
77	6	0	-2.458637	-4.554335	1.037894
78	6	0	-2.707976	-3.608892	-1.299716
79	1	0	-0.926783	-4.380036	-0.453433
80	6	0	-2.978919	-5.906996	0.547672
81	1	0	-3.284549	-3.932995	1.402898
82	1	0	-1.741707	-4.691061	1.858478
83	6	0	-3.215805	-4.971357	-1.779194
84	1	0	-3.559833	-2.993280	-0.994789
85	1	0	-2.180029	-3.080745	-2.098682
86	6	0	-3.915276	-5.729874	-0.649186
87	1	0	-3.490808	-6.427742	1.363356
88	1	0	-2.130520	-6.540413	0.250527
89	1	0	-3.896336	-4.832271	-2.625529

90	1	0	-2.370897	-5.570372	-2.148582
91	1	0	-4.269506	-6.703566	-1.004492
92	1	0	-4.798119	-5.159788	-0.330664
93	6	0	-3.057514	1.051231	-1.884857
94	1	0	-3.115861	2.074002	-2.253576
95	1	0	-3.907573	0.448479	-2.200793
96	7	0	-1.836302	0.475287	-2.514583
97	8	0	-1.679185	-0.730026	-2.484303
98	8	0	-1.049093	1.264729	-3.012228
99	6	0	-3.419434	4.327015	1.270836
100	6	0	-2.963601	3.128008	0.747916
101	6	0	-3.817874	2.151484	0.218020
102	6	0	-5.182414	2.395410	0.208793
103	6	0	-5.668313	3.593693	0.745471
104	6	0	-4.798938	4.545731	1.266529
105	1	0	-2.730304	5.072466	1.653414
106	1	0	-5.861384	1.663443	-0.207776
107	1	0	-6.736699	3.782803	0.745131
108	1	0	-5.190896	5.474509	1.668646
109	7	0	-1.627261	2.702151	0.662292
110	6	0	-1.542230	1.477337	0.075630
111	8	0	-0.510644	0.849606	-0.104557
112	6	0	-0.478635	3.511927	1.024130
113	1	0	0.386477	2.838878	1.056220
114	6	0	-2.962556	0.998426	-0.325246
115	7	0	-3.099001	-0.296953	0.262618
116	6	0	-4.252153	-0.950856	0.256509
117	8	0	-4.479739	-1.983494	0.897388
118	8	0	-5.240682	-0.455437	-0.579941
119	6	0	-6.474463	-1.171312	-0.566016
120	6	0	-7.373622	-0.557767	-1.618070
121	1	0	-7.588440	0.491927	-1.397625
122	1	0	-6.906224	-0.613752	-2.605090
123	1	0	-8.324076	-1.096998	-1.657880
124	1	0	-6.285329	-2.229208	-0.772514
125	1	0	-6.922718	-1.109671	0.431843
126	1	0	-0.630090	3.924329	2.027043
127	6	0	-0.234944	4.612409	0.014042
128	6	0	-0.105369	5.940768	0.410219
129	6	0	-0.083412	4.277115	-1.333996
130	6	0	0.186920	6.930299	-0.528076
131	1	0	-0.218804	6.201716	1.460344
132	6	0	0.208097	5.262522	-2.270233
133	1	0	-0.171100	3.238095	-1.648447

134	6	0	0.347263	6.591683	-1.868702
135	1	0	0.286201	7.963940	-0.211061
136	1	0	0.332238	4.991464	-3.314125
137	1	0	0.574220	7.360440	-2.600836

G1-1b-I-*si*-IM3

Zero-point correction= 1.17744 (a.u.)

Thermal correction to Gibbs Free Energy= 1.10756 (a.u.)

Sum of electronic and zero-point Energies= -3044.75398 (a.u.)

Sum of electronic and thermal Free Energies= -3044.82386 (a.u.)

Standard orientation:

Center Number	Atomic Number	Atomic Type	Coordinates (Angstroms) X	Y	Z
1	6	0	1.176034	-0.779043	4.862995
2	6	0	1.622884	-2.195232	4.497680
3	6	0	1.294021	-2.500360	3.040414
4	7	0	1.967236	-1.497749	2.188054
5	6	0	1.755740	0.237789	3.881074
6	6	0	1.415924	-0.153960	2.436738
7	1	0	1.482222	-0.530911	5.884402
8	1	0	0.081929	-0.725466	4.822817
9	1	0	1.123807	-2.931004	5.136326
10	1	0	2.704702	-2.298147	4.647374
11	1	0	1.656374	-3.491170	2.753581
12	1	0	0.206611	-2.477323	2.883699
13	1	0	1.357988	1.239455	4.075303
14	1	0	2.848250	0.283540	3.965060
15	1	0	0.322680	-0.165136	2.309430
16	6	0	2.065929	-1.854093	0.817325
17	7	0	0.983455	-2.027312	0.095261
18	7	0	3.341132	-1.971442	0.436435
19	6	0	4.012906	-2.048753	-0.863664
20	1	0	0.006003	-2.044872	0.584486
21	6	0	2.045815	0.868927	1.492402
22	8	0	3.215116	0.784128	1.139517
23	7	0	1.200976	1.875191	1.162090
24	1	0	0.217690	1.656562	1.284269
25	6	0	4.193397	-0.663039	-1.489692
26	6	0	4.902606	-0.787686	-2.838711
27	6	0	6.252336	-1.492867	-2.688354
28	6	0	5.365003	-2.738592	-0.673317

29	1	0	4.783429	-0.043608	-0.802238
30	1	0	3.222815	-0.168621	-1.594043
31	1	0	4.265227	-1.360191	-3.529391
32	1	0	5.030677	0.205173	-3.281090
33	1	0	3.424483	-2.672706	-1.537159
34	1	0	6.735988	-1.608276	-3.663769
35	1	0	5.216552	-3.724643	-0.220176
36	1	0	5.972096	-2.143866	0.024217
37	6	0	6.090606	-2.857791	-2.015591
38	1	0	6.914133	-0.866784	-2.075140
39	1	0	7.065589	-3.333045	-1.870012
40	1	0	5.511152	-3.518939	-2.674886
41	1	0	3.933257	-1.616742	1.180923
42	6	0	2.611942	3.867764	0.755590
43	6	0	1.488932	2.956868	0.205015
44	6	0	1.797538	2.345216	-1.178265
45	6	0	0.172429	3.757924	0.070091
46	6	0	2.309751	5.073271	1.395597
47	6	0	3.316291	5.879553	1.923949
48	6	0	4.648729	5.501031	1.814686
49	6	0	4.964438	4.305168	1.174810
50	6	0	3.959836	3.496677	0.654427
51	1	0	1.280823	5.401844	1.484127
52	1	0	3.050325	6.809390	2.417151
53	1	0	5.433960	6.130762	2.221521
54	1	0	6.000366	3.993806	1.081116
55	1	0	4.215792	2.560157	0.176485
56	6	0	-0.730901	3.847574	1.136235
57	6	0	-1.921941	4.557924	1.006155
58	6	0	-0.134271	4.433790	-1.114625
59	6	0	-1.323098	5.148211	-1.242119
60	6	0	-2.227645	5.205746	-0.186145
61	1	0	-2.615565	4.591453	1.840506
62	1	0	-0.512967	3.365243	2.083936
63	1	0	-3.171740	5.730674	-0.298604
64	1	0	-1.547150	5.649982	-2.178589
65	1	0	0.549885	4.388720	-1.955412
66	6	0	1.003421	1.276265	-1.615803
67	6	0	1.126594	0.777314	-2.908450
68	6	0	2.063031	1.320923	-3.787142
69	6	0	2.717453	2.895409	-2.071906
70	6	0	2.858935	2.377960	-3.361055
71	1	0	0.278959	0.833758	-0.933394
72	1	0	0.487866	-0.038830	-3.232978

73	1	0	2.165675	0.925258	-4.792844
74	1	0	3.588147	2.817174	-4.035183
75	1	0	3.321736	3.745265	-1.774621
76	6	0	-3.570474	-1.275040	2.089399
77	1	0	-3.887606	-2.257248	2.436105
78	1	0	-4.406168	-0.606611	1.884477
79	7	0	-2.793463	-0.646758	3.193958
80	8	0	-2.971981	0.539365	3.411943
81	8	0	-2.022406	-1.358687	3.813060
82	8	0	-0.662141	-4.335888	1.406592
83	8	0	-1.339949	0.579556	1.024448
84	7	0	-2.800874	0.315625	-0.724434
85	7	0	-1.537579	-2.328190	1.021401
86	8	0	-2.893883	-4.092831	1.704153
87	1	0	-2.315250	1.476379	-2.390246
88	6	0	-0.823644	-5.699705	1.787026
89	6	0	-2.678500	-1.476439	0.826744
90	6	0	-2.166273	-0.078992	0.414694
91	6	0	-3.610638	-1.802591	-0.342133
92	6	0	-3.646707	-0.700504	-1.205940
93	6	0	-4.361824	-2.927716	-0.645836
94	1	0	-4.342837	-3.782451	0.019371
95	6	0	-1.812323	-3.577320	1.395002
96	6	0	-5.125699	-2.932221	-1.820150
97	1	0	-5.713296	-3.809425	-2.070183
98	6	0	-2.619826	1.613250	-1.345822
99	1	0	-1.793423	2.096792	-0.813574
100	6	0	-4.403177	-0.679849	-2.366279
101	1	0	-4.427983	0.193733	-3.009495
102	6	0	-5.146485	-1.825984	-2.663730
103	1	0	-5.751186	-1.844609	-3.564871
104	6	0	-3.856280	2.480092	-1.255590
105	6	0	-4.275674	3.225888	-2.355191
106	6	0	-4.539612	2.600758	-0.043750
107	6	0	-5.359650	4.095191	-2.246427
108	1	0	-3.742170	3.135482	-3.298689
109	6	0	-5.625212	3.463088	0.064929
110	1	0	-4.203354	2.033649	0.822328
111	6	0	-6.036073	4.215134	-1.035848
112	1	0	-5.676436	4.674089	-3.108482
113	1	0	-6.148831	3.553983	1.011583
114	1	0	-6.882210	4.889498	-0.949355
115	6	0	-1.344094	-6.546906	0.640132
116	1	0	-2.326017	-6.186237	0.326852

117	1	0	-0.662124	-6.510389	-0.215072
118	1	0	-1.440116	-7.590978	0.953662
119	1	0	0.174038	-6.028687	2.093298
120	1	0	-1.495511	-5.763308	2.646584
121	6	0	0.951109	-2.517842	-1.288139
122	6	0	1.295877	-4.011373	-1.342052
123	6	0	-0.428391	-2.291105	-1.910525
124	1	0	1.668851	-1.933180	-1.879298
125	6	0	1.293557	-4.512899	-2.786306
126	1	0	0.537655	-4.533644	-0.746739
127	1	0	2.260616	-4.206155	-0.859246
128	6	0	-0.431920	-2.764747	-3.365754
129	1	0	-1.175020	-2.854799	-1.342526
130	1	0	-0.707159	-1.231429	-1.831341
131	6	0	-0.056083	-4.245780	-3.455478
132	1	0	1.529899	-5.581600	-2.811521
133	1	0	2.087023	-3.999778	-3.350773
134	1	0	-1.420273	-2.592517	-3.803951
135	1	0	0.288965	-2.177071	-3.952934
136	1	0	-0.034694	-4.570989	-4.500830
137	1	0	-0.829299	-4.838816	-2.949307

G1-1b-I-*re*-TS3

Zero-point correction= 1.17334 (a.u.)

Thermal correction to Gibbs Free Energy= 1.10273 (a.u.)

Sum of electronic and zero-point Energies= -3044.74217 (a.u.)

Sum of electronic and thermal Free Energies= -3044.81278 (a.u.)

Standard orientation:

Center Number	Atomic Number	Atomic Type	Coordinates (Angstroms) X	Y	Z
1	6	0	-0.380025	0.580215	4.628158
2	6	0	-1.498859	-0.422229	4.356115
3	6	0	-1.653937	-0.652307	2.855897
4	7	0	-0.383118	-1.108120	2.289020
5	6	0	0.919732	0.129725	3.964365
6	6	0	0.697235	-0.127557	2.471320
7	1	0	-0.229243	0.715937	5.703463
8	1	0	-0.671763	1.558840	4.219311
9	1	0	-2.450190	-0.063389	4.761475
10	1	0	-1.269666	-1.379388	4.839392
11	1	0	-2.419332	-1.398373	2.639258

12	1	0	-1.971816	0.285888	2.375227
13	1	0	1.697886	0.891187	4.080697
14	1	0	1.297414	-0.791777	4.420107
15	1	0	0.338491	0.814371	2.022172
16	6	0	-0.356052	-2.173989	1.409906
17	7	0	-1.387857	-2.384969	0.636449
18	7	0	0.769620	-2.933959	1.510313
19	6	0	1.393007	-3.845404	0.550408
20	1	0	-2.224541	-1.436864	0.423045
21	6	0	2.069851	-0.425844	1.808542
22	8	0	2.969292	-0.936719	2.459253
23	7	0	2.173964	0.028735	0.529167
24	1	0	1.300801	0.407146	0.168697
25	6	0	1.514951	-3.241098	-0.849799
26	6	0	2.178597	-4.234706	-1.804826
27	6	0	3.544407	-4.690829	-1.284234
28	6	0	2.767664	-4.239054	1.094046
29	1	0	2.104181	-2.320703	-0.775743
30	1	0	0.534606	-2.936063	-1.231375
31	1	0	1.524444	-5.110800	-1.921866
32	1	0	2.279739	-3.780084	-2.795314
33	1	0	0.807415	-4.774366	0.491377
34	1	0	3.961318	-5.458965	-1.944192
35	1	0	2.647265	-4.690095	2.086331
36	1	0	3.375599	-3.334892	1.224238
37	6	0	3.449464	-5.229681	0.146582
38	1	0	4.242799	-3.844760	-1.314256
39	1	0	4.444577	-5.481717	0.528240
40	1	0	2.871487	-6.163889	0.137666
41	1	0	1.333143	-2.725623	2.325032
42	6	0	4.672652	-0.053462	0.309800
43	6	0	3.362056	0.611581	-0.140071
44	6	0	3.222926	0.402962	-1.661654
45	6	0	3.325452	2.114999	0.210342
46	6	0	5.759766	0.651994	0.817590
47	6	0	6.950971	-0.011387	1.127028
48	6	0	7.069250	-1.379670	0.929430
49	6	0	5.989783	-2.088904	0.399344
50	6	0	4.815653	-1.424558	0.084559
51	1	0	5.699705	1.723121	0.972876
52	1	0	7.787266	0.556473	1.523081
53	1	0	7.994284	-1.892215	1.173734
54	1	0	6.066975	-3.158948	0.225146
55	1	0	3.984880	-1.966809	-0.351041

56	6	0	3.358365	2.476813	1.566848
57	6	0	3.259294	3.806603	1.952539
58	6	0	3.182217	3.120107	-0.744740
59	6	0	3.079481	4.457971	-0.356229
60	6	0	3.117212	4.806319	0.987205
61	1	0	3.294262	4.065092	3.006697
62	1	0	3.481185	1.702709	2.321159
63	1	0	3.032117	5.847038	1.284944
64	1	0	2.942962	5.222578	-1.115365
65	1	0	3.131171	2.871538	-1.799009
66	6	0	2.011997	0.065688	-2.269811
67	6	0	1.922492	-0.047120	-3.658457
68	6	0	3.036327	0.180496	-4.457341
69	6	0	4.339982	0.626547	-2.475735
70	6	0	4.248795	0.519004	-3.858264
71	1	0	1.115052	-0.123778	-1.691521
72	1	0	0.965908	-0.308435	-4.100338
73	1	0	2.964654	0.092898	-5.536979
74	1	0	5.129467	0.697461	-4.467687
75	1	0	5.290947	0.885463	-2.018472
76	6	0	-1.673862	-3.667988	-0.012897
77	6	0	-2.497678	-4.591583	0.892093
78	6	0	-2.362657	-3.435778	-1.358610
79	1	0	-0.748124	-4.197305	-0.240723
80	6	0	-2.788023	-5.916099	0.180335
81	1	0	-3.428865	-4.078294	1.149723
82	1	0	-1.942781	-4.779730	1.819571
83	6	0	-2.629293	-4.769959	-2.056980
84	1	0	-3.312934	-2.920930	-1.197977
85	1	0	-1.741901	-2.780465	-1.980029
86	6	0	-3.472804	-5.686960	-1.168823
87	1	0	-3.404622	-6.556354	0.820163
88	1	0	-1.842899	-6.453969	0.012803
89	1	0	-3.129680	-4.593731	-3.014841
90	1	0	-1.674065	-5.266249	-2.284331
91	1	0	-3.656754	-6.644005	-1.669497
92	1	0	-4.450518	-5.216851	-0.998835
93	6	0	-3.303885	1.164707	-1.690573
94	1	0	-2.956073	2.171809	-1.925809
95	1	0	-4.367297	1.038040	-1.874550
96	7	0	-2.592576	0.241643	-2.618184
97	8	0	-3.142599	-0.808511	-2.886389
98	8	0	-1.503198	0.591289	-3.044742
99	6	0	-2.964263	4.027510	1.639383

100	6	0	-2.679965	2.853812	0.960482
101	6	0	-3.661714	1.913453	0.623227
102	6	0	-4.974937	2.149476	0.999415
103	6	0	-5.281101	3.317551	1.707226
104	6	0	-4.292070	4.246170	2.012585
105	1	0	-2.187981	4.749130	1.869378
106	1	0	-5.751205	1.437408	0.752580
107	1	0	-6.305807	3.501732	2.012133
108	1	0	-4.549265	5.153001	2.550336
109	7	0	-1.415447	2.400929	0.549203
110	6	0	-1.501262	1.159222	-0.013051
111	8	0	-0.562556	0.457986	-0.348572
112	6	0	-0.206969	3.206427	0.602141
113	1	0	0.641229	2.528439	0.467321
114	6	0	-2.993123	0.804381	-0.197026
115	7	0	-3.184284	-0.567055	0.172573
116	6	0	-4.399334	-1.139283	0.152323
117	8	0	-4.663822	-2.240561	0.618749
118	8	0	-5.372182	-0.402445	-0.471363
119	6	0	-6.663039	-1.015070	-0.528745
120	6	0	-7.553517	-0.111019	-1.352941
121	1	0	-7.647960	0.878490	-0.896140
122	1	0	-7.149473	0.010165	-2.361684
123	1	0	-8.553204	-0.545602	-1.434555
124	1	0	-6.568886	-2.006912	-0.978930
125	1	0	-7.047932	-1.149157	0.487812
126	1	0	-0.118977	3.658596	1.595407
127	6	0	-0.204755	4.257699	-0.484942
128	6	0	-0.154963	5.614802	-0.178229
129	6	0	-0.228661	3.851822	-1.822410
130	6	0	-0.119936	6.565309	-1.197226
131	1	0	-0.126468	5.930213	0.862455
132	6	0	-0.192372	4.799697	-2.839658
133	1	0	-0.263819	2.789863	-2.064927
134	6	0	-0.135888	6.158639	-2.528096
135	1	0	-0.081191	7.621839	-0.950562
136	1	0	-0.201801	4.477627	-3.876226
137	1	0	-0.105610	6.897666	-3.322735

G1-1b-I-*si*-TS3

Zero-point correction= 1.17305 (a.u.)

Thermal correction to Gibbs Free Energy= 1.10329 (a.u.)

Sum of electronic and zero-point Energies= -3044.75106 (a.u.)

Sum of electronic and thermal Free Energies= -3044.82082 (a.u.)

Standard orientation:

Center Number	Atomic Number	Atomic Type	Coordinates (Angstroms) X	Y	Z
1	6	0	-0.835947	-2.542021	-1.551972
2	6	0	-1.926518	-1.692734	-2.227646
3	6	0	-3.361819	-2.214882	-2.102441
4	6	0	-3.455085	-3.694876	-2.463412
5	6	0	-1.047070	-4.039165	-1.845593
6	1	0	-1.670815	-1.667234	-3.296229
7	1	0	-1.854086	-0.657937	-1.869406
8	1	0	-3.715580	-2.088120	-1.076867
9	1	0	-4.014928	-1.608265	-2.739902
10	1	0	0.104841	-2.213942	-2.005146
11	1	0	-4.482247	-4.048918	-2.324658
12	1	0	-0.785309	-4.219526	-2.898494
13	6	0	-2.483663	-4.491933	-1.595670
14	1	0	-3.205187	-3.845409	-3.523371
15	1	0	-2.570232	-5.565956	-1.792063
16	1	0	-2.725872	-4.325553	-0.537817
17	6	0	0.330553	-1.671611	4.757773
18	6	0	-0.318431	-2.945415	4.216603
19	6	0	-0.744676	-2.743151	2.766816
20	7	0	0.439347	-2.399314	1.962750
21	6	0	1.474877	-1.234612	3.846882
22	6	0	1.001581	-1.103097	2.392994
23	1	0	0.698376	-1.824753	5.777496
24	1	0	-0.421063	-0.873835	4.792918
25	1	0	-1.197378	-3.213163	4.811942
26	1	0	0.391320	-3.780037	4.269509
27	1	0	-1.187970	-3.650818	2.352618
28	1	0	-1.499266	-1.948012	2.711748
29	1	0	1.882878	-0.269580	4.167509
30	1	0	2.289644	-1.967616	3.862562
31	1	0	0.215193	-0.336493	2.335788
32	6	0	0.338051	-2.635233	0.564463
33	7	0	-0.723769	-2.265691	-0.109812
34	7	0	1.438688	-3.289189	0.137810
35	6	0	2.062456	-3.703088	-1.126613
36	1	0	-1.632318	-1.605699	0.401019
37	6	0	2.213368	-0.679265	1.555665
38	8	0	3.158679	-1.433965	1.367700

39	7	0	2.138817	0.611053	1.148843
40	1	0	1.208780	1.010905	1.196060
41	6	0	3.336827	-2.895033	-1.385062
42	6	0	4.020640	-3.361030	-2.671503
43	6	0	4.338920	-4.856195	-2.621685
44	6	0	2.392404	-5.198455	-1.046887
45	1	0	4.013137	-3.027559	-0.529668
46	1	0	3.095359	-1.831365	-1.430270
47	1	0	3.358298	-3.156426	-3.524888
48	1	0	4.931642	-2.775732	-2.836918
49	1	0	1.379046	-3.546153	-1.958366
50	1	0	4.794072	-5.184152	-3.562043
51	1	0	1.480673	-5.774247	-0.855082
52	1	0	3.059852	-5.359834	-0.188286
53	6	0	3.078960	-5.672718	-2.330315
54	1	0	5.076326	-5.039271	-1.828672
55	1	0	3.319376	-6.737749	-2.251329
56	1	0	2.376370	-5.568890	-3.169098
57	1	0	-0.360475	-4.637616	-1.240517
58	1	0	2.082386	-3.375411	0.918334
59	6	0	4.494832	1.308231	0.824028
60	6	0	3.059065	1.324567	0.251271
61	6	0	2.950332	0.731301	-1.169469
62	6	0	2.528434	2.777576	0.179446
63	6	0	4.973975	2.380440	1.580137
64	6	0	6.255016	2.357042	2.129376
65	6	0	7.085613	1.262560	1.923690
66	6	0	6.620999	0.188171	1.168604
67	6	0	5.339781	0.208020	0.629814
68	1	0	4.351675	3.252810	1.745639
69	1	0	6.599123	3.204543	2.714067
70	1	0	8.085611	1.244293	2.346022
71	1	0	7.255725	-0.676723	1.001238
72	1	0	4.981457	-0.645027	0.066569
73	6	0	1.815093	3.344391	1.242704
74	6	0	1.337340	4.650794	1.165786
75	6	0	2.772651	3.567985	-0.947541
76	6	0	2.295714	4.874050	-1.022781
77	6	0	1.567943	5.419939	0.030146
78	1	0	0.768652	5.058526	1.995815
79	1	0	1.627884	2.773194	2.146570
80	1	0	1.165458	6.425943	-0.041876
81	1	0	2.486412	5.460312	-1.916627
82	1	0	3.324844	3.158728	-1.786714

83	6	0	1.693769	0.318329	-1.631615
84	6	0	1.521735	-0.109469	-2.945373
85	6	0	2.606009	-0.154412	-3.819918
86	6	0	4.024566	0.707243	-2.061760
87	6	0	3.857208	0.254806	-3.370831
88	1	0	0.839846	0.328408	-0.957469
89	1	0	0.532500	-0.403285	-3.286187
90	1	0	2.474805	-0.497739	-4.841354
91	1	0	4.711442	0.232967	-4.040561
92	1	0	5.003187	1.047009	-1.740421
93	6	0	-3.465471	1.293310	1.845838
94	1	0	-4.346681	0.801037	2.252248
95	1	0	-3.647537	2.335309	1.584592
96	7	0	-2.431914	1.306887	2.918657
97	8	0	-1.788884	2.331237	3.075830
98	8	0	-2.289202	0.289111	3.571531
99	8	0	-3.453977	-2.943946	1.383540
100	8	0	-0.609056	1.097687	0.772842
101	7	0	-1.900908	1.933697	-0.932148
102	7	0	-2.732264	-0.910724	0.838514
103	8	0	-4.885575	-1.215066	1.648485
104	1	0	-0.821264	2.716050	-2.541226
105	6	0	-4.485934	-3.801800	1.874233
106	6	0	-2.977675	0.486237	0.600284
107	6	0	-1.667052	1.174244	0.172199
108	6	0	-3.904948	0.870213	-0.550723
109	6	0	-3.220413	1.755653	-1.391958
110	6	0	-5.217241	0.530406	-0.838628
111	1	0	-5.755607	-0.148007	-0.186839
112	6	0	-3.769345	-1.616701	1.311950
113	6	0	-5.817770	1.079062	-1.978470
114	1	0	-6.842917	0.817934	-2.219544
115	6	0	-0.951491	2.892428	-1.467951
116	1	0	0.005483	2.690834	-0.972667
117	6	0	-3.800618	2.321779	-2.514613
118	1	0	-3.256114	3.022098	-3.139447
119	6	0	-5.121526	1.963331	-2.797590
120	1	0	-5.608696	2.388611	-3.669257
121	6	0	-1.380796	4.319240	-1.200795
122	6	0	-1.272530	5.288980	-2.194887
123	6	0	-1.830256	4.685029	0.070136
124	6	0	-1.598754	6.616886	-1.923281
125	1	0	-0.922567	5.004828	-3.184660
126	6	0	-2.162460	6.008068	0.340480

127	1	0	-1.889751	3.937184	0.859781
128	6	0	-2.044583	6.978089	-0.655228
129	1	0	-1.508966	7.366087	-2.703827
130	1	0	-2.505990	6.285862	1.332203
131	1	0	-2.302704	8.010722	-0.442032
132	6	0	-5.482460	-4.154671	0.786417
133	1	0	-5.952786	-3.246902	0.400928
134	1	0	-4.991848	-4.681025	-0.037326
135	1	0	-6.264290	-4.805593	1.189438
136	1	0	-3.962594	-4.692924	2.231907
137	1	0	-4.985571	-3.320182	2.717746

G1-1b-I-*re*-IM4

Zero-point correction= 1.17707 (a.u.)

Thermal correction to Gibbs Free Energy= 1.10593 (a.u.)

Sum of electronic and zero-point Energies= -3044.74969 (a.u.)

Sum of electronic and thermal Free Energies= -3044.82083 (a.u.)

Standard orientation:

Center Number	Atomic Number	Atomic Type	Coordinates (Angstroms) X	Y	Z
1	6	0	-0.553929	0.522326	4.834318
2	6	0	0.139463	1.687351	4.125002
3	6	0	0.674670	1.258658	2.756580
4	7	0	-0.386816	0.677568	1.920243
5	6	0	-1.626989	-0.113796	3.945087
6	6	0	-1.003948	-0.462704	2.588727
7	1	0	-0.991289	0.852630	5.781492
8	1	0	0.197434	-0.241319	5.080181
9	1	0	0.966818	2.069501	4.732436
10	1	0	-0.566690	2.515951	3.988559
11	1	0	1.122814	2.099200	2.229021
12	1	0	1.450820	0.493365	2.892877
13	1	0	-2.036725	-1.019376	4.397260
14	1	0	-2.461239	0.586597	3.810986
15	1	0	-0.156945	-1.137884	2.805418
16	6	0	-1.118596	1.587884	1.112369
17	7	0	-0.423885	2.378201	0.377071
18	7	0	-2.502312	1.464912	1.138850
19	6	0	-3.441121	2.595995	1.074319
20	1	0	1.393898	2.211233	0.149843
21	6	0	-1.912237	-1.339184	1.707096

22	8	0	-2.830988	-1.956338	2.227858
23	7	0	-1.587924	-1.396143	0.392323
24	1	0	-0.730868	-0.927118	0.095944
25	6	0	-4.516594	2.367661	0.011179
26	6	0	-5.475815	3.556258	-0.069159
27	6	0	-6.129707	3.816882	1.289027
28	6	0	-4.097095	2.839341	2.438847
29	1	0	-5.083127	1.468559	0.288424
30	1	0	-4.048795	2.163358	-0.958546
31	1	0	-4.919420	4.450847	-0.382247
32	1	0	-6.236737	3.373601	-0.834402
33	1	0	-2.872354	3.490157	0.818122
34	1	0	-6.789665	4.689016	1.236375
35	1	0	-3.326616	3.021414	3.196730
36	1	0	-4.639783	1.929030	2.736702
37	6	0	-5.071665	4.020204	2.374846
38	1	0	-6.758309	2.956178	1.555287
39	1	0	-5.544992	4.165476	3.351085
40	1	0	-4.504661	4.935814	2.157518
41	1	0	-2.848572	0.749924	1.765191
42	6	0	-3.695881	-2.433633	-0.450003
43	6	0	-2.162645	-2.360568	-0.565068
44	6	0	-1.930067	-1.830132	-1.992752
45	6	0	-1.463142	-3.708944	-0.307468
46	6	0	-4.395048	-3.609523	-0.718086
47	6	0	-5.789752	-3.615583	-0.737188
48	6	0	-6.500696	-2.446509	-0.497784
49	6	0	-5.804806	-1.263666	-0.250268
50	6	0	-4.416723	-1.257330	-0.234244
51	1	0	-3.858504	-4.532368	-0.912679
52	1	0	-6.316188	-4.542783	-0.942226
53	1	0	-7.586042	-2.451394	-0.510837
54	1	0	-6.351739	-0.341071	-0.076615
55	1	0	-3.870435	-0.333037	-0.064838
56	6	0	-1.863390	-4.506741	0.774108
57	6	0	-1.171908	-5.672981	1.087805
58	6	0	-0.341369	-4.099210	-1.044250
59	6	0	0.343141	-5.273352	-0.734571
60	6	0	-0.070557	-6.066108	0.329634
61	1	0	-1.498629	-6.278052	1.928226
62	1	0	-2.715799	-4.201035	1.371035
63	1	0	0.468675	-6.976725	0.572668
64	1	0	1.218522	-5.549615	-1.315957
65	1	0	0.015338	-3.480270	-1.861880

66	6	0	-1.635722	-0.491932	-2.254405
67	6	0	-1.511278	-0.042676	-3.569973
68	6	0	-1.690788	-0.914863	-4.635519
69	6	0	-2.134199	-2.695849	-3.073951
70	6	0	-2.009540	-2.247704	-4.382017
71	1	0	-1.498796	0.217684	-1.442602
72	1	0	-1.269589	0.998918	-3.756716
73	1	0	-1.587884	-0.559495	-5.655813
74	1	0	-2.164357	-2.940770	-5.203067
75	1	0	-2.390335	-3.734660	-2.885160
76	6	0	-0.982156	3.172123	-0.706143
77	6	0	-0.976836	4.675989	-0.401373
78	6	0	-0.137735	2.930758	-1.965505
79	1	0	-2.008824	2.853237	-0.945393
80	6	0	-1.555844	5.464493	-1.579132
81	1	0	0.064241	4.966430	-0.218434
82	1	0	-1.534452	4.894110	0.517470
83	6	0	-0.729338	3.676851	-3.160733
84	1	0	0.876087	3.303024	-1.782772
85	1	0	-0.049976	1.854701	-2.151675
86	6	0	-0.791077	5.180039	-2.875060
87	1	0	-1.542036	6.537170	-1.357840
88	1	0	-2.610797	5.183812	-1.717484
89	1	0	-0.131237	3.480817	-4.057620
90	1	0	-1.745059	3.305489	-3.364772
91	1	0	-1.253348	5.713067	-3.713300
92	1	0	0.233273	5.562793	-2.774967
93	6	0	3.889812	0.625088	-1.303145
94	1	0	4.191811	-0.422058	-1.334261
95	1	0	4.742888	1.295715	-1.369284
96	7	0	3.058496	0.869777	-2.519307
97	8	0	2.893180	2.030402	-2.849499
98	8	0	2.610739	-0.097034	-3.106304
99	6	0	4.274589	-1.365606	2.659292
100	6	0	3.597663	-0.708319	1.644954
101	6	0	4.003554	0.539519	1.160442
102	6	0	5.107609	1.166760	1.711750
103	6	0	5.792682	0.528960	2.752534
104	6	0	5.385033	-0.720733	3.209759
105	1	0	3.959707	-2.342267	3.010376
106	1	0	5.428246	2.134276	1.343437
107	1	0	6.654429	1.011082	3.201348
108	1	0	5.934510	-1.207697	4.008958
109	7	0	2.429478	-1.128611	0.986116

110	6	0	2.017147	-0.204552	0.066703
111	8	0	1.032432	-0.271277	-0.642193
112	6	0	1.891114	-2.475687	1.071792
113	1	0	0.878120	-2.445483	0.658565
114	6	0	3.091265	0.912724	0.001279
115	7	0	2.432615	2.189228	0.044280
116	6	0	3.008040	3.392762	-0.210432
117	8	0	2.410328	4.449689	-0.243637
118	8	0	4.341762	3.296604	-0.420549
119	6	0	4.973615	4.504671	-0.871788
120	6	0	6.405312	4.160381	-1.212877
121	1	0	6.936977	3.771140	-0.340170
122	1	0	6.444790	3.413133	-2.010119
123	1	0	6.927443	5.055936	-1.558901
124	1	0	4.428487	4.880011	-1.741900
125	1	0	4.910195	5.257923	-0.081329
126	1	0	1.816266	-2.767644	2.124086
127	6	0	2.749451	-3.448521	0.293290
128	6	0	3.246206	-4.606566	0.884210
129	6	0	3.016232	-3.193571	-1.056043
130	6	0	3.999908	-5.510790	0.136417
131	1	0	3.027122	-4.812937	1.929269
132	6	0	3.771252	-4.092596	-1.801086
133	1	0	2.610859	-2.301478	-1.532219
134	6	0	4.263961	-5.254927	-1.205898
135	1	0	4.383127	-6.412380	0.604211
136	1	0	3.967889	-3.890199	-2.849346
137	1	0	4.851690	-5.957004	-1.788880

G1-1b-I-*si*-IM4

Zero-point correction= 1.17814 (a.u.)

Thermal correction to Gibbs Free Energy= 1.10811 (a.u.)

Sum of electronic and zero-point Energies= -3044.75591 (a.u.)

Sum of electronic and thermal Free Energies= -3044.82595 (a.u.)

Standard orientation:

Center Number	Atomic Number	Atomic Type	Coordinates (Angstroms) X	Y	Z
1	6	0	-0.051912	-2.655834	-1.605150
2	6	0	-1.281725	-1.987274	-2.244981
3	6	0	-2.604304	-2.745908	-2.089270
4	6	0	-2.466208	-4.210901	-2.493162

5	6	0	-0.020874	-4.162113	-1.941734
6	1	0	-1.077317	-1.884886	-3.320430
7	1	0	-1.374379	-0.965648	-1.847289
8	1	0	-2.930181	-2.719674	-1.046281
9	1	0	-3.381681	-2.240981	-2.674517
10	1	0	0.818862	-2.172824	-2.067853
11	1	0	-3.419048	-4.731592	-2.345492
12	1	0	0.231821	-4.274932	-3.006275
13	6	0	-1.354434	-4.854564	-1.668110
14	1	0	-2.220878	-4.290676	-3.561875
15	1	0	-1.269566	-5.924754	-1.886711
16	1	0	-1.590563	-4.750042	-0.600159
17	6	0	0.596332	-1.725737	4.660075
18	6	0	0.446018	-3.153889	4.137729
19	6	0	0.089536	-3.129910	2.655305
20	7	0	1.148161	-2.440457	1.908657
21	6	0	1.591962	-0.952125	3.798655
22	6	0	1.209136	-1.031638	2.313028
23	1	0	0.919359	-1.723820	5.706199
24	1	0	-0.377494	-1.222034	4.616936
25	1	0	-0.335538	-3.686901	4.689555
26	1	0	1.386498	-3.702598	4.269449
27	1	0	-0.009367	-4.144438	2.259693
28	1	0	-0.880453	-2.628907	2.524947
29	1	0	1.630564	0.101502	4.095966
30	1	0	2.599880	-1.370663	3.902766
31	1	0	0.223735	-0.557304	2.171815
32	6	0	1.089025	-2.646572	0.480978
33	7	0	-0.006567	-2.409709	-0.160733
34	7	0	2.302403	-3.133797	0.078065
35	6	0	3.007584	-3.269489	-1.197933
36	1	0	-1.655542	-1.676657	0.632063
37	6	0	2.267407	-0.265492	1.517393
38	8	0	3.360489	-0.745835	1.257312
39	7	0	1.880314	1.003939	1.223926
40	1	0	0.881518	1.161485	1.280327
41	6	0	3.928158	-2.076080	-1.476530
42	6	0	4.691151	-2.274590	-2.787320
43	6	0	5.496710	-3.575209	-2.769536
44	6	0	3.817984	-4.570276	-1.163889
45	1	0	4.631984	-1.976307	-0.639158
46	1	0	3.343704	-1.152805	-1.498923
47	1	0	3.973412	-2.302152	-3.620082
48	1	0	5.346855	-1.416120	-2.969046

49	1	0	2.292576	-3.350281	-2.015866
50	1	0	6.013325	-3.721264	-3.724249
51	1	0	3.145695	-5.414188	-0.973018
52	1	0	4.520569	-4.521391	-0.319154
53	6	0	4.594310	-4.773008	-2.466741
54	1	0	6.271943	-3.504421	-1.994408
55	1	0	5.182814	-5.694870	-2.412596
56	1	0	3.879022	-4.903349	-3.290913
57	1	0	0.774281	-4.653365	-1.372214
58	1	0	2.942345	-3.073368	0.862088
59	6	0	3.999911	2.238338	0.977387
60	6	0	2.605070	1.954860	0.372966
61	6	0	2.644813	1.421923	-1.075713
62	6	0	1.757350	3.251667	0.359616
63	6	0	4.181417	3.297991	1.869155
64	6	0	5.419692	3.532709	2.464270
65	6	0	6.504831	2.716686	2.167165
66	6	0	6.336858	1.656830	1.279544
67	6	0	5.097359	1.414712	0.697851
68	1	0	3.352572	3.955609	2.107172
69	1	0	5.530948	4.361123	3.156980
70	1	0	7.472119	2.900110	2.624970
71	1	0	7.172507	1.004964	1.043882
72	1	0	4.971460	0.566035	0.036713
73	6	0	0.889321	3.564736	1.412506
74	6	0	0.119543	4.725654	1.385096
75	6	0	1.854850	4.155060	-0.703905
76	6	0	1.090440	5.318654	-0.727634
77	6	0	0.211695	5.606154	0.312560
78	1	0	-0.561117	4.929927	2.206005
79	1	0	0.804126	2.905293	2.269818
80	1	0	-0.408951	6.496413	0.277767
81	1	0	1.173213	5.995875	-1.572182
82	1	0	2.520305	3.943886	-1.533927
83	6	0	1.544523	0.705705	-1.564164
84	6	0	1.479677	0.322267	-2.901542
85	6	0	2.524070	0.622843	-3.773305
86	6	0	3.676613	1.736054	-1.964226
87	6	0	3.623758	1.328262	-3.296712
88	1	0	0.735723	0.426994	-0.890479
89	1	0	0.608525	-0.219124	-3.259963
90	1	0	2.478763	0.312394	-4.812510
91	1	0	4.445921	1.573693	-3.962102
92	1	0	4.530559	2.310100	-1.619946

93	6	0	-3.824889	0.586165	1.861059
94	1	0	-4.609124	-0.068022	2.237738
95	1	0	-4.205265	1.565182	1.572501
96	7	0	-2.863539	0.814102	2.981043
97	8	0	-2.501059	1.958243	3.188044
98	8	0	-2.506438	-0.162022	3.612638
99	8	0	-2.808110	-3.567039	1.428094
100	8	0	-0.958110	1.011450	0.909364
101	7	0	-2.298230	1.470500	-0.905680
102	7	0	-2.600869	-1.401467	0.961417
103	8	0	-4.608846	-2.225010	1.701258
104	1	0	-1.291825	2.336223	-2.518157
105	6	0	-3.606197	-4.684748	1.848758
106	6	0	-3.125799	-0.093824	0.655533
107	6	0	-1.965751	0.838431	0.250474
108	6	0	-4.047554	0.020294	-0.548865
109	6	0	-3.519545	0.985388	-1.413872
110	6	0	-5.239253	-0.613145	-0.857852
111	1	0	-5.651714	-1.356432	-0.184593
112	6	0	-3.437531	-2.380875	1.390776
113	6	0	-5.882500	-0.274846	-2.053741
114	1	0	-6.812717	-0.766435	-2.317262
115	6	0	-1.523397	2.560953	-1.471588
116	1	0	-0.579453	2.586389	-0.915802
117	6	0	-4.149331	1.344984	-2.593753
118	1	0	-3.735960	2.107774	-3.245055
119	6	0	-5.345734	0.690546	-2.901721
120	1	0	-5.865483	0.946048	-3.819473
121	6	0	-2.236474	3.890038	-1.344765
122	6	0	-2.198826	4.809267	-2.390901
123	6	0	-2.880033	4.232590	-0.153639
124	6	0	-2.794276	6.061722	-2.250504
125	1	0	-1.691920	4.547251	-3.316555
126	6	0	-3.482901	5.478234	-0.015211
127	1	0	-2.882150	3.532700	0.680191
128	6	0	-3.440754	6.396100	-1.064103
129	1	0	-2.757444	6.772266	-3.070415
130	1	0	-3.980741	5.736128	0.914387
131	1	0	-3.910745	7.368478	-0.955159
132	6	0	-4.420824	-5.232355	0.694981
133	1	0	-5.102765	-4.468681	0.312154
134	1	0	-3.767185	-5.565764	-0.115513
135	1	0	-5.015350	-6.086885	1.030595
136	1	0	-2.881274	-5.417109	2.209956

137	1	0	-4.245886	-4.374759	2.677724

G1-1b-P-*R*

Zero-point correction= 0.36895 (a.u.)

Thermal correction to Gibbs Free Energy= 0.32802 (a.u.)

Sum of electronic and zero-point Energies= -1274.96287 (a.u.)

Sum of electronic and thermal Free Energies= -1275.00380 (a.u.)

Standard orientation:

Center Number	Atomic Number	Atomic Type	Coordinates (Angstroms) X	Y	Z
1	1	0	-2.245340	-0.701130	1.404585
2	6	0	-0.811616	-0.706139	-1.296296
3	1	0	0.192643	-0.664569	-1.715595
4	1	0	-1.561702	-0.313714	-1.983190
5	7	0	-1.129398	-2.147135	-1.074288
6	8	0	-2.292822	-2.426439	-0.845484
7	8	0	-0.211582	-2.943767	-1.121890
8	6	0	1.885654	2.506966	0.222472
9	6	0	1.018028	1.436590	0.358436
10	6	0	-0.264070	1.439955	-0.206318
11	6	0	-0.704513	2.535876	-0.927103
12	6	0	0.151576	3.635979	-1.055616
13	6	0	1.425066	3.614798	-0.494694
14	1	0	2.884629	2.481710	0.644072
15	1	0	-1.694356	2.532512	-1.369803
16	1	0	-0.178563	4.509972	-1.606650
17	1	0	2.080040	4.471648	-0.616162
18	7	0	1.241049	0.234359	1.052669
19	6	0	0.138217	-0.566468	1.012582
20	8	0	-0.046175	-1.607573	1.607333
21	6	0	2.456281	-0.104290	1.769381
22	1	0	2.205442	-0.965863	2.395149
23	6	0	-0.878457	0.077303	0.041070
24	7	0	-2.164120	0.015945	0.690845
25	6	0	-3.310115	0.302251	0.001972
26	8	0	-3.351640	0.883379	-1.066098
27	8	0	-4.381088	-0.117715	0.681368
28	6	0	-5.646429	0.119931	0.040696
29	6	0	-6.716675	-0.422338	0.958490
30	1	0	-6.574942	-1.492538	1.126823
31	1	0	-6.694680	0.089350	1.923770

32	1	0	-7.700386	-0.269711	0.507600
33	1	0	-5.759701	1.194056	-0.132259
34	1	0	-5.647705	-0.382800	-0.930820
35	1	0	2.726996	0.729436	2.425897
36	6	0	3.593423	-0.433587	0.825268
37	6	0	4.842074	0.165472	0.979490
38	6	0	3.396401	-1.355134	-0.206591
39	6	0	5.888376	-0.153701	0.115770
40	1	0	4.997362	0.885164	1.779844
41	6	0	4.439329	-1.672758	-1.069327
42	1	0	2.424110	-1.829142	-0.330086
43	6	0	5.687839	-1.071780	-0.909980
44	1	0	6.856971	0.319670	0.242474
45	1	0	4.279781	-2.393093	-1.865338
46	1	0	6.500811	-1.319792	-1.584932

G1-1b-P-*S*

Zero-point correction= 0.36911 (a.u.)
Thermal correction to Gibbs Free Energy= 0.32757 (a.u.)
Sum of electronic and zero-point Energies= -1274.96297 (a.u.)
Sum of electronic and thermal Free Energies= -1275.00450 (a.u.)

Standard orientation:

Center Number	Atomic Number	Atomic Type	Coordinates (Angstroms) X	Y	Z
1	1	0	2.180927	-0.926954	1.505582
2	6	0	0.781850	-0.911220	-1.213762
3	1	0	1.575258	-0.610895	-1.898190
4	1	0	-0.207439	-0.797968	-1.654766
5	7	0	0.968720	-2.365765	-0.938403
6	8	0	-0.012416	-3.082723	-0.988106
7	8	0	2.097479	-2.736200	-0.670579
8	8	0	4.365489	-0.517289	0.808676
9	8	0	-0.097621	-1.649226	1.696956
10	7	0	-1.213693	0.279985	1.072390
11	7	0	2.164415	-0.229800	0.768036
12	8	0	3.434331	0.487310	-0.996640
13	1	0	-2.698430	0.926953	2.383815
14	6	0	5.666185	-0.361868	0.212574
15	6	0	0.896084	-0.088327	0.096317
16	6	0	-0.183236	-0.613294	1.071195
17	6	0	0.399300	1.310639	-0.206777

18	6	0	-0.884428	1.434737	0.340743
19	6	0	0.937042	2.337431	-0.962253
20	1	0	1.926725	2.234008	-1.392844
21	6	0	3.338732	-0.047916	0.092743
22	6	0	0.177716	3.498839	-1.143305
23	1	0	0.586234	4.320019	-1.722324
24	6	0	-2.470133	0.054690	1.762689
25	1	0	-2.300408	-0.800876	2.422801
26	6	0	-1.658077	2.567790	0.153600
27	1	0	-2.659419	2.641401	0.564135
28	6	0	-1.098894	3.605046	-0.598585
29	1	0	-1.677536	4.508604	-0.762497
30	6	0	-3.599420	-0.223719	0.793098
31	6	0	-4.803093	0.472492	0.879420
32	6	0	-3.437917	-1.195767	-0.198301
33	6	0	-5.838245	0.204513	-0.014831
34	1	0	-4.931623	1.229248	1.649847
35	6	0	-4.468644	-1.461040	-1.092487
36	1	0	-2.503581	-1.750609	-0.265214
37	6	0	-5.671099	-0.760378	-1.003129
38	1	0	-6.771719	0.753604	0.058755
39	1	0	-4.335654	-2.218704	-1.858128
40	1	0	-6.474267	-0.967242	-1.703203
41	6	0	6.194947	1.043026	0.413967
42	1	0	5.563620	1.766663	-0.106034
43	1	0	6.224565	1.294123	1.477249
44	1	0	7.209320	1.118341	0.012558
45	1	0	6.285715	-1.101904	0.721749
46	1	0	5.601506	-0.617771	-0.847411

G2

Zero-point correction= 0.72456 (a.u.)
Thermal correction to Gibbs Free Energy= 0.67399 (a.u.)
Sum of electronic and zero-point Energies= -1538.88204 (a.u.)
Sum of electronic and thermal Free Energies= -1538.93260 (a.u.)

Standard orientation:

Center Number	Atomic Number	Atomic Type	Coordinates (Angstroms) X	Y	Z
1	6	0	-0.360653	4.817667	0.554509
2	6	0	1.071009	4.894879	0.039159
3	6	0	1.813118	3.605576	0.358448

4	7	0	1.154381	2.435577	-0.239973
5	6	0	-1.025489	3.585825	-0.041910
6	6	0	-0.227149	2.296599	0.237416
7	1	0	-0.929057	5.715963	0.293925
8	1	0	-0.352814	4.743241	1.650198
9	1	0	1.608998	5.733299	0.493252
10	1	0	1.065993	5.051721	-1.046060
11	1	0	2.832761	3.637523	-0.025793
12	1	0	1.895455	3.483078	1.450853
13	1	0	-2.043672	3.453571	0.341004
14	1	0	-1.095059	3.685810	-1.130492
15	1	0	-0.225044	2.109226	1.327764
16	6	0	1.980591	1.271230	-0.229499
17	7	0	3.010947	1.271018	0.530039
18	7	0	1.554059	0.335403	-1.141011
19	6	0	1.792619	-1.099597	-1.148235
20	6	0	-1.056216	1.212488	-0.455289
21	8	0	-1.189063	1.150460	-1.671733
22	7	0	-1.760304	0.421833	0.391488
23	1	0	-1.560265	0.445178	1.381561
24	6	0	-2.790103	-0.480207	-0.104465
25	6	0	0.482429	-1.884255	-0.999046
26	6	0	0.745637	-3.392575	-1.024295
27	6	0	1.476777	-3.813042	-2.299791
28	6	0	2.523819	-1.523508	-2.427496
29	1	0	-0.190162	-1.601095	-1.823377
30	1	0	-0.012701	-1.591933	-0.065916
31	1	0	1.359579	-3.659306	-0.152732
32	1	0	-0.194391	-3.944987	-0.921539
33	1	0	2.417740	-1.345120	-0.285949
34	1	0	1.675677	-4.889786	-2.287643
35	1	0	3.460834	-0.962962	-2.515733
36	1	0	1.900504	-1.236820	-3.286495
37	6	0	2.780620	-3.030475	-2.459862
38	1	0	0.831210	-3.616127	-3.166527
39	1	0	3.285037	-3.306817	-3.391432
40	1	0	3.462775	-3.296494	-1.640046
41	1	0	0.851818	0.670874	-1.789982
42	6	0	-4.116572	0.234074	-0.340714
43	6	0	-4.453381	1.401835	0.341742
44	6	0	-5.036048	-0.318534	-1.234595
45	6	0	-5.688274	2.012489	0.131367
46	1	0	-3.742124	1.842470	1.034685
47	6	0	-6.267653	0.290683	-1.448593

48	1	0	-4.779032	-1.229963	-1.769448
49	6	0	-6.597427	1.459823	-0.764944
50	1	0	-5.936004	2.923536	0.667275
51	1	0	-6.969629	-0.143367	-2.153741
52	1	0	-7.556715	1.938643	-0.933788
53	6	0	-2.941649	-1.652438	0.848603
54	6	0	-2.610841	-2.941022	0.432841
55	6	0	-3.399927	-1.453818	2.153741
56	6	0	-2.718142	-4.017498	1.311021
57	1	0	-2.265689	-3.097235	-0.586060
58	6	0	-3.501432	-2.525542	3.034216
59	1	0	-3.693829	-0.456943	2.475608
60	6	0	-3.158420	-3.810054	2.614653
61	1	0	-2.455230	-5.016075	0.975805
62	1	0	-3.856586	-2.361061	4.046727
63	1	0	-3.240267	-4.645869	3.302211
64	1	0	-2.435769	-0.855855	-1.069741
65	6	0	4.120717	0.350136	0.400865
66	6	0	5.418938	1.168732	0.358394
67	6	0	4.169610	-0.616290	1.592547
68	1	0	4.086103	-0.238785	-0.529966
69	6	0	6.654928	0.270530	0.291953
70	1	0	5.453406	1.794828	1.260131
71	1	0	5.383867	1.848165	-0.500671
72	6	0	5.397993	-1.525890	1.534367
73	1	0	4.193237	-0.010889	2.508682
74	1	0	3.248496	-1.209912	1.637603
75	6	0	6.689066	-0.707506	1.468139
76	1	0	7.565030	0.879752	0.274318
77	1	0	6.638683	-0.299116	-0.647878
78	1	0	5.413344	-2.195766	2.400929
79	1	0	5.332537	-2.165640	0.642475
80	1	0	7.557767	-1.370381	1.391756
81	1	0	6.801141	-0.140949	2.402821

G2-COM

Zero-point correction= 0.77873 (a.u.)

Thermal correction to Gibbs Free Energy= 0.72627 (a.u.)

Sum of electronic and zero-point Energies= -1783.76078 (a.u.)

Sum of electronic and thermal Free Energies= -1783.81324 (a.u.)

Standard orientation:

Center Atomic Atomic Coordinates (Angstroms)

Number	Number	Type	X	Y	Z
1	6	0	0.611260	4.622819	-1.138965
2	6	0	1.802847	4.285860	-0.239725
3	6	0	1.445124	3.223640	0.804870
4	7	0	0.872530	2.026135	0.173902
5	6	0	-0.019055	3.356892	-1.726784
6	6	0	-0.330750	2.391023	-0.582109
7	1	0	0.916682	5.305408	-1.937977
8	1	0	-0.149790	5.148324	-0.545655
9	1	0	2.167538	5.185985	0.267030
10	1	0	2.630508	3.907223	-0.852590
11	1	0	2.327409	2.924917	1.367705
12	1	0	0.697815	3.611813	1.509829
13	1	0	-0.937990	3.591241	-2.271187
14	1	0	0.663655	2.882790	-2.442736
15	1	0	-0.945984	2.943559	0.145301
16	6	0	1.771953	1.011878	-0.206798
17	7	0	2.766398	0.801235	0.583872
18	7	0	1.485023	0.351016	-1.381526
19	6	0	1.624569	-1.092925	-1.578649
20	6	0	-1.225577	1.223215	-1.023785
21	8	0	-1.427698	0.976388	-2.209096
22	7	0	-1.820484	0.558821	-0.014725
23	1	0	-1.566288	0.777144	0.944412
24	6	0	-2.788812	-0.496639	-0.258841
25	6	0	0.274892	-1.807119	-1.451812
26	6	0	0.425160	-3.312510	-1.684136
27	6	0	1.071192	-3.603269	-3.039735
28	6	0	2.269671	-1.384322	-2.934633
29	1	0	-0.415923	-1.379418	-2.191949
30	1	0	-0.141206	-1.602490	-0.457786
31	1	0	1.047003	-3.740405	-0.885151
32	1	0	-0.551451	-3.805529	-1.619426
33	1	0	2.273804	-1.475756	-0.789482
34	1	0	1.197802	-4.682195	-3.178863
35	1	0	3.240849	-0.880148	-2.994485
36	1	0	1.634615	-0.949794	-3.720353
37	6	0	2.417543	-2.889030	-3.167926
38	1	0	0.402141	-3.254186	-3.837830
39	1	0	2.859931	-3.074782	-4.152028
40	1	0	3.115288	-3.300044	-2.424716
41	1	0	0.778202	0.760402	-1.979976
42	6	0	-4.167755	0.043901	-0.626582

43	6	0	-4.525263	1.376503	-0.436616
44	6	0	-5.114360	-0.844963	-1.143227
45	6	0	-5.809368	1.815411	-0.758031
46	1	0	-3.796466	2.077118	-0.039632
47	6	0	-6.393216	-0.409138	-1.466741
48	1	0	-4.841627	-1.887974	-1.288450
49	6	0	-6.745934	0.926222	-1.273568
50	1	0	-6.073866	2.857573	-0.606192
51	1	0	-7.116235	-1.110322	-1.871949
52	1	0	-7.744007	1.268883	-1.527893
53	6	0	-2.852129	-1.394044	0.965042
54	6	0	-2.437089	-2.721324	0.893705
55	6	0	-3.302902	-0.883223	2.185171
56	6	0	-2.459554	-3.531177	2.028028
57	1	0	-2.087647	-3.119827	-0.055180
58	6	0	-3.319715	-1.687155	3.318974
59	1	0	-3.645300	0.147649	2.242192
60	6	0	-2.897073	-3.013980	3.242700
61	1	0	-2.132022	-4.563927	1.960093
62	1	0	-3.670233	-1.281687	4.263091
63	1	0	-2.912989	-3.641612	4.128186
64	1	0	-2.428050	-1.085140	-1.109770
65	6	0	3.931117	0.031180	0.205456
66	6	0	5.178078	0.910158	0.375810
67	6	0	4.075244	-1.219157	1.085008
68	1	0	3.906870	-0.280375	-0.851642
69	6	0	6.457526	0.146238	0.031865
70	1	0	5.212182	1.254126	1.419145
71	1	0	5.075818	1.803113	-0.251336
72	6	0	5.340945	-2.004329	0.738293
73	1	0	4.119599	-0.886824	2.132077
74	1	0	3.182008	-1.849543	1.001829
75	6	0	6.586753	-1.125694	0.873461
76	1	0	7.332955	0.788824	0.174910
77	1	0	6.438150	-0.127912	-1.032088
78	1	0	5.425901	-2.889674	1.377567
79	1	0	5.266151	-2.368198	-0.296495
80	1	0	7.482837	-1.685997	0.585508
81	1	0	6.711877	-0.845592	1.928572
82	1	0	2.128893	1.054780	2.679857
83	6	0	1.353499	1.032086	3.456437
84	1	0	1.204424	1.991732	3.943257
85	1	0	1.549232	0.229845	4.167002
86	7	0	0.110999	0.676208	2.734876

87	8	0	0.058799	-0.424328	2.228563
88	8	0	-0.784509	1.510545	2.668473

G2-TS1

Zero-point correction= 0.77423 (a.u.)
Thermal correction to Gibbs Free Energy= 0.72297 (a.u.)
Sum of electronic and zero-point Energies= -1783.74480 (a.u.)
Sum of electronic and thermal Free Energies= -1783.79605 (a.u.)

Standard orientation:

Center Number	Atomic Number	Atomic Type	Coordinates (Angstroms) X	Y	Z
1	6	0	0.335427	4.816629	-0.807970
2	6	0	1.666238	4.479521	-0.133175
3	6	0	1.548532	3.266701	0.793980
4	7	0	0.967849	2.127269	0.069599
5	6	0	-0.286550	3.581359	-1.464347
6	6	0	-0.372953	2.459575	-0.429976
7	1	0	0.473222	5.613510	-1.544815
8	1	0	-0.363645	5.198238	-0.052008
9	1	0	2.029554	5.335120	0.445863
10	1	0	2.423044	4.263702	-0.897320
11	1	0	2.525002	2.978217	1.177840
12	1	0	0.894499	3.480399	1.649038
13	1	0	-1.287187	3.802385	-1.846914
14	1	0	0.316339	3.241290	-2.314977
15	1	0	-0.895529	2.850203	0.455183
16	6	0	1.730806	1.048936	-0.276520
17	7	0	2.708851	0.735505	0.546887
18	7	0	1.432709	0.384288	-1.420135
19	6	0	1.566980	-1.063140	-1.610376
20	6	0	-1.236732	1.287609	-0.925822
21	8	0	-1.422296	1.087230	-2.124334
22	7	0	-1.824469	0.586435	0.057319
23	1	0	-1.519615	0.743778	1.020542
24	6	0	-2.781501	-0.470856	-0.220624
25	6	0	0.211021	-1.761391	-1.482943
26	6	0	0.354693	-3.267841	-1.712813
27	6	0	1.006949	-3.567447	-3.063421
28	6	0	2.220417	-1.357988	-2.960572
29	1	0	-0.474514	-1.328634	-2.224596
30	1	0	-0.197626	-1.551901	-0.486761

31	1	0	0.967123	-3.697373	-0.907752
32	1	0	-0.625668	-3.752215	-1.651274
33	1	0	2.207490	-1.442492	-0.812428
34	1	0	1.126959	-4.647568	-3.197792
35	1	0	3.195368	-0.860160	-3.016192
36	1	0	1.593145	-0.923025	-3.751977
37	6	0	2.359727	-2.864627	-3.185965
38	1	0	0.346071	-3.216689	-3.867542
39	1	0	2.807306	-3.055941	-4.166418
40	1	0	3.049867	-3.277033	-2.436508
41	1	0	0.758989	0.823802	-2.039675
42	6	0	-4.176244	0.070646	-0.529432
43	6	0	-4.536640	1.396014	-0.299694
44	6	0	-5.132428	-0.813160	-1.037537
45	6	0	-5.832338	1.832613	-0.574853
46	1	0	-3.802319	2.092265	0.094380
47	6	0	-6.422625	-0.379700	-1.314573
48	1	0	-4.858830	-1.851389	-1.212252
49	6	0	-6.778025	0.948856	-1.082652
50	1	0	-6.099120	2.869026	-0.390941
51	1	0	-7.152645	-1.077630	-1.712829
52	1	0	-7.785519	1.289939	-1.299335
53	6	0	-2.816436	-1.434754	0.951956
54	6	0	-2.545124	-2.787601	0.764858
55	6	0	-3.114535	-0.968855	2.234781
56	6	0	-2.562607	-3.668513	1.845004
57	1	0	-2.312251	-3.151800	-0.232661
58	6	0	-3.125837	-1.843710	3.313599
59	1	0	-3.332989	0.085412	2.389527
60	6	0	-2.849812	-3.197223	3.121222
61	1	0	-2.345757	-4.720397	1.686499
62	1	0	-3.351459	-1.470719	4.307755
63	1	0	-2.859227	-3.879822	3.965089
64	1	0	-2.436608	-1.008988	-1.110351
65	6	0	3.913099	0.003583	0.193846
66	6	0	5.137280	0.907808	0.391715
67	6	0	4.066415	-1.263385	1.045085
68	1	0	3.886720	-0.279171	-0.868144
69	6	0	6.431547	0.172265	0.038970
70	1	0	5.162264	1.229667	1.442360
71	1	0	5.023978	1.810766	-0.218721
72	6	0	5.347760	-2.014329	0.680914
73	1	0	4.105423	-0.961553	2.100898
74	1	0	3.183851	-1.904409	0.939729

75	6	0	6.576643	-1.118095	0.848801
76	1	0	7.293200	0.827202	0.204138
77	1	0	6.422244	-0.074415	-1.031703
78	1	0	5.443373	-2.915758	1.294785
79	1	0	5.283333	-2.348517	-0.364465
80	1	0	7.483653	-1.654783	0.551516
81	1	0	6.690242	-0.864255	1.911472
82	1	0	2.353376	0.869647	1.763514
83	6	0	1.668651	0.799308	3.020589
84	1	0	1.660337	1.688093	3.643564
85	1	0	2.092240	-0.090412	3.480413
86	7	0	0.371650	0.508892	2.547784
87	8	0	0.164767	-0.589583	2.028347
88	8	0	-0.508050	1.395455	2.562204

G2-IM1

Zero-point correction= 0.77827 (a.u.)

Thermal correction to Gibbs Free Energy= 0.72557 (a.u.)

Sum of electronic and zero-point Energies= -1783.76165 (a.u.)

Sum of electronic and thermal Free Energies= -1783.81434 (a.u.)

Standard orientation:

Center Number	Atomic Number	Atomic Type	Coordinates (Angstroms) X	Y	Z
1	6	0	0.462570	4.456126	-2.112415
2	6	0	1.646529	4.296089	-1.148835
3	6	0	1.330920	3.334745	0.002179
4	7	0	0.837861	2.071032	-0.562874
5	6	0	-0.106553	3.105435	-2.572318
6	6	0	-0.397441	2.275598	-1.327319
7	1	0	0.763483	5.057333	-2.975340
8	1	0	-0.337375	5.008663	-1.603234
9	1	0	1.928166	5.269285	-0.733762
10	1	0	2.519129	3.916126	-1.694760
11	1	0	2.212041	3.141030	0.614602
12	1	0	0.541344	3.729410	0.650144
13	1	0	-1.025299	3.244175	-3.148571
14	1	0	0.607940	2.583115	-3.221480
15	1	0	-1.023748	2.872711	-0.651262
16	6	0	1.617266	0.976577	-0.663626
17	7	0	2.406931	0.701401	0.374311
18	7	0	1.559304	0.197325	-1.759889

19	6	0	1.673494	-1.273280	-1.746871
20	6	0	-1.179595	0.971264	-1.536394
21	8	0	-1.337563	0.483436	-2.654620
22	7	0	-1.656096	0.478398	-0.381518
23	1	0	-1.438740	1.015693	0.480828
24	6	0	-2.644514	-0.587267	-0.348275
25	6	0	0.642332	-1.901241	-0.813812
26	6	0	0.769069	-3.422357	-0.810168
27	6	0	0.592129	-3.969117	-2.228648
28	6	0	1.483762	-1.789310	-3.172113
29	1	0	-0.349914	-1.622897	-1.186881
30	1	0	0.731724	-1.485696	0.195073
31	1	0	1.757929	-3.713184	-0.426100
32	1	0	0.023810	-3.847040	-0.129436
33	1	0	2.680928	-1.549314	-1.415423
34	1	0	0.715142	-5.056929	-2.241959
35	1	0	2.230068	-1.339999	-3.836999
36	1	0	0.490913	-1.471584	-3.520905
37	6	0	1.581167	-3.317594	-3.200203
38	1	0	-0.433190	-3.758315	-2.561805
39	1	0	1.410297	-3.677238	-4.219597
40	1	0	2.602859	-3.614839	-2.926812
41	1	0	0.994393	0.556978	-2.523079
42	6	0	-4.027269	-0.019024	-0.045658
43	6	0	-4.203618	0.867232	1.021011
44	6	0	-5.128207	-0.394792	-0.813114
45	6	0	-5.469039	1.366373	1.309194
46	1	0	-3.346563	1.175311	1.618225
47	6	0	-6.396884	0.102720	-0.520257
48	1	0	-4.990860	-1.074560	-1.650568
49	6	0	-6.569261	0.984915	0.541213
50	1	0	-5.597361	2.058285	2.135995
51	1	0	-7.246952	-0.193465	-1.127374
52	1	0	-7.555580	1.377252	0.769292
53	6	0	-2.294196	-1.688407	0.645042
54	6	0	-2.839124	-2.960888	0.455038
55	6	0	-1.471302	-1.455727	1.747987
56	6	0	-2.567721	-3.990875	1.350956
57	1	0	-3.482346	-3.145368	-0.402844
58	6	0	-1.193331	-2.491907	2.638645
59	1	0	-1.008597	-0.483349	1.910108
60	6	0	-1.739055	-3.758509	2.447220
61	1	0	-2.997894	-4.974782	1.189612
62	1	0	-0.542821	-2.298605	3.486603

63	1	0	-1.520597	-4.560523	3.145690
64	1	0	-2.664654	-1.018742	-1.354409
65	6	0	3.697007	0.022588	0.370963
66	6	0	4.766227	0.968675	0.932340
67	6	0	3.643186	-1.273095	1.185120
68	1	0	3.957058	-0.200079	-0.670663
69	6	0	6.136625	0.289593	0.978022
70	1	0	4.464869	1.267554	1.946272
71	1	0	4.800238	1.878466	0.322558
72	6	0	5.013147	-1.952418	1.229383
73	1	0	3.315658	-1.021097	2.203131
74	1	0	2.887537	-1.947803	0.770163
75	6	0	6.083091	-1.009681	1.783271
76	1	0	6.877226	0.975570	1.401002
77	1	0	6.460967	0.066327	-0.047617
78	1	0	4.956059	-2.865106	1.830488
79	1	0	5.294254	-2.260786	0.212349
80	1	0	7.061945	-1.500114	1.778338
81	1	0	5.848131	-0.775338	2.829993
82	1	0	1.968656	0.976104	1.278199
83	6	0	0.190272	2.979491	3.367099
84	1	0	-0.463216	3.832210	3.456349
85	1	0	1.058433	2.810892	3.983297
86	7	0	-0.112974	2.068782	2.484464
87	8	0	0.617712	1.010924	2.339630
88	8	0	-1.153995	2.202403	1.729136

G2-1a-I-*re*-IM2

Zero-point correction= 1.14972 (a.u.)

Thermal correction to Gibbs Free Energy= 1.08117 (a.u.)

Sum of electronic and zero-point Energies= -2892.38831 (a.u.)

Sum of electronic and thermal Free Energies= -2892.45687 (a.u.)

Standard orientation:

Center Number	Atomic Number	Atomic Type	Coordinates (Angstroms) X	Y	Z
1	6	0	1.599513	1.911530	3.792408
2	6	0	2.093810	3.023091	2.873244
3	6	0	1.757466	2.704514	1.422930
4	7	0	0.297400	2.540297	1.262878
5	6	0	0.102892	1.705481	3.578970
6	6	0	-0.212543	1.425586	2.103164

7	1	0	1.802445	2.148838	4.841983
8	1	0	2.138313	0.986795	3.548849
9	1	0	3.179256	3.144249	2.953675
10	1	0	1.630881	3.977790	3.152218
11	1	0	2.077611	3.514972	0.766628
12	1	0	2.264001	1.788389	1.098167
13	1	0	-0.262915	0.859517	4.171568
14	1	0	-0.465141	2.591644	3.882935
15	1	0	0.331567	0.528067	1.780641
16	6	0	-0.261213	2.905056	0.067630
17	7	0	0.517251	3.015213	-0.993853
18	7	0	-1.572940	3.206133	0.049037
19	6	0	-2.494824	2.931374	-1.059484
20	1	0	1.330615	2.330371	-1.047126
21	6	0	-1.718381	1.150285	2.024327
22	8	0	-2.561339	1.971865	2.382187
23	7	0	-2.033403	-0.106740	1.652010
24	1	0	-1.317721	-0.682972	1.217273
25	6	0	-2.327707	1.509400	-1.598986
26	6	0	-3.341034	1.233808	-2.710720
27	6	0	-4.773804	1.479959	-2.230891
28	6	0	-3.918671	3.165938	-0.555065
29	1	0	-2.493376	0.819972	-0.759697
30	1	0	-1.300444	1.334490	-1.941869
31	1	0	-3.127498	1.891300	-3.565230
32	1	0	-3.221187	0.207161	-3.074541
33	1	0	-2.314144	3.647007	-1.873267
34	1	0	-5.485386	1.314807	-3.046457
35	1	0	-4.012309	4.194406	-0.187612
36	1	0	-4.102004	2.500072	0.299932
37	6	0	-4.932394	2.894008	-1.667363
38	1	0	-5.023341	0.754988	-1.442266
39	1	0	-5.947922	3.044391	-1.287955
40	1	0	-4.783760	3.623990	-2.474657
41	1	0	-2.000682	3.254629	0.969781
42	6	0	-4.064409	-0.606757	2.974505
43	6	0	-3.422874	-0.549095	1.597226
44	6	0	-3.488507	-1.885605	0.861206
45	6	0	-3.314825	-0.852122	4.124020
46	6	0	-3.940181	-0.948959	5.365069
47	6	0	-5.320303	-0.799238	5.467207
48	6	0	-6.073477	-0.552710	4.321217
49	6	0	-5.447302	-0.459584	3.082633
50	1	0	-2.237155	-0.965880	4.042960

51	1	0	-3.346575	-1.139378	6.254053
52	1	0	-5.806650	-0.870435	6.435013
53	1	0	-7.149701	-0.428899	4.392792
54	1	0	-6.034136	-0.264587	2.187908
55	6	0	-3.439716	-3.103705	1.542343
56	6	0	-3.503464	-4.306859	0.841952
57	6	0	-3.584209	-1.897092	-0.533708
58	6	0	-3.651402	-3.097121	-1.235560
59	6	0	-3.617411	-4.307480	-0.546352
60	1	0	-3.474344	-5.245702	1.386496
61	1	0	-3.374277	-3.112957	2.626012
62	1	0	-3.675936	-5.245370	-1.090481
63	1	0	-3.720305	-3.085090	-2.319393
64	1	0	-3.612461	-0.956866	-1.075855
65	1	0	-3.985070	0.187756	1.005474
66	8	0	4.329651	0.304316	2.365223
67	8	0	0.167781	-1.114380	0.174729
68	7	0	0.989945	-3.147484	-0.524954
69	7	0	2.853088	-0.600650	0.970029
70	8	0	5.072084	-0.365271	0.329412
71	6	0	5.635853	0.765668	2.826851
72	6	0	2.557293	-1.655072	0.316124
73	6	0	1.089085	-1.888648	-0.010784
74	6	0	3.254702	-2.881472	-0.127377
75	6	0	2.254513	-3.744269	-0.622373
76	6	0	4.578511	-3.305276	-0.107998
77	1	0	5.358443	-2.648476	0.255948
78	6	0	4.194900	-0.249991	1.158407
79	6	0	4.885635	-4.578357	-0.591000
80	1	0	5.915685	-4.917377	-0.587056
81	6	0	-0.274398	-3.740791	-0.918422
82	1	0	-1.046135	-3.216733	-0.344272
83	6	0	2.549278	-5.001182	-1.121608
84	1	0	1.775459	-5.642017	-1.530144
85	6	0	3.885836	-5.407284	-1.094915
86	1	0	4.146551	-6.387624	-1.481203
87	6	0	5.347592	1.216652	4.253169
88	6	0	6.637879	-0.385097	2.827044
89	6	0	6.099854	1.935819	1.967645
90	1	0	6.290105	1.620598	0.941248
91	1	0	7.021371	2.351558	2.386156
92	1	0	5.341165	2.724506	1.960115
93	1	0	6.261020	1.598986	4.716298
94	1	0	4.974780	0.380566	4.851653

95	1	0	4.594845	2.010030	4.260488
96	1	0	7.537900	-0.075931	3.366472
97	1	0	6.921190	-0.671967	1.814284
98	1	0	6.215091	-1.253459	3.342220
99	1	0	-0.280309	-4.790990	-0.609327
100	6	0	-0.557364	-3.604576	-2.397301
101	6	0	-0.954031	-4.705250	-3.153949
102	6	0	-0.466643	-2.346282	-2.999922
103	6	0	-1.262309	-4.556126	-4.505856
104	1	0	-1.025969	-5.684101	-2.684467
105	6	0	-0.763821	-2.202087	-4.351289
106	1	0	-0.159495	-1.470040	-2.429479
107	6	0	-1.166960	-3.303973	-5.106526
108	1	0	-1.568219	-5.419263	-5.089149
109	1	0	-0.674510	-1.220874	-4.808194
110	1	0	-1.400412	-3.187394	-6.160407
111	6	0	2.745413	-0.735355	-2.247401
112	1	0	2.345631	-1.450438	-2.950456
113	1	0	3.756398	-0.739620	-1.871560
114	7	0	2.022431	0.340426	-1.999365
115	8	0	2.502200	1.253391	-1.222011
116	8	0	0.862682	0.503898	-2.496516
117	6	0	0.245565	3.757335	-2.224781
118	6	0	-0.125630	5.218543	-1.948863
119	6	0	1.495346	3.683883	-3.106267
120	1	0	-0.568312	3.269857	-2.781667
121	6	0	-0.358529	5.968860	-3.261985
122	1	0	0.707302	5.679452	-1.400347
123	1	0	-1.008272	5.287161	-1.305123
124	6	0	1.277534	4.441305	-4.416212
125	1	0	2.340095	4.118901	-2.554626
126	1	0	1.727023	2.633032	-3.294599
127	6	0	0.873191	5.894808	-4.165233
128	1	0	-0.621587	7.010698	-3.052552
129	1	0	-1.218278	5.523979	-3.783178
130	1	0	2.186038	4.395000	-5.024739
131	1	0	0.486589	3.940683	-4.991199
132	1	0	0.682882	6.409275	-5.113148
133	1	0	1.704866	6.420725	-3.677228

G2-1a-I-*si*-IM2

Zero-point correction= 1.14868 (a.u.)

Thermal correction to Gibbs Free Energy= 1.08030 (a.u.)

Sum of electronic and zero-point Energies= -2892.38852 (a.u.)
Sum of electronic and thermal Free Energies= -2892.45690 (a.u.)

Standard orientation:

Center Number	Atomic Number	Atomic Type	Coordinates (Angstroms) X	Y	Z
1	6	0	0.238580	2.472359	-4.624341
2	6	0	-0.251075	3.400809	-3.507409
3	6	0	-0.637783	2.625822	-2.244444
4	7	0	0.489441	1.770331	-1.835218
5	6	0	1.337467	1.521390	-4.134134
6	6	0	0.825794	0.803147	-2.887145
7	1	0	0.594775	3.060221	-5.475700
8	1	0	-0.606916	1.870890	-4.984104
9	1	0	-1.112404	3.985261	-3.847175
10	1	0	0.540919	4.115975	-3.248801
11	1	0	-0.907012	3.293430	-1.428847
12	1	0	-1.491907	1.963952	-2.420258
13	1	0	1.594879	0.786653	-4.902281
14	1	0	2.253050	2.081189	-3.905670
15	1	0	-0.137189	0.341001	-3.137109
16	6	0	1.384478	2.205955	-0.905423
17	7	0	0.907057	2.881117	0.126244
18	7	0	2.693166	1.935892	-1.084300
19	6	0	3.719455	1.778140	-0.049089
20	1	0	-0.118705	2.667165	0.378371
21	6	0	1.723191	-0.350329	-2.395218
22	8	0	2.878378	-0.464686	-2.793805
23	7	0	1.069805	-1.174827	-1.557526
24	1	0	0.156552	-0.853724	-1.226978
25	6	0	3.323844	0.800742	1.060746
26	6	0	4.448571	0.757309	2.098975
27	6	0	5.758869	0.294979	1.458198
28	6	0	5.004531	1.308625	-0.734529
29	1	0	3.182227	-0.190162	0.603676
30	1	0	2.363920	1.079865	1.510518
31	1	0	4.583104	1.764104	2.520963
32	1	0	4.179354	0.107907	2.936967
33	1	0	3.925485	2.756851	0.404108
34	1	0	6.564573	0.279151	2.199631
35	1	0	5.272576	2.011423	-1.532268
36	1	0	4.811525	0.335099	-1.205708
37	6	0	6.142674	1.194520	0.281426

38	1	0	5.634454	-0.735723	1.097955
39	1	0	7.042468	0.815950	-0.212991
40	1	0	6.385859	2.196252	0.661002
41	1	0	2.950845	1.526050	-1.974828
42	6	0	2.696014	-3.072500	-1.636901
43	6	0	1.558633	-2.383659	-0.902521
44	6	0	0.382442	-3.337879	-0.715573
45	6	0	2.530999	-3.550473	-2.938095
46	6	0	3.567303	-4.219720	-3.575597
47	6	0	4.777964	-4.431749	-2.914339
48	6	0	4.943886	-3.968306	-1.614370
49	6	0	3.903521	-3.290557	-0.979744
50	1	0	1.585620	-3.389684	-3.450621
51	1	0	3.433161	-4.582088	-4.590224
52	1	0	5.585691	-4.957859	-3.413462
53	1	0	5.881510	-4.131420	-1.091758
54	1	0	4.028892	-2.925193	0.037363
55	6	0	-0.709607	-3.347992	-1.583739
56	6	0	-1.809411	-4.164480	-1.324650
57	6	0	0.381365	-4.201659	0.380676
58	6	0	-0.713002	-5.019956	0.640255
59	6	0	-1.820782	-4.992810	-0.205118
60	1	0	-2.664445	-4.144546	-1.994288
61	1	0	-0.726996	-2.675590	-2.436352
62	1	0	-2.686293	-5.611870	0.008887
63	1	0	-0.707270	-5.670265	1.509907
64	1	0	1.235419	-4.201379	1.052722
65	1	0	1.910673	-2.119201	0.103595
66	6	0	-2.785650	1.101184	2.088937
67	1	0	-2.762161	0.398841	2.907501
68	1	0	-3.683307	1.516825	1.658444
69	7	0	-1.629203	1.558244	1.669923
70	8	0	-1.595610	2.441757	0.706118
71	8	0	-0.528957	1.157046	2.158069
72	8	0	0.800934	-1.846013	2.277226
73	8	0	-1.744606	-0.269496	-1.501378
74	7	0	-3.865892	-0.848667	-0.823240
75	7	0	-0.922368	-1.279347	0.993734
76	8	0	-1.200813	-1.589889	3.295353
77	1	0	-5.408878	-0.930374	-2.219047
78	6	0	1.527059	-2.082083	3.515302
79	6	0	-2.157721	-1.328045	0.681745
80	6	0	-2.514214	-0.747421	-0.684983
81	6	0	-3.439233	-1.819041	1.239047

82	6	0	-4.433050	-1.474809	0.299391
83	6	0	-3.802358	-2.521927	2.382626
84	1	0	-3.052565	-2.798907	3.110490
85	6	0	-0.505614	-1.584820	2.303697
86	6	0	-5.146672	-2.841258	2.583239
87	1	0	-5.436997	-3.386010	3.474970
88	6	0	-4.594558	-0.259156	-1.929963
89	1	0	-3.886539	-0.210572	-2.763022
90	6	0	-5.772529	-1.758356	0.500173
91	1	0	-6.527574	-1.448945	-0.214633
92	6	0	-6.116582	-2.451298	1.663855
93	1	0	-7.159734	-2.687624	1.848962
94	6	0	-5.117072	1.122967	-1.595802
95	6	0	-6.422213	1.496250	-1.911274
96	6	0	-4.271064	2.042511	-0.969927
97	6	0	-6.879374	2.778335	-1.608736
98	1	0	-7.087365	0.781801	-2.391122
99	6	0	-4.731483	3.316887	-0.658062
100	1	0	-3.248774	1.781069	-0.701649
101	6	0	-6.036456	3.689677	-0.979194
102	1	0	-7.898835	3.058726	-1.855555
103	1	0	-4.060313	4.006459	-0.154560
104	1	0	-6.396319	4.684102	-0.733631
105	6	0	0.917779	-3.231798	4.315123
106	1	0	0.691858	-4.071522	3.649334
107	1	0	1.646859	-3.574864	5.055607
108	1	0	0.004507	-2.933972	4.827598
109	6	0	1.565709	-0.779976	4.305554
110	1	0	2.016271	0.014333	3.703948
111	1	0	0.556726	-0.468665	4.580985
112	1	0	2.158263	-0.915759	5.215569
113	6	0	2.913267	-2.480660	3.019908
114	1	0	3.614374	-2.531011	3.857976
115	1	0	2.885479	-3.463955	2.538455
116	1	0	3.283197	-1.748891	2.296382
117	6	0	1.621180	3.768398	1.038683
118	6	0	2.310353	4.924465	0.306282
119	6	0	0.610699	4.302327	2.057571
120	1	0	2.378887	3.210932	1.608118
121	6	0	3.003663	5.852558	1.306022
122	1	0	1.541670	5.478472	-0.249984
123	1	0	3.028120	4.544168	-0.430010
124	6	0	1.295840	5.235965	3.054563
125	1	0	-0.181316	4.839799	1.518832

126	1	0	0.140381	3.451787	2.560128
127	6	0	2.012936	6.385029	2.342823
128	1	0	3.489230	6.678335	0.776042
129	1	0	3.800536	5.296123	1.820662
130	1	0	0.559417	5.624706	3.764887
131	1	0	2.027130	4.662896	3.641496
132	1	0	2.528711	7.023387	3.068134
133	1	0	1.268623	7.013855	1.835491

G2-1a-I-*re*-TS2

Zero-point correction= 1.14960 (a.u.)

Thermal correction to Gibbs Free Energy= 1.08205 (a.u.)

Sum of electronic and zero-point Energies= -2892.38788 (a.u.)

Sum of electronic and thermal Free Energies= -2892.45542 (a.u.)

Standard orientation:

Center Number	Atomic Number	Atomic Type	Coordinates (Angstroms) X	Y	Z
1	6	0	1.710724	1.898811	3.762579
2	6	0	2.219279	3.001404	2.840747
3	6	0	1.876362	2.681006	1.392793
4	7	0	0.412216	2.546139	1.235299
5	6	0	0.209535	1.722120	3.556773
6	6	0	-0.122856	1.447854	2.083398
7	1	0	1.922672	2.132881	4.811067
8	1	0	2.230670	0.964790	3.514880
9	1	0	3.306099	3.108830	2.917300
10	1	0	1.769853	3.963609	3.116441
11	1	0	2.211691	3.482520	0.732847
12	1	0	2.363759	1.750777	1.077449
13	1	0	-0.169381	0.882570	4.150231
14	1	0	-0.339836	2.618842	3.864315
15	1	0	0.392399	0.534596	1.756513
16	6	0	-0.144210	2.923104	0.046855
17	7	0	0.632941	3.014814	-1.021651
18	7	0	-1.447252	3.252491	0.025631
19	6	0	-2.379168	2.993197	-1.078948
20	1	0	1.430841	2.341507	-1.059654
21	6	0	-1.636902	1.222898	2.018754
22	8	0	-2.448072	2.078629	2.372206
23	7	0	-1.998809	-0.026783	1.667688
24	1	0	-1.309768	-0.628029	1.221657

25	6	0	-2.261446	1.558927	-1.598991
26	6	0	-3.288203	1.301028	-2.703076
27	6	0	-4.710219	1.608606	-2.226500
28	6	0	-3.792789	3.284019	-0.575826
29	1	0	-2.441889	0.883479	-0.751483
30	1	0	-1.242289	1.350389	-1.946354
31	1	0	-3.053045	1.933203	-3.570824
32	1	0	-3.207280	0.263439	-3.045893
33	1	0	-2.175754	3.690885	-1.902399
34	1	0	-5.427521	1.457069	-3.039603
35	1	0	-3.849030	4.319361	-0.220573
36	1	0	-3.998522	2.635079	0.287060
37	6	0	-4.816172	3.035568	-1.684435
38	1	0	-4.986907	0.906791	-1.426429
39	1	0	-5.825393	3.229259	-1.307997
40	1	0	-4.639951	3.747989	-2.501890
41	1	0	-1.872916	3.313283	0.947135
42	6	0	-4.035771	-0.431520	3.014782
43	6	0	-3.404799	-0.415043	1.631430
44	6	0	-3.531189	-1.756272	0.914946
45	6	0	-3.284155	-0.667061	4.164789
46	6	0	-3.903877	-0.723946	5.411215
47	6	0	-5.279951	-0.544474	5.518081
48	6	0	-6.035239	-0.308407	4.371199
49	6	0	-5.414983	-0.255059	3.127557
50	1	0	-2.209347	-0.803283	4.080740
51	1	0	-3.308561	-0.906495	6.300698
52	1	0	-5.761718	-0.584333	6.489971
53	1	0	-7.108380	-0.161966	4.446128
54	1	0	-6.003340	-0.068880	2.231859
55	6	0	-3.479564	-2.966312	1.610033
56	6	0	-3.601923	-4.175791	0.929084
57	6	0	-3.687698	-1.781737	-0.473980
58	6	0	-3.812385	-2.988168	-1.156442
59	6	0	-3.776080	-4.190210	-0.453024
60	1	0	-3.570395	-5.108615	1.483709
61	1	0	-3.366691	-2.962296	2.689956
62	1	0	-3.880092	-5.133147	-0.981293
63	1	0	-3.927358	-2.988438	-2.236371
64	1	0	-3.718067	-0.846921	-1.025479
65	1	0	-3.944323	0.335483	1.035706
66	8	0	4.332816	0.207536	2.311976
67	8	0	0.116869	-1.072200	0.103604
68	7	0	0.849907	-3.153587	-0.547541

69	7	0	2.820320	-0.686599	0.948086
70	8	0	5.051761	-0.584709	0.313742
71	6	0	5.660904	0.606616	2.754429
72	6	0	2.491050	-1.688332	0.201950
73	6	0	1.011496	-1.881864	-0.077369
74	6	0	3.124042	-2.981138	-0.173490
75	6	0	2.080915	-3.818169	-0.620824
76	6	0	4.423640	-3.472411	-0.140022
77	1	0	5.238138	-2.839484	0.187964
78	6	0	4.163698	-0.393960	1.124354
79	6	0	4.662237	-4.782540	-0.561486
80	1	0	5.674202	-5.172371	-0.542468
81	6	0	-0.445406	-3.705075	-0.892920
82	1	0	-1.183368	-3.128054	-0.324862
83	6	0	2.307382	-5.111687	-1.060455
84	1	0	1.497185	-5.730961	-1.430743
85	6	0	3.621014	-5.584961	-1.021292
86	1	0	3.829225	-6.595052	-1.359747
87	6	0	5.408974	1.111846	4.170169
88	6	0	6.604078	-0.594247	2.789832
89	6	0	6.186604	1.725689	1.862629
90	1	0	6.342359	1.371119	0.843220
91	1	0	7.136925	2.096109	2.258993
92	1	0	5.476385	2.558224	1.842460
93	1	0	6.344391	1.460868	4.615767
94	1	0	4.999944	0.312514	4.794908
95	1	0	4.696722	1.941511	4.163376
96	1	0	7.508446	-0.324061	3.343058
97	1	0	6.889557	-0.913173	1.787805
98	1	0	6.127081	-1.431212	3.309578
99	1	0	-0.492107	-4.741738	-0.544771
100	6	0	-0.757327	-3.612694	-2.369862
101	6	0	-1.259417	-4.711873	-3.063804
102	6	0	-0.592516	-2.393209	-3.033480
103	6	0	-1.603833	-4.598613	-4.410397
104	1	0	-1.386079	-5.661926	-2.549114
105	6	0	-0.928163	-2.285037	-4.379555
106	1	0	-0.200528	-1.522788	-2.507878
107	6	0	-1.439011	-3.384114	-5.070032
108	1	0	-1.993581	-5.460755	-4.943183
109	1	0	-0.788742	-1.334426	-4.885894
110	1	0	-1.702127	-3.295277	-6.119607
111	6	0	2.755704	-0.918953	-2.068276
112	1	0	2.317188	-1.632803	-2.751198

113	1	0	3.801529	-0.903989	-1.801686
114	7	0	2.108239	0.246557	-1.935052
115	8	0	2.652617	1.167662	-1.242791
116	8	0	0.963567	0.426555	-2.434689
117	6	0	0.377283	3.748970	-2.260885
118	6	0	0.035874	5.219277	-1.999391
119	6	0	1.625991	3.640234	-3.139888
120	1	0	-0.446327	3.271291	-2.811857
121	6	0	-0.185750	5.957445	-3.321087
122	1	0	0.879390	5.669211	-1.458227
123	1	0	-0.843241	5.312403	-1.353802
124	6	0	1.420945	4.384976	-4.459523
125	1	0	2.478106	4.068126	-2.593960
126	1	0	1.841721	2.582621	-3.313407
127	6	0	1.042695	5.848523	-4.225414
128	1	0	-0.428071	7.006511	-3.124102
129	1	0	-1.055078	5.522773	-3.834863
130	1	0	2.328563	4.315516	-5.067021
131	1	0	0.621247	3.891383	-5.028190
132	1	0	0.860039	6.353518	-5.179732
133	1	0	1.884728	6.365683	-3.746052

G2-1a-I-*si*-TS2

Zero-point correction= 1.14905 (a.u.)

Thermal correction to Gibbs Free Energy= 1.08068 (a.u.)

Sum of electronic and zero-point Energies= -2892.38705 (a.u.)

Sum of electronic and thermal Free Energies= -2892.45542 (a.u.)

Standard orientation:

Center	Atomic	Atomic	Coordinates (Angstroms)		
Number	Number	Type	X	Y	Z
1	6	0	-0.068713	2.737253	4.543231
2	6	0	0.234594	3.707463	3.396725
3	6	0	0.515951	2.981789	2.076814
4	7	0	-0.584952	2.048589	1.790291
5	6	0	-1.150571	1.720151	4.160400
6	6	0	-0.731070	1.048365	2.856277
7	1	0	-0.363951	3.293442	5.437951
8	1	0	0.847376	2.187801	4.797829
9	1	0	1.099688	4.330175	3.647484
10	1	0	-0.616588	4.384944	3.254487
11	1	0	0.617822	3.687436	1.254379

12	1	0	1.436150	2.390827	2.120754
13	1	0	-1.270428	0.961348	4.938453
14	1	0	-2.124515	2.209775	4.038718
15	1	0	0.285746	0.659426	2.991417
16	6	0	-1.523384	2.312079	0.852072
17	7	0	-1.129265	2.954312	-0.241839
18	7	0	-2.790694	1.916032	1.060624
19	6	0	-3.777549	1.556110	0.037067
20	1	0	-0.127440	2.795714	-0.497595
21	6	0	-1.593933	-0.170364	2.467037
22	8	0	-2.686370	-0.353665	2.996157
23	7	0	-0.980245	-0.952419	1.563352
24	1	0	-0.123235	-0.579965	1.145994
25	6	0	-3.236042	0.598667	-1.028669
26	6	0	-4.340729	0.361441	-2.062342
27	6	0	-5.573877	-0.262331	-1.406506
28	6	0	-4.978492	0.927774	0.747822
29	1	0	-2.960623	-0.346978	-0.538698
30	1	0	-2.321018	0.984448	-1.491087
31	1	0	-4.616727	1.322572	-2.520006
32	1	0	-3.978224	-0.272281	-2.875994
33	1	0	-4.124371	2.474972	-0.457815
34	1	0	-6.366286	-0.416592	-2.146146
35	1	0	-5.349800	1.613291	1.518974
36	1	0	-4.641088	0.014390	1.257389
37	6	0	-6.087928	0.606286	-0.255247
38	1	0	-5.305045	-1.253894	-1.015280
39	1	0	-6.916841	0.110131	0.258773
40	1	0	-6.483989	1.545585	-0.664229
41	1	0	-3.009600	1.578731	1.992086
42	6	0	-2.472485	-2.946231	1.817049
43	6	0	-1.348594	-2.270847	1.052149
44	6	0	-0.093188	-3.138931	1.008065
45	6	0	-2.280307	-3.435391	3.110125
46	6	0	-3.318961	-4.067058	3.782258
47	6	0	-4.559686	-4.229065	3.164224
48	6	0	-4.753211	-3.757545	1.870699
49	6	0	-3.709139	-3.121065	1.200929
50	1	0	-1.311006	-3.316409	3.588095
51	1	0	-3.162871	-4.439724	4.790002
52	1	0	-5.368544	-4.726930	3.689894
53	1	0	-5.713057	-3.885844	1.379431
54	1	0	-3.852609	-2.753015	0.187304
55	6	0	1.009222	-2.894042	1.826090

56	6	0	2.179827	-3.637008	1.677323
57	6	0	-0.022634	-4.174252	0.073527
58	6	0	1.140658	-4.921006	-0.072397
59	6	0	2.254268	-4.644022	0.720304
60	1	0	3.040283	-3.414510	2.302414
61	1	0	0.974033	-2.084839	2.549621
62	1	0	3.173692	-5.205664	0.587689
63	1	0	1.188667	-5.707916	-0.819362
64	1	0	-0.879342	-4.365086	-0.568300
65	1	0	-1.669064	-2.149158	0.010002
66	6	0	2.592052	0.926568	-2.062988
67	1	0	2.531867	0.335362	-2.964115
68	1	0	3.516079	1.332871	-1.677485
69	7	0	1.465111	1.579497	-1.736805
70	8	0	1.492444	2.490567	-0.834249
71	8	0	0.378601	1.242627	-2.256681
72	8	0	-0.904506	-2.163545	-2.180055
73	8	0	1.689948	0.222243	1.179912
74	7	0	3.766041	-0.596987	0.618129
75	7	0	0.779379	-1.209861	-1.089739
76	8	0	0.972165	-1.701952	-3.357247
77	1	0	5.220782	-0.763521	2.096253
78	6	0	-1.606205	-2.723550	-3.319216
79	6	0	2.050163	-1.054351	-0.886790
80	6	0	2.431567	-0.383742	0.420949
81	6	0	3.308360	-1.697758	-1.363622
82	6	0	4.304174	-1.370387	-0.420303
83	6	0	3.641472	-2.507317	-2.442844
84	1	0	2.892382	-2.761823	-3.180104
85	6	0	0.352523	-1.704431	-2.308997
86	6	0	4.955943	-2.964177	-2.569333
87	1	0	5.221247	-3.592603	-3.412823
88	6	0	4.499872	-0.026283	1.729510
89	1	0	3.761198	0.145413	2.518618
90	6	0	5.615351	-1.797794	-0.545879
91	1	0	6.373046	-1.508040	0.174586
92	6	0	5.929026	-2.606968	-1.640719
93	1	0	6.948896	-2.956917	-1.765588
94	6	0	5.189948	1.269761	1.359471
95	6	0	6.545003	1.461528	1.618326
96	6	0	4.453194	2.290152	0.748323
97	6	0	7.165748	2.661511	1.273296
98	1	0	7.119881	0.668371	2.090999
99	6	0	5.076073	3.483023	0.396728

100	1	0	3.393771	2.153873	0.531215
101	6	0	6.432817	3.672252	0.659341
102	1	0	8.223082	2.800655	1.476381
103	1	0	4.496830	4.263608	-0.087433
104	1	0	6.917258	4.603623	0.383048
105	6	0	-0.883278	-3.961622	-3.842189
106	1	0	-0.708945	-4.672173	-3.027766
107	1	0	-1.504951	-4.453573	-4.596585
108	1	0	0.073739	-3.698109	-4.292413
109	6	0	-1.780954	-1.662789	-4.402737
110	1	0	-2.141103	-0.729963	-3.959133
111	1	0	-0.837515	-1.457660	-4.908243
112	1	0	-2.514462	-2.005960	-5.138794
113	6	0	-2.950920	-3.115995	-2.716331
114	1	0	-3.618318	-3.508157	-3.488658
115	1	0	-2.819893	-3.887248	-1.950035
116	1	0	-3.426674	-2.246345	-2.252201
117	6	0	-1.925124	3.746751	-1.173897
118	6	0	-2.691592	4.869459	-0.469172
119	6	0	-0.962478	4.317028	-2.218036
120	1	0	-2.643300	3.107919	-1.708501
121	6	0	-3.449038	5.722917	-1.489581
122	1	0	-1.963674	5.490311	0.071242
123	1	0	-3.378540	4.454708	0.277866
124	6	0	-1.714269	5.172655	-3.235888
125	1	0	-0.207567	4.925027	-1.700121
126	1	0	-0.436519	3.487579	-2.700771
127	6	0	-2.500604	6.290287	-2.548123
128	1	0	-3.981404	6.530395	-0.976816
129	1	0	-4.212524	5.103815	-1.981896
130	1	0	-1.009252	5.589096	-3.961932
131	1	0	-2.408616	4.535430	-3.800386
132	1	0	-3.061205	6.873958	-3.285773
133	1	0	-1.795793	6.979838	-2.063979

G2-1a-I-*re*-IM3

Zero-point correction= 1.15238 (a.u.)

Thermal correction to Gibbs Free Energy= 1.08320 (a.u.)

Sum of electronic and zero-point Energies= -2892.40906 (a.u.)

Sum of electronic and thermal Free Energies= -2892.47825 (a.u.)

Standard orientation:

Center Atomic Atomic Coordinates (Angstroms)

Number	Number	Type	X	Y	Z
1	6	0	2.049745	1.421795	3.729176
2	6	0	2.674117	2.492591	2.842372
3	6	0	2.281092	2.260441	1.390694
4	7	0	0.808200	2.324363	1.249891
5	6	0	0.535316	1.439224	3.546400
6	6	0	0.147764	1.271501	2.070381
7	1	0	2.302879	1.585776	4.781851
8	1	0	2.451419	0.446681	3.428147
9	1	0	3.765741	2.465996	2.901201
10	1	0	2.346131	3.492198	3.155383
11	1	0	2.719770	3.027686	0.751001
12	1	0	2.628756	1.273307	1.060527
13	1	0	0.064136	0.625448	4.108560
14	1	0	0.102520	2.380369	3.904143
15	1	0	0.546087	0.319703	1.694360
16	6	0	0.266131	2.910475	0.157301
17	7	0	1.017215	3.062853	-0.934030
18	7	0	-0.991022	3.379837	0.210492
19	6	0	-1.969434	3.350587	-0.884454
20	1	0	1.790925	2.406070	-1.033402
21	6	0	-1.381811	1.245053	2.034976
22	8	0	-2.065928	2.170978	2.474223
23	7	0	-1.918845	0.083758	1.614883
24	1	0	-1.335357	-0.558219	1.081066
25	6	0	-2.043853	1.967469	-1.536600
26	6	0	-3.126130	1.927249	-2.616271
27	6	0	-4.484195	2.365943	-2.062611
28	6	0	-3.324358	3.769679	-0.313889
29	1	0	-2.267239	1.234017	-0.748867
30	1	0	-1.067509	1.686694	-1.949326
31	1	0	-2.841788	2.595449	-3.441143
32	1	0	-3.185952	0.918197	-3.039610
33	1	0	-1.693017	4.093939	-1.644613
34	1	0	-5.235617	2.371511	-2.858902
35	1	0	-3.240596	4.769629	0.126786
36	1	0	-3.591732	3.080604	0.499592
37	6	0	-4.394351	3.744628	-1.405791
38	1	0	-4.826264	1.640414	-1.310903
39	1	0	-5.361680	4.031242	-0.982162
40	1	0	-4.148254	4.494746	-2.169606
41	1	0	-1.389578	3.386210	1.147033
42	6	0	-3.926453	-0.101566	3.049325

43	6	0	-3.367555	-0.098146	1.634725
44	6	0	-3.730191	-1.359434	0.860650
45	6	0	-3.170468	-0.526855	4.140968
46	6	0	-3.731990	-0.556356	5.415619
47	6	0	-5.053220	-0.163356	5.608436
48	6	0	-5.813650	0.257619	4.519382
49	6	0	-5.251300	0.286976	3.247733
50	1	0	-2.138117	-0.831153	3.990475
51	1	0	-3.132521	-0.882821	6.259818
52	1	0	-5.488302	-0.182207	6.602676
53	1	0	-6.844063	0.568423	4.661788
54	1	0	-5.843181	0.616321	2.396772
55	6	0	-3.810404	-2.604529	1.487518
56	6	0	-4.147152	-3.740907	0.754738
57	6	0	-3.974141	-1.275371	-0.513198
58	6	0	-4.314039	-2.408540	-1.246196
59	6	0	-4.406167	-3.644739	-0.610344
60	1	0	-4.215156	-4.701620	1.255983
61	1	0	-3.629856	-2.682591	2.555357
62	1	0	-4.675072	-4.528519	-1.180657
63	1	0	-4.494270	-2.329651	-2.314255
64	1	0	-3.898175	-0.312639	-1.011474
65	1	0	-3.816710	0.757686	1.110760
66	8	0	4.333477	-0.417233	2.136604
67	8	0	0.023946	-0.907819	-0.242926
68	7	0	0.328992	-3.134792	-0.694818
69	7	0	2.712580	-1.172241	0.816346
70	8	0	4.936043	-1.362418	0.154048
71	6	0	5.705377	-0.241007	2.531761
72	6	0	2.299646	-1.870890	-0.353841
73	6	0	0.757864	-1.876558	-0.401153
74	6	0	2.604388	-3.364445	-0.495554
75	6	0	1.404175	-4.040429	-0.746088
76	6	0	3.794277	-4.073942	-0.473141
77	1	0	4.724192	-3.549774	-0.286561
78	6	0	4.025522	-1.034520	0.939660
79	6	0	3.757985	-5.457051	-0.690621
80	1	0	4.682084	-6.025377	-0.666501
81	6	0	-1.062134	-3.467098	-0.932790
82	1	0	-1.658929	-2.724852	-0.393266
83	6	0	1.348759	-5.403592	-0.985472
84	1	0	0.410732	-5.905509	-1.199417
85	6	0	2.555919	-6.107795	-0.949986
86	1	0	2.549454	-7.178153	-1.130917

87	6	0	5.595995	0.279796	3.963768
88	6	0	6.474604	-1.564311	2.547080
89	6	0	6.381667	0.795079	1.636733
90	1	0	6.418696	0.435116	0.608058
91	1	0	7.399823	0.995678	1.986839
92	1	0	5.819397	1.735275	1.661220
93	1	0	6.590339	0.473007	4.377351
94	1	0	5.091968	-0.456687	4.596855
95	1	0	5.021878	1.209479	3.999433
96	1	0	7.421403	-1.429976	3.080821
97	1	0	6.678116	-1.919218	1.538365
98	1	0	5.890924	-2.324554	3.076573
99	1	0	-1.273079	-4.447784	-0.496877
100	6	0	-1.413523	-3.436927	-2.404123
101	6	0	-1.998322	-4.536316	-3.028372
102	6	0	-1.185247	-2.270195	-3.141038
103	6	0	-2.363644	-4.473836	-4.372919
104	1	0	-2.176814	-5.445294	-2.458169
105	6	0	-1.546100	-2.208846	-4.482766
106	1	0	-0.729422	-1.407646	-2.658214
107	6	0	-2.139929	-3.309569	-5.101010
108	1	0	-2.818761	-5.336312	-4.850047
109	1	0	-1.363799	-1.298822	-5.046560
110	1	0	-2.422325	-3.259784	-6.148038
111	6	0	2.764572	-1.273354	-1.740927
112	1	0	2.317686	-1.811165	-2.576866
113	1	0	3.853237	-1.277461	-1.737815
114	7	0	2.342756	0.134298	-1.882468
115	8	0	3.019252	0.982229	-1.311130
116	8	0	1.348244	0.392805	-2.540134
117	6	0	0.847479	3.996583	-2.049645
118	6	0	0.693876	5.445236	-1.581045
119	6	0	2.070542	3.850116	-2.957706
120	1	0	-0.033179	3.708903	-2.641730
121	6	0	0.577537	6.383674	-2.784858
122	1	0	1.582714	5.704355	-0.990188
123	1	0	-0.172335	5.552106	-0.920127
124	6	0	1.965447	4.789086	-4.159615
125	1	0	2.970501	4.087797	-2.373760
126	1	0	2.154562	2.806986	-3.280176
127	6	0	1.782683	6.241532	-3.715840
128	1	0	0.478236	7.416976	-2.438486
129	1	0	-0.340833	6.146929	-3.340432
130	1	0	2.856911	4.687352	-4.785800

131	1	0	1.108390	4.487375	-4.776594
132	1	0	1.668169	6.893522	-4.587866
133	1	0	2.685977	6.572655	-3.186192

G2-1a-I-*si*-IM3

Zero-point correction= 1.15275 (a.u.)

Thermal correction to Gibbs Free Energy= 1.08450 (a.u.)

Sum of electronic and zero-point Energies= -2892.40585 (a.u.)

Sum of electronic and thermal Free Energies= -2892.47410 (a.u.)

Standard orientation:

Center Number	Atomic Number	Atomic Type	Coordinates (Angstroms) X	Y	Z
1	6	0	-0.268496	3.096402	4.141009
2	6	0	-0.446844	4.020381	2.934500
3	6	0	-0.114254	3.315088	1.617350
4	7	0	-0.893172	2.066278	1.512848
5	6	0	-1.020839	1.775999	3.952635
6	6	0	-0.588162	1.150335	2.628058
7	1	0	-0.601261	3.599971	5.053513
8	1	0	0.799398	2.876230	4.268862
9	1	0	0.193852	4.903213	3.029448
10	1	0	-1.483049	4.379683	2.895722
11	1	0	-0.338463	3.957216	0.767808
12	1	0	0.941018	3.036200	1.557534
13	1	0	-0.805614	1.078447	4.766144
14	1	0	-2.105109	1.942530	3.955851
15	1	0	0.504157	1.082176	2.617898
16	6	0	-2.016965	1.996443	0.772001
17	7	0	-2.058502	2.689019	-0.374311
18	7	0	-3.049202	1.255571	1.192796
19	6	0	-4.080696	0.596088	0.379383
20	1	0	-1.151547	2.871654	-0.798829
21	6	0	-1.109289	-0.291506	2.438114
22	8	0	-2.025261	-0.699758	3.151506
23	7	0	-0.441064	-0.975049	1.499655
24	1	0	0.171797	-0.457443	0.860356
25	6	0	-3.528435	-0.183793	-0.817182
26	6	0	-4.710837	-0.778278	-1.589386
27	6	0	-5.534105	-1.715810	-0.704272
28	6	0	-4.858144	-0.341175	1.307257
29	1	0	-2.862422	-0.978527	-0.450798

30	1	0	-2.915778	0.450978	-1.465514
31	1	0	-5.352653	0.041086	-1.945443
32	1	0	-4.357671	-1.303771	-2.480770
33	1	0	-4.781231	1.359055	0.010431
34	1	0	-6.386668	-2.117589	-1.261365
35	1	0	-5.232962	0.224598	2.168700
36	1	0	-4.164640	-1.100268	1.694654
37	6	0	-6.017259	-1.000291	0.558980
38	1	0	-4.910764	-2.573092	-0.414993
39	1	0	-6.532243	-1.700461	1.224066
40	1	0	-6.748613	-0.229651	0.279177
41	1	0	-2.988728	0.904284	2.144302
42	6	0	-1.432348	-3.219849	2.018044
43	6	0	-0.570701	-2.376483	1.096059
44	6	0	0.821630	-2.975354	0.923570
45	6	0	-0.988135	-3.610855	3.282130
46	6	0	-1.796942	-4.389041	4.100780
47	6	0	-3.056048	-4.799042	3.660487
48	6	0	-3.497438	-4.429690	2.394662
49	6	0	-2.682943	-3.646724	1.577715
50	1	0	-0.004378	-3.297671	3.622622
51	1	0	-1.445627	-4.683445	5.085075
52	1	0	-3.684212	-5.410593	4.300856
53	1	0	-4.470853	-4.753424	2.037671
54	1	0	-3.019362	-3.358627	0.584070
55	6	0	1.948106	-2.461739	1.564235
56	6	0	3.208056	-3.007370	1.320589
57	6	0	0.972337	-4.060970	0.059629
58	6	0	2.227430	-4.604343	-0.186377
59	6	0	3.354917	-4.072226	0.437836
60	1	0	4.081730	-2.586656	1.811980
61	1	0	1.845676	-1.605461	2.225996
62	1	0	4.340208	-4.476981	0.228402
63	1	0	2.330302	-5.431710	-0.882098
64	1	0	0.094688	-4.458709	-0.444242
65	1	0	-1.023091	-2.381592	0.097491
66	6	0	2.019944	1.120256	-2.330831
67	1	0	1.886771	0.728002	-3.336601
68	1	0	2.870672	1.794457	-2.227926
69	7	0	0.802534	1.914252	-2.033409
70	8	0	0.890414	2.852624	-1.245034
71	8	0	-0.237919	1.583035	-2.565871
72	8	0	-0.704068	-2.123364	-2.058277
73	8	0	1.756461	0.995853	0.824187

74	7	0	3.823629	0.194944	0.258814
75	7	0	0.888323	-0.829644	-1.202664
76	8	0	0.869207	-1.298012	-3.480157
77	1	0	5.169533	-0.174365	1.801774
78	6	0	-1.319498	-2.895037	-3.101605
79	6	0	2.111203	-0.112872	-1.354701
80	6	0	2.500301	0.432464	0.035714
81	6	0	3.403376	-0.839693	-1.748301
82	6	0	4.377752	-0.578738	-0.775529
83	6	0	3.738006	-1.620688	-2.843411
84	1	0	2.989055	-1.825455	-3.598025
85	6	0	0.431080	-1.377749	-2.318207
86	6	0	5.036586	-2.137749	-2.936676
87	1	0	5.302342	-2.758093	-3.786431
88	6	0	4.512686	0.629765	1.454896
89	1	0	3.737762	0.785910	2.211982
90	6	0	5.672354	-1.063918	-0.856948
91	1	0	6.414096	-0.829987	-0.099912
92	6	0	5.988983	-1.859281	-1.962463
93	1	0	6.993714	-2.259229	-2.057807
94	6	0	5.295957	1.906889	1.234187
95	6	0	6.622083	2.018304	1.646131
96	6	0	4.673927	2.999673	0.622572
97	6	0	7.323765	3.207045	1.451005
98	1	0	7.110607	1.170871	2.121339
99	6	0	5.374226	4.183872	0.424712
100	1	0	3.636739	2.917483	0.302373
101	6	0	6.701702	4.290538	0.839286
102	1	0	8.358276	3.281825	1.771590
103	1	0	4.884442	5.026522	-0.053409
104	1	0	7.247754	5.215312	0.681611
105	6	0	-0.348038	-3.909647	-3.705781
106	1	0	0.173439	-4.442810	-2.903953
107	1	0	-0.902586	-4.642010	-4.302210
108	1	0	0.393144	-3.419817	-4.334794
109	6	0	-1.912781	-1.976453	-4.168340
110	1	0	-2.534709	-1.211089	-3.692779
111	1	0	-1.118092	-1.476719	-4.721304
112	1	0	-2.535859	-2.550446	-4.862480
113	6	0	-2.428745	-3.640558	-2.361626
114	1	0	-3.047250	-4.212900	-3.059537
115	1	0	-2.003525	-4.335018	-1.629056
116	1	0	-3.069174	-2.931734	-1.827608
117	6	0	-3.234138	3.216787	-1.067056

118	6	0	-4.131007	4.037096	-0.136963
119	6	0	-2.746091	4.079305	-2.231708
120	1	0	-3.821687	2.392394	-1.494910
121	6	0	-5.333859	4.580772	-0.909915
122	1	0	-3.534958	4.869287	0.262837
123	1	0	-4.458585	3.435239	0.717791
124	6	0	-3.934720	4.643536	-3.012253
125	1	0	-2.138938	4.900988	-1.825880
126	1	0	-2.097260	3.480330	-2.879259
127	6	0	-4.883835	5.427374	-2.102440
128	1	0	-5.972211	5.166774	-0.241727
129	1	0	-5.941315	3.738217	-1.269050
130	1	0	-3.573028	5.279185	-3.826132
131	1	0	-4.480841	3.813098	-3.479083
132	1	0	-5.752486	5.773159	-2.671741
133	1	0	-4.369111	6.323429	-1.730542

G3

Zero-point correction= 0.81586 (a.u.)

Thermal correction to Gibbs Free Energy= 0.76399 (a.u.)

Sum of electronic and zero-point Energies= -1845.87547 (a.u.)

Sum of electronic and thermal Free Energies= -1845.92734 (a.u.)

Standard orientation:

Center Number	Atomic Number	Atomic Type	Coordinates (Angstroms) X	Y	Z
1	7	0	-0.419199	-2.875437	-1.394842
2	6	0	-1.538151	-2.167227	-2.040616
3	1	0	-1.092028	-1.483903	-2.770522
4	6	0	0.682025	-2.129183	-1.172080
5	7	0	1.065968	0.304419	-1.893456
6	7	0	3.108451	-2.386316	-0.203273
7	6	0	4.081080	-1.432681	0.326841
8	6	0	3.721785	-0.946634	1.733595
9	6	0	4.752748	0.057051	2.252673
10	6	0	6.160516	-0.541685	2.223615
11	6	0	5.482829	-2.038343	0.300022
12	1	0	3.683181	-1.819312	2.403001
13	1	0	2.718891	-0.503152	1.719196
14	1	0	4.725743	0.955804	1.620191
15	1	0	4.489475	0.376541	3.265910
16	1	0	4.061883	-0.569886	-0.353870

17	1	0	6.894570	0.196135	2.563271
18	1	0	5.721136	-2.356159	-0.719765
19	1	0	5.491155	-2.938970	0.931709
20	6	0	6.518353	-1.038428	0.821273
21	1	0	6.207159	-1.384344	2.927168
22	1	0	7.513137	-1.495047	0.817843
23	1	0	6.560673	-0.180831	0.135841
24	1	0	3.038324	-3.268125	0.290746
25	6	0	2.200863	1.223369	-1.846736
26	6	0	2.028470	2.274813	-2.942591
27	6	0	2.326850	1.896652	-0.475951
28	1	0	3.145443	0.681660	-2.045581
29	6	0	3.145459	3.318830	-2.918420
30	1	0	1.057537	2.762519	-2.781984
31	1	0	1.975978	1.773140	-3.915112
32	6	0	3.441075	2.945057	-0.444217
33	1	0	1.364894	2.369213	-0.242890
34	1	0	2.498915	1.128753	0.290426
35	6	0	3.240166	3.987778	-1.545773
36	1	0	2.980192	4.069364	-3.698919
37	1	0	4.104129	2.832083	-3.147670
38	1	0	3.473922	3.428068	0.539338
39	1	0	4.414973	2.453396	-0.589988
40	1	0	4.052193	4.723209	-1.530558
41	1	0	2.306351	4.532041	-1.348447
42	6	0	1.268141	-0.876323	-1.418822
43	6	0	1.963081	-1.928826	-0.763603
44	6	0	-2.282586	-1.341221	-0.981811
45	6	0	-0.744689	-4.006839	-0.524349
46	6	0	-1.551843	-5.034600	-1.308254
47	6	0	-2.805299	-4.388463	-1.899196
48	6	0	-2.441200	-3.172871	-2.758149
49	1	0	0.199868	-4.429087	-0.169464
50	1	0	-0.921699	-5.435939	-2.111679
51	1	0	-1.820520	-5.865268	-0.648128
52	1	0	-3.346741	-5.114128	-2.514548
53	1	0	-3.469335	-4.072725	-1.091502
54	1	0	-1.903740	-3.510637	-3.651865
55	1	0	-3.345750	-2.656000	-3.093034
56	1	0	-1.314037	-3.659489	0.350496
57	8	0	-3.214376	-1.809433	-0.341198
58	7	0	-1.793493	-0.085614	-0.786589
59	1	0	-0.930710	0.192839	-1.268949
60	6	0	-2.133028	0.674068	0.428049

61	6	0	-1.712052	-0.198827	1.627009
62	6	0	-0.346437	-0.457090	1.804331
63	6	0	-2.626506	-0.851215	2.452247
64	6	0	0.090894	-1.320169	2.802987
65	1	0	0.374139	0.027844	1.146053
66	6	0	-2.188687	-1.713909	3.456514
67	1	0	-3.689137	-0.707961	2.295996
68	6	0	-0.831093	-1.948742	3.638687
69	1	0	1.153058	-1.505380	2.929557
70	1	0	-2.918633	-2.212378	4.086858
71	1	0	-0.490393	-2.621598	4.419644
72	6	0	-3.617180	1.083105	0.451014
73	6	0	-4.131048	1.717201	1.586755
74	6	0	-4.440010	0.944385	-0.664665
75	6	0	-5.444999	2.170079	1.618048
76	1	0	-3.494175	1.859814	2.456181
77	6	0	-5.754385	1.406270	-0.638663
78	1	0	-4.053125	0.474703	-1.561722
79	6	0	-6.265434	2.013640	0.503004
80	1	0	-5.825683	2.651578	2.513571
81	1	0	-6.380232	1.284514	-1.517625
82	1	0	-7.291167	2.368024	0.523668
83	6	0	-1.367395	2.010818	0.375723
84	6	0	-1.323181	2.725249	-0.826603
85	6	0	-0.829128	2.597824	1.522712
86	6	0	-0.754367	3.993628	-0.878526
87	1	0	-1.750496	2.291158	-1.726312
88	6	0	-0.255496	3.867311	1.469897
89	1	0	-0.855282	2.064005	2.467100
90	6	0	-0.217318	4.571340	0.270861
91	1	0	-0.732544	4.533321	-1.820689
92	1	0	0.158987	4.303760	2.373391
93	1	0	0.225503	5.561765	0.229362

G3-COM

Zero-point correction= 0.86889 (a.u.)

Thermal correction to Gibbs Free Energy= 0.81051 (a.u.)

Sum of electronic and zero-point Energies= -2090.74019 (a.u.)

Sum of electronic and thermal Free Energies= -2090.79857 (a.u.)

Standard orientation:

Center Number	Atomic Number	Atomic Type	Coordinates (Angstroms) X	Y	Z

1	7	0	0.241754	-2.004234	-2.611846
2	6	0	-0.940934	-1.163316	-2.417025
3	1	0	-0.670813	-0.152150	-2.735585
4	6	0	1.358019	-1.450823	-2.070939
5	7	0	2.126831	1.022037	-1.842425
6	7	0	3.479900	-2.338073	-0.823304
7	6	0	4.157523	-1.802120	0.363041
8	6	0	3.257003	-1.839990	1.601427
9	6	0	3.982494	-1.286077	2.828633
10	6	0	5.293916	-2.035568	3.074272
11	6	0	5.459307	-2.558971	0.605434
12	1	0	2.969862	-2.886098	1.787147
13	1	0	2.330961	-1.285733	1.405832
14	1	0	4.195211	-0.218782	2.668245
15	1	0	3.332779	-1.348721	3.708060
16	1	0	4.396963	-0.754328	0.135561
17	1	0	5.822658	-1.607417	3.932276
18	1	0	6.087642	-2.499915	-0.288691
19	1	0	5.223808	-3.621254	0.767767
20	6	0	6.188125	-2.005530	1.832752
21	1	0	5.066595	-3.079998	3.328634
22	1	0	7.108780	-2.570897	2.009648
23	1	0	6.487299	-0.967561	1.631083
24	1	0	3.172861	-3.298535	-0.696303
25	6	0	3.286061	1.600258	-1.166519
26	6	0	3.682785	2.922380	-1.824575
27	6	0	2.986151	1.831323	0.316815
28	1	0	4.157251	0.921487	-1.237542
29	6	0	4.873370	3.569187	-1.112626
30	1	0	2.818188	3.599524	-1.776005
31	1	0	3.902632	2.751817	-2.884968
32	6	0	4.162286	2.479271	1.047627
33	1	0	2.096635	2.473606	0.376327
34	1	0	2.705952	0.880652	0.789120
35	6	0	4.574701	3.789598	0.372496
36	1	0	5.133164	4.518020	-1.594166
37	1	0	5.751294	2.915698	-1.210302
38	1	0	3.909183	2.651196	2.100657
39	1	0	5.019071	1.788638	1.039158
40	1	0	5.444613	4.225116	0.876138
41	1	0	3.754113	4.513993	0.468136
42	6	0	2.057966	-0.259714	-1.781994
43	6	0	2.527846	-1.554540	-1.403293

44	1	0	1.112101	2.485632	-3.142218
45	6	0	-1.349974	-1.124563	-0.932208
46	6	0	0.090208	-3.461108	-2.483281
47	6	0	-1.063726	-3.943540	-3.355395
48	6	0	-2.343915	-3.166598	-3.042606
49	6	0	-2.105980	-1.673698	-3.272304
50	1	0	1.031680	-3.909693	-2.816670
51	1	0	-0.802593	-3.801838	-4.411428
52	1	0	-1.208240	-5.015506	-3.190276
53	1	0	-3.166803	-3.515153	-3.674161
54	1	0	-2.630994	-3.342008	-1.998001
55	1	0	-1.854121	-1.499964	-4.324678
56	1	0	-2.998384	-1.080411	-3.045571
57	1	0	-0.086123	-3.735699	-1.437252
58	6	0	0.281088	3.193371	-3.271263
59	1	0	-0.130509	3.179541	-4.277020
60	1	0	0.560210	4.190450	-2.937791
61	7	0	-0.754698	2.683993	-2.346317
62	8	0	-0.662925	2.957689	-1.169416
63	8	0	-1.624945	1.957645	-2.815653
64	8	0	-1.140375	-2.068742	-0.183298
65	7	0	-2.033697	-0.010932	-0.564019
66	1	0	-1.972171	0.805804	-1.164986
67	6	0	-2.540336	0.196977	0.797404
68	6	0	-1.333151	0.292956	1.754012
69	6	0	-0.595653	1.480926	1.803673
70	6	0	-0.896000	-0.796865	2.510771
71	6	0	0.511469	1.596932	2.639079
72	1	0	-0.895395	2.324280	1.188829
73	6	0	0.216803	-0.683354	3.341924
74	1	0	-1.420215	-1.742708	2.439287
75	6	0	0.917029	0.517736	3.420605
76	1	0	1.059558	2.534155	2.675737
77	1	0	0.534730	-1.541644	3.927470
78	1	0	1.776422	0.610693	4.078690
79	6	0	-3.544134	-0.908848	1.183198
80	6	0	-3.947574	-1.047139	2.514449
81	6	0	-4.165016	-1.700101	0.216873
82	6	0	-4.920067	-1.974347	2.874176
83	1	0	-3.494385	-0.425007	3.280960
84	6	0	-5.139731	-2.628202	0.573905
85	1	0	-3.889236	-1.591100	-0.827213
86	6	0	-5.518829	-2.774415	1.904489
87	1	0	-5.211665	-2.067055	3.916141

88	1	0	-5.602265	-3.238783	-0.195887
89	1	0	-6.277067	-3.499433	2.183769
90	6	0	-3.383883	1.489057	0.789238
91	6	0	-4.109083	1.853402	-0.348766
92	6	0	-3.540941	2.250713	1.950821
93	6	0	-4.945629	2.966696	-0.333852
94	1	0	-4.037826	1.260980	-1.255967
95	6	0	-4.380201	3.360376	1.966814
96	1	0	-2.996080	1.982772	2.850312
97	6	0	-5.083090	3.727113	0.822391
98	1	0	-5.494869	3.232643	-1.231774
99	1	0	-4.480846	3.939735	2.879650
100	1	0	-5.734107	4.595620	0.834097

G3-TS1

Zero-point correction= 0.86528 (a.u.)

Thermal correction to Gibbs Free Energy= 0.80781 (a.u.)

Sum of electronic and zero-point Energies= -2090.72762 (a.u.)

Sum of electronic and thermal Free Energies= -2090.78510 (a.u.)

Standard orientation:

Center Number	Atomic Number	Atomic Type	Coordinates (Angstroms) X	Y	Z
1	7	0	0.259151	-2.152657	-2.324726
2	6	0	-0.942256	-1.312629	-2.293424
3	1	0	-0.661037	-0.323197	-2.663985
4	6	0	1.356577	-1.560439	-1.824924
5	7	0	2.172536	0.915514	-1.722367
6	7	0	3.544587	-2.350253	-0.612428
7	6	0	4.309971	-1.728844	0.477279
8	6	0	3.501104	-1.679787	1.776623
9	6	0	4.310279	-1.041163	2.905877
10	6	0	5.641088	-1.770199	3.106961
11	6	0	5.630590	-2.466987	0.674822
12	1	0	3.233370	-2.710123	2.053519
13	1	0	2.560176	-1.140535	1.611729
14	1	0	4.504371	0.012844	2.659047
15	1	0	3.726435	-1.046126	3.831751
16	1	0	4.527935	-0.701020	0.156921
17	1	0	6.228387	-1.281004	3.890671
18	1	0	6.193011	-2.468563	-0.264047
19	1	0	5.413748	-3.515528	0.926415

20	6	0	6.442796	-1.828567	1.804755
21	1	0	5.439811	-2.793622	3.451639
22	1	0	7.375500	-2.382118	1.952566
23	1	0	6.724528	-0.808521	1.509318
24	1	0	3.281863	-3.313158	-0.425043
25	6	0	3.374181	1.565583	-1.200287
26	6	0	3.690866	2.824604	-2.007993
27	6	0	3.218953	1.914600	0.280732
28	1	0	4.227122	0.874302	-1.309518
29	6	0	4.955740	3.506095	-1.480701
30	1	0	2.845883	3.519300	-1.918837
31	1	0	3.791292	2.567937	-3.068556
32	6	0	4.470924	2.604785	0.825913
33	1	0	2.345310	2.574267	0.374979
34	1	0	2.988180	1.005970	0.851830
35	6	0	4.816539	3.848859	0.004530
36	1	0	5.164514	4.409577	-2.062561
37	1	0	5.814730	2.834917	-1.619455
38	1	0	4.329180	2.866361	1.880447
39	1	0	5.316713	1.902204	0.785698
40	1	0	5.737972	4.307604	0.378033
41	1	0	4.016821	4.591374	0.127127
42	6	0	2.068010	-0.374355	-1.601978
43	6	0	2.558227	-1.625773	-1.189471
44	1	0	1.297739	1.602373	-2.384361
45	6	0	-1.462043	-1.190742	-0.848217
46	6	0	0.119367	-3.605275	-2.133946
47	6	0	-0.956006	-4.149867	-3.067248
48	6	0	-2.264940	-3.376334	-2.899993
49	6	0	-2.028783	-1.896325	-3.202298
50	1	0	1.090254	-4.054470	-2.366636
51	1	0	-0.612135	-4.063261	-4.105319
52	1	0	-1.100245	-5.212959	-2.852917
53	1	0	-3.031228	-3.776264	-3.570540
54	1	0	-2.629537	-3.494425	-1.871826
55	1	0	-1.703286	-1.779714	-4.242375
56	1	0	-2.941405	-1.303929	-3.077735
57	1	0	-0.137583	-3.820131	-1.091977
58	6	0	0.418623	2.446605	-3.102471
59	1	0	0.289679	2.144284	-4.137453
60	1	0	0.846733	3.436283	-2.963462
61	7	0	-0.812136	2.368328	-2.417307
62	8	0	-0.946495	2.940025	-1.336858
63	8	0	-1.710774	1.631912	-2.876437

64	8	0	-1.296153	-2.091037	-0.034112
65	7	0	-2.157858	-0.060512	-0.587431
66	1	0	-2.132715	0.693529	-1.275475
67	6	0	-2.698546	0.234324	0.744332
68	6	0	-1.518624	0.253154	1.738239
69	6	0	-0.577455	1.282776	1.619261
70	6	0	-1.308122	-0.738942	2.695376
71	6	0	0.508401	1.353052	2.486258
72	1	0	-0.706369	2.035525	0.844651
73	6	0	-0.212180	-0.676659	3.554911
74	1	0	-1.991885	-1.577087	2.762590
75	6	0	0.690912	0.377634	3.465744
76	1	0	1.211093	2.176185	2.402351
77	1	0	-0.069225	-1.457290	4.295924
78	1	0	1.532886	0.440604	4.148955
79	6	0	-3.812043	-0.758805	1.129114
80	6	0	-4.383084	-0.681763	2.404141
81	6	0	-4.347232	-1.667247	0.217486
82	6	0	-5.438354	-1.509955	2.767406
83	1	0	-3.996527	0.038113	3.120454
84	6	0	-5.408658	-2.495934	0.577311
85	1	0	-3.941821	-1.727341	-0.786835
86	6	0	-5.955621	-2.426134	1.853384
87	1	0	-5.862446	-1.432501	3.763803
88	1	0	-5.806805	-3.198423	-0.148707
89	1	0	-6.782361	-3.071446	2.133009
90	6	0	-3.398674	1.606781	0.662837
91	6	0	-4.137278	1.940069	-0.476653
92	6	0	-3.401224	2.491873	1.742556
93	6	0	-4.831447	3.142714	-0.547322
94	1	0	-4.174816	1.257440	-1.320464
95	6	0	-4.103751	3.692449	1.674991
96	1	0	-2.841514	2.253577	2.641071
97	6	0	-4.816515	4.026131	0.528231
98	1	0	-5.387957	3.386602	-1.446735
99	1	0	-4.086007	4.369279	2.523600
100	1	0	-5.356796	4.965989	0.472930

G3-IM1

Zero-point correction= 0.86824 (a.u.)

Thermal correction to Gibbs Free Energy= 0.81074 (a.u.)

Sum of electronic and zero-point Energies= -2090.74442 (a.u.)

Sum of electronic and thermal Free Energies= -2090.80193 (a.u.)

Standard orientation:

Center Number	Atomic Number	Atomic Type	Coordinates (Angstroms) X	Y	Z
1	7	0	-0.202130	-2.473845	1.924278
2	6	0	1.046030	-1.697362	2.052036
3	1	0	0.796379	-0.743632	2.526305
4	6	0	-1.250915	-1.776905	1.502897
5	7	0	-1.653933	0.787526	1.350437
6	7	0	-3.667433	-2.223747	0.567075
7	6	0	-4.576937	-1.447059	-0.281104
8	6	0	-4.085500	-1.380003	-1.729286
9	6	0	-5.042757	-0.556517	-2.592750
10	6	0	-6.463641	-1.121532	-2.522876
11	6	0	-5.987880	-2.024028	-0.204874
12	1	0	-4.025206	-2.405948	-2.119832
13	1	0	-3.069051	-0.969337	-1.759015
14	1	0	-5.048464	0.483654	-2.235419
15	1	0	-4.688029	-0.532132	-3.628040
16	1	0	-4.592729	-0.431493	0.139494
17	1	0	-7.147824	-0.504170	-3.113638
18	1	0	-6.320792	-2.043734	0.837417
19	1	0	-5.962523	-3.064521	-0.560269
20	6	0	-6.950520	-1.210184	-1.074571
21	1	0	-6.473358	-2.124888	-2.970116
22	1	0	-7.951254	-1.651073	-1.031983
23	1	0	-7.033495	-0.196082	-0.660515
24	1	0	-3.620187	-3.216911	0.369789
25	6	0	-2.786519	1.695786	1.174711
26	6	0	-2.544828	2.968984	1.984493
27	6	0	-3.027544	2.030736	-0.297012
28	1	0	-3.688367	1.201491	1.571174
29	6	0	-3.729436	3.926150	1.837917
30	1	0	-1.625091	3.444424	1.615852
31	1	0	-2.365252	2.709897	3.032316
32	6	0	-4.226314	2.968685	-0.447265
33	1	0	-2.122245	2.511757	-0.692445
34	1	0	-3.173912	1.105830	-0.867971
35	6	0	-4.019506	4.247154	0.368847
36	1	0	-3.534309	4.846386	2.397507
37	1	0	-4.619724	3.465603	2.288219
38	1	0	-4.388231	3.208226	-1.503635
39	1	0	-5.134448	2.455718	-0.096625

40	1	0	-4.897856	4.896122	0.289894
41	1	0	-3.171304	4.803740	-0.051931
42	6	0	-1.800214	-0.519574	1.290371
43	6	0	-2.522434	-1.665555	0.991896
44	1	0	-0.813330	1.124190	1.878015
45	6	0	1.600090	-1.452605	0.640622
46	6	0	-0.140685	-3.916155	1.643434
47	6	0	0.842168	-4.592971	2.590910
48	6	0	2.206224	-3.902894	2.542835
49	6	0	2.049729	-2.435562	2.939391
50	1	0	-1.150052	-4.317706	1.778213
51	1	0	0.446645	-4.548826	3.613311
52	1	0	0.923753	-5.647766	2.311195
53	1	0	2.903484	-4.399006	3.224791
54	1	0	2.620313	-3.974626	1.531134
55	1	0	1.691197	-2.371565	3.973417
56	1	0	2.999741	-1.893724	2.898913
57	1	0	0.172201	-4.065159	0.604960
58	6	0	1.658590	2.967178	3.750847
59	1	0	2.699632	3.235614	3.833432
60	1	0	0.841845	3.599848	4.059336
61	7	0	1.369474	1.791938	3.269826
62	8	0	0.137250	1.397784	3.179973
63	8	0	2.293465	0.973144	2.893598
64	8	0	1.650597	-2.359292	-0.186216
65	7	0	1.994657	-0.183158	0.404967
66	1	0	2.004149	0.452289	1.218481
67	6	0	2.495386	0.233514	-0.907559
68	6	0	1.364061	-0.033157	-1.925874
69	6	0	0.222864	0.776368	-1.880014
70	6	0	1.391634	-1.091353	-2.833736
71	6	0	-0.833681	0.571114	-2.762450
72	1	0	0.170901	1.582406	-1.151689
73	6	0	0.331418	-1.304870	-3.712608
74	1	0	2.237977	-1.767686	-2.842717
75	6	0	-0.779364	-0.468631	-3.690091
76	1	0	-1.698753	1.227613	-2.731397
77	1	0	0.377607	-2.133370	-4.413013
78	1	0	-1.599470	-0.626615	-4.384615
79	6	0	3.818932	-0.479991	-1.258084
80	6	0	4.362153	-0.357494	-2.541767
81	6	0	4.551845	-1.166096	-0.291543
82	6	0	5.585331	-0.937977	-2.857963
83	1	0	3.820697	0.193886	-3.305506

84	6	0	5.781455	-1.741883	-0.603370
85	1	0	4.165130	-1.246098	0.718580
86	6	0	6.300787	-1.637985	-1.888992
87	1	0	5.982561	-0.838023	-3.863446
88	1	0	6.331239	-2.274400	0.166537
89	1	0	7.256007	-2.091827	-2.133907
90	6	0	2.851601	1.735573	-0.855573
91	6	0	3.403886	2.299362	0.298550
92	6	0	2.740776	2.533798	-1.999162
93	6	0	3.803505	3.633995	0.311963
94	1	0	3.514672	1.716212	1.207959
95	6	0	3.151275	3.863417	-1.985885
96	1	0	2.322449	2.117695	-2.909961
97	6	0	3.681090	4.422818	-0.826845
98	1	0	4.219291	4.052728	1.223301
99	1	0	3.053014	4.461620	-2.886905
100	1	0	3.996497	5.461532	-0.812682

G3-I-*re*-TS2

Zero-point correction= 1.23959 (a.u.)

Thermal correction to Gibbs Free Energy= 1.16442 (a.u.)

Sum of electronic and zero-point Energies= -3199.37659 (a.u.)

Sum of electronic and thermal Free Energies= -3199.45176 (a.u.)

Standard orientation:

Center Number	Atomic Number	Atomic Type	Coordinates (Angstroms) X	Y	Z
1	7	0	1.743378	-2.764364	-0.605004
2	6	0	0.415739	-2.142174	-0.681734
3	1	0	0.539608	-1.053002	-0.651072
4	6	0	2.840815	-2.024465	-0.606414
5	7	0	3.461351	0.519628	-0.570188
6	7	0	5.409778	-2.583773	-0.765276
7	6	0	6.624093	-1.889469	-0.325962
8	6	0	6.773604	-1.882946	1.197642
9	6	0	8.044478	-1.140643	1.616286
10	6	0	9.279437	-1.746957	0.946349
11	6	0	7.847997	-2.508963	-0.995760
12	1	0	6.820292	-2.924472	1.546624
13	1	0	5.884986	-1.430597	1.653650
14	1	0	7.956699	-0.083935	1.324840
15	1	0	8.147990	-1.159074	2.705494

16	1	0	6.527368	-0.850988	-0.671378
17	1	0	10.176265	-1.183998	1.223720
18	1	0	7.720232	-2.487575	-2.082585
19	1	0	7.917061	-3.565018	-0.695633
20	6	0	9.123300	-1.773711	-0.575720
21	1	0	9.420662	-2.772472	1.313357
22	1	0	9.994486	-2.242628	-1.043460
23	1	0	9.078874	-0.742195	-0.951428
24	1	0	5.400445	-3.584843	-0.608323
25	6	0	4.652991	1.316676	-0.854453
26	6	0	4.249268	2.561428	-1.647390
27	6	0	5.415399	1.697490	0.416349
28	1	0	5.316021	0.710664	-1.490577
29	6	0	5.475944	3.409968	-1.984033
30	1	0	3.537121	3.141622	-1.047778
31	1	0	3.717782	2.253672	-2.554549
32	6	0	6.644882	2.543625	0.076460
33	1	0	4.736950	2.261553	1.069776
34	1	0	5.702308	0.788360	0.960168
35	6	0	6.250376	3.790064	-0.720094
36	1	0	5.168938	4.307807	-2.529598
37	1	0	6.137004	2.843035	-2.654168
38	1	0	7.176700	2.821703	0.992257
39	1	0	7.342334	1.940677	-0.524362
40	1	0	7.140032	4.373990	-0.977845
41	1	0	5.617881	4.430195	-0.090469
42	6	0	3.492959	-0.791874	-0.589572
43	6	0	4.215032	-1.970942	-0.645354
44	1	0	2.534689	1.019866	-0.547873
45	6	0	-0.456211	-2.601718	0.496458
46	6	0	1.856494	-4.205697	-0.878846
47	6	0	1.216172	-4.539144	-2.223881
48	6	0	-0.222840	-4.022530	-2.261957
49	6	0	-0.261204	-2.513717	-2.010892
50	1	0	2.919470	-4.461202	-0.869982
51	1	0	1.796334	-4.069744	-3.028571
52	1	0	1.249155	-5.622520	-2.376898
53	1	0	-0.682956	-4.251983	-3.229125
54	1	0	-0.809677	-4.544310	-1.493477
55	1	0	0.259610	-1.977151	-2.813311
56	1	0	-1.289904	-2.146575	-1.986293
57	1	0	1.357746	-4.741711	-0.069914
58	6	0	-0.212783	2.693688	-2.102526
59	1	0	-0.169061	3.688776	-1.685501

60	1	0	-0.668899	2.452477	-3.050468
61	7	0	0.646971	1.799387	-1.615425
62	8	0	0.758941	0.657503	-2.127347
63	8	0	1.326364	2.087909	-0.562285
64	8	0	-3.183676	-1.145924	-2.756166
65	8	0	-0.892525	1.122812	0.899400
66	7	0	-2.096980	3.081094	0.644038
67	7	0	-2.095028	0.368973	-1.554671
68	8	0	-2.473672	0.729379	-3.817201
69	6	0	-3.712713	-1.781774	-3.954406
70	6	0	-2.168956	1.585674	-1.134520
71	6	0	-1.606381	1.859581	0.251396
72	6	0	-3.018886	2.757801	-1.452286
73	6	0	-2.939736	3.614358	-0.333721
74	6	0	-3.832048	3.102871	-2.525067
75	1	0	-3.885042	2.464231	-3.397817
76	6	0	-2.593032	0.056783	-2.811749
77	6	0	-4.557378	4.294850	-2.466494
78	1	0	-5.191880	4.574878	-3.300370
79	6	0	-1.848310	3.662684	1.945672
80	1	0	-1.443486	2.850961	2.559226
81	6	0	-3.643222	4.807220	-0.271919
82	1	0	-3.551631	5.468600	0.583240
83	6	0	-4.458747	5.133452	-1.358304
84	1	0	-5.019622	6.062530	-1.336966
85	6	0	-4.201965	-3.125692	-3.427934
86	6	0	-4.871116	-0.958506	-4.506922
87	6	0	-2.605529	-1.985557	-4.983712
88	1	0	-2.250301	-1.036554	-5.384606
89	1	0	-2.988684	-2.596071	-5.806956
90	1	0	-1.762062	-2.515553	-4.529575
91	1	0	-4.648080	-3.707729	-4.238983
92	1	0	-4.950532	-2.985569	-2.642944
93	1	0	-3.365010	-3.694494	-3.009278
94	1	0	-5.364824	-1.512880	-5.310506
95	1	0	-4.520967	-0.005162	-4.905202
96	1	0	-5.607370	-0.769328	-3.719645
97	1	0	-2.805508	3.968144	2.383880
98	6	0	-0.886194	4.830069	1.894547
99	6	0	-1.190152	6.031117	2.532095
100	6	0	0.330403	4.694235	1.219391
101	6	0	-0.288934	7.094183	2.501058
102	1	0	-2.137922	6.136817	3.055651
103	6	0	1.226147	5.758023	1.186164

104	1	0	0.574840	3.756148	0.720592
105	6	0	0.920843	6.958712	1.827117
106	1	0	-0.535005	8.026553	2.999960
107	1	0	2.170235	5.646429	0.660422
108	1	0	1.623470	7.785933	1.798866
109	8	0	-0.242640	-3.634741	1.117240
110	7	0	-1.516543	-1.790818	0.720581
111	1	0	-1.544826	-0.901933	0.221319
112	6	0	-2.414726	-1.954420	1.872107
113	6	0	-1.547977	-2.070915	3.143061
114	6	0	-0.602585	-1.061666	3.369594
115	6	0	-1.622086	-3.129682	4.044766
116	6	0	0.228485	-1.101539	4.482394
117	1	0	-0.524233	-0.242340	2.659116
118	6	0	-0.787637	-3.169178	5.162487
119	1	0	-2.311326	-3.948094	3.872651
120	6	0	0.136105	-2.156544	5.389298
121	1	0	0.953660	-0.308459	4.638144
122	1	0	-0.857717	-4.006965	5.849658
123	1	0	0.785253	-2.190803	6.258906
124	6	0	-3.373232	-3.139816	1.674268
125	6	0	-4.380788	-3.356775	2.621902
126	6	0	-3.337810	-3.951736	0.543087
127	6	0	-5.307281	-4.378991	2.459221
128	1	0	-4.441938	-2.706382	3.491165
129	6	0	-4.272218	-4.973974	0.373184
130	1	0	-2.591962	-3.771842	-0.223361
131	6	0	-5.254682	-5.196705	1.331155
132	1	0	-6.078632	-4.529119	3.208189
133	1	0	-4.229656	-5.593479	-0.517914
134	1	0	-5.982137	-5.991127	1.197497
135	6	0	-3.305323	-0.691178	1.899112
136	6	0	-4.035261	-0.384993	0.744166
137	6	0	-3.438784	0.134502	3.014096
138	6	0	-4.838051	0.748715	0.688657
139	1	0	-3.952012	-1.029408	-0.128188
140	6	0	-4.258578	1.263785	2.964291
141	1	0	-2.900556	-0.091234	3.927663
142	6	0	-4.949784	1.583321	1.800661
143	1	0	-5.377529	0.980894	-0.225296
144	1	0	-4.347757	1.894536	3.844144
145	1	0	-5.573525	2.471391	1.757338

G3-I-*si*-TS2

Zero-point correction= 1.23960 (a.u.)

Thermal correction to Gibbs Free Energy= 1.16447 (a.u.)

Sum of electronic and zero-point Energies= -3199.37644 (a.u.)

Sum of electronic and thermal Free Energies= -3199.45157 (a.u.)

Standard orientation:

Center Number	Atomic Number	Atomic Type	Coordinates (Angstroms) X	Y	Z
1	7	0	-1.913522	0.050592	-2.939645
2	6	0	-0.547414	0.384942	-2.536875
3	1	0	-0.263676	-0.289061	-1.717525
4	6	0	-2.782246	-0.286320	-1.991063
5	7	0	-2.683356	-0.961179	0.540105
6	7	0	-5.375728	-0.692945	-1.926988
7	6	0	-6.367914	-0.906634	-0.871815
8	6	0	-6.628384	0.357258	-0.048596
9	6	0	-7.659162	0.086110	1.048500
10	6	0	-8.958681	-0.466613	0.458438
11	6	0	-7.660427	-1.445353	-1.479179
12	1	0	-6.998929	1.139864	-0.725266
13	1	0	-5.687040	0.723768	0.378737
14	1	0	-7.245256	-0.643617	1.759159
15	1	0	-7.852141	1.001902	1.615360
16	1	0	-5.953065	-1.682614	-0.213599
17	1	0	-9.672317	-0.692892	1.256952
18	1	0	-7.445933	-2.354368	-2.050260
19	1	0	-8.057764	-0.699698	-2.183336
20	6	0	-8.694827	-1.716417	-0.383998
21	1	0	-9.421178	0.303094	-0.174069
22	1	0	-9.624022	-2.080811	-0.832574
23	1	0	-8.321968	-2.519161	0.267083
24	1	0	-5.692917	-0.245978	-2.778160
25	6	0	-3.605029	-1.583782	1.491273
26	6	0	-3.754666	-3.081850	1.209526
27	6	0	-3.128368	-1.337123	2.921680
28	1	0	-4.582101	-1.095035	1.366938
29	6	0	-4.697207	-3.742497	2.216107
30	1	0	-2.757217	-3.538216	1.269988
31	1	0	-4.111035	-3.228513	0.181908
32	6	0	-4.064738	-2.006102	3.930605
33	1	0	-2.111340	-1.739664	3.025049
34	1	0	-3.073531	-0.258861	3.102343

35	6	0	-4.219204	-3.501858	3.649262
36	1	0	-4.775678	-4.814511	2.009510
37	1	0	-5.707387	-3.323123	2.096847
38	1	0	-3.686871	-1.844009	4.944915
39	1	0	-5.052100	-1.525129	3.880402
40	1	0	-4.915403	-3.952647	4.364029
41	1	0	-3.248869	-3.996046	3.790395
42	6	0	-3.060757	-0.693284	-0.693206
43	6	0	-4.078569	-0.557747	-1.622342
44	1	0	-1.675169	-1.050747	0.733146
45	6	0	-0.512939	1.819745	-1.974745
46	7	0	0.723780	2.211223	-1.563855
47	1	0	1.397386	1.452620	-1.479143
48	6	0	0.927431	3.275360	-0.556986
49	6	0	-1.486017	0.406365	-5.332403
50	6	0	-2.437742	0.658619	-4.170452
51	6	0	0.409235	0.159430	-3.713579
52	6	0	-0.074864	0.871479	-4.975041
53	1	0	-2.565233	1.735270	-4.003724
54	1	0	-3.419293	0.216153	-4.365371
55	1	0	-1.470382	-0.665870	-5.561181
56	1	0	-1.862250	0.930382	-6.216339
57	1	0	1.408727	0.491589	-3.427603
58	1	0	0.469756	-0.920229	-3.894824
59	1	0	-0.075721	1.957796	-4.808998
60	1	0	0.612605	0.675345	-5.802979
61	8	0	-1.519433	2.507233	-1.893646
62	6	0	0.808349	4.674662	-1.171733
63	6	0	0.332361	4.892938	-2.462711
64	6	0	1.235734	5.776504	-0.420194
65	6	0	0.276572	6.183292	-2.989284
66	1	0	-0.001450	4.053806	-3.060323
67	6	0	1.170543	7.063817	-0.939342
68	1	0	1.632874	5.616212	0.579198
69	6	0	0.690160	7.272553	-2.231249
70	1	0	-0.095059	6.332042	-3.998716
71	1	0	1.505245	7.903844	-0.338460
72	1	0	0.645017	8.275998	-2.643086
73	6	0	-0.059561	3.036602	0.601272
74	6	0	-0.892866	4.018138	1.134201
75	6	0	-0.113695	1.742254	1.131896
76	6	0	-1.750269	3.717883	2.194016
77	1	0	-0.893532	5.019966	0.719557
78	6	0	-0.959882	1.447541	2.191609

79	1	0	0.540346	0.958132	0.752284
80	6	0	-1.784762	2.434995	2.730480
81	1	0	-2.396479	4.494090	2.593122
82	1	0	-0.953375	0.437203	2.587615
83	1	0	-2.446525	2.206436	3.562281
84	6	0	2.393421	3.092193	-0.101904
85	6	0	3.389338	3.164733	-1.086181
86	6	0	2.767312	2.839090	1.214536
87	6	0	4.724970	2.973500	-0.761857
88	1	0	3.104234	3.373443	-2.114764
89	6	0	4.111768	2.650307	1.540230
90	1	0	2.020202	2.739062	1.992771
91	6	0	5.092282	2.714773	0.559813
92	1	0	5.481877	3.029405	-1.538580
93	1	0	4.377393	2.426407	2.569188
94	1	0	6.135749	2.558070	0.815846
95	6	0	1.560100	-2.885656	0.816700
96	1	0	1.724151	-3.097173	1.863988
97	1	0	1.883135	-3.555432	0.030657
98	7	0	0.412789	-2.206060	0.561179
99	8	0	-0.078442	-2.159297	-0.595211
100	8	0	-0.104288	-1.543437	1.499311
101	8	0	1.952123	0.738666	3.593631
102	8	0	2.152598	-0.286361	-1.185193
103	7	0	3.912988	-1.760134	-1.096102
104	7	0	2.383425	-0.271126	1.669930
105	8	0	2.328727	-1.497886	3.643531
106	6	0	1.678691	0.836101	5.012186
107	6	0	2.959470	-1.199516	0.955201
108	6	0	2.922445	-0.989616	-0.550836
109	6	0	4.121748	-2.115151	1.175229
110	6	0	4.632773	-2.430919	-0.098242
111	6	0	4.722229	-2.657587	2.303455
112	1	0	4.326475	-2.431697	3.285358
113	6	0	2.250148	-0.445403	3.022722
114	6	0	5.817903	-3.510485	2.142443
115	1	0	6.291801	-3.940517	3.018503
116	6	0	4.122061	-1.887627	-2.524008
117	1	0	3.695744	-0.984606	-2.971604
118	6	0	5.702871	-3.293178	-0.273500
119	1	0	6.062465	-3.551075	-1.264116
120	6	0	6.293316	-3.829392	0.873348
121	1	0	7.132919	-4.509206	0.767471
122	6	0	1.426392	2.327478	5.205370

123	6	0	0.426850	0.038336	5.370510
124	6	0	2.892726	0.392276	5.825587
125	1	0	3.045998	-0.684545	5.753616
126	1	0	2.747059	0.662046	6.876187
127	1	0	3.790923	0.903608	5.464183
128	1	0	1.161894	2.535071	6.246429
129	1	0	0.605565	2.657663	4.560999
130	1	0	2.320670	2.905679	4.953041
131	1	0	0.226288	0.135741	6.442250
132	1	0	0.553922	-1.017912	5.129543
133	1	0	-0.436742	0.430458	4.824712
134	1	0	5.197571	-1.889700	-2.726484
135	6	0	3.461808	-3.123755	-3.096563
136	6	0	4.177160	-4.015854	-3.891796
137	6	0	2.110317	-3.369289	-2.830561
138	6	0	3.554495	-5.145146	-4.422228
139	1	0	5.228421	-3.827609	-4.097821
140	6	0	1.492083	-4.499534	-3.355511
141	1	0	1.538049	-2.683902	-2.206062
142	6	0	2.211346	-5.389139	-4.153599
143	1	0	4.122649	-5.835381	-5.038248
144	1	0	0.444674	-4.684288	-3.136299
145	1	0	1.726943	-6.270915	-4.561498

G4

Zero-point correction= 0.88157 (a.u.)
Thermal correction to Gibbs Free Energy= 0.82042 (a.u.)
Sum of electronic and zero-point Energies= -2839.71652 (a.u.)
Sum of electronic and thermal Free Energies= -2839.77767 (a.u.)

Standard orientation:

Center Number	Atomic Number	Atomic Type	Coordinates (Angstroms) X	Y	Z
1	6	0	-2.791957	0.490487	1.033697
2	7	0	-1.717645	2.802930	1.334302
3	7	0	-4.657098	1.323883	-0.566196
4	6	0	-5.423482	0.106665	-0.833036
5	6	0	-6.100476	-0.467435	0.414807
6	6	0	-6.859357	-1.750401	0.069876
7	6	0	-7.883454	-1.499627	-1.039215
8	6	0	-6.441332	0.372956	-1.938656
9	1	0	-6.796298	0.284677	0.811957

10	1	0	-5.351232	-0.656712	1.193170
11	1	0	-6.142857	-2.514085	-0.265337
12	1	0	-7.348112	-2.150328	0.963446
13	1	0	-4.700609	-0.633767	-1.207905
14	1	0	-8.400351	-2.429039	-1.298691
15	1	0	-5.929087	0.767424	-2.823473
16	1	0	-7.141253	1.148090	-1.593187
17	6	0	-7.217250	-0.901556	-2.280307
18	1	0	-8.647483	-0.802527	-0.669650
19	1	0	-7.963348	-0.687764	-3.051736
20	1	0	-6.521970	-1.637198	-2.706969
21	1	0	-5.143198	2.207549	-0.646449
22	6	0	-1.966632	4.148475	0.832388
23	6	0	-1.918739	5.149581	1.989795
24	6	0	-0.914851	4.512906	-0.222356
25	1	0	-2.962548	4.218024	0.356402
26	6	0	-2.070593	6.593092	1.506673
27	1	0	-0.952652	5.024361	2.497496
28	1	0	-2.695443	4.894180	2.719071
29	6	0	-1.061141	5.954807	-0.711713
30	1	0	0.076432	4.372234	0.233179
31	1	0	-0.987142	3.809211	-1.061903
32	6	0	-1.010264	6.938415	0.458891
33	1	0	-2.010240	7.283566	2.354642
34	1	0	-3.067662	6.724719	1.063518
35	1	0	-0.279408	6.186332	-1.442862
36	1	0	-2.022726	6.064461	-1.232035
37	1	0	-1.145363	7.964752	0.101012
38	1	0	-0.016583	6.889810	0.924624
39	6	0	-2.537106	1.872543	0.994662
40	6	0	-3.640616	1.272911	0.322686
41	1	0	-0.245996	1.462225	1.273359
42	1	0	-1.836072	-1.705221	-0.305035
43	1	0	-0.056597	-2.012179	3.582017
44	1	0	-1.199531	-6.564784	2.830086
45	1	0	-0.585558	-4.451765	3.958961
46	1	0	-3.082397	0.729489	-2.530999
47	1	0	-0.989087	1.470959	-1.495289
48	1	0	1.881267	0.714800	-2.115378
49	1	0	6.180242	2.423355	1.675976
50	1	0	5.313758	3.374488	-0.450933
51	1	0	3.055485	2.765321	-1.246764
52	1	0	6.022750	-2.308491	2.259330
53	1	0	8.037115	-0.900729	1.739851

54	1	0	8.071820	0.484100	-0.343198
55	16	0	3.693916	-1.234117	-1.882180
56	9	0	6.193663	0.578351	-2.057475
57	9	0	3.949639	-2.460814	0.737153
58	8	0	0.721900	-1.561804	1.026391
59	8	0	3.781081	-0.224476	-2.924440
60	8	0	3.776473	-2.647566	-2.215608
61	7	0	-2.366362	-0.718263	1.490383
62	7	0	2.322231	-0.967762	-1.055679
63	7	0	0.320359	0.689200	0.903832
64	6	0	-2.485529	-1.853408	0.570744
65	6	0	-1.065650	-1.913201	3.171063
66	6	0	-1.129451	-0.674342	2.285123
67	6	0	0.087432	-0.558697	1.350886
68	6	0	1.266514	1.096803	-0.125282
69	6	0	1.405532	0.121017	-1.327716
70	6	0	-2.136383	-3.157537	1.239631
71	6	0	-2.469412	-4.351932	0.597449
72	6	0	-2.138952	-5.577436	1.159727
73	6	0	-1.464791	-5.613311	2.379787
74	6	0	-1.123314	-4.426103	3.014207
75	6	0	-1.445711	-3.187987	2.452258
76	6	0	2.638327	1.458229	0.412876
77	6	0	3.435604	2.340894	-0.320096
78	6	0	4.705800	2.686439	0.128164
79	6	0	5.191365	2.151137	1.319948
80	6	0	4.406474	1.260186	2.047123
81	6	0	3.136821	0.909376	1.592603
82	6	0	0.070656	-0.376519	-1.876490
83	6	0	-0.046938	-1.663474	-2.407218
84	6	0	-1.243622	-2.088899	-2.980757
85	6	0	-2.339875	-1.232070	-3.037907
86	6	0	-2.234433	0.050834	-2.504448
87	6	0	-1.042283	0.470577	-1.918988
88	6	0	5.026796	-0.972812	-0.713692
89	6	0	6.140760	-0.176132	-0.961698
90	6	0	7.225901	-0.145935	-0.094040
91	6	0	7.192350	-0.917740	1.059802
92	6	0	6.083142	-1.706368	1.360712
93	6	0	5.029986	-1.714317	0.467346
94	1	0	4.783904	0.834120	2.971811
95	1	0	-3.270007	-1.559292	-3.494484
96	1	0	-1.312449	-3.092608	-3.388680
97	1	0	0.806356	-2.333206	-2.374476

98	1	0	2.081376	-1.595198	-0.286729
99	1	0	0.834642	2.016899	-0.537565
100	1	0	-2.402985	-6.499036	0.650926
101	1	0	-2.991273	-4.310175	-0.356457
102	1	0	-1.748592	-1.758250	4.013916
103	1	0	-3.520434	-1.882681	0.214724
104	1	0	2.530426	0.206614	2.156394
105	1	0	-1.186610	0.225626	2.905001

G4-COM

Zero-point correction= 0.93558 (a.u.)
Thermal correction to Gibbs Free Energy= 0.87014 (a.u.)
Sum of electronic and zero-point Energies= -3084.58706 (a.u.)
Sum of electronic and thermal Free Energies= -3084.65250 (a.u.)

Standard orientation:

Center Number	Atomic Number	Atomic Type	Coordinates (Angstroms) X	Y	Z
1	6	0	-2.964938	-1.046622	-1.183397
2	7	0	-0.965795	-2.557700	-1.856420
3	7	0	-4.188731	-2.990772	0.042669
4	6	0	-5.092986	-2.309525	0.972462
5	6	0	-6.140975	-3.299221	1.474714
6	6	0	-7.128623	-2.619167	2.425319
7	6	0	-6.404349	-1.938998	3.589648
8	6	0	-4.358084	-1.650012	2.144586
9	1	0	-5.624515	-4.112149	2.007038
10	1	0	-6.660059	-3.748602	0.621895
11	1	0	-7.700052	-1.865639	1.866571
12	1	0	-7.851740	-3.351623	2.797884
13	1	0	-5.602237	-1.527037	0.391242
14	1	0	-7.124949	-1.426037	4.235094
15	1	0	-3.609347	-0.939989	1.773098
16	1	0	-3.814560	-2.432684	2.692885
17	6	0	-5.347881	-0.955803	3.082038
18	1	0	-5.913897	-2.705329	4.205374
19	1	0	-4.812986	-0.496322	3.918714
20	1	0	-5.844859	-0.138325	2.540065
21	1	0	-3.833545	-3.885573	0.358549
22	6	0	-0.660905	-3.947375	-1.519408
23	6	0	0.037800	-4.632188	-2.695800
24	6	0	0.238693	-4.011445	-0.280537

25	1	0	-1.588250	-4.510749	-1.297270
26	6	0	0.435573	-6.072024	-2.364075
27	1	0	0.938364	-4.046106	-2.926988
28	1	0	-0.611637	-4.596999	-3.578423
29	6	0	0.639665	-5.445452	0.068240
30	1	0	1.133946	-3.415678	-0.504923
31	1	0	-0.264207	-3.525524	0.565009
32	6	0	1.325306	-6.124003	-1.119625
33	1	0	0.945111	-6.531220	-3.218154
34	1	0	-0.470852	-6.666163	-2.181753
35	1	0	1.294494	-5.454905	0.947218
36	1	0	-0.257234	-6.019380	0.340626
37	1	0	1.581228	-7.160693	-0.875299
38	1	0	2.268891	-5.602151	-1.332053
39	6	0	-2.077981	-2.120521	-1.384623
40	6	0	-3.301840	-2.252675	-0.663665
41	1	0	0.562588	0.767212	-0.715506
42	1	0	-4.293864	0.694473	0.379481
43	1	0	-1.189875	3.008035	-1.862271
44	1	0	-4.601027	6.252631	-1.290975
45	1	0	-2.593451	5.039445	-2.093827
46	1	0	-1.666072	5.608366	0.486486
47	1	0	-0.773014	3.488405	1.294613
48	1	0	2.349915	2.723767	1.916990
49	1	0	3.349313	-2.795598	3.589109
50	1	0	3.222989	-0.596881	4.744898
51	1	0	2.035064	1.285851	3.685602
52	1	0	4.478834	-2.421017	-1.840999
53	1	0	5.604430	-3.643178	0.041577
54	1	0	6.158784	-2.421644	2.153077
55	16	0	4.411430	1.874019	0.437393
56	9	0	5.674019	0.038276	2.580018
57	9	0	3.903165	0.093861	-1.774365
58	8	0	-2.017516	1.418961	1.001109
59	8	0	4.581931	2.352301	1.797081
60	8	0	5.165039	2.462154	-0.659617
61	7	0	-3.143285	0.290231	-1.352890
62	7	0	2.813037	1.964993	0.061140
63	7	0	0.044304	1.065357	0.105903
64	6	0	-4.298363	0.902912	-0.698573
65	6	0	-2.083391	2.393200	-2.022359
66	6	0	-1.879874	1.023682	-1.375605
67	6	0	-1.302349	1.171018	0.035916
68	6	0	0.787668	1.380135	1.305528

69	6	0	1.814072	2.493524	0.990925
70	6	0	-4.350638	2.398424	-0.897718
71	6	0	-5.475283	3.088212	-0.437274
72	6	0	-5.573895	4.465991	-0.574093
73	6	0	-4.535381	5.174923	-1.177780
74	6	0	-3.412258	4.494502	-1.629764
75	6	0	-3.304819	3.106571	-1.494231
76	6	0	1.488431	0.179005	1.919462
77	6	0	2.093102	0.329515	3.170943
78	6	0	2.760203	-0.732023	3.772423
79	6	0	2.830869	-1.962402	3.124019
80	6	0	2.241564	-2.116209	1.872193
81	6	0	1.574079	-1.051584	1.267696
82	6	0	1.204413	3.784542	0.453113
83	6	0	1.994128	4.660467	-0.298706
84	6	0	1.477145	5.866223	-0.763647
85	6	0	0.159552	6.217958	-0.484360
86	6	0	-0.634011	5.354114	0.265597
87	6	0	-0.118803	4.145407	0.729610
88	6	0	4.761297	0.125993	0.401852
89	6	0	5.368791	-0.576079	1.439812
90	6	0	5.682760	-1.923447	1.316719
91	6	0	5.368150	-2.589017	0.139368
92	6	0	4.748037	-1.924574	-0.917112
93	6	0	4.468428	-0.580698	-0.765075
94	1	0	2.307559	-3.069666	1.356374
95	1	0	-0.244880	7.157590	-0.848065
96	1	0	2.109190	6.529573	-1.345823
97	1	0	3.024113	4.399351	-0.526554
98	1	0	2.647699	2.061358	-0.936365
99	1	0	0.060953	1.756272	2.029774
100	1	0	-6.454709	4.986352	-0.211363
101	1	0	-6.279573	2.530799	0.037988
102	1	0	-2.186104	2.243099	-3.103230
103	1	0	-5.197808	0.448632	-1.132132
104	1	0	1.111219	-1.190905	0.293238
105	1	0	-1.183678	0.436530	-1.982650
106	6	0	0.886099	-1.393021	-4.150693
107	1	0	0.792868	-0.515623	-4.785218
108	1	0	1.584463	-2.128005	-4.543609
109	7	0	1.386973	-0.948646	-2.831136
110	8	0	1.983175	-1.744403	-2.142918
111	8	0	1.129547	0.203532	-2.499859
112	1	0	-0.082705	-1.847876	-3.921798

G4-TS1

Zero-point correction= 0.92989 (a.u.)

Thermal correction to Gibbs Free Energy= 0.86404 (a.u.)

Sum of electronic and zero-point Energies= -3084.57574 (a.u.)

Sum of electronic and thermal Free Energies= -3084.64159 (a.u.)

Standard orientation:

Center Number	Atomic Number	Atomic Type	Coordinates (Angstroms) X	Y	Z
1	6	0	-2.917815	-1.124920	-1.253053
2	7	0	-0.908285	-2.529432	-2.113840
3	7	0	-3.950546	-3.190141	-0.052200
4	6	0	-4.836838	-2.605884	0.960770
5	6	0	-5.793143	-3.674221	1.482119
6	6	0	-6.755038	-3.082485	2.515426
7	6	0	-5.999112	-2.405138	3.661195
8	6	0	-4.062691	-1.953283	2.110196
9	1	0	-5.201348	-4.474082	1.950993
10	1	0	-6.340175	-4.119975	0.644892
11	1	0	-7.398454	-2.342840	2.020423
12	1	0	-7.414438	-3.866851	2.899682
13	1	0	-5.424496	-1.832367	0.446614
14	1	0	-6.706351	-1.955815	4.365596
15	1	0	-3.377438	-1.188095	1.724507
16	1	0	-3.445457	-2.725013	2.591364
17	6	0	-5.027426	-1.348346	3.132008
18	1	0	-5.433926	-3.164727	4.217950
19	1	0	-4.464849	-0.893224	3.952362
20	1	0	-5.595721	-0.538214	2.653271
21	1	0	-3.575650	-4.109875	0.145465
22	6	0	-0.431755	-3.888929	-1.857675
23	6	0	0.464700	-4.352366	-3.006753
24	6	0	0.343703	-3.953324	-0.538432
25	1	0	-1.298723	-4.569308	-1.793436
26	6	0	1.001804	-5.762784	-2.755445
27	1	0	1.305286	-3.648916	-3.075549
28	1	0	-0.087481	-4.303212	-3.952201
29	6	0	0.879973	-5.360216	-0.269759
30	1	0	1.177270	-3.242940	-0.623533
31	1	0	-0.291931	-3.608766	0.287430
32	6	0	1.759195	-5.836915	-1.427696

33	1	0	1.650726	-6.067062	-3.582993
34	1	0	0.164022	-6.473571	-2.733787
35	1	0	1.440154	-5.376167	0.671674
36	1	0	0.038012	-6.055844	-0.145945
37	1	0	2.111112	-6.858000	-1.246190
38	1	0	2.649630	-5.195708	-1.485376
39	6	0	-2.014476	-2.145208	-1.555736
40	6	0	-3.165782	-2.379675	-0.778085
41	1	0	0.447445	0.872666	-0.719901
42	1	0	-4.328923	0.422294	0.434051
43	1	0	-1.467561	3.089955	-1.730766
44	1	0	-5.134380	6.004509	-0.957888
45	1	0	-3.044595	5.007002	-1.842369
46	1	0	-1.860948	5.561053	0.609795
47	1	0	-0.801035	3.585553	1.583669
48	1	0	2.157310	2.811689	1.923494
49	1	0	3.378866	-2.766761	3.563737
50	1	0	3.143079	-0.607649	4.772084
51	1	0	1.903497	1.258731	3.737284
52	1	0	4.775489	-2.135946	-1.889704
53	1	0	5.791065	-3.408839	0.027116
54	1	0	6.147056	-2.263041	2.221966
55	16	0	4.324709	2.030581	0.558812
56	9	0	5.536276	0.160865	2.714392
57	9	0	4.132470	0.362214	-1.760651
58	8	0	-2.130704	1.343978	1.054914
59	8	0	4.395306	2.485018	1.936783
60	8	0	5.097210	2.706777	-0.470992
61	7	0	-3.182389	0.196445	-1.330662
62	7	0	2.743814	2.000093	0.135044
63	7	0	-0.066473	1.017664	0.147670
64	6	0	-4.375304	0.680774	-0.631557
65	6	0	-2.312002	2.413410	-1.907709
66	6	0	-1.982281	1.036832	-1.334556
67	6	0	-1.408393	1.144625	0.083978
68	6	0	0.691099	1.354763	1.333241
69	6	0	1.676862	2.502261	0.990586
70	6	0	-4.553922	2.173911	-0.758436
71	6	0	-5.726191	2.740899	-0.252063
72	6	0	-5.942304	4.110205	-0.318878
73	6	0	-4.976343	4.932213	-0.898918
74	6	0	-3.807340	4.372573	-1.397484
75	6	0	-3.579691	2.994393	-1.330545
76	6	0	1.431154	0.174967	1.936887

77	6	0	2.005935	0.317413	3.202266
78	6	0	2.703112	-0.734311	3.787942
79	6	0	2.836389	-1.943005	3.109259
80	6	0	2.281946	-2.084873	1.839651
81	6	0	1.583725	-1.031222	1.253515
82	6	0	0.985735	3.711390	0.363088
83	6	0	1.611605	4.434689	-0.654185
84	6	0	0.997215	5.551480	-1.216582
85	6	0	-0.252100	5.965916	-0.766699
86	6	0	-0.882852	5.254057	0.252324
87	6	0	-0.272481	4.134157	0.809940
88	6	0	4.797255	0.311545	0.476109
89	6	0	5.344881	-0.414566	1.528948
90	6	0	5.716633	-1.743598	1.373718
91	6	0	5.513709	-2.367109	0.148444
92	6	0	4.956414	-1.676380	-0.925940
93	6	0	4.627940	-0.347781	-0.741293
94	1	0	2.401139	-3.018051	1.295825
95	1	0	-0.730315	6.837626	-1.203405
96	1	0	1.502900	6.096174	-2.007823
97	1	0	2.591064	4.125875	-1.005043
98	1	0	2.548164	1.708778	-0.823871
99	1	0	-0.032628	1.716752	2.068024
100	1	0	-6.857745	4.535890	0.079492
101	1	0	-6.473901	2.093831	0.201694
102	1	0	-2.419739	2.308006	-2.993501
103	1	0	-5.239520	0.172710	-1.075538
104	1	0	1.154989	-1.152672	0.262761
105	1	0	-1.246272	0.548133	-1.982748
106	6	0	0.539144	-0.941623	-3.732873
107	1	0	0.121709	-0.104935	-4.283331
108	1	0	1.055606	-1.684998	-4.335523
109	7	0	1.383380	-0.487287	-2.698173
110	8	0	2.076670	-1.302367	-2.096385
111	8	0	1.303099	0.708441	-2.335402
112	1	0	-0.293887	-1.712813	-2.961231

G4-IM1

Zero-point correction= 0.93380 (a.u.)

Thermal correction to Gibbs Free Energy= 0.86753 (a.u.)

Sum of electronic and zero-point Energies= -3084.59948 (a.u.)

Sum of electronic and thermal Free Energies= -3084.66575 (a.u.)

Standard orientation:

Center Number	Atomic Number	Atomic Type	Coordinates (Angstroms) X	Y	Z
1	6	0	2.754378	-0.391098	1.413368
2	7	0	0.690346	-1.633213	2.411735
3	7	0	3.966073	-2.665903	0.992308
4	6	0	4.925312	-2.340408	-0.072667
5	6	0	5.955445	-3.459384	-0.187823
6	6	0	6.981306	-3.141109	-1.278690
7	6	0	6.302944	-2.867211	-2.622986
8	6	0	4.232681	-2.087239	-1.415526
9	1	0	5.430282	-4.391472	-0.443237
10	1	0	6.443642	-3.613271	0.779729
11	1	0	7.559250	-2.256843	-0.978667
12	1	0	7.693767	-3.966876	-1.368356
13	1	0	5.438214	-1.421204	0.243338
14	1	0	7.052503	-2.604415	-3.376168
15	1	0	3.490990	-1.284034	-1.319923
16	1	0	3.680801	-2.996639	-1.693841
17	6	0	5.260207	-1.754541	-2.498894
18	1	0	5.809157	-3.784527	-2.971429
19	1	0	4.753395	-1.588658	-3.453875
20	1	0	5.763571	-0.812018	-2.240839
21	1	0	3.616397	-3.617327	1.002012
22	6	0	0.250270	-3.017190	2.589551
23	6	0	-1.008850	-3.025972	3.455095
24	6	0	-0.020512	-3.704372	1.248862
25	1	0	1.050854	-3.560254	3.114072
26	6	0	-1.503003	-4.457296	3.675030
27	1	0	-1.777193	-2.433617	2.940419
28	1	0	-0.801424	-2.529404	4.409029
29	6	0	-0.501166	-5.141247	1.460237
30	1	0	-0.794882	-3.125544	0.725167
31	1	0	0.884892	-3.678405	0.626542
32	6	0	-1.750898	-5.169836	2.343212
33	1	0	-2.418477	-4.443424	4.273776
34	1	0	-0.752879	-5.015196	4.252576
35	1	0	-0.699796	-5.615554	0.493112
36	1	0	0.297221	-5.724360	1.940248
37	1	0	-2.067940	-6.203145	2.518476
38	1	0	-2.570010	-4.661367	1.820936
39	6	0	1.859727	-1.344018	1.885979
40	6	0	3.084174	-1.724682	1.360725

41	1	0	-0.592554	2.037820	0.784667
42	1	0	4.118056	0.847764	-0.602946
43	1	0	1.416916	3.872079	1.451172
44	1	0	5.019848	6.558054	-0.076251
45	1	0	3.002300	5.741304	1.103853
46	1	0	-2.688575	6.551678	-1.724835
47	1	0	-2.381320	4.205585	-2.431259
48	1	0	-3.063058	1.801190	-1.852462
49	1	0	-0.287452	-2.949197	-3.903546
50	1	0	-0.868125	-0.889255	-5.169370
51	1	0	-1.180377	1.250631	-3.968305
52	1	0	-3.388146	-4.658193	-0.807819
53	1	0	-3.484212	-4.441506	-3.301325
54	1	0	-3.819898	-2.174115	-4.326495
55	16	0	-4.261125	0.154811	-0.026257
56	9	0	-4.102845	-0.021544	-3.005292
57	9	0	-3.626295	-2.736697	0.826839
58	8	0	1.805973	1.554359	-1.217044
59	8	0	-5.222233	0.956739	-0.763658
60	8	0	-4.560679	-0.323447	1.310519
61	7	0	2.998425	0.894406	1.180306
62	7	0	-2.859691	0.961639	0.040714
63	7	0	-0.171968	1.895660	-0.141087
64	6	0	4.191196	1.257057	0.411220
65	6	0	2.258378	3.189216	1.604167
66	6	0	1.835507	1.784224	1.190252
67	6	0	1.165216	1.744243	-0.189531
68	6	0	-0.988755	1.753061	-1.331881
69	6	0	-2.472008	1.963454	-0.944785
70	6	0	4.389267	2.751377	0.317193
71	6	0	5.521932	3.216046	-0.355650
72	6	0	5.758414	4.576311	-0.496405
73	6	0	4.849455	5.491777	0.033821
74	6	0	3.719157	5.033160	0.696272
75	6	0	3.475800	3.664788	0.849694
76	6	0	-0.787808	0.415980	-2.027518
77	6	0	-0.938926	0.347173	-3.412548
78	6	0	-0.759740	-0.855679	-4.089398
79	6	0	-0.432272	-2.008341	-3.380899
80	6	0	-0.298453	-1.951314	-1.995237
81	6	0	-0.477250	-0.746775	-1.318856
82	6	0	-2.710174	3.389822	-0.465174
83	6	0	-3.009945	3.687736	0.862001
84	6	0	-3.182701	5.012931	1.259554

85	6	0	-3.062736	6.047422	0.337759
86	6	0	-2.772973	5.753282	-0.993882
87	6	0	-2.597799	4.432667	-1.389350
88	6	0	-3.958457	-1.308535	-1.034063
89	6	0	-3.955726	-1.218711	-2.427942
90	6	0	-3.801456	-2.319563	-3.253005
91	6	0	-3.607280	-3.567259	-2.671080
92	6	0	-3.550622	-3.700613	-1.289293
93	6	0	-3.716980	-2.573956	-0.496748
94	1	0	-0.051565	-2.850998	-1.439823
95	1	0	-3.201467	7.077693	0.650811
96	1	0	-3.413947	5.232683	2.297343
97	1	0	-3.117249	2.883176	1.582702
98	1	0	-2.181992	0.557066	0.725117
99	1	0	-0.722926	2.542060	-2.046290
100	1	0	6.642870	4.922451	-1.021727
101	1	0	6.221159	2.496233	-0.775780
102	1	0	2.468115	3.177249	2.679993
103	1	0	5.055418	0.805368	0.912929
104	1	0	-0.360727	-0.695735	-0.236065
105	1	0	1.122280	1.405577	1.927631
106	6	0	-2.565246	1.040952	3.669351
107	1	0	-2.775160	1.918567	4.261102
108	1	0	-3.142714	0.131261	3.695385
109	7	0	-1.573769	1.106279	2.837063
110	8	0	-1.296985	0.063434	2.068166
111	8	0	-0.853925	2.152327	2.669599
112	1	0	-0.044915	-0.879087	2.415692

G4-I-*re*-IM2

Zero-point correction= 1.30451 (a.u.)

Thermal correction to Gibbs Free Energy= 1.22122 (a.u.)

Sum of electronic and zero-point Energies= -4193.23422 (a.u.)

Sum of electronic and thermal Free Energies= -4193.31751 (a.u.)

Standard orientation:

Center Number	Atomic Number	Atomic Type	Coordinates (Angstroms) X	Y	Z
1	6	0	-3.477451	1.771158	0.455609
2	7	0	-1.736414	3.690673	0.270889
3	7	0	-5.292451	3.240035	-0.702719
4	6	0	-6.290092	2.270232	-1.172525

5	6	0	-7.541537	3.017541	-1.623471
6	6	0	-8.615879	2.037729	-2.101989
7	6	0	-8.083496	1.119488	-3.204412
8	6	0	-5.747709	1.369214	-2.285224
9	1	0	-7.266322	3.688380	-2.450894
10	1	0	-7.913766	3.642079	-0.804585
11	1	0	-8.946438	1.427521	-1.250961
12	1	0	-9.492181	2.592111	-2.451848
13	1	0	-6.551635	1.644836	-0.307099
14	1	0	-8.856182	0.404174	-3.504329
15	1	0	-4.853776	0.833260	-1.942388
16	1	0	-5.442322	2.008036	-3.125952
17	6	0	-6.821830	0.383187	-2.748438
18	1	0	-7.845803	1.721841	-4.091707
19	1	0	-6.428072	-0.246431	-3.551704
20	1	0	-7.074831	-0.289583	-1.916759
21	1	0	-5.244650	4.126038	-1.189314
22	6	0	-1.603961	5.035668	-0.279709
23	6	0	-1.648815	6.080119	0.840133
24	6	0	-0.281641	5.113861	-1.046681
25	1	0	-2.440702	5.201976	-0.973636
26	6	0	-1.385115	7.487724	0.302092
27	1	0	-0.882617	5.805586	1.576704
28	1	0	-2.618122	6.028644	1.348718
29	6	0	-0.012306	6.522839	-1.575316
30	1	0	0.513049	4.820858	-0.346275
31	1	0	-0.291235	4.374664	-1.855314
32	6	0	-0.051004	7.550805	-0.443693
33	1	0	-1.401198	8.209190	1.125497
34	1	0	-2.194787	7.774035	-0.384526
35	1	0	0.958912	6.549090	-2.080925
36	1	0	-0.769604	6.782638	-2.328678
37	1	0	0.118440	8.558219	-0.838503
38	1	0	0.766936	7.337220	0.257211
39	6	0	-2.823232	2.971216	0.214929
40	6	0	-4.152015	2.793638	-0.160399
41	1	0	-0.956469	3.383269	0.886832
42	1	0	-0.175084	-1.320761	-0.199520
43	1	0	-5.008067	-0.504667	-0.014349
44	1	0	-1.252501	-1.913474	1.749107
45	1	0	-4.349803	-4.640326	4.062216
46	1	0	-2.291725	-3.417407	3.446950
47	1	0	-4.540486	-4.593876	0.230713
48	1	0	-3.016574	-3.353052	-1.225237

49	1	0	-0.181103	-4.348710	-2.450267
50	1	0	2.840392	-0.330700	-5.539011
51	1	0	1.645313	-2.457866	-6.018969
52	1	0	0.011932	-3.350332	-4.411914
53	1	0	5.073499	-0.514148	-0.579070
54	1	0	6.088076	-0.371618	-2.868550
55	1	0	5.409421	-2.015611	-4.629362
56	16	0	2.341788	-4.425019	-1.573809
57	9	0	3.724319	-3.887887	-4.298951
58	9	0	3.324749	-2.291382	0.103200
59	8	0	-3.093217	-0.912445	-1.332771
60	8	0	1.919537	-5.143546	-2.763932
61	8	0	3.011467	-5.122555	-0.486382
62	7	0	-3.434168	0.529020	0.942310
63	7	0	1.051947	-3.629599	-0.966845
64	7	0	-0.948686	-1.485073	-0.842757
65	6	0	-4.684488	-0.225187	0.995896
66	6	0	-2.028206	-1.174963	1.975771
67	6	0	-2.178243	-0.203169	0.802576
68	6	0	-2.131442	-0.893644	-0.568936
69	6	0	-0.741189	-2.322287	-2.005382
70	6	0	-0.282045	-3.729156	-1.554281
71	6	0	-4.549197	-1.462225	1.849922
72	6	0	-5.708453	-2.174512	2.172869
73	6	0	-5.648462	-3.310093	2.968760
74	6	0	-4.412721	-3.750046	3.443942
75	6	0	-3.259061	-3.056480	3.107085
76	6	0	-3.307700	-1.906311	2.311683
77	6	0	0.255284	-1.738306	-2.996417
78	6	0	0.524535	-2.418339	-4.188619
79	6	0	1.445734	-1.913987	-5.101049
80	6	0	2.116671	-0.722859	-4.830719
81	6	0	1.853019	-0.041657	-3.646367
82	6	0	0.924424	-0.543625	-2.736471
83	6	0	-1.244956	-4.432385	-0.602256
84	6	0	-0.770296	-5.433760	0.249570
85	6	0	-1.644286	-6.145367	1.067324
86	6	0	-3.006083	-5.859862	1.054180
87	6	0	-3.485205	-4.851252	0.223331
88	6	0	-2.613993	-4.142972	-0.599065
89	6	0	3.471939	-3.129906	-2.074566
90	6	0	4.052249	-3.029679	-3.336165
91	6	0	4.989652	-2.046359	-3.630665
92	6	0	5.354436	-1.139116	-2.646087

93	6	0	4.794281	-1.200181	-1.370845
94	6	0	3.871819	-2.196790	-1.118370
95	1	0	2.367540	0.889872	-3.427192
96	1	0	-3.688287	-6.407096	1.697602
97	1	0	-1.254255	-6.922406	1.717642
98	1	0	0.291830	-5.660381	0.276185
99	1	0	1.127139	-3.409856	0.031894
100	1	0	-1.711887	-2.403502	-2.501759
101	1	0	-6.557144	-3.848666	3.218827
102	1	0	-6.667146	-1.821012	1.799402
103	1	0	-1.680389	-0.582760	2.831104
104	1	0	-5.441552	0.431836	1.438370
105	1	0	0.700910	0.009503	-1.830819
106	1	0	-1.355125	0.519990	0.856452
107	8	0	1.638021	-1.305614	3.382095
108	8	0	0.605785	1.719553	-0.311370
109	7	0	2.839896	2.209949	0.059344
110	7	0	1.134524	-0.602185	1.289551
111	8	0	1.102870	-2.829645	1.802771
112	6	0	1.652041	-2.275865	4.487274
113	6	0	2.008519	0.320048	1.134876
114	6	0	1.672564	1.495124	0.218706
115	6	0	3.440531	0.455882	1.443952
116	6	0	3.892317	1.591054	0.741696
117	6	0	4.342888	-0.337197	2.141249
118	1	0	4.008868	-1.231227	2.654527
119	6	0	1.322627	-1.682850	2.147699
120	6	0	5.689645	0.026411	2.152282
121	1	0	6.406465	-0.581682	2.692702
122	6	0	2.997102	3.266568	-0.921130
123	1	0	2.005971	3.398437	-1.367290
124	6	0	5.228731	1.963818	0.746262
125	1	0	5.567165	2.846754	0.215136
126	6	0	6.119206	1.163029	1.466687
127	1	0	7.170221	1.433604	1.486297
128	6	0	1.945606	-1.393576	5.692408
129	6	0	0.279908	-2.928528	4.614128
130	6	0	2.762400	-3.296936	4.270678
131	1	0	2.618059	-3.852321	3.342855
132	1	0	2.759783	-4.005846	5.103861
133	1	0	3.741684	-2.808808	4.253452
134	1	0	2.034658	-2.010279	6.590723
135	1	0	1.142098	-0.667817	5.845886
136	1	0	2.884425	-0.850776	5.548803

137	1	0	0.234189	-3.481286	5.557038
138	1	0	0.086471	-3.620848	3.793231
139	1	0	-0.498719	-2.159359	4.634300
140	6	0	1.491978	2.021630	3.141517
141	1	0	2.327155	2.702312	3.199697
142	1	0	1.425631	1.092698	3.685350
143	7	0	0.394876	2.457557	2.542448
144	8	0	-0.657529	1.757386	2.486626
145	8	0	0.403783	3.600725	1.949659
146	1	0	3.678514	2.920439	-1.709503
147	6	0	3.504513	4.573309	-0.349917
148	6	0	4.447887	5.314986	-1.061932
149	6	0	3.015678	5.061499	0.864844
150	6	0	4.898569	6.539423	-0.573882
151	1	0	4.832405	4.932142	-2.004714
152	6	0	3.473948	6.283574	1.352531
153	1	0	2.263910	4.491188	1.410398
154	6	0	4.412537	7.025123	0.637034
155	1	0	5.633163	7.108082	-1.135671
156	1	0	3.090714	6.659071	2.296759
157	1	0	4.766194	7.976281	1.022634

G4-I-*si*-IM2

Zero-point correction= 1.30451 (a.u.)

Thermal correction to Gibbs Free Energy= 1.21901 (a.u.)

Sum of electronic and zero-point Energies= -4193.22220 (a.u.)

Sum of electronic and thermal Free Energies= -4193.30770 (a.u.)

Standard orientation:

Center Number	Atomic Number	Atomic Type	Coordinates (Angstroms) X	Y	Z
1	6	0	-3.863363	1.274305	0.863643
2	7	0	-2.203152	3.269407	0.945369
3	7	0	-5.790688	2.821513	0.041269
4	6	0	-6.728751	1.876719	-0.578898
5	6	0	-8.060794	2.576437	-0.831866
6	6	0	-9.072540	1.611461	-1.456031
7	6	0	-8.523841	0.972300	-2.733838
8	6	0	-6.172552	1.261399	-1.866464
9	1	0	-7.887044	3.417617	-1.519481
10	1	0	-8.442947	2.993597	0.105544
11	1	0	-9.307092	0.822104	-0.729353

12	1	0	-10.008880	2.139597	-1.661276
13	1	0	-6.889108	1.074569	0.155849
14	1	0	-9.247573	0.256894	-3.137304
15	1	0	-5.215900	0.761087	-1.670136
16	1	0	-5.969975	2.075503	-2.576827
17	6	0	-7.181728	0.284818	-2.473954
18	1	0	-8.386546	1.750526	-3.496610
19	1	0	-6.778483	-0.140433	-3.397767
20	1	0	-7.333096	-0.554653	-1.780263
21	1	0	-5.786555	3.763794	-0.329519
22	6	0	-2.161963	4.690784	0.613332
23	6	0	-1.848396	5.511023	1.867799
24	6	0	-1.108379	4.935444	-0.472025
25	1	0	-3.151955	4.977084	0.228565
26	6	0	-1.723716	7.000498	1.540338
27	1	0	-0.905642	5.130352	2.281895
28	1	0	-2.625798	5.338698	2.619918
29	6	0	-0.993780	6.425669	-0.800686
30	1	0	-0.148197	4.555226	-0.094102
31	1	0	-1.356449	4.348984	-1.363155
32	6	0	-0.674975	7.242927	0.452836
33	1	0	-1.472119	7.560438	2.446573
34	1	0	-2.695697	7.379665	1.194093
35	1	0	-0.230704	6.582716	-1.570907
36	1	0	-1.943511	6.777329	-1.227797
37	1	0	-0.617249	8.309096	0.208965
38	1	0	0.310030	6.944924	0.835722
39	6	0	-3.269689	2.529083	0.815242
40	6	0	-4.606352	2.353619	0.467316
41	1	0	-1.306465	2.874830	1.360336
42	1	0	-0.318048	-1.497051	-0.122923
43	1	0	-5.083281	-0.967627	-0.168460
44	1	0	-1.144512	-2.167092	1.683561
45	1	0	-3.818792	-5.977197	2.543821
46	1	0	-2.008609	-4.282229	2.612909
47	1	0	-2.070247	-6.580214	-3.548727
48	1	0	-0.761967	-4.526808	-3.995335
49	1	0	0.867537	-2.953950	-3.246929
50	1	0	1.815519	2.623842	-4.506452
51	1	0	1.148127	0.664605	-5.882738
52	1	0	0.076274	-1.288179	-4.816541
53	1	0	4.266475	1.353735	-2.894126
54	1	0	5.050959	0.709560	-5.200025
55	1	0	4.898685	-1.673982	-5.935022

56	16	0	3.032004	-3.158465	-1.586146
57	9	0	4.011188	-3.585325	-4.502171
58	9	0	3.306257	-0.314761	-1.196637
59	8	0	-3.024573	-0.628496	-1.441066
60	8	0	2.910354	-4.458897	-2.210973
61	8	0	3.819951	-2.965625	-0.380702
62	7	0	-3.695591	-0.026087	1.109238
63	7	0	1.510668	-2.617605	-1.255284
64	7	0	-1.001981	-1.496956	-0.871411
65	6	0	-4.816491	-0.939647	0.896134
66	6	0	-2.109108	-1.684877	1.871945
67	6	0	-2.350617	-0.544834	0.890594
68	6	0	-2.182783	-0.900583	-0.595017
69	6	0	-0.528379	-1.688263	-2.224335
70	6	0	0.445585	-2.891826	-2.234521
71	6	0	-4.495790	-2.340987	1.370291
72	6	0	-5.510261	-3.300861	1.326662
73	6	0	-5.276313	-4.603341	1.745183
74	6	0	-4.012503	-4.960817	2.214789
75	6	0	-2.998859	-4.013012	2.250969
76	6	0	-3.223459	-2.699629	1.827239
77	6	0	0.123077	-0.448559	-2.831383
78	6	0	0.371146	-0.434200	-4.209270
79	6	0	0.970301	0.665494	-4.811484
80	6	0	1.340676	1.766237	-4.037604
81	6	0	1.113730	1.753354	-2.665843
82	6	0	0.501073	0.652982	-2.063065
83	6	0	-0.275871	-4.188595	-1.928315
84	6	0	-0.416563	-4.660630	-0.624075
85	6	0	-1.148616	-5.817292	-0.372194
86	6	0	-1.750172	-6.509015	-1.420241
87	6	0	-1.611030	-6.042703	-2.724922
88	6	0	-0.876136	-4.888003	-2.975328
89	6	0	3.654136	-1.994162	-2.793525
90	6	0	4.079225	-2.324958	-4.078333
91	6	0	4.579241	-1.364280	-4.947107
92	6	0	4.659860	-0.042983	-4.523899
93	6	0	4.237910	0.331158	-3.250136
94	6	0	3.741315	-0.652049	-2.419315
95	1	0	1.426631	2.584109	-2.040520
96	1	0	-2.319960	-7.411158	-1.220814
97	1	0	-1.249210	-6.179200	0.646301
98	1	0	0.064507	-4.125714	0.189907
99	1	0	1.550786	-1.665483	-0.882350

100	1	0	-1.394791	-1.963386	-2.833474
101	1	0	-6.073953	-5.338404	1.704511
102	1	0	-6.493808	-3.016138	0.958873
103	1	0	-2.032180	-1.230997	2.866264
104	1	0	-5.672398	-0.551928	1.460357
105	1	0	0.342471	0.668379	-0.988415
106	1	0	-1.631728	0.249660	1.133239
107	6	0	1.318280	1.731613	3.548967
108	1	0	2.052702	2.517598	3.469544
109	1	0	1.327522	0.954697	4.297826
110	7	0	0.246609	1.837506	2.788438
111	8	0	-0.726594	1.017642	2.868622
112	8	0	0.183397	2.785163	1.918763
113	8	0	3.155888	3.637090	-0.732681
114	8	0	1.002629	-0.507962	0.844012
115	7	0	2.789443	-1.119911	2.153324
116	7	0	2.438203	1.941566	0.517464
117	8	0	3.320061	3.841937	1.519241
118	1	0	3.307930	-3.122262	2.299764
119	6	0	3.719474	4.950907	-1.019710
120	6	0	2.835663	1.052982	1.339388
121	6	0	2.064140	-0.257184	1.396919
122	6	0	4.011096	0.844380	2.214512
123	6	0	3.937039	-0.490565	2.660138
124	6	0	5.087429	1.636140	2.597896
125	1	0	5.150045	2.668535	2.277744
126	6	0	3.030382	3.207602	0.527996
127	6	0	6.067950	1.084774	3.424498
128	1	0	6.909689	1.694116	3.734932
129	6	0	2.408073	-2.504238	2.358628
130	1	0	1.786874	-2.772324	1.497245
131	6	0	4.894416	-1.044986	3.491176
132	1	0	4.809307	-2.066574	3.845374
133	6	0	5.967109	-0.232759	3.866232
134	1	0	6.731905	-0.639604	4.520186
135	6	0	1.661404	-2.732082	3.653832
136	6	0	1.972826	-3.831093	4.452660
137	6	0	0.628085	-1.871025	4.037430
138	6	0	1.258357	-4.078773	5.623021
139	1	0	2.778600	-4.497832	4.154699
140	6	0	-0.081634	-2.118257	5.209721
141	1	0	0.363729	-1.007559	3.425884
142	6	0	0.229534	-3.222293	6.003345
143	1	0	1.510715	-4.937320	6.237739

144	1	0	-0.880441	-1.441941	5.500635
145	1	0	-0.326068	-3.411621	6.916592
146	6	0	2.845309	6.042248	-0.412846
147	1	0	2.883492	6.026939	0.676373
148	1	0	3.188226	7.019595	-0.765952
149	1	0	1.809112	5.906232	-0.736731
150	6	0	3.658828	5.014004	-2.540918
151	1	0	4.239128	4.200867	-2.986694
152	1	0	2.622206	4.933045	-2.882154
153	1	0	4.065907	5.964734	-2.895035
154	6	0	5.161268	5.020311	-0.528878
155	1	0	5.626482	5.938940	-0.898011
156	1	0	5.208019	5.021265	0.560647
157	1	0	5.733611	4.169269	-0.911283

G4-I-*re*-TS2

Zero-point correction= 1.30502 (a.u.)

Thermal correction to Gibbs Free Energy= 1.22388 (a.u.)

Sum of electronic and zero-point Energies= -4193.23372 (a.u.)

Sum of electronic and thermal Free Energies= -4193.31487 (a.u.)

Standard orientation:

Center Number	Atomic Number	Atomic Type	Coordinates (Angstroms) X	Y	Z
1	6	0	-3.374544	2.014683	0.557224
2	7	0	-1.513627	3.823486	0.420960
3	7	0	-5.083308	3.615247	-0.586228
4	6	0	-6.139313	2.718994	-1.073856
5	6	0	-7.338030	3.549936	-1.521164
6	6	0	-8.469285	2.646297	-2.018811
7	6	0	-7.989057	1.712267	-3.131881
8	6	0	-5.648281	1.801034	-2.196057
9	1	0	-7.016442	4.214289	-2.337018
10	1	0	-7.676351	4.184754	-0.695358
11	1	0	-8.841536	2.045862	-1.177973
12	1	0	-9.307404	3.259618	-2.364031
13	1	0	-6.445544	2.099178	-0.219159
14	1	0	-8.803592	1.052030	-3.446740
15	1	0	-4.792793	1.203887	-1.856609
16	1	0	-5.298328	2.430723	-3.026235
17	6	0	-6.779871	0.891567	-2.678542
18	1	0	-7.707323	2.311493	-4.008303

19	1	0	-6.421051	0.249120	-3.488111
20	1	0	-7.080764	0.224605	-1.858062
21	1	0	-4.983223	4.508993	-1.049837
22	6	0	-1.243573	5.108198	-0.216049
23	6	0	-1.023882	6.199054	0.835853
24	6	0	-0.006694	4.961602	-1.105824
25	1	0	-2.115528	5.365185	-0.834942
26	6	0	-0.640341	7.528128	0.180642
27	1	0	-0.221210	5.860823	1.504210
28	1	0	-1.927871	6.306751	1.445439
29	6	0	0.379936	6.287510	-1.760483
30	1	0	0.808746	4.611349	-0.457579
31	1	0	-0.183344	4.177914	-1.850453
32	6	0	0.596886	7.374880	-0.707058
33	1	0	-0.468010	8.285392	0.952104
34	1	0	-1.480331	7.886978	-0.431383
35	1	0	1.285316	6.153761	-2.362630
36	1	0	-0.416698	6.601133	-2.449976
37	1	0	0.840222	8.328438	-1.187689
38	1	0	1.458550	7.098336	-0.084860
39	6	0	-2.642535	3.173551	0.339491
40	6	0	-3.978033	3.089557	-0.044582
41	1	0	-0.752285	3.442143	1.012733
42	1	0	-0.283424	-1.279162	-0.174076
43	1	0	-5.054063	-0.133910	0.040252
44	1	0	-1.372982	-1.821021	1.767971
45	1	0	-4.634794	-4.464880	3.935803
46	1	0	-2.508088	-3.334235	3.378244
47	1	0	-4.890142	-4.278086	0.099686
48	1	0	-3.274403	-3.090244	-1.302541
49	1	0	-0.505438	-4.217954	-2.538918
50	1	0	2.847913	-0.330189	-5.435597
51	1	0	1.493330	-2.333479	-6.015211
52	1	0	-0.224699	-3.158279	-4.461901
53	1	0	5.060616	-0.903448	-0.560819
54	1	0	6.084522	-0.799863	-2.847947
55	1	0	5.249311	-2.324645	-4.649113
56	16	0	1.992942	-4.535042	-1.643627
57	9	0	3.399101	-4.038491	-4.360622
58	9	0	3.151396	-2.520558	0.082439
59	8	0	-3.163283	-0.632362	-1.297081
60	8	0	1.521049	-5.194029	-2.849786
61	8	0	2.598381	-5.307603	-0.568514
62	7	0	-3.410814	0.762827	1.018972

63	7	0	0.769611	-3.651144	-1.025661
64	7	0	-1.067690	-1.372505	-0.820294
65	6	0	-4.706722	0.087054	1.057188
66	6	0	-2.113675	-1.050867	2.004712
67	6	0	-2.203998	-0.045153	0.855810
68	6	0	-2.204656	-0.704392	-0.532150
69	6	0	-0.914909	-2.177152	-2.014045
70	6	0	-0.564853	-3.630402	-1.618149
71	6	0	-4.644952	-1.188805	1.861311
72	6	0	-5.843869	-1.847222	2.150950
73	6	0	-5.851382	-3.018324	2.896340
74	6	0	-4.645060	-3.546890	3.356311
75	6	0	-3.452560	-2.906179	3.051954
76	6	0	-3.432996	-1.722345	2.306750
77	6	0	0.131915	-1.633080	-2.975035
78	6	0	0.358704	-2.281348	-4.193583
79	6	0	1.328279	-1.815183	-5.075794
80	6	0	2.088299	-0.693308	-4.749771
81	6	0	1.866730	-0.043983	-3.539317
82	6	0	0.892465	-0.508973	-2.658318
83	6	0	-1.581384	-4.297605	-0.696418
84	6	0	-1.179211	-5.350185	0.130431
85	6	0	-2.104913	-6.029305	0.918274
86	6	0	-3.445800	-5.658742	0.900813
87	6	0	-3.852397	-4.598546	0.095923
88	6	0	-2.929621	-3.922703	-0.697045
89	6	0	3.226577	-3.325308	-2.113264
90	6	0	3.809024	-3.242986	-3.375152
91	6	0	4.832348	-2.343454	-3.649013
92	6	0	5.282859	-1.501156	-2.642300
93	6	0	4.719324	-1.541578	-1.367955
94	6	0	3.707452	-2.452977	-1.136510
95	1	0	2.449596	0.834750	-3.277241
96	1	0	-4.168214	-6.181947	1.519878
97	1	0	-1.771869	-6.848715	1.547923
98	1	0	-0.133389	-5.642600	0.161118
99	1	0	0.857192	-3.453409	-0.022769
100	1	0	-1.885034	-2.165844	-2.517948
101	1	0	-6.790188	-3.515515	3.119724
102	1	0	-6.779756	-1.426934	1.788681
103	1	0	-1.747114	-0.499358	2.878842
104	1	0	-5.417762	0.773761	1.529877
105	1	0	0.706644	0.018471	-1.729450
106	1	0	-1.335663	0.621744	0.921131

107	8	0	1.599755	-1.493316	3.343352
108	8	0	0.732663	1.733003	-0.221114
109	7	0	3.008583	2.017186	0.103108
110	7	0	1.103812	-0.684692	1.282936
111	8	0	0.864001	-2.908285	1.745442
112	6	0	1.571008	-2.502324	4.410755
113	6	0	2.034679	0.197054	1.180933
114	6	0	1.786199	1.405424	0.283352
115	6	0	3.482593	0.206849	1.462432
116	6	0	4.016169	1.308840	0.764279
117	6	0	4.330696	-0.660996	2.138292
118	1	0	3.934576	-1.529772	2.649864
119	6	0	1.208436	-1.796269	2.107183
120	6	0	5.702392	-0.404614	2.132751
121	1	0	6.375375	-1.073646	2.657576
122	6	0	3.243512	3.062537	-0.872880
123	1	0	2.275577	3.234577	-1.355482
124	6	0	5.377404	1.576773	0.753724
125	1	0	5.776716	2.437044	0.227131
126	6	0	6.212619	0.701996	1.453941
127	1	0	7.281557	0.889940	1.463353
128	6	0	1.968329	-1.691903	5.637057
129	6	0	0.158565	-3.057079	4.564426
130	6	0	2.593660	-3.595196	4.121687
131	1	0	2.400460	-4.081326	3.164218
132	1	0	2.539161	-4.350655	4.911196
133	1	0	3.609416	-3.188121	4.122220
134	1	0	2.054557	-2.350336	6.505507
135	1	0	1.218493	-0.925649	5.853074
136	1	0	2.932690	-1.201321	5.475147
137	1	0	0.096434	-3.612963	5.504614
138	1	0	-0.101811	-3.726471	3.743612
139	1	0	-0.563948	-2.235725	4.607532
140	6	0	1.661933	1.798668	3.137399
141	1	0	2.540549	2.420992	3.211656
142	1	0	1.523933	0.882905	3.689872
143	7	0	0.572063	2.358650	2.614549
144	8	0	-0.527267	1.743297	2.576804
145	8	0	0.653256	3.516635	2.073946
146	1	0	3.936109	2.682929	-1.635362
147	6	0	3.788078	4.349269	-0.288540
148	6	0	4.734575	5.083527	-1.004582
149	6	0	3.332975	4.825962	0.943043
150	6	0	5.220577	6.288436	-0.502428

151	1	0	5.094235	4.710148	-1.960890
152	6	0	3.827843	6.027498	1.446282
153	1	0	2.576876	4.266207	1.491557
154	6	0	4.769300	6.761391	0.727205
155	1	0	5.957125	6.851544	-1.067249
156	1	0	3.469113	6.392983	2.403902
157	1	0	5.151438	7.696911	1.123719

G4-I-*si*-TS2

Zero-point correction= 1.30570 (a.u.)

Thermal correction to Gibbs Free Energy= 1.22196 (a.u.)

Sum of electronic and zero-point Energies= -4193.22011 (a.u.)

Sum of electronic and thermal Free Energies= -4193.30385 (a.u.)

Standard orientation:

Center Number	Atomic Number	Atomic Type	Coordinates (Angstroms) X	Y	Z
1	6	0	3.853106	1.160453	-0.838762
2	7	0	2.255478	3.213482	-0.875864
3	7	0	5.822673	2.647064	-0.006878
4	6	0	6.767024	1.672396	0.553531
5	6	0	8.117302	2.345548	0.780025
6	6	0	9.134959	1.346226	1.337329
7	6	0	8.621392	0.675026	2.613348
8	6	0	6.246471	1.029624	1.842246
9	1	0	7.980605	3.166534	1.499503
10	1	0	8.473090	2.786179	-0.156975
11	1	0	9.331077	0.577239	0.578234
12	1	0	10.086073	1.853788	1.525283
13	1	0	6.889824	0.889247	-0.208515
14	1	0	9.346890	-0.063766	2.968540
15	1	0	5.272880	0.554782	1.667985
16	1	0	6.087350	1.825199	2.583631
17	6	0	7.259358	0.017244	2.380968
18	1	0	8.523614	1.430892	3.404261
19	1	0	6.882322	-0.430314	3.305156
20	1	0	7.370803	-0.801758	1.656001
21	1	0	5.855269	3.586697	0.368418
22	6	0	2.279917	4.636378	-0.543178
23	6	0	2.141689	5.481876	-1.810997
24	6	0	1.164092	4.950043	0.456994
25	1	0	3.252574	4.843478	-0.073532

26	6	0	2.116964	6.974114	-1.474708
27	1	0	1.208444	5.187500	-2.308967
28	1	0	2.963523	5.247055	-2.496253
29	6	0	1.135178	6.444329	0.787839
30	1	0	0.210515	4.635869	0.008387
31	1	0	1.305696	4.347597	1.360786
32	6	0	1.002587	7.295061	-0.477012
33	1	0	1.991017	7.559680	-2.390801
34	1	0	3.085008	7.265182	-1.042673
35	1	0	0.316127	6.657633	1.483595
36	1	0	2.063545	6.716512	1.309677
37	1	0	1.015440	8.359794	-0.220957
38	1	0	0.033805	7.091182	-0.951804
39	6	0	3.296451	2.428166	-0.767247
40	6	0	4.627083	2.214838	-0.427447
41	1	0	1.382185	2.864652	-1.309820
42	1	0	0.254594	-1.530683	0.131183
43	1	0	5.023655	-1.107903	0.153603
44	1	0	1.039117	-2.193921	-1.688393
45	1	0	3.594247	-6.093511	-2.511144
46	1	0	1.836140	-4.344879	-2.592626
47	1	0	1.917390	-6.618987	3.543903
48	1	0	0.659602	-4.534716	3.993435
49	1	0	-0.974093	-2.944718	3.252165
50	1	0	-1.736885	2.668860	4.501772
51	1	0	-1.110136	0.702332	5.887502
52	1	0	-0.101927	-1.287492	4.827143
53	1	0	-4.240175	1.512766	2.769272
54	1	0	-5.049994	0.965770	5.090889
55	1	0	-4.974109	-1.398526	5.900704
56	16	0	-3.145409	-3.073148	1.605697
57	9	0	-4.140973	-3.379220	4.533090
58	9	0	-3.338906	-0.240133	1.124721
59	8	0	2.980999	-0.714378	1.436787
60	8	0	-3.060249	-4.357635	2.269718
61	8	0	-3.926289	-2.897572	0.393569
62	7	0	3.651669	-0.129084	-1.112242
63	7	0	-1.605993	-2.589802	1.261764
64	7	0	0.941169	-1.551308	0.877561
65	6	0	4.748973	-1.075190	-0.908505
66	6	0	2.019540	-1.744670	-1.873631
67	6	0	2.294366	-0.615087	-0.889776
68	6	0	2.128355	-0.973407	0.597607
69	6	0	0.462681	-1.723711	2.232179

70	6	0	-0.549195	-2.894458	2.240573
71	6	0	4.384729	-2.468926	-1.373454
72	6	0	5.369406	-3.459055	-1.321688
73	6	0	5.094711	-4.757288	-1.728387
74	6	0	3.819817	-5.080398	-2.192773
75	6	0	2.835253	-4.102695	-2.236054
76	6	0	3.101520	-2.793441	-1.824328
77	6	0	-0.147850	-0.462674	2.836038
78	6	0	-0.380867	-0.431057	4.215830
79	6	0	-0.944485	0.689494	4.814324
80	6	0	-1.292719	1.793737	4.035188
81	6	0	-1.084272	1.762775	2.660604
82	6	0	-0.507388	0.641110	2.062503
83	6	0	0.135503	-4.211247	1.933502
84	6	0	0.239154	-4.700598	0.632360
85	6	0	0.942522	-5.874832	0.378899
86	6	0	1.552552	-6.566542	1.421928
87	6	0	1.451093	-6.082132	2.723654
88	6	0	0.744447	-4.910454	2.975792
89	6	0	-3.733061	-1.854444	2.775124
90	6	0	-4.170331	-2.131427	4.068309
91	6	0	-4.644036	-1.129490	4.904355
92	6	0	-4.681918	0.180080	4.439949
93	6	0	-4.245986	0.501055	3.156757
94	6	0	-3.780301	-0.522872	2.357208
95	1	0	-1.384370	2.592967	2.028016
96	1	0	2.100390	-7.481976	1.221638
97	1	0	1.013746	-6.250278	-0.637247
98	1	0	-0.248928	-4.165865	-0.177701
99	1	0	-1.608079	-1.645083	0.867199
100	1	0	1.319001	-2.026462	2.842487
101	1	0	5.869371	-5.516129	-1.681136
102	1	0	6.361593	-3.202370	-0.956364
103	1	0	1.963349	-1.292576	-2.870455
104	1	0	5.609923	-0.712938	-1.481711
105	1	0	-0.365514	0.643122	0.985713
106	1	0	1.593276	0.199568	-1.112921
107	6	0	-1.512506	1.733612	-3.353522
108	1	0	-2.173719	2.583166	-3.433495
109	1	0	-1.516561	0.910118	-4.053227
110	7	0	-0.348793	1.975668	-2.723246
111	8	0	0.611433	1.155293	-2.768286
112	8	0	-0.254161	3.017235	-2.011717
113	8	0	-2.841943	3.825957	0.659406

114	8	0	-0.969388	-0.473810	-0.860725
115	7	0	-2.787845	-1.075374	-2.124032
116	7	0	-2.373881	2.001415	-0.524272
117	8	0	-3.355885	3.847135	-1.545445
118	1	0	-3.344954	-3.071791	-2.213577
119	6	0	-3.383764	5.149442	0.908415
120	6	0	-2.729576	1.147329	-1.433833
121	6	0	-2.025578	-0.198780	-1.419805
122	6	0	-3.966101	0.909770	-2.236849
123	6	0	-3.939370	-0.446306	-2.619485
124	6	0	-5.049189	1.695353	-2.615043
125	1	0	-5.081907	2.741879	-2.343526
126	6	0	-2.916345	3.270639	-0.566039
127	6	0	-6.074651	1.119717	-3.369058
128	1	0	-6.921191	1.728740	-3.667935
129	6	0	-2.435534	-2.471247	-2.294811
130	1	0	-1.812813	-2.732717	-1.432580
131	6	0	-4.938510	-1.024889	-3.383376
132	1	0	-4.884639	-2.065162	-3.685914
133	6	0	-6.015924	-0.217473	-3.752689
134	1	0	-6.815592	-0.643710	-4.350243
135	6	0	-1.698782	-2.746451	-3.587288
136	6	0	-2.002417	-3.881360	-4.338156
137	6	0	-0.675174	-1.894385	-4.014176
138	6	0	-1.290189	-4.171128	-5.500332
139	1	0	-2.800165	-4.542923	-4.009003
140	6	0	0.032971	-2.183252	-5.177602
141	1	0	-0.417051	-1.007845	-3.435159
142	6	0	-0.270694	-3.322631	-5.921819
143	1	0	-1.536933	-5.056673	-6.077867
144	1	0	0.825440	-1.514502	-5.501070
145	1	0	0.283520	-3.545254	-6.828249
146	6	0	-2.636632	6.197275	0.090591
147	1	0	-2.813010	6.068740	-0.977200
148	1	0	-2.963221	7.198026	0.390550
149	1	0	-1.563948	6.115534	0.285716
150	6	0	-3.107929	5.347332	2.394952
151	1	0	-3.617183	4.579227	2.984775
152	1	0	-2.033303	5.282095	2.593465
153	1	0	-3.463892	6.328247	2.721466
154	6	0	-4.885020	5.166367	0.636638
155	1	0	-5.308794	6.110629	0.991557
156	1	0	-5.094913	5.066362	-0.428677
157	1	0	-5.375271	4.348455	1.174310

G4-I-*re*-IM3

Zero-point correction= 1.30727 (a.u.)
Thermal correction to Gibbs Free Energy= 1.22556 (a.u.)
Sum of electronic and zero-point Energies= -4193.26647 (a.u.)
Sum of electronic and thermal Free Energies= -4193.34817 (a.u.)

Standard orientation:

Center Number	Atomic Number	Atomic Type	Coordinates (Angstroms) X	Y	Z
1	6	0	-4.058629	-0.090351	0.696493
2	7	0	-3.455637	2.425603	0.895973
3	7	0	-6.386266	0.465874	-0.334101
4	6	0	-6.762215	-0.788897	-1.003073
5	6	0	-8.229270	-0.722978	-1.415968
6	6	0	-8.656388	-2.027384	-2.095073
7	6	0	-7.753405	-2.361539	-3.284821
8	6	0	-5.858061	-1.101609	-2.198441
9	1	0	-8.358757	0.113023	-2.119001
10	1	0	-8.851312	-0.513678	-0.539473
11	1	0	-8.605642	-2.843783	-1.362257
12	1	0	-9.700924	-1.953533	-2.413226
13	1	0	-6.648157	-1.586230	-0.255688
14	1	0	-8.056421	-3.314297	-3.730506
15	1	0	-4.807712	-1.150125	-1.883598
16	1	0	-5.943481	-0.275152	-2.918537
17	6	0	-6.283051	-2.411422	-2.864724
18	1	0	-7.879195	-1.592818	-4.059106
19	1	0	-5.640253	-2.616012	-3.725985
20	1	0	-6.134744	-3.240158	-2.157933
21	1	0	-6.781928	1.319601	-0.708099
22	6	0	-3.882936	3.752819	0.456614
23	6	0	-4.236655	4.623148	1.665226
24	6	0	-2.753487	4.374357	-0.368837
25	1	0	-4.773902	3.617578	-0.171995
26	6	0	-4.559802	6.057121	1.242142
27	1	0	-3.373189	4.620969	2.343263
28	1	0	-5.072820	4.169354	2.207876
29	6	0	-3.049681	5.821061	-0.762460
30	1	0	-1.842815	4.341640	0.245376
31	1	0	-2.562193	3.750217	-1.248745
32	6	0	-3.394716	6.668701	0.462250

33	1	0	-4.792794	6.659703	2.125731
34	1	0	-5.460611	6.058320	0.612576
35	1	0	-2.180694	6.239971	-1.282504
36	1	0	-3.890817	5.845361	-1.469334
37	1	0	-3.629495	7.694939	0.160572
38	1	0	-2.514062	6.720130	1.116220
39	6	0	-4.079184	1.294785	0.657992
40	6	0	-5.163542	0.562764	0.202724
41	1	0	-2.526015	2.359365	1.302095
42	1	0	0.403343	-0.814243	-0.007427
43	1	0	-4.001065	-2.718873	-0.290579
44	1	0	-0.234392	-2.273719	1.585103
45	1	0	-1.553038	-6.431786	3.511578
46	1	0	-0.378479	-4.286781	3.117216
47	1	0	-2.628973	-5.538793	-1.451077
48	1	0	-1.365411	-3.623359	-2.292182
49	1	0	1.673721	-3.237152	-2.709182
50	1	0	3.459394	1.971143	-4.638820
51	1	0	2.057887	0.347068	-5.896523
52	1	0	0.647975	-1.273876	-4.673614
53	1	0	5.679083	1.306060	-0.168755
54	1	0	6.865793	1.576255	-2.369560
55	1	0	6.669324	-0.218482	-4.102678
56	16	0	3.879473	-3.102615	-1.193325
57	9	0	5.381134	-2.381800	-3.815627
58	9	0	4.240120	-0.727492	0.441975
59	8	0	-2.345736	-1.538342	-1.439400
60	8	0	3.848482	-3.977102	-2.354586
61	8	0	4.475776	-3.559536	0.054164
62	7	0	-3.379754	-1.187455	1.013322
63	7	0	2.375209	-2.565213	-0.887176
64	7	0	-0.187851	-1.193926	-0.777180
65	6	0	-4.005312	-2.490979	0.782774
66	6	0	-1.267432	-2.079320	1.886864
67	6	0	-1.921399	-1.104775	0.906588
68	6	0	-1.509780	-1.303224	-0.565486
69	6	0	0.410523	-1.639566	-2.019373
70	6	0	1.253012	-2.914900	-1.750201
71	6	0	-3.293472	-3.578821	1.549288
72	6	0	-3.946987	-4.797677	1.751751
73	6	0	-3.334707	-5.822512	2.459739
74	6	0	-2.047028	-5.635438	2.963055
75	6	0	-1.389527	-4.433098	2.744728
76	6	0	-2.001502	-3.389224	2.041783

77	6	0	1.238626	-0.585572	-2.724713
78	6	0	1.259344	-0.564169	-4.120898
79	6	0	2.052730	0.348178	-4.810880
80	6	0	2.835264	1.259420	-4.106467
81	6	0	2.820988	1.244073	-2.713323
82	6	0	2.033510	0.324074	-2.023438
83	6	0	0.430002	-4.067021	-1.171645
84	6	0	0.992604	-4.946712	-0.241659
85	6	0	0.271324	-6.040854	0.228531
86	6	0	-1.029342	-6.269665	-0.208774
87	6	0	-1.607068	-5.386926	-1.115276
88	6	0	-0.882131	-4.300703	-1.597316
89	6	0	4.796226	-1.633359	-1.647628
90	6	0	5.444571	-1.449649	-2.866673
91	6	0	6.184832	-0.304887	-3.137247
92	6	0	6.286045	0.682037	-2.166113
93	6	0	5.640949	0.544428	-0.938922
94	6	0	4.904515	-0.602125	-0.713739
95	1	0	3.442200	1.938428	-2.153293
96	1	0	-1.595184	-7.115210	0.169635
97	1	0	0.728827	-6.711062	0.950331
98	1	0	1.998741	-4.776106	0.128675
99	1	0	2.193843	-2.393761	0.128204
100	1	0	-0.416280	-1.904625	-2.681160
101	1	0	-3.856522	-6.760773	2.621257
102	1	0	-4.952401	-4.931875	1.358155
103	1	0	-1.214717	-1.581682	2.863059
104	1	0	-5.041805	-2.423719	1.128480
105	1	0	2.059409	0.290460	-0.934762
106	1	0	-1.611353	-0.091053	1.176782
107	8	0	2.522535	-0.423791	3.237569
108	8	0	-0.642881	1.834369	0.239261
109	7	0	1.297804	2.991934	-0.165775
110	7	0	1.273279	0.017291	1.362708
111	8	0	1.985337	-2.087252	1.795697
112	6	0	3.282000	-1.329007	4.085332
113	6	0	1.365842	1.424183	1.625945
114	6	0	0.519837	2.089529	0.500171
115	6	0	2.717720	2.111854	1.417559
116	6	0	2.594504	3.034770	0.370181
117	6	0	3.917612	2.030276	2.105926
118	1	0	4.026761	1.322697	2.917867
119	6	0	1.926800	-0.876280	2.088385
120	6	0	4.975220	2.870936	1.737565

121	1	0	5.915914	2.812386	2.275570
122	6	0	0.845162	3.730842	-1.323345
123	1	0	-0.105256	3.272776	-1.617114
124	6	0	3.630279	3.873051	-0.016964
125	1	0	3.498634	4.595259	-0.816404
126	6	0	4.832127	3.777125	0.689612
127	1	0	5.659780	4.425406	0.419552
128	6	0	3.665432	-0.446994	5.270501
129	6	0	2.416580	-2.489757	4.574796
130	6	0	4.526851	-1.813438	3.349189
131	1	0	4.262076	-2.399124	2.467441
132	1	0	5.129734	-2.433724	4.020412
133	1	0	5.137778	-0.960692	3.034731
134	1	0	4.277182	-1.014126	5.977425
135	1	0	2.771029	-0.095072	5.794482
136	1	0	4.242009	0.424344	4.945588
137	1	0	2.907113	-2.972499	5.426294
138	1	0	2.260599	-3.224699	3.787560
139	1	0	1.442972	-2.116259	4.910294
140	6	0	0.811560	1.897983	3.020328
141	1	0	1.207176	2.874474	3.293313
142	1	0	1.026519	1.139798	3.769189
143	7	0	-0.660957	2.073152	2.959204
144	8	0	-1.376003	1.101286	3.111301
145	8	0	-1.078292	3.194324	2.693776
146	1	0	1.553147	3.568659	-2.144259
147	6	0	0.664551	5.214470	-1.072942
148	6	0	0.665393	6.087141	-2.163661
149	6	0	0.458964	5.727047	0.208512
150	6	0	0.450883	7.450159	-1.980278
151	1	0	0.832411	5.692049	-3.162994
152	6	0	0.251971	7.092803	0.393246
153	1	0	0.451681	5.054778	1.063253
154	6	0	0.242770	7.956928	-0.698866
155	1	0	0.452184	8.116856	-2.836902
156	1	0	0.098093	7.481486	1.395354
157	1	0	0.080603	9.019957	-0.551665

G4-I-*si*-IM3

Zero-point correction= 1.30804 (a.u.)

Thermal correction to Gibbs Free Energy= 1.22739 (a.u.)

Sum of electronic and zero-point Energies= -4193.25566 (a.u.)

Sum of electronic and thermal Free Energies= -4193.33632 (a.u.)

Standard orientation:

Center Number	Atomic Number	Atomic Type	Coordinates (Angstroms) X	Y	Z
1	6	0	-3.737546	-0.553005	0.432271
2	7	0	-3.564545	1.964254	-0.288437
3	7	0	-6.350816	-0.362129	0.260251
4	6	0	-6.872937	-1.726592	0.342460
5	6	0	-8.391875	-1.683836	0.488797
6	6	0	-8.960089	-3.100278	0.611357
7	6	0	-8.535737	-3.980911	-0.566383
8	6	0	-6.464255	-2.580177	-0.860388
9	1	0	-8.816034	-1.193534	-0.399909
10	1	0	-8.661799	-1.077811	1.359872
11	1	0	-8.598764	-3.548257	1.546385
12	1	0	-10.051096	-3.055425	0.683901
13	1	0	-6.458083	-2.173678	1.257070
14	1	0	-8.918509	-4.997555	-0.432864
15	1	0	-5.372769	-2.590435	-0.962823
16	1	0	-6.865639	-2.108409	-1.767917
17	6	0	-7.013483	-4.000544	-0.723304
18	1	0	-8.985058	-3.590528	-1.489468
19	1	0	-6.722329	-4.601138	-1.590173
20	1	0	-6.562491	-4.477188	0.158790
21	1	0	-6.944637	0.334537	-0.171087
22	6	0	-4.524349	3.062487	-0.423022
23	6	0	-3.774846	4.375077	-0.647552
24	6	0	-5.529268	2.817941	-1.553644
25	1	0	-5.077561	3.152323	0.525322
26	6	0	-4.754185	5.544287	-0.775387
27	1	0	-3.173870	4.289989	-1.561882
28	1	0	-3.088702	4.541335	0.188209
29	6	0	-6.516594	3.981085	-1.668541
30	1	0	-4.979144	2.705431	-2.496588
31	1	0	-6.063342	1.874190	-1.390767
32	6	0	-5.774510	5.300496	-1.888676
33	1	0	-4.200888	6.470630	-0.959838
34	1	0	-5.282548	5.675712	0.178386
35	1	0	-7.221938	3.794958	-2.484658
36	1	0	-7.107916	4.048388	-0.745021
37	1	0	-6.484471	6.132105	-1.943381
38	1	0	-5.252506	5.260467	-2.854372
39	6	0	-3.981806	0.760344	0.038984

40	6	0	-5.031163	-0.120480	0.236679
41	1	0	-2.513076	2.125691	-0.322462
42	1	0	0.920506	-0.797833	0.469084
43	1	0	-3.342950	-3.499745	0.239495
44	1	0	-0.091727	-1.327029	2.648534
45	1	0	-0.798487	-5.282849	5.152623
46	1	0	-0.079394	-3.030484	4.412230
47	1	0	5.392633	-4.589619	3.029540
48	1	0	4.845410	-3.870747	0.728201
49	1	0	3.829739	-2.519206	-0.729499
50	1	0	1.558374	-3.504360	-5.811774
51	1	0	2.891452	-4.958521	-4.293852
52	1	0	2.930762	-4.492976	-1.867029
53	1	0	3.444622	-0.841495	-5.506570
54	1	0	5.339764	-2.435125	-5.911502
55	1	0	6.927125	-2.968529	-4.053148
56	16	0	4.604482	0.034276	-0.855767
57	9	0	6.797970	-1.997155	-1.708498
58	9	0	3.003086	0.315101	-3.252234
59	8	0	-0.999941	-3.143246	-0.403433
60	8	0	5.605580	-0.438487	0.082416
61	8	0	4.442660	1.453629	-1.115034
62	7	0	-2.894047	-1.518325	0.769625
63	7	0	3.151243	-0.555876	-0.372755
64	7	0	0.686442	-1.695257	0.049746
65	6	0	-3.404680	-2.846502	1.115080
66	6	0	-1.160583	-1.469849	2.474432
67	6	0	-1.481301	-1.220513	0.993653
68	6	0	-0.599037	-2.111671	0.114449
69	6	0	1.722553	-2.550216	-0.494100
70	6	0	3.107010	-1.998978	-0.085302
71	6	0	-2.657639	-3.488725	2.261715
72	6	0	-3.050058	-4.765137	2.669712
73	6	0	-2.396681	-5.409748	3.711862
74	6	0	-1.325239	-4.782319	4.346313
75	6	0	-0.925487	-3.517589	3.934391
76	6	0	-1.588795	-2.852703	2.898962
77	6	0	1.650665	-2.766950	-1.999360
78	6	0	2.364076	-3.844961	-2.533685
79	6	0	2.338831	-4.112220	-3.896925
80	6	0	1.592350	-3.297365	-4.746246
81	6	0	0.892454	-2.216258	-4.220511
82	6	0	0.919277	-1.944014	-2.850682
83	6	0	3.469767	-2.340273	1.348965

84	6	0	2.888902	-1.694843	2.443003
85	6	0	3.202753	-2.096071	3.740438
86	6	0	4.095770	-3.142866	3.958946
87	6	0	4.682371	-3.784079	2.871235
88	6	0	4.370325	-3.381606	1.575838
89	6	0	4.904652	-0.774421	-2.428733
90	6	0	5.938742	-1.676500	-2.675102
91	6	0	6.105684	-2.275325	-3.916701
92	6	0	5.216076	-1.970124	-4.939318
93	6	0	4.161681	-1.084799	-4.732152
94	6	0	4.023016	-0.513921	-3.481046
95	1	0	0.316578	-1.576177	-4.883307
96	1	0	4.341002	-3.449840	4.970998
97	1	0	2.751375	-1.576925	4.581458
98	1	0	2.207426	-0.863862	2.286923
99	1	0	2.369854	-0.112194	-0.882487
100	1	0	1.624082	-3.538050	-0.027123
101	1	0	-2.713527	-6.400486	4.021854
102	1	0	-3.875432	-5.256868	2.158816
103	1	0	-1.683439	-0.704835	3.057481
104	1	0	-4.457807	-2.732454	1.392733
105	1	0	0.371850	-1.097565	-2.439276
106	1	0	-1.307279	-0.164139	0.763549
107	6	0	-0.732113	3.513486	1.717131
108	1	0	-1.125443	4.462191	1.355673
109	1	0	-0.136364	3.688142	2.618808
110	7	0	-1.941092	2.754442	2.180252
111	8	0	-1.851170	1.558045	2.359131
112	8	0	-2.949565	3.410171	2.382166
113	8	0	-1.089186	0.721766	-2.015923
114	8	0	0.894321	0.749328	1.749552
115	7	0	2.343403	2.456206	1.243688
116	7	0	-0.822555	2.186364	-0.319068
117	8	0	1.007748	1.140998	-1.252781
118	1	0	4.393970	2.208227	1.284958
119	6	0	-1.207797	1.216544	-3.372077
120	6	0	0.080050	2.805934	0.614018
121	6	0	1.121228	1.844103	1.258720
122	6	0	1.004920	3.872327	0.046015
123	6	0	2.309955	3.632004	0.478785
124	6	0	0.716160	4.968241	-0.743515
125	1	0	-0.302218	5.146229	-1.081233
126	6	0	-0.208999	1.384342	-1.176103
127	6	0	1.757604	5.830989	-1.102065

128	1	0	1.554893	6.693428	-1.728228
129	6	0	3.522486	1.877152	1.856955
130	1	0	3.463541	0.793986	1.732902
131	6	0	3.354434	4.481034	0.146478
132	1	0	4.369061	4.292803	0.479813
133	6	0	3.053744	5.585697	-0.655793
134	1	0	3.854794	6.261349	-0.938577
135	6	0	3.669677	2.215783	3.324840
136	6	0	3.139827	3.384321	3.872472
137	6	0	4.380248	1.340250	4.148785
138	6	0	3.311969	3.670767	5.224922
139	1	0	2.592900	4.076934	3.237041
140	6	0	4.553887	1.626324	5.499431
141	1	0	4.790662	0.426632	3.724776
142	6	0	4.017373	2.792061	6.042432
143	1	0	2.894382	4.583302	5.639655
144	1	0	5.104308	0.934465	6.129940
145	1	0	4.148601	3.013922	7.096930
146	6	0	-1.925129	2.562263	-3.349921
147	1	0	-1.327734	3.308270	-2.817005
148	1	0	-2.099647	2.921093	-4.369315
149	1	0	-2.885079	2.457658	-2.839323
150	6	0	-2.069913	0.163093	-4.054356
151	1	0	-1.595346	-0.819661	-3.986248
152	1	0	-3.043619	0.100586	-3.557912
153	1	0	-2.229087	0.411242	-5.107842
154	6	0	0.151201	1.364819	-4.052817
155	1	0	-0.003770	1.622777	-5.105980
156	1	0	0.744281	2.154314	-3.584454
157	1	0	0.732800	0.445609	-3.992766

G4-I-*re*-TS3

Zero-point correction= 1.30347 (a.u.)

Thermal correction to Gibbs Free Energy= 1.22252 (a.u.)

Sum of electronic and zero-point Energies= -4193.26580 (a.u.)

Sum of electronic and thermal Free Energies= -4193.34676 (a.u.)

Standard orientation:

Center Number	Atomic Number	Atomic Type	Coordinates (Angstroms) X	Y	Z
1	6	0	-4.132503	0.133956	-0.370701
2	7	0	-3.074152	2.528267	-0.280506

3	7	0	-6.376796	1.206662	-1.205350
4	6	0	-7.252332	0.101936	-1.590426
5	6	0	-8.656906	0.635314	-1.859185
6	6	0	-9.607313	-0.509943	-2.216822
7	6	0	-9.083976	-1.323563	-3.402828
8	6	0	-6.724618	-0.678754	-2.797015
9	1	0	-8.608490	1.347317	-2.696252
10	1	0	-9.016359	1.183224	-0.981939
11	1	0	-9.713509	-1.168897	-1.344892
12	1	0	-10.602807	-0.111009	-2.434465
13	1	0	-7.309865	-0.576811	-0.726890
14	1	0	-9.757499	-2.160879	-3.611889
15	1	0	-5.711762	-1.046633	-2.593263
16	1	0	-6.650062	0.012129	-3.648041
17	6	0	-7.665191	-1.834475	-3.141864
18	1	0	-9.075952	-0.689616	-4.299586
19	1	0	-7.286553	-2.380929	-4.010840
20	1	0	-7.683113	-2.546317	-2.304289
21	1	0	-6.577612	2.121401	-1.587736
22	6	0	-3.673161	3.845149	-0.497515
23	6	0	-2.846054	4.908396	0.217826
24	6	0	-3.800210	4.175701	-1.988301
25	1	0	-4.685680	3.857085	-0.058533
26	6	0	-3.435161	6.305060	0.014325
27	1	0	-1.826678	4.883270	-0.192993
28	1	0	-2.770827	4.655752	1.281706
29	6	0	-4.399283	5.567835	-2.199224
30	1	0	-2.798940	4.126287	-2.438971
31	1	0	-4.406106	3.407269	-2.486155
32	6	0	-3.575385	6.634352	-1.473636
33	1	0	-2.807870	7.051068	0.513360
34	1	0	-4.423074	6.352835	0.492170
35	1	0	-4.462426	5.793784	-3.268589
36	1	0	-5.427243	5.582560	-1.810724
37	1	0	-4.032132	7.620857	-1.603537
38	1	0	-2.575683	6.684350	-1.926359
39	6	0	-3.868446	1.502140	-0.443028
40	6	0	-5.115142	0.992105	-0.796335
41	1	0	-2.025442	2.341999	0.334491
42	1	0	0.075543	-1.124781	-0.157732
43	1	0	-4.465516	-2.812416	-1.050146
44	1	0	-1.490942	-2.300974	2.158741
45	1	0	-4.451223	-5.418454	4.184797
46	1	0	-2.726590	-3.667622	3.864783

47	1	0	-1.407934	-5.907040	2.223110
48	1	0	-0.389602	-5.122399	0.133177
49	1	0	2.214544	-4.226329	-0.649430
50	1	0	3.212503	-1.102961	-5.626029
51	1	0	3.152476	-3.529585	-5.092902
52	1	0	1.936734	-4.327080	-3.095862
53	1	0	4.775524	1.086754	-3.083002
54	1	0	6.638905	-0.010224	-4.374771
55	1	0	7.408450	-2.300353	-3.724119
56	16	0	4.276445	-2.615598	0.066399
57	9	0	6.466071	-3.652996	-1.782305
58	9	0	3.575565	-0.009245	-1.101220
59	8	0	-2.129911	-3.249170	-0.971712
60	8	0	4.477753	-4.051245	-0.016046
61	8	0	4.818307	-1.850342	1.185592
62	7	0	-3.721964	-1.099200	-0.085508
63	7	0	2.679746	-2.335823	0.000260
64	7	0	-0.291265	-1.954408	-0.618907
65	6	0	-4.652965	-2.223672	-0.146916
66	6	0	-2.468777	-1.894073	1.865209
67	6	0	-2.377314	-1.317782	0.445220
68	6	0	-1.600134	-2.266447	-0.468415
69	6	0	0.630676	-2.892340	-1.231849
70	6	0	1.687760	-3.377466	-0.201810
71	6	0	-4.553369	-3.111255	1.070705
72	6	0	-5.518729	-4.106151	1.246683
73	6	0	-5.487128	-4.941708	2.354257
74	6	0	-4.481594	-4.779389	3.307782
75	6	0	-3.516596	-3.797141	3.129531
76	6	0	-3.530562	-2.959239	2.008929
77	6	0	1.314839	-2.360667	-2.476973
78	6	0	1.966634	-3.263229	-3.321362
79	6	0	2.649658	-2.815652	-4.447791
80	6	0	2.679763	-1.455644	-4.748033
81	6	0	2.036813	-0.550999	-3.907882
82	6	0	1.366210	-0.999608	-2.772705
83	6	0	1.048774	-3.833675	1.105417
84	6	0	1.501062	-3.363224	2.337789
85	6	0	0.920894	-3.812054	3.524915
86	6	0	-0.120446	-4.732171	3.492151
87	6	0	-0.584716	-5.200459	2.262698
88	6	0	-0.005399	-4.754857	1.080311
89	6	0	4.969460	-1.866521	-1.401829
90	6	0	6.008159	-2.448652	-2.126164

91	6	0	6.605494	-1.799857	-3.196162
92	6	0	6.168617	-0.526254	-3.544701
93	6	0	5.140776	0.095343	-2.842645
94	6	0	4.566448	-0.588516	-1.786798
95	1	0	2.073353	0.514055	-4.115225
96	1	0	-0.568859	-5.086119	4.415803
97	1	0	1.292031	-3.438244	4.474468
98	1	0	2.320907	-2.652407	2.375762
99	1	0	2.382912	-1.385397	0.233344
100	1	0	0.020617	-3.753662	-1.513935
101	1	0	-6.244307	-5.709061	2.479732
102	1	0	-6.303417	-4.219703	0.501525
103	1	0	-2.683564	-1.052190	2.532352
104	1	0	-5.663098	-1.807087	-0.205566
105	1	0	0.910181	-0.276949	-2.103398
106	1	0	-1.880409	-0.341887	0.489795
107	8	0	0.123381	1.508609	2.925250
108	8	0	1.347998	0.184851	0.459527
109	7	0	2.630802	1.951965	1.205859
110	7	0	-0.904664	2.067331	1.007626
111	8	0	-2.130663	1.213358	2.727574
112	6	0	0.217193	1.030970	4.286919
113	6	0	0.405933	2.465815	0.576300
114	6	0	1.494491	1.366340	0.743286
115	6	0	1.065130	3.635747	1.285969
116	6	0	2.378477	3.284710	1.599181
117	6	0	0.577655	4.875056	1.650089
118	1	0	-0.452990	5.143414	1.443656
119	6	0	-1.058207	1.575868	2.253810
120	6	0	1.430574	5.766948	2.310506
121	1	0	1.063152	6.743992	2.605892
122	6	0	3.865708	1.254521	1.507410
123	1	0	3.680137	0.183081	1.400432
124	6	0	3.245756	4.151390	2.243232
125	1	0	4.271083	3.872058	2.462586
126	6	0	2.745515	5.409429	2.593264
127	1	0	3.398103	6.113774	3.099055
128	6	0	1.722650	1.042848	4.533127
129	6	0	-0.499704	2.007149	5.211476
130	6	0	-0.323370	-0.390762	4.409307
131	1	0	-1.411850	-0.408558	4.358374
132	1	0	-0.003431	-0.820252	5.364356
133	1	0	0.082622	-1.010171	3.602659
134	1	0	1.944447	0.771209	5.568977

135	1	0	2.129764	2.040232	4.335796
136	1	0	2.216539	0.323028	3.871077
137	1	0	-0.372442	1.699330	6.253888
138	1	0	-1.565476	2.034876	4.978189
139	1	0	-0.080675	3.011316	5.091631
140	6	0	0.243350	2.716237	-0.944555
141	1	0	-0.405751	3.574679	-1.116560
142	1	0	-0.144592	1.813960	-1.417612
143	7	0	1.552402	3.024299	-1.590471
144	8	0	2.161104	2.095931	-2.095149
145	8	0	1.951990	4.173069	-1.542587
146	1	0	4.106173	1.436450	2.563121
147	6	0	5.038201	1.659066	0.632986
148	6	0	6.247564	0.978144	0.801523
149	6	0	4.948356	2.657380	-0.336855
150	6	0	7.352897	1.299035	0.019796
151	1	0	6.310269	0.176268	1.531686
152	6	0	6.060861	2.984242	-1.111634
153	1	0	4.012571	3.180583	-0.508953
154	6	0	7.265007	2.310005	-0.935497
155	1	0	8.283244	0.756224	0.155773
156	1	0	5.975183	3.766566	-1.859926
157	1	0	8.128138	2.563635	-1.543233

G4-I-*si*-TS3

Zero-point correction= 1.30387 (a.u.)
Thermal correction to Gibbs Free Energy= 1.22244 (a.u.)
Sum of electronic and zero-point Energies= -4193.25607 (a.u.)
Sum of electronic and thermal Free Energies= -4193.33750 (a.u.)

Standard orientation:

Center Number	Atomic Number	Atomic Type	Coordinates (Angstroms) X	Y	Z
1	6	0	3.665641	-0.558403	-0.494802
2	7	0	3.426168	1.949260	0.245706
3	7	0	6.275158	-0.305483	-0.324246
4	6	0	6.826630	-1.659494	-0.349239
5	6	0	8.347213	-1.588911	-0.469406
6	6	0	8.951332	-2.994020	-0.530466
7	6	0	8.526416	-3.841238	0.671392
8	6	0	6.413308	-2.484942	0.872296
9	1	0	8.743315	-1.058399	0.409177

10	1	0	8.619378	-1.006073	-1.355472
11	1	0	8.618439	-3.485326	-1.454385
12	1	0	10.042304	-2.925594	-0.585204
13	1	0	6.438747	-2.146203	-1.255760
14	1	0	8.937343	-4.851982	0.583397
15	1	0	5.320180	-2.522783	0.948696
16	1	0	6.778538	-1.971066	1.772507
17	6	0	7.002274	-3.894082	0.799549
18	1	0	8.947539	-3.405324	1.587444
19	1	0	6.708877	-4.470861	1.681918
20	1	0	6.582048	-4.414468	-0.072937
21	1	0	6.834061	0.404357	0.132631
22	6	0	4.451567	2.973633	0.464978
23	6	0	3.796987	4.348763	0.592070
24	6	0	5.330982	2.685168	1.690674
25	1	0	5.109465	3.004945	-0.420612
26	6	0	4.838386	5.447831	0.815150
27	1	0	3.086758	4.327808	1.430661
28	1	0	3.230649	4.553292	-0.321693
29	6	0	6.385254	3.775929	1.889205
30	1	0	4.692100	2.629476	2.581242
31	1	0	5.803568	1.700221	1.588457
32	6	0	5.722653	5.148112	2.026742
33	1	0	4.339604	6.415081	0.934700
34	1	0	5.469359	5.526319	-0.080472
35	1	0	6.995725	3.553344	2.770635
36	1	0	7.064632	3.789160	1.025463
37	1	0	6.481067	5.928622	2.147138
38	1	0	5.106076	5.158040	2.936059
39	6	0	3.870393	0.766650	-0.088070
40	6	0	4.942548	-0.098887	-0.304666
41	1	0	2.138738	2.080955	0.324803
42	1	0	-0.953285	-0.827308	-0.494291
43	1	0	3.333708	-3.535728	-0.327285
44	1	0	0.041107	-1.403832	-2.715957
45	1	0	0.868838	-5.283848	-5.295466
46	1	0	0.088842	-3.065366	-4.515949
47	1	0	-5.462170	-4.482236	-3.122252
48	1	0	-4.908816	-3.825637	-0.805110
49	1	0	-3.888664	-2.503562	0.687645
50	1	0	-1.517778	-3.767838	5.710576
51	1	0	-3.036840	-5.025173	4.190727
52	1	0	-3.120653	-4.454955	1.790025
53	1	0	-3.347460	-1.003876	5.455191

54	1	0	-5.346515	-2.455295	5.894535
55	1	0	-7.038604	-2.813069	4.087532
56	16	0	-4.596348	0.074140	0.871921
57	9	0	-6.915848	-1.793416	1.759389
58	9	0	-2.899423	0.174712	3.212891
59	8	0	0.922835	-3.243808	0.273085
60	8	0	-5.613755	-0.354917	-0.069407
61	8	0	-4.388899	1.483250	1.153546
62	7	0	2.842328	-1.554205	-0.824169
63	7	0	-3.159469	-0.548835	0.369409
64	7	0	-0.727505	-1.726466	-0.075704
65	6	0	3.383804	-2.862206	-1.189022
66	6	0	1.114401	-1.517444	-2.541834
67	6	0	1.429929	-1.280122	-1.057613
68	6	0	0.544205	-2.181580	-0.195615
69	6	0	-1.779549	-2.577932	0.443517
70	6	0	-3.154032	-1.987949	0.053692
71	6	0	2.661650	-3.501863	-2.353644
72	6	0	3.088772	-4.759485	-2.785223
73	6	0	2.459464	-5.399496	-3.845009
74	6	0	1.376868	-4.787053	-4.474926
75	6	0	0.942756	-3.541337	-4.040636
76	6	0	1.581240	-2.881276	-2.986773
77	6	0	-1.698049	-2.844979	1.941128
78	6	0	-2.479920	-3.883144	2.459131
79	6	0	-2.426795	-4.212762	3.807658
80	6	0	-1.577806	-3.506846	4.658193
81	6	0	-0.805477	-2.467679	4.149474
82	6	0	-0.867058	-2.127367	2.796430
83	6	0	-3.523817	-2.287965	-1.387772
84	6	0	-2.936751	-1.622111	-2.466118
85	6	0	-3.252398	-1.989921	-3.772983
86	6	0	-4.154494	-3.023139	-4.016744
87	6	0	-4.746686	-3.685359	-2.944788
88	6	0	-4.431748	-3.316972	-1.639920
89	6	0	-4.904287	-0.750722	2.434569
90	6	0	-5.998376	-1.572644	2.699566
91	6	0	-6.167616	-2.187043	3.933279
92	6	0	-5.220495	-1.978062	4.928647
93	6	0	-4.107673	-1.172446	4.702479
94	6	0	-3.970888	-0.581949	3.460797
95	1	0	-0.143216	-1.916851	4.811734
96	1	0	-4.400749	-3.304723	-5.035871
97	1	0	-2.793136	-1.456872	-4.600839

98	1	0	-2.243829	-0.804590	-2.291675
99	1	0	-2.379969	-0.161698	0.921149
100	1	0	-1.700447	-3.553693	-0.052249
101	1	0	2.804334	-6.374929	-4.173239
102	1	0	3.923633	-5.240056	-2.279089
103	1	0	1.616268	-0.730639	-3.113933
104	1	0	4.436684	-2.720449	-1.455245
105	1	0	-0.263552	-1.312387	2.401957
106	1	0	1.242386	-0.229131	-0.819716
107	6	0	0.858963	3.480115	-1.651943
108	1	0	1.321567	4.377591	-1.244708
109	1	0	0.242414	3.757368	-2.512519
110	7	0	1.996012	2.684330	-2.224762
111	8	0	1.855406	1.489044	-2.375832
112	8	0	2.983967	3.318638	-2.546096
113	8	0	1.069986	0.649342	2.086600
114	8	0	-0.871443	0.757831	-1.733636
115	7	0	-2.254802	2.513031	-1.209095
116	7	0	0.867721	2.084348	0.358955
117	8	0	-0.999295	1.152979	1.300904
118	1	0	-4.312199	2.324026	-1.224339
119	6	0	1.187416	1.169962	3.440557
120	6	0	0.017878	2.771052	-0.578006
121	6	0	-1.057968	1.856619	-1.238129
122	6	0	-0.865767	3.860742	0.011456
123	6	0	-2.177584	3.675209	-0.426719
124	6	0	-0.533386	4.939423	0.807922
125	1	0	0.491908	5.078981	1.143207
126	6	0	0.221491	1.316115	1.241293
127	6	0	-1.541856	5.836900	1.174659
128	1	0	-1.306744	6.686483	1.806683
129	6	0	-3.459186	1.976721	-1.814311
130	1	0	-3.427752	0.890617	-1.706691
131	6	0	-3.188809	4.560442	-0.087133
132	1	0	-4.208638	4.417326	-0.426868
133	6	0	-2.846419	5.644351	0.726656
134	1	0	-3.620850	6.347868	1.015351
135	6	0	-3.615758	2.341616	-3.274565
136	6	0	-3.068714	3.507831	-3.810008
137	6	0	-4.355156	1.494458	-4.102690
138	6	0	-3.253366	3.820512	-5.154871
139	1	0	-2.497864	4.177982	-3.171560
140	6	0	-4.541252	1.806836	-5.445744
141	1	0	-4.778193	0.582276	-3.688195

142	6	0	-3.988587	2.970517	-5.976619
143	1	0	-2.822629	4.730907	-5.560653
144	1	0	-5.114399	1.137110	-6.079762
145	1	0	-4.129909	3.212795	-7.025271
146	6	0	1.741193	2.589222	3.380723
147	1	0	1.003992	3.284228	2.965667
148	1	0	2.007087	2.933865	4.384822
149	1	0	2.630971	2.605972	2.748044
150	6	0	2.187172	0.221673	4.085599
151	1	0	1.818967	-0.807464	4.033429
152	1	0	3.144273	0.267390	3.556852
153	1	0	2.349931	0.486879	5.134021
154	6	0	-0.151956	1.153327	4.168702
155	1	0	0.005134	1.454397	5.209921
156	1	0	-0.864521	1.841292	3.709367
157	1	0	-0.596488	0.158298	4.151592

G4-I-*re*-IM4

Zero-point correction= 1.30657 (a.u.)

Thermal correction to Gibbs Free Energy= 1.22340 (a.u.)

Sum of electronic and zero-point Energies= -4193.26784 (a.u.)

Sum of electronic and thermal Free Energies= -4193.35101 (a.u.)

Standard orientation:

Center Number	Atomic Number	Atomic Type	Coordinates (Angstroms) X	Y	Z
1	6	0	-4.148966	0.324212	-0.332868
2	7	0	-2.916281	2.623929	-0.400873
3	7	0	-6.340145	1.440482	-1.244028
4	6	0	-7.221289	0.334204	-1.613766
5	6	0	-8.613328	0.876061	-1.928366
6	6	0	-9.574451	-0.265087	-2.270872
7	6	0	-9.039518	-1.122684	-3.419993
8	6	0	-6.683367	-0.498617	-2.780553
9	1	0	-8.538326	1.560851	-2.786171
10	1	0	-8.980729	1.457741	-1.076638
11	1	0	-9.709444	-0.895615	-1.381786
12	1	0	-10.559269	0.141193	-2.521956
13	1	0	-7.304912	-0.313271	-0.728942
14	1	0	-9.722999	-1.954298	-3.620102
15	1	0	-5.683280	-0.878213	-2.540324
16	1	0	-6.575299	0.160759	-3.653452

17	6	0	-7.636054	-1.648584	-3.111374
18	1	0	-9.000387	-0.515454	-4.334425
19	1	0	-7.247978	-2.228961	-3.954081
20	1	0	-7.685405	-2.333368	-2.252717
21	1	0	-6.454829	2.293717	-1.777026
22	6	0	-3.402302	3.948856	-0.782603
23	6	0	-2.832166	4.998464	0.172807
24	6	0	-3.032968	4.292526	-2.231266
25	1	0	-4.504464	3.991727	-0.702100
26	6	0	-3.277872	6.413238	-0.197649
27	1	0	-1.734676	4.943510	0.121829
28	1	0	-3.115254	4.743417	1.200317
29	6	0	-3.480133	5.706137	-2.610302
30	1	0	-1.941935	4.210110	-2.339849
31	1	0	-3.471292	3.546742	-2.905360
32	6	0	-2.902693	6.743147	-1.643906
33	1	0	-2.834172	7.141547	0.489396
34	1	0	-4.367243	6.491476	-0.079364
35	1	0	-3.184321	5.934519	-3.639712
36	1	0	-4.577477	5.758836	-2.577936
37	1	0	-3.248848	7.747451	-1.910029
38	1	0	-1.807724	6.748685	-1.737318
39	6	0	-3.790084	1.673807	-0.506501
40	6	0	-5.074333	1.195202	-0.827069
41	1	0	-1.648649	2.283614	0.729896
42	1	0	-0.020283	-1.101772	-0.147424
43	1	0	-4.722554	-2.546054	-0.893794
44	1	0	-1.506696	-2.276331	2.130291
45	1	0	-4.463714	-5.327717	4.252211
46	1	0	-2.700982	-3.623194	3.889062
47	1	0	-1.618838	-5.956448	2.013911
48	1	0	-0.619415	-5.118462	-0.065447
49	1	0	1.964482	-4.251311	-0.888946
50	1	0	2.885555	-0.881323	-5.733665
51	1	0	2.716021	-3.333226	-5.357027
52	1	0	1.556373	-4.199434	-3.355671
53	1	0	4.673626	1.091490	-3.071291
54	1	0	6.438292	-0.006056	-4.497019
55	1	0	7.134426	-2.357881	-4.003492
56	16	0	4.112114	-2.765083	-0.130306
57	9	0	6.204456	-3.781288	-2.105266
58	9	0	3.506449	-0.065000	-1.105789
59	8	0	-2.354710	-3.063851	-1.002967
60	8	0	4.257315	-4.200074	-0.294921

61	8	0	4.699650	-2.084237	1.020438
62	7	0	-3.819582	-0.906724	0.077860
63	7	0	2.526952	-2.419151	-0.154548
64	7	0	-0.440726	-1.884499	-0.641070
65	6	0	-4.801005	-1.983714	0.043161
66	6	0	-2.481034	-1.812923	1.919676
67	6	0	-2.460193	-1.193374	0.514358
68	6	0	-1.761646	-2.136965	-0.467676
69	6	0	0.415693	-2.824038	-1.339393
70	6	0	1.487352	-3.409371	-0.377167
71	6	0	-4.653590	-2.924266	1.214527
72	6	0	-5.638837	-3.895914	1.412594
73	6	0	-5.575691	-4.765891	2.491587
74	6	0	-4.517940	-4.662942	3.395619
75	6	0	-3.531829	-3.707167	3.193169
76	6	0	-3.575870	-2.838794	2.096915
77	6	0	1.075378	-2.249655	-2.578782
78	6	0	1.636262	-3.125801	-3.511826
79	6	0	2.287753	-2.638950	-4.640740
80	6	0	2.379987	-1.265271	-4.852816
81	6	0	1.830018	-0.387131	-3.922744
82	6	0	1.189701	-0.876327	-2.787024
83	6	0	0.873370	-3.909470	0.925924
84	6	0	1.366877	-3.504495	2.165829
85	6	0	0.801731	-3.988850	3.346489
86	6	0	-0.266759	-4.876863	3.299194
87	6	0	-0.773316	-5.277474	2.062916
88	6	0	-0.206882	-4.799191	0.887241
89	6	0	4.801927	-1.959836	-1.569168
90	6	0	5.788188	-2.541333	-2.363504
91	6	0	6.373039	-1.856404	-3.417995
92	6	0	5.977520	-0.548705	-3.678680
93	6	0	5.004194	0.073483	-2.902999
94	6	0	4.442526	-0.645383	-1.864507
95	1	0	1.912641	0.686201	-4.063731
96	1	0	-0.704990	-5.257066	4.217196
97	1	0	1.205879	-3.668947	4.302400
98	1	0	2.208967	-2.820769	2.213707
99	1	0	2.278565	-1.480047	0.161742
100	1	0	-0.242696	-3.641613	-1.643895
101	1	0	-6.348778	-5.514214	2.634101
102	1	0	-6.466270	-3.960292	0.708970
103	1	0	-2.617070	-0.993447	2.633395
104	1	0	-5.791593	-1.519310	0.088430

105	1	0	0.801768	-0.178335	-2.051992
106	1	0	-1.922969	-0.239201	0.551973
107	8	0	0.242463	1.333222	3.125227
108	8	0	1.341131	0.128225	0.537564
109	7	0	2.713306	1.810069	1.336306
110	7	0	-0.790308	2.039222	1.292524
111	8	0	-2.037329	1.268904	3.038276
112	6	0	0.367161	0.831242	4.484033
113	6	0	0.500836	2.429440	0.794720
114	6	0	1.548713	1.284185	0.875568
115	6	0	1.208260	3.538512	1.543200
116	6	0	2.515516	3.130568	1.803318
117	6	0	0.759620	4.771144	1.972810
118	1	0	-0.269197	5.075238	1.802540
119	6	0	-0.955349	1.526653	2.544386
120	6	0	1.653879	5.605544	2.652566
121	1	0	1.323280	6.578092	3.000634
122	6	0	3.933284	1.056551	1.557449
123	1	0	3.701764	-0.001752	1.413559
124	6	0	3.421844	3.942199	2.465131
125	1	0	4.444165	3.626246	2.645456
126	6	0	2.963899	5.195942	2.884953
127	1	0	3.648051	5.857698	3.406178
128	6	0	1.877367	0.705443	4.645930
129	6	0	-0.209951	1.859712	5.448008
130	6	0	-0.297025	-0.535163	4.616744
131	1	0	-1.384214	-0.453708	4.617091
132	1	0	0.023766	-1.001318	5.553714
133	1	0	0.014259	-1.181966	3.789730
134	1	0	2.123039	0.380776	5.660624
135	1	0	2.360939	1.670348	4.460913
136	1	0	2.272924	-0.032508	3.939750
137	1	0	-0.041370	1.535776	6.479181
138	1	0	-1.283301	1.975890	5.287662
139	1	0	0.280604	2.827346	5.302841
140	6	0	0.285052	2.759518	-0.701340
141	1	0	-0.326238	3.654593	-0.809165
142	1	0	-0.165979	1.908673	-1.212532
143	7	0	1.592909	3.041860	-1.364364
144	8	0	2.138702	2.119172	-1.943588
145	8	0	2.046259	4.166354	-1.260525
146	1	0	4.220766	1.185077	2.609174
147	6	0	5.084489	1.454138	0.652836
148	6	0	6.256281	0.694241	0.716585

149	6	0	5.012975	2.522495	-0.240758
150	6	0	7.343189	1.003756	-0.094567
151	1	0	6.300956	-0.159743	1.386394
152	6	0	6.107448	2.835885	-1.046432
153	1	0	4.105656	3.112742	-0.331005
154	6	0	7.274033	2.080946	-0.976233
155	1	0	8.243414	0.399330	-0.041273
156	1	0	6.037431	3.671581	-1.736276
157	1	0	8.122242	2.324593	-1.608451

G4-I-*si*-IM4

Zero-point correction= 1.30723 (a.u.)

Thermal correction to Gibbs Free Energy= 1.22531 (a.u.)

Sum of electronic and zero-point Energies= -4193.25479 (a.u.)

Sum of electronic and thermal Free Energies= -4193.33671 (a.u.)

Standard orientation:

Center Number	Atomic Number	Atomic Type	Coordinates (Angstroms) X	Y	Z
1	6	0	3.635685	-0.670196	-0.714537
2	7	0	3.641276	1.892807	-0.144673
3	7	0	6.251897	-0.710976	-0.498460
4	6	0	6.586812	-2.110271	-0.225137
5	6	0	8.101743	-2.289312	-0.281736
6	6	0	8.487710	-3.748703	-0.028508
7	6	0	7.921259	-4.255009	1.299900
8	6	0	6.026227	-2.603609	1.112334
9	1	0	8.558591	-1.652478	0.490650
10	1	0	8.475361	-1.944916	-1.251493
11	1	0	8.097898	-4.368646	-0.846974
12	1	0	9.577104	-3.853061	-0.045687
13	1	0	6.143896	-2.708008	-1.033962
14	1	0	8.179118	-5.309424	1.443421
15	1	0	4.937303	-2.475496	1.131698
16	1	0	6.438192	-1.968947	1.910480
17	6	0	6.404131	-4.064373	1.361026
18	1	0	8.386417	-3.698321	2.124843
19	1	0	6.011832	-4.395761	2.327424
20	1	0	5.929038	-4.694213	0.595576
21	1	0	6.861371	-0.031424	-0.057309
22	6	0	4.795455	2.758975	0.112603
23	6	0	4.361011	4.222024	0.039782

24	6	0	5.471954	2.462000	1.459091
25	1	0	5.555025	2.604168	-0.675791
26	6	0	5.516343	5.181155	0.335891
27	1	0	3.550037	4.378448	0.767810
28	1	0	3.951246	4.411897	-0.958959
29	6	0	6.641863	3.410335	1.728874
30	1	0	4.726920	2.567625	2.258475
31	1	0	5.803114	1.415040	1.481920
32	6	0	6.175738	4.866553	1.679833
33	1	0	5.160710	6.216770	0.316213
34	1	0	6.267795	5.091277	-0.460359
35	1	0	7.098544	3.185019	2.698511
36	1	0	7.419728	3.257395	0.967621
37	1	0	7.016849	5.544742	1.858710
38	1	0	5.450540	5.037193	2.487848
39	6	0	3.969661	0.661449	-0.393532
40	6	0	4.946429	-0.337060	-0.536283
41	1	0	1.985429	2.112703	0.099003
42	1	0	-1.040098	-0.754307	-0.495554
43	1	0	3.237941	-3.556412	-0.348416
44	1	0	-0.230764	-1.433808	-2.613012
45	1	0	-0.050452	-5.698398	-4.638323
46	1	0	-0.552175	-3.327917	-4.111057
47	1	0	-5.109466	-4.654540	-2.891616
48	1	0	-4.598733	-3.950661	-0.578145
49	1	0	-3.935734	-2.297791	0.915910
50	1	0	-1.420297	-3.188033	6.000594
51	1	0	-3.015383	-4.514588	4.625490
52	1	0	-3.164401	-4.121125	2.192644
53	1	0	-3.169999	-0.383323	5.636125
54	1	0	-5.156265	-1.772305	6.288932
55	1	0	-6.925215	-2.276331	4.595095
56	16	0	-4.620262	0.292374	1.033733
57	9	0	-6.897877	-1.474080	2.182616
58	9	0	-2.812574	0.585042	3.278357
59	8	0	0.882503	-3.036107	0.513730
60	8	0	-5.673174	-0.222574	0.179271
61	8	0	-4.412490	1.720758	1.184026
62	7	0	2.726716	-1.614089	-0.979050
63	7	0	-3.201778	-0.360373	0.513621
64	7	0	-0.781147	-1.559422	0.069189
65	6	0	3.164456	-2.975441	-1.274773
66	6	0	0.840047	-1.629778	-2.512259
67	6	0	1.319507	-1.275126	-1.096288

68	6	0	0.482933	-2.042231	-0.069838
69	6	0	-1.819836	-2.378024	0.670251
70	6	0	-3.197979	-1.811690	0.262104
71	6	0	2.246108	-3.701033	-2.233033
72	6	0	2.514450	-5.041220	-2.517857
73	6	0	1.704597	-5.759092	-3.388324
74	6	0	0.598805	-5.140900	-3.970423
75	6	0	0.321324	-3.810919	-3.679907
76	6	0	1.143599	-3.074881	-2.822435
77	6	0	-1.704170	-2.537725	2.179923
78	6	0	-2.491302	-3.515746	2.797223
79	6	0	-2.401376	-3.746905	4.164484
80	6	0	-1.509408	-3.002126	4.934430
81	6	0	-0.730722	-2.022964	4.326700
82	6	0	-0.829182	-1.781851	2.955062
83	6	0	-3.553114	-2.165731	-1.171794
84	6	0	-3.140165	-1.387957	-2.252839
85	6	0	-3.435301	-1.778581	-3.557782
86	6	0	-4.140208	-2.955703	-3.794172
87	6	0	-4.552181	-3.739159	-2.717761
88	6	0	-4.262808	-3.342777	-1.415687
89	6	0	-4.849424	-0.397888	2.671303
90	6	0	-5.937698	-1.179656	3.056789
91	6	0	-6.057505	-1.676334	4.347888
92	6	0	-5.068080	-1.386737	5.278985
93	6	0	-3.961213	-0.614842	4.933718
94	6	0	-3.874101	-0.142426	3.638515
95	1	0	-0.031937	-1.444889	4.925221
96	1	0	-4.375929	-3.257226	-4.810145
97	1	0	-3.118260	-1.147790	-4.384764
98	1	0	-2.586120	-0.471117	-2.085618
99	1	0	-2.398224	0.053669	1.004176
100	1	0	-1.751963	-3.385650	0.239196
101	1	0	1.926857	-6.799758	-3.602948
102	1	0	3.366260	-5.524462	-2.043948
103	1	0	1.357432	-0.961136	-3.208069
104	1	0	4.162621	-2.908628	-1.722918
105	1	0	-0.216342	-1.018729	2.481301
106	1	0	1.210451	-0.198755	-0.942495
107	6	0	1.165709	3.471300	-1.910217
108	1	0	1.811671	4.217998	-1.447265
109	1	0	0.620934	3.940707	-2.733600
110	7	0	2.110116	2.505968	-2.565307
111	8	0	1.828936	1.326205	-2.581603

112	8	0	3.084053	3.006306	-3.091288
113	8	0	1.039175	0.877880	1.966475
114	8	0	-0.875187	0.859525	-1.779882
115	7	0	-2.071544	2.801794	-1.558306
116	7	0	0.924289	2.193561	0.146861
117	8	0	-0.992331	1.511101	1.163328
118	1	0	-4.141083	2.851678	-1.508000
119	6	0	1.286358	1.525167	3.254499
120	6	0	0.196885	2.906217	-0.865665
121	6	0	-0.944931	2.032414	-1.465719
122	6	0	-0.596963	4.111649	-0.386026
123	6	0	-1.903095	4.018284	-0.870023
124	6	0	-0.186075	5.211758	0.341652
125	1	0	0.833181	5.276055	0.715351
126	6	0	0.229462	1.540288	1.098480
127	6	0	-1.112746	6.227814	0.596856
128	1	0	-0.819894	7.095159	1.178294
129	6	0	-3.353120	2.202489	-1.894655
130	1	0	-3.426502	1.261616	-1.340614
131	6	0	-2.830288	5.022729	-0.640804
132	1	0	-3.845962	4.959369	-1.014260
133	6	0	-2.411936	6.127609	0.106169
134	1	0	-3.122722	6.922923	0.305965
135	6	0	-3.567411	1.924819	-3.366547
136	6	0	-2.562224	2.047962	-4.322257
137	6	0	-4.827449	1.463113	-3.759889
138	6	0	-2.814580	1.727658	-5.656382
139	1	0	-1.574930	2.389965	-4.028154
140	6	0	-5.080881	1.144932	-5.088572
141	1	0	-5.605722	1.337075	-3.010676
142	6	0	-4.073281	1.278916	-6.043694
143	1	0	-2.022249	1.828757	-6.391693
144	1	0	-6.063677	0.787174	-5.379270
145	1	0	-4.268969	1.030697	-7.082215
146	6	0	1.864255	2.915717	3.012765
147	1	0	1.101751	3.602922	2.631065
148	1	0	2.252472	3.326173	3.949701
149	1	0	2.676273	2.854126	2.283828
150	6	0	2.312489	0.608584	3.900738
151	1	0	1.913200	-0.406776	3.983403
152	1	0	3.220043	0.570292	3.290384
153	1	0	2.571353	0.968649	4.900264
154	6	0	0.001001	1.606090	4.068037
155	1	0	0.226874	2.014764	5.058386

156	1	0	-0.733935	2.252856	3.583881
157	1	0	-0.449895	0.619836	4.186531
